# Supplementary figures and images for: Multi-tiered actions of Legionella effectors to modulate host Rab10 dynamics (part 1 of 2)
Source: eLife. 2024 May 21;12:RP89002. doi: 10.7554/eLife.89002 (PMC11108646; doi:10.7554/eLife.89002)

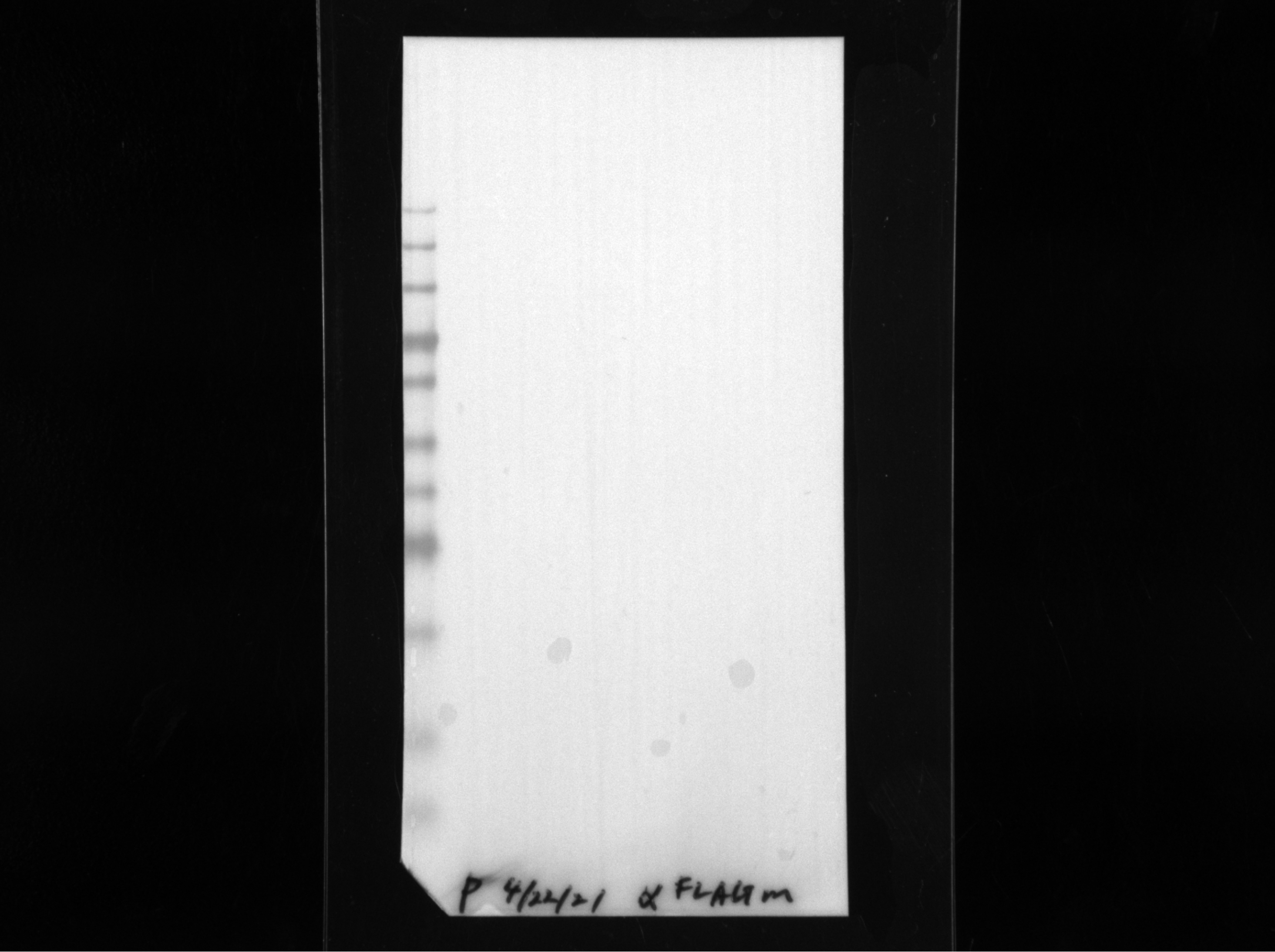

Supplement: Figure 1—source data 1. [file elife-89002-fig1-data1.zip › anti-FLAGm Marker.jpg]

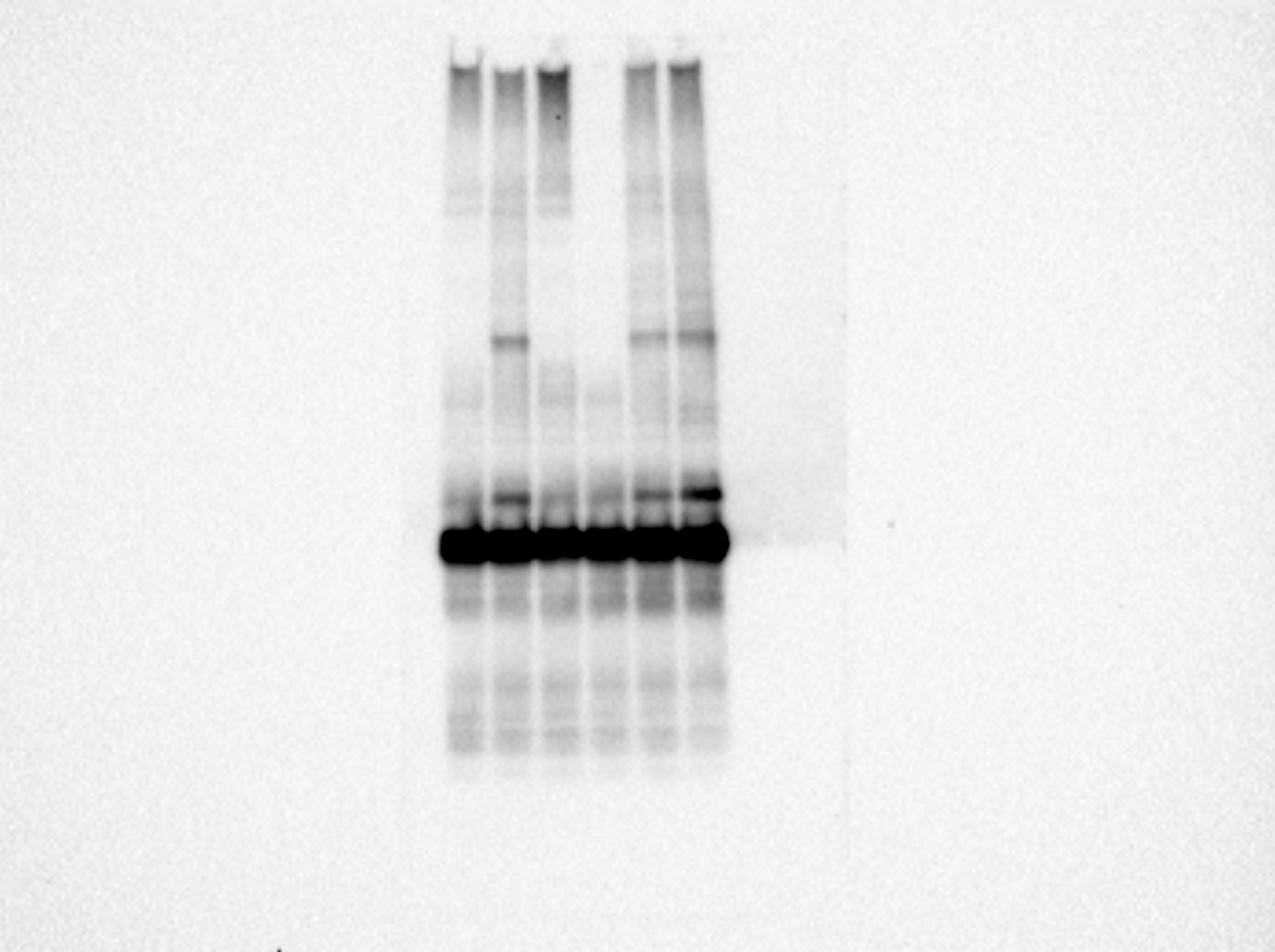

Supplement: Figure 1—source data 1. [file elife-89002-fig1-data1.zip › anti-FLAGm_Exposure_41.4sec.jpg]

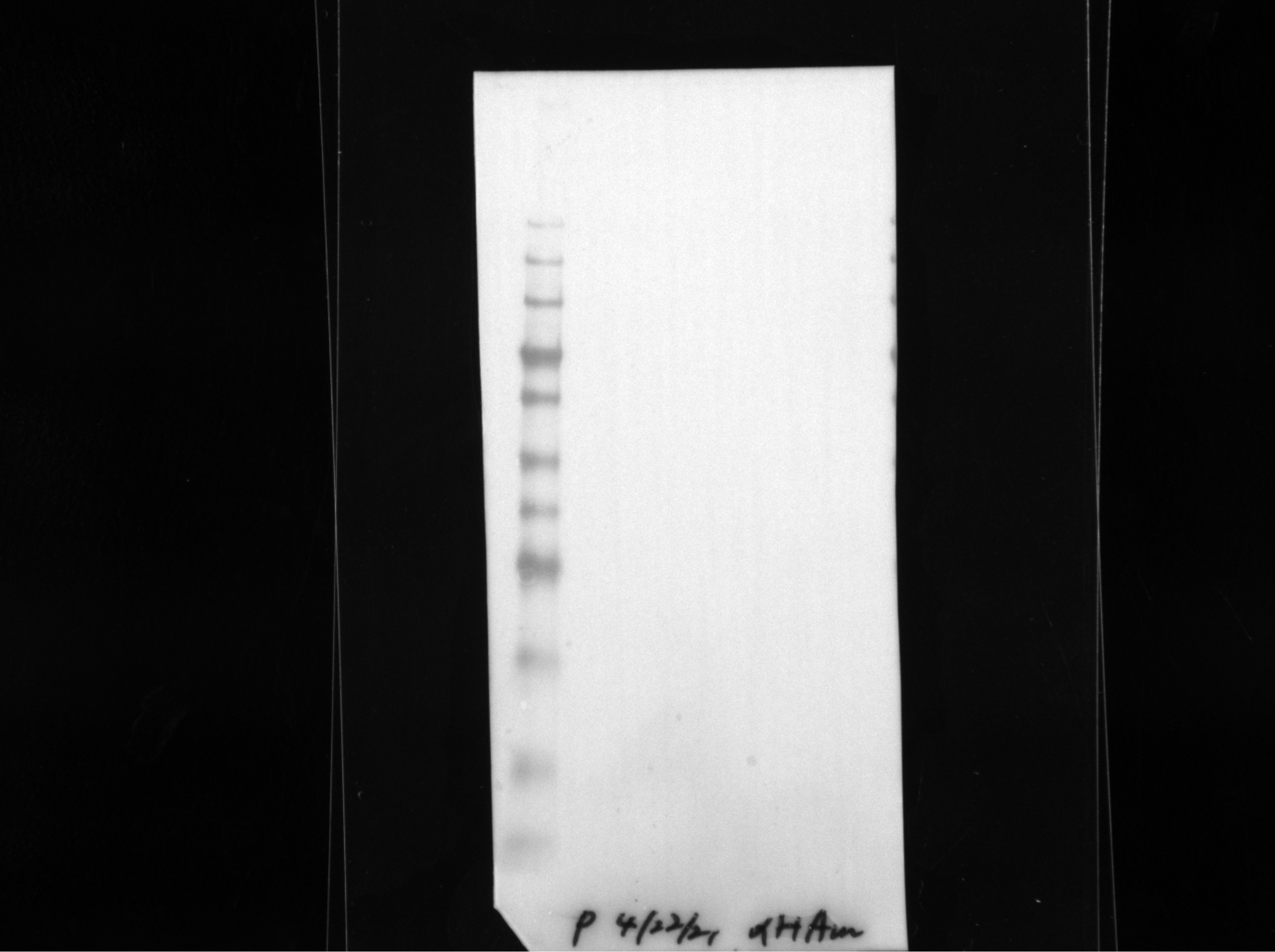

Supplement: Figure 1—source data 1. [file elife-89002-fig1-data1.zip › anti-HAm Marker.jpg]

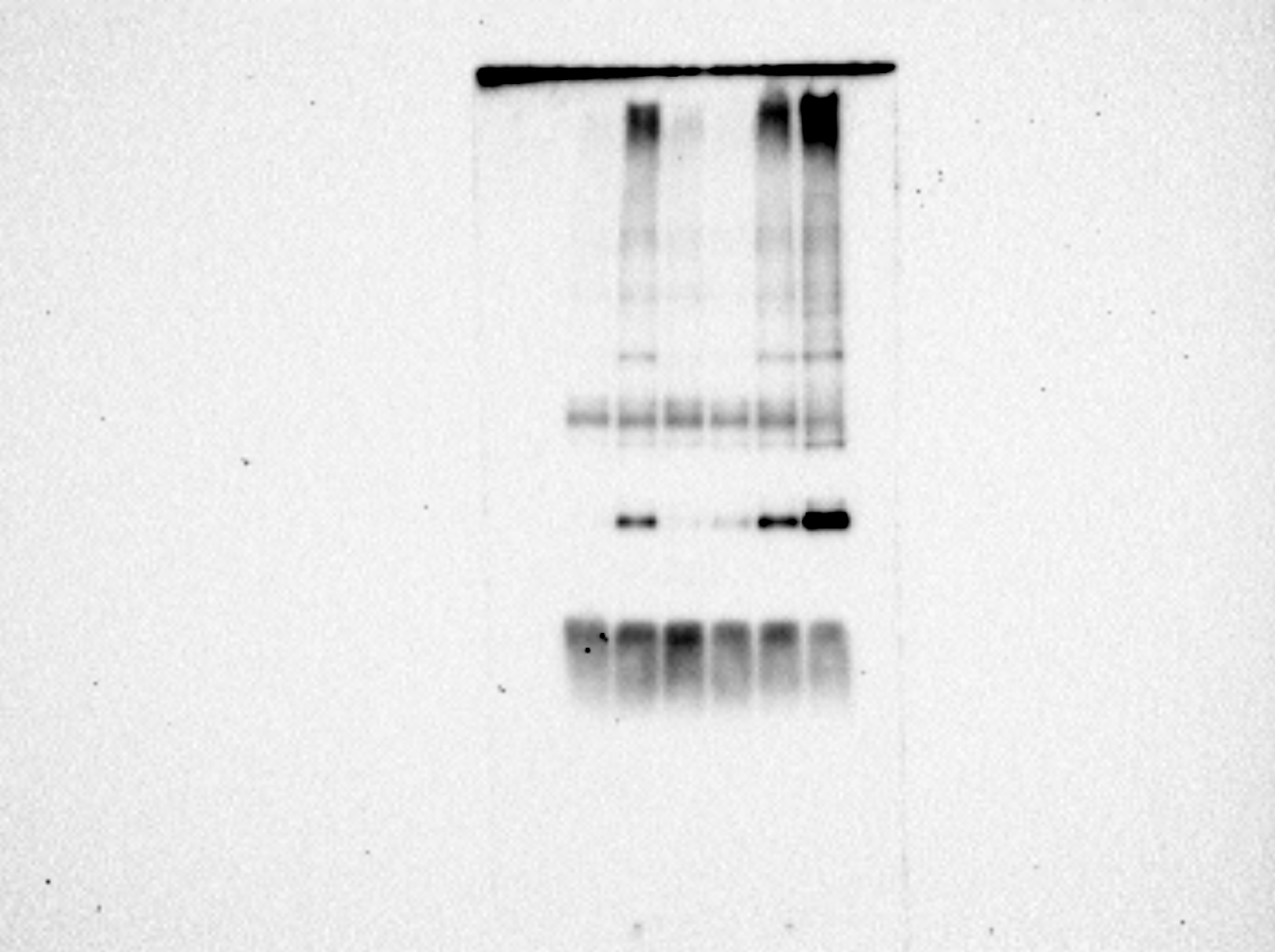

Supplement: Figure 1—source data 1. [file elife-89002-fig1-data1.zip › anti-HAm_Exposure_284.2sec.jpg]

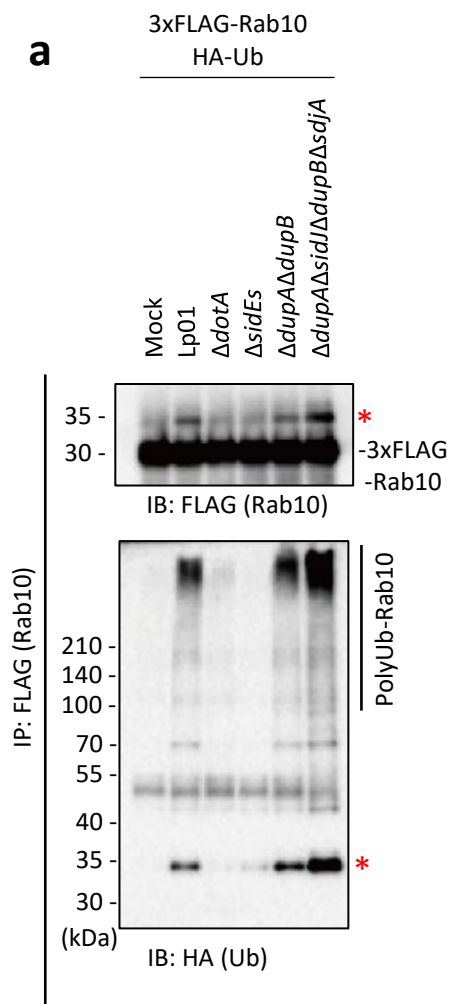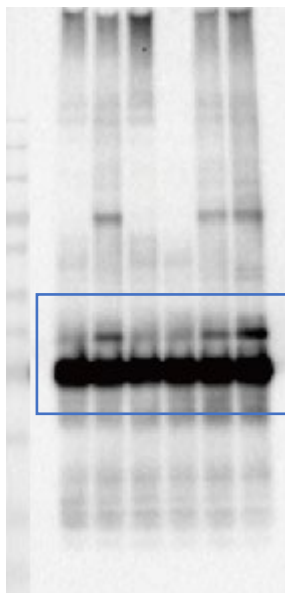

**Figure 1a**  
**top**

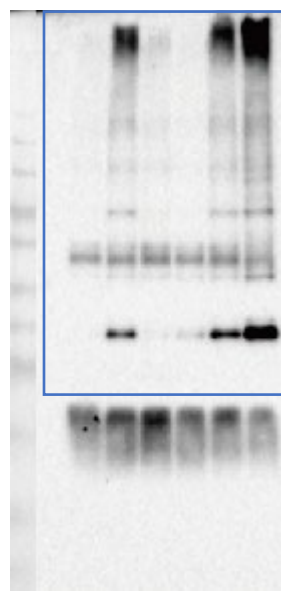

**Figure 1a**  
**bottom**

Supplement: Figure 1—source data 2. [file elife-89002-fig1-data2.pdf]

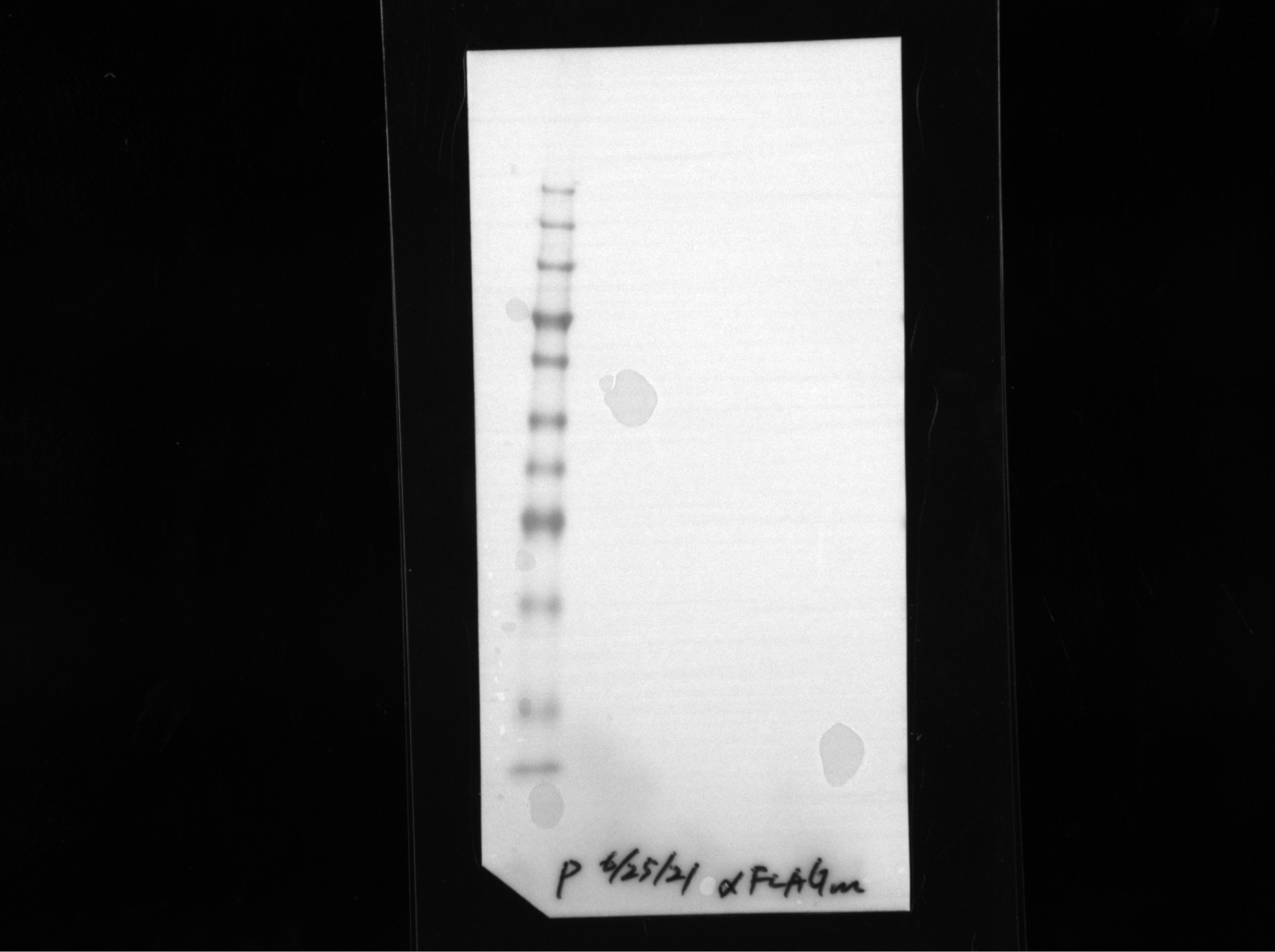

Supplement: Figure 1—source data 3. [file elife-89002-fig1-data3.zip › anti-FLAGm Marker.jpg]

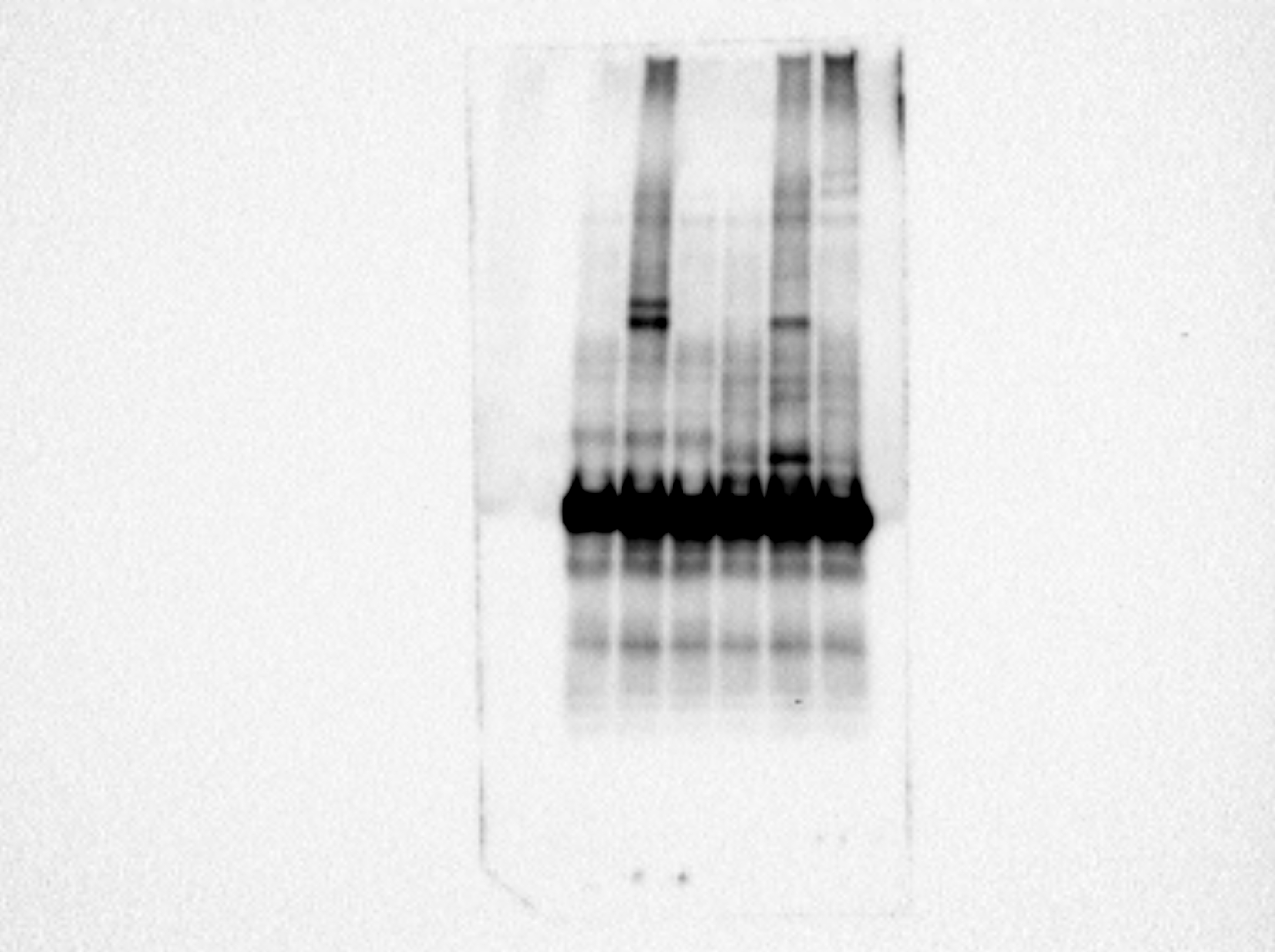

Supplement: Figure 1—source data 3. [file elife-89002-fig1-data3.zip › anti-FLAGm_Exposure_35.2sec.jpg]

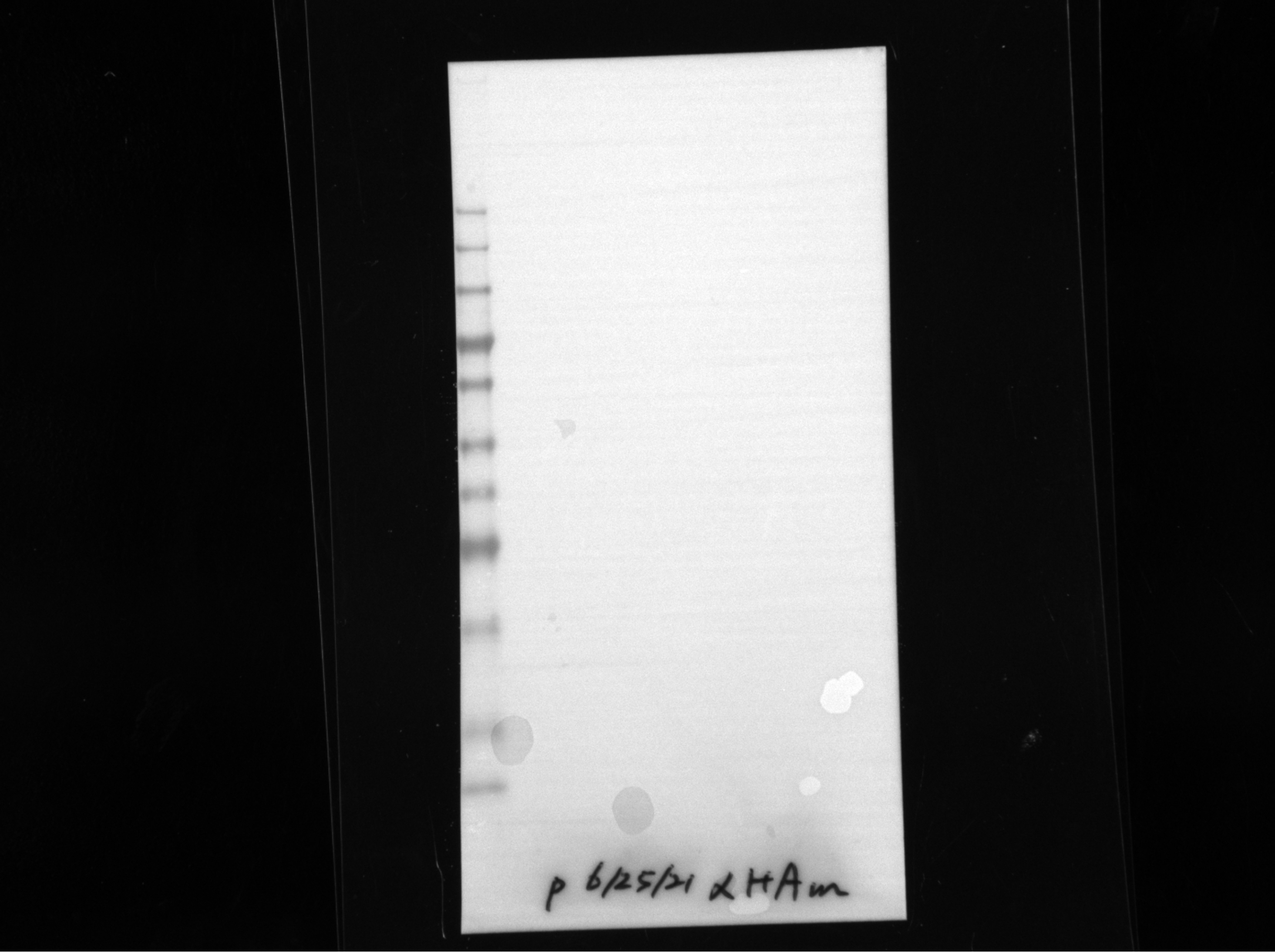

Supplement: Figure 1—source data 3. [file elife-89002-fig1-data3.zip › anti-HAm Marker.jpg]

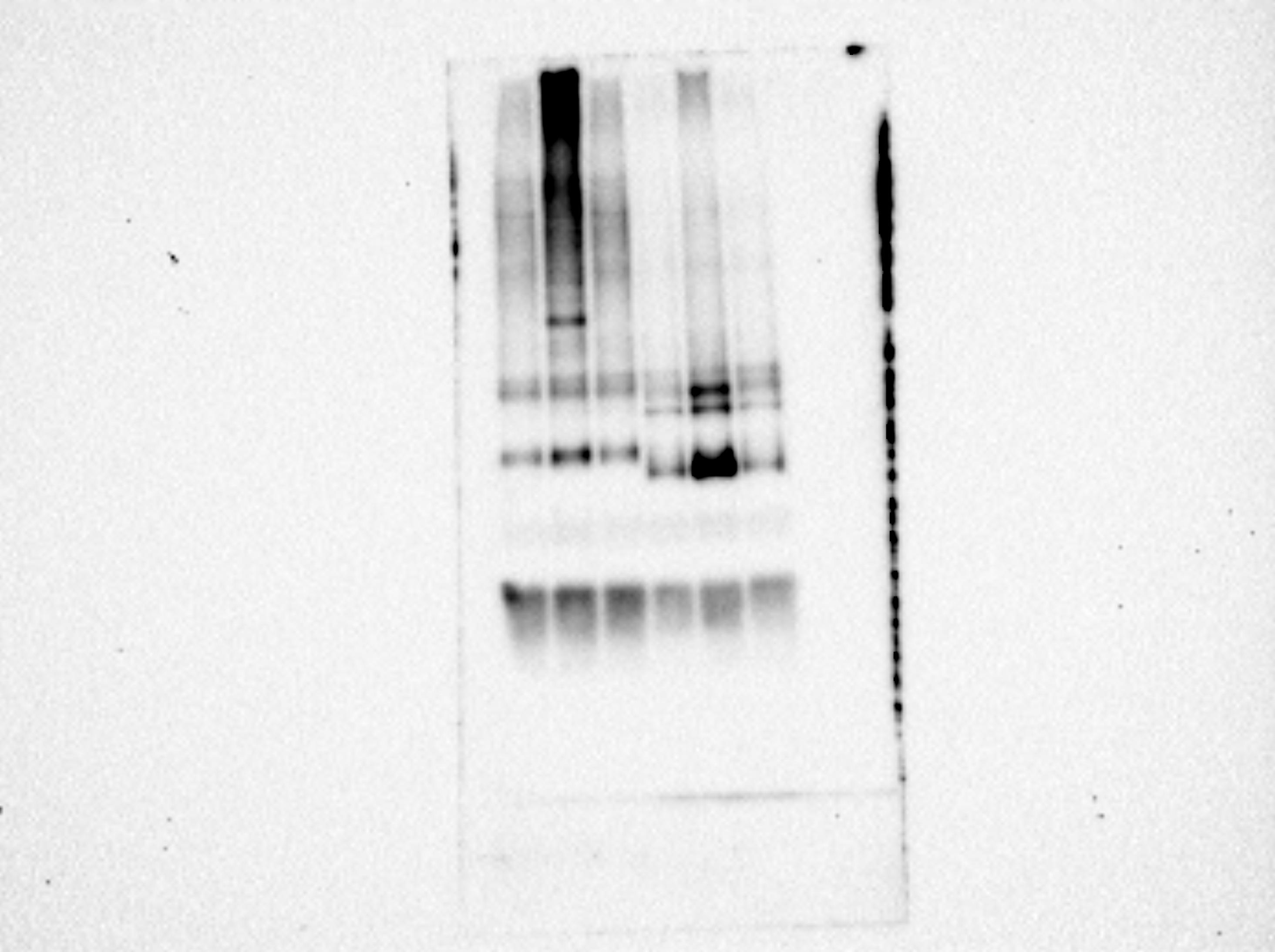

Supplement: Figure 1—source data 3. [file elife-89002-fig1-data3.zip › anti-HAm_Exposure_151.7sec.jpg]

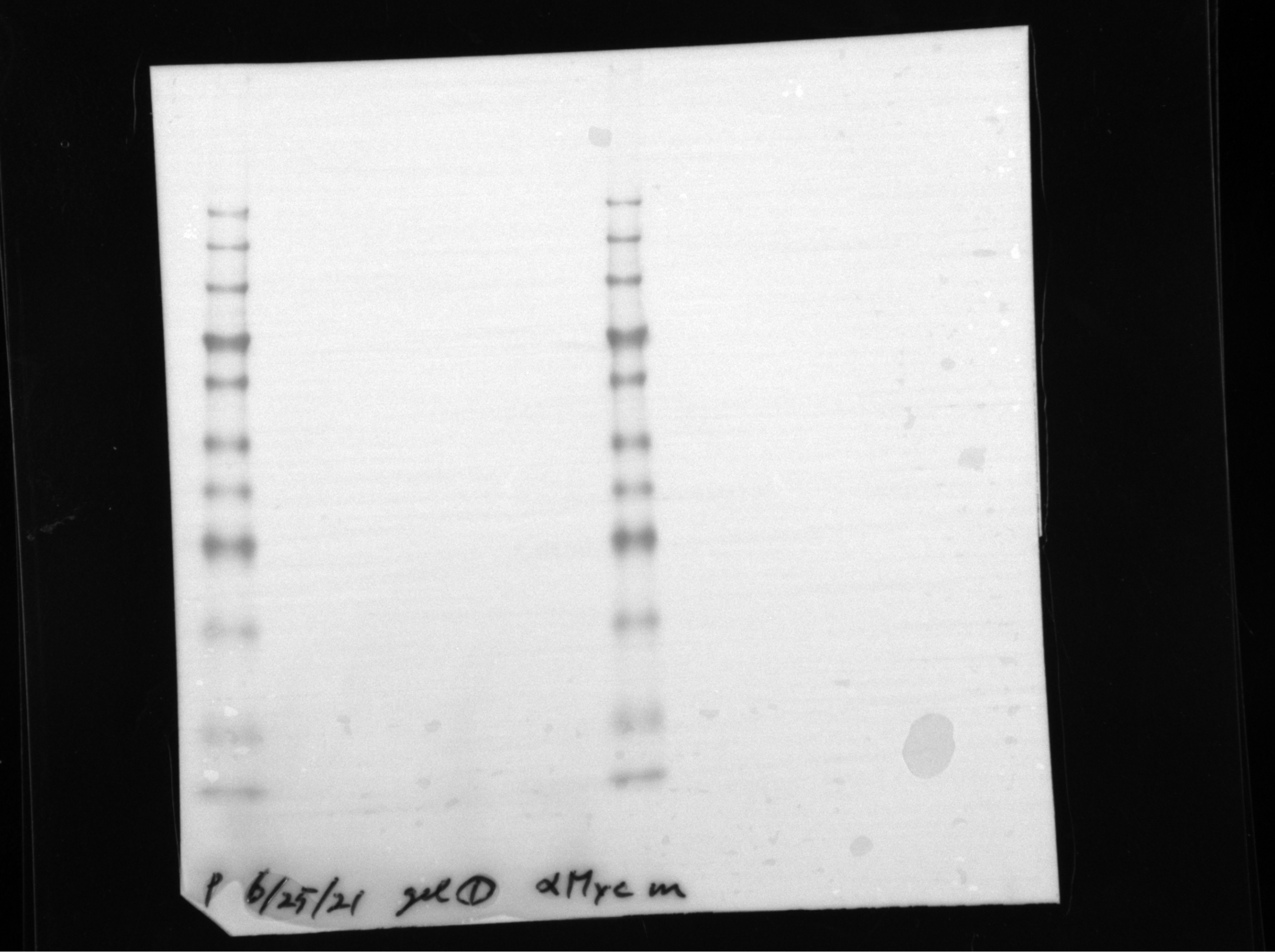

Supplement: Figure 1—source data 3. [file elife-89002-fig1-data3.zip › anti-Myc Marker.jpg]

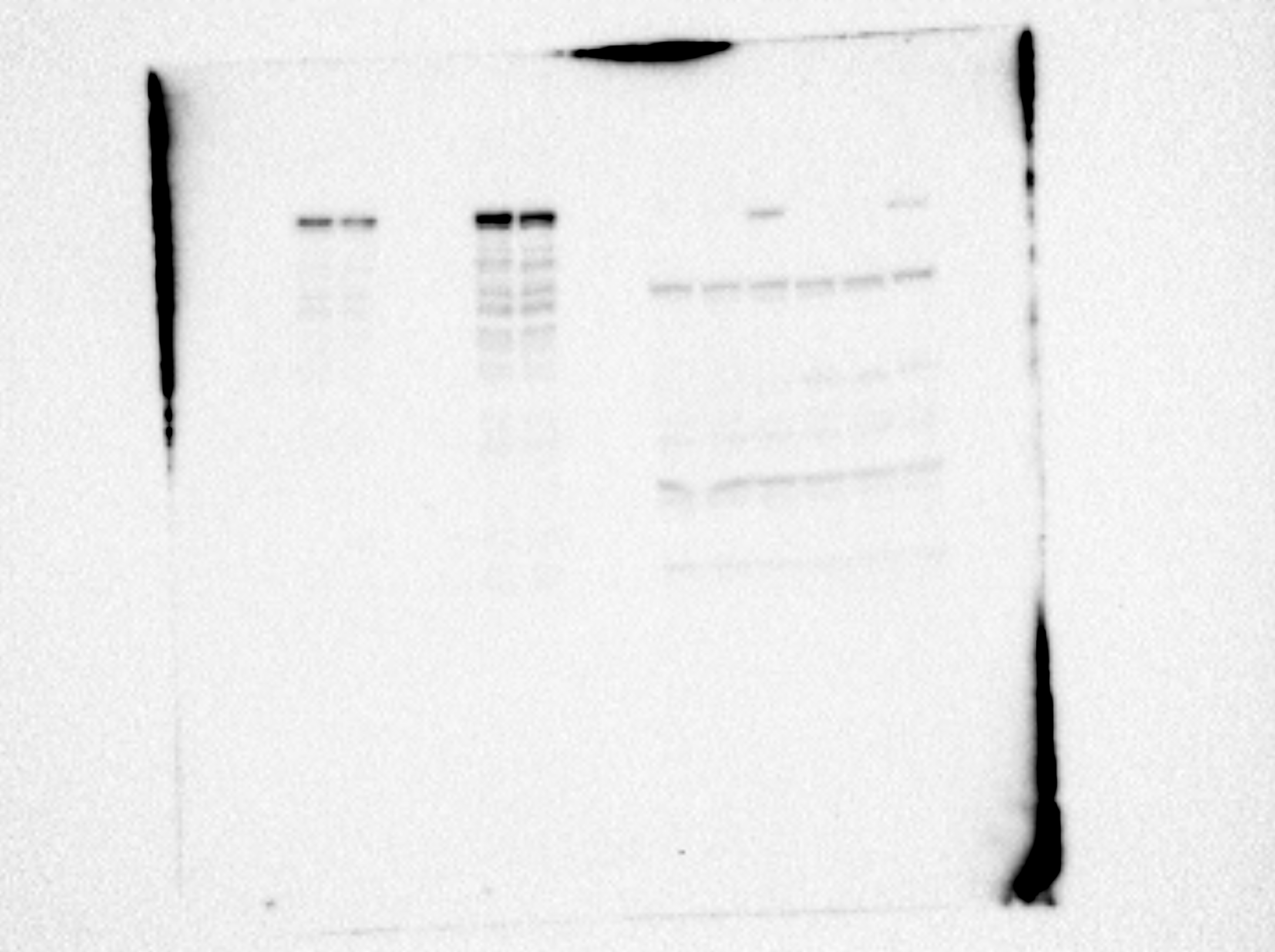

Supplement: Figure 1—source data 3. [file elife-89002-fig1-data3.zip › anti-Myc_Exposure_32.0sec.jpg]

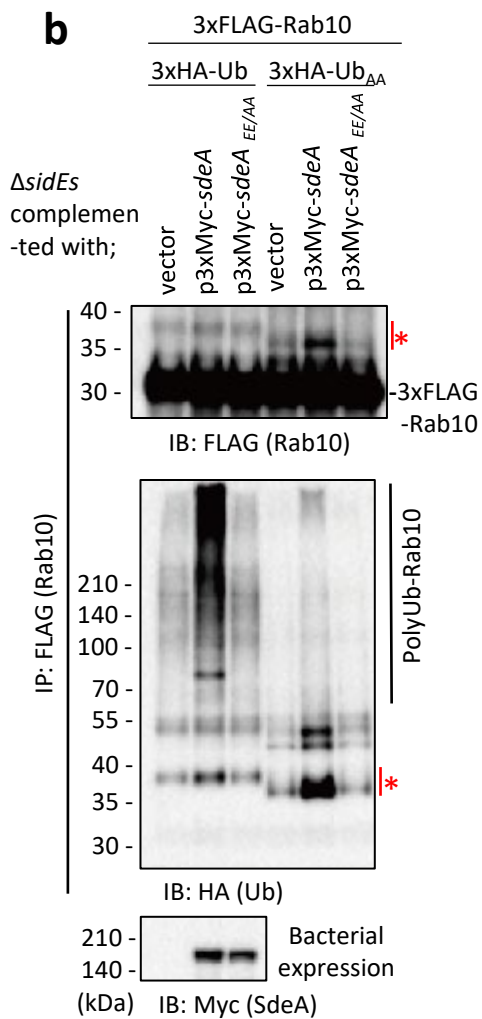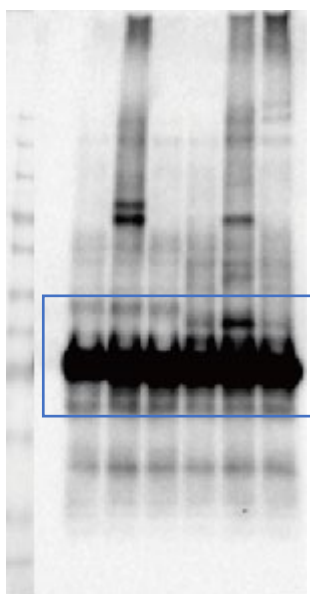

**Figure 1b**  
**top**

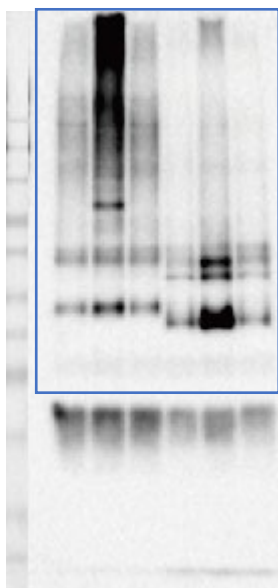

**Figure 1b**  
**middle**

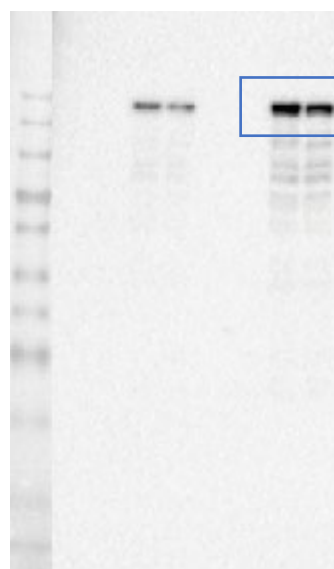

**Figure 1b**  
**bottom**

Supplement: Figure 1—source data 4. [file elife-89002-fig1-data4.pdf]

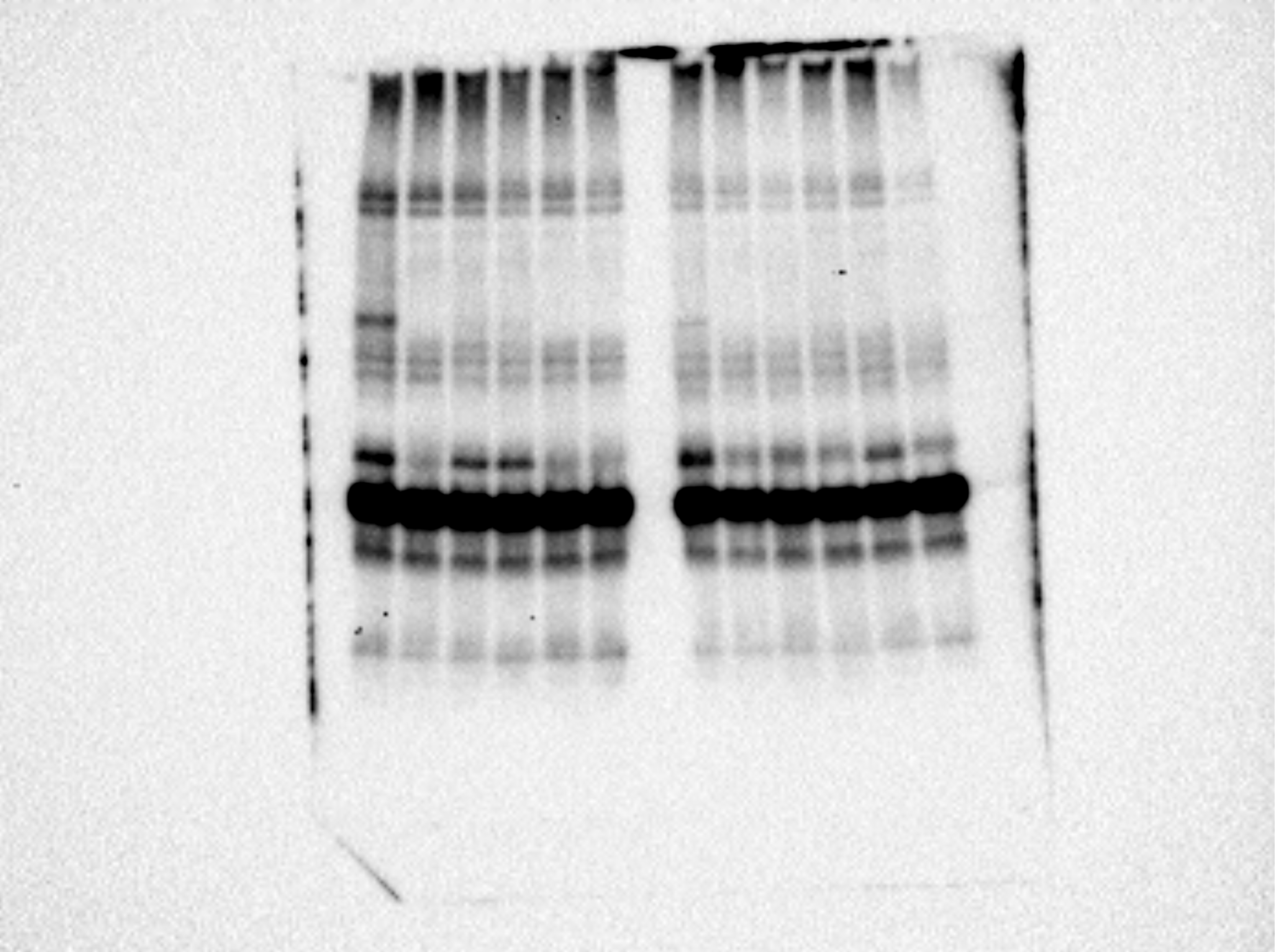

Supplement: Figure 1—source data 5. [file elife-89002-fig1-data5.zip › IP FLAG anti-FLAGm_Exposure_25.8sec.jpg]

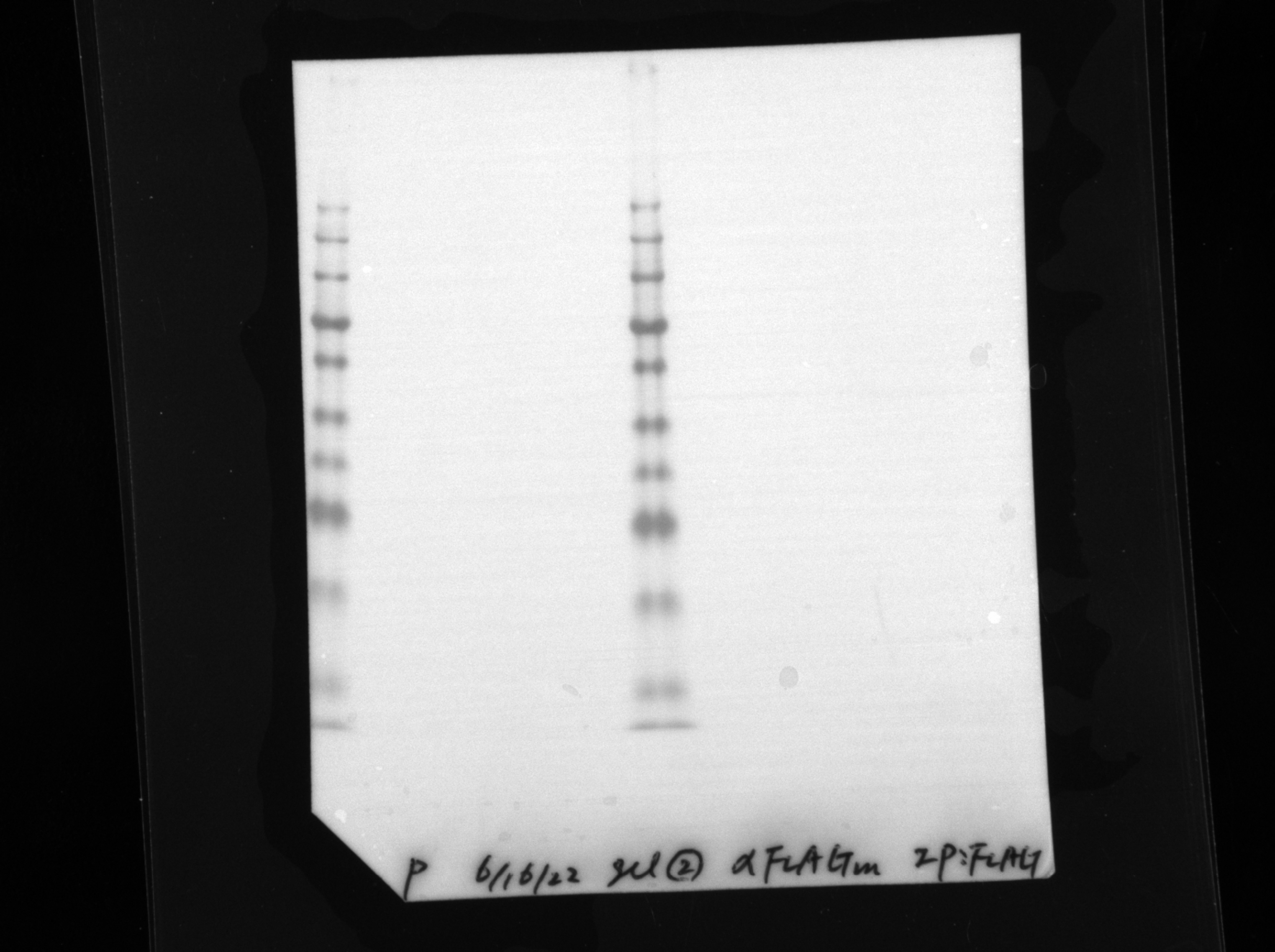

Supplement: Figure 1—source data 5. [file elife-89002-fig1-data5.zip › IP FLAG anti-FLAGm_Marker.jpg]

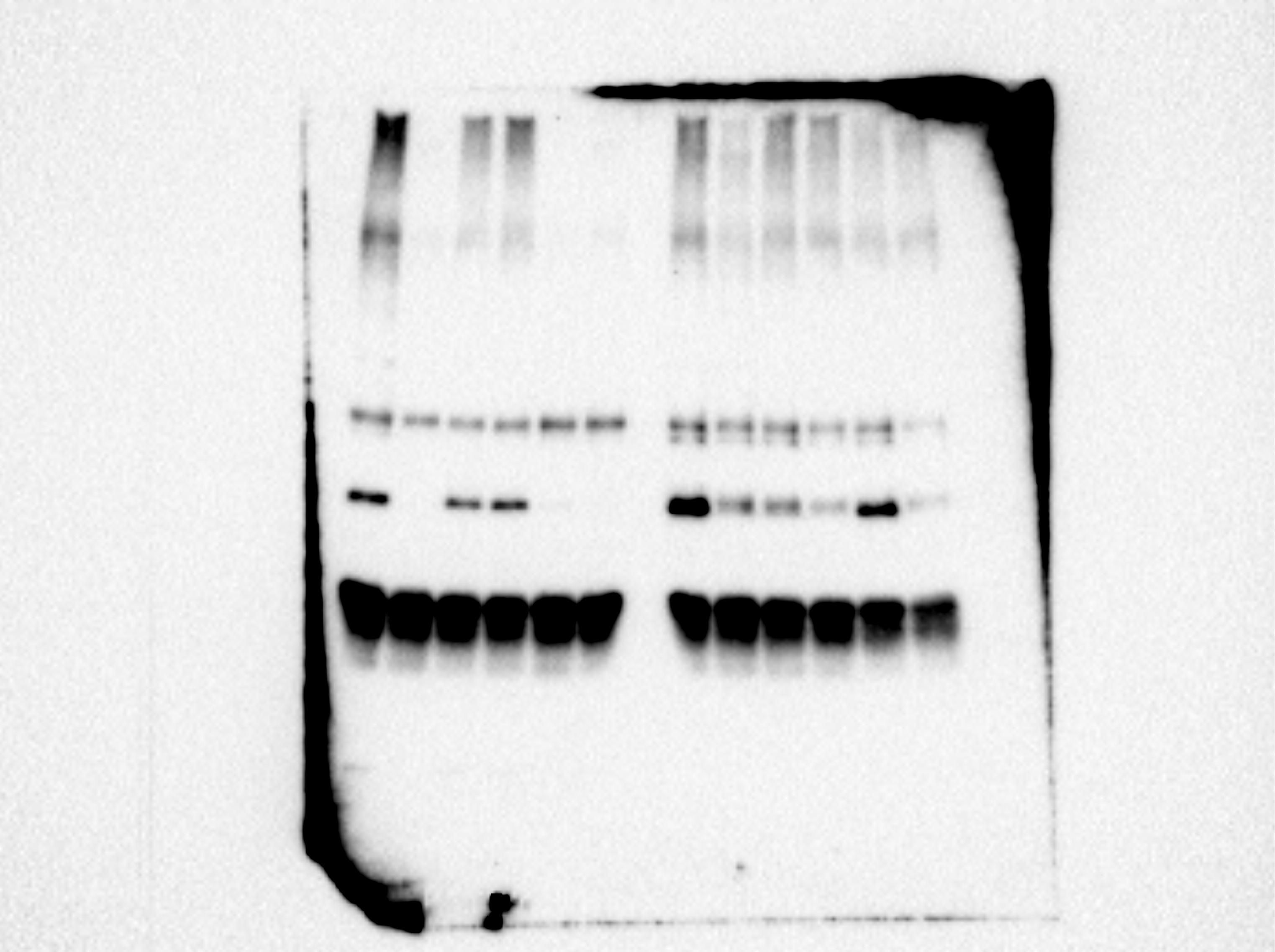

Supplement: Figure 1—source data 5. [file elife-89002-fig1-data5.zip › IP FLAG anti-HAm super_Exposure_14.7sec.jpg]

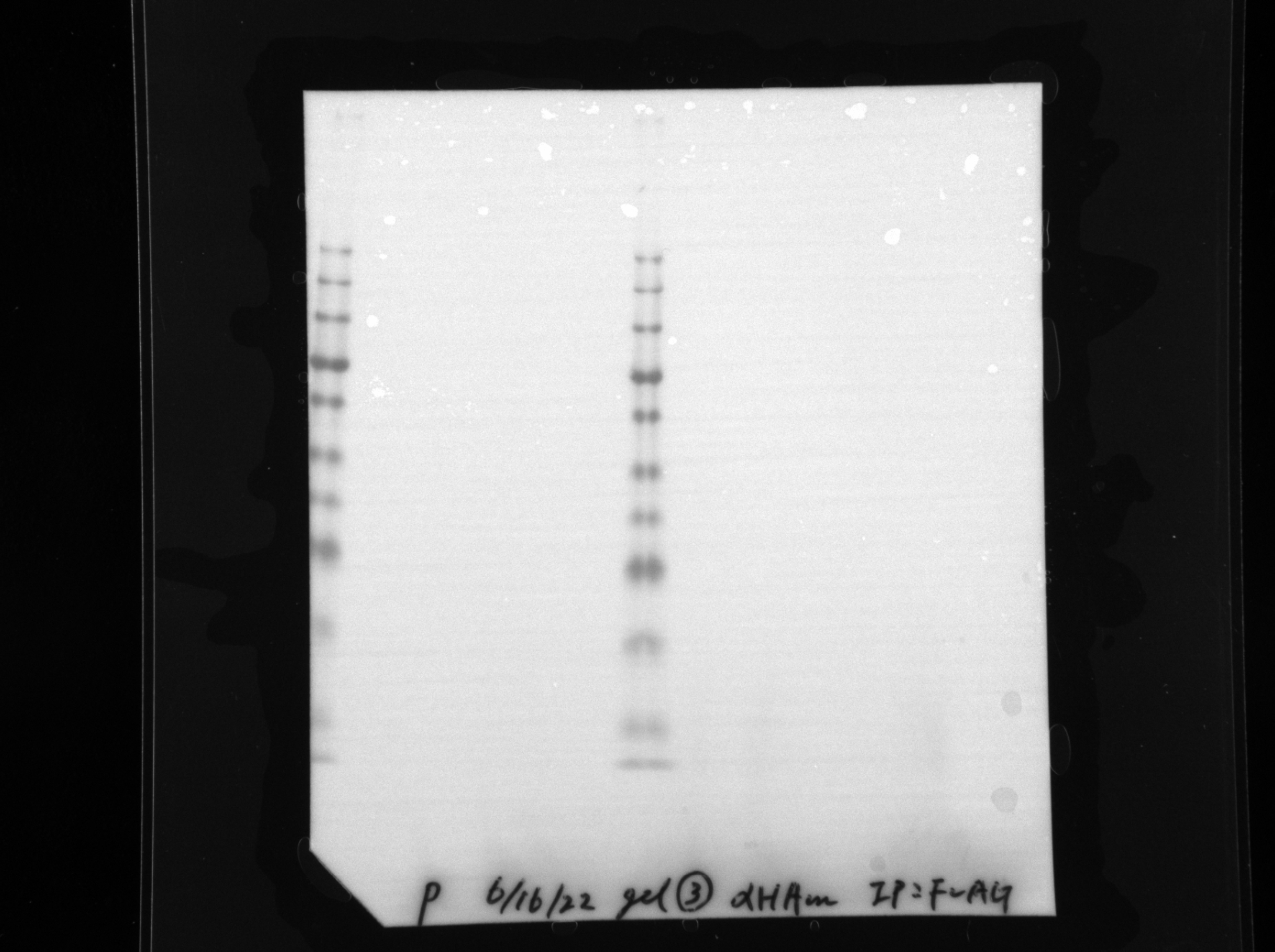

Supplement: Figure 1—source data 5. [file elife-89002-fig1-data5.zip › IP FLAG anti-HAm super_Marker.jpg]

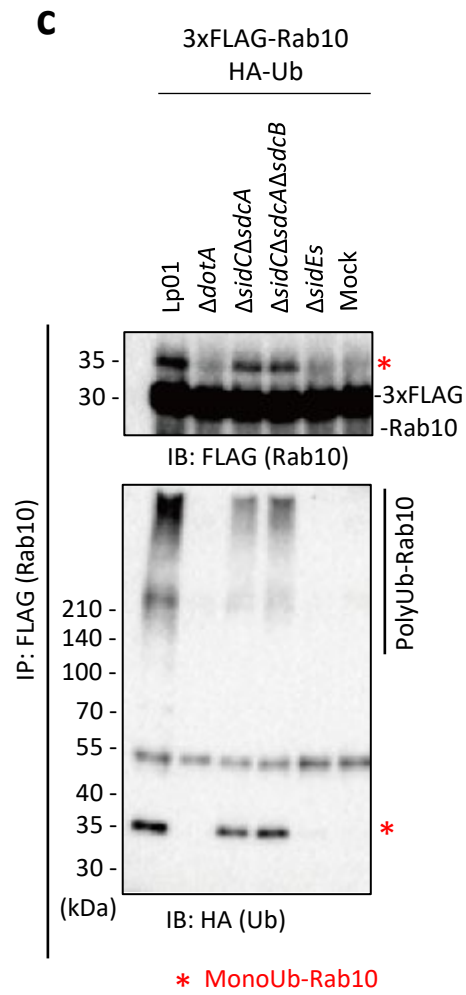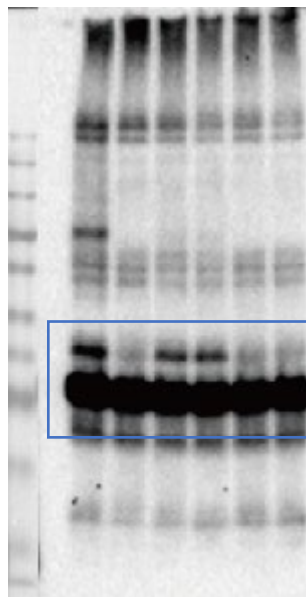

Figure 1c  
top

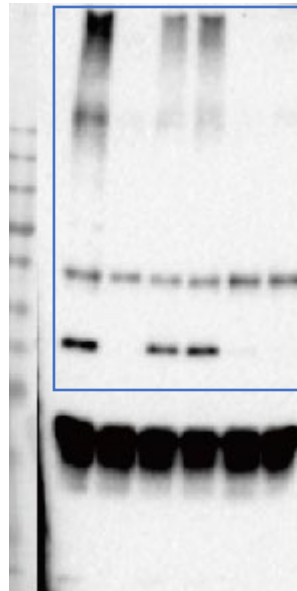

Figure 1c  
bottom

Supplement: Figure 1—source data 6. [file elife-89002-fig1-data6.pdf]

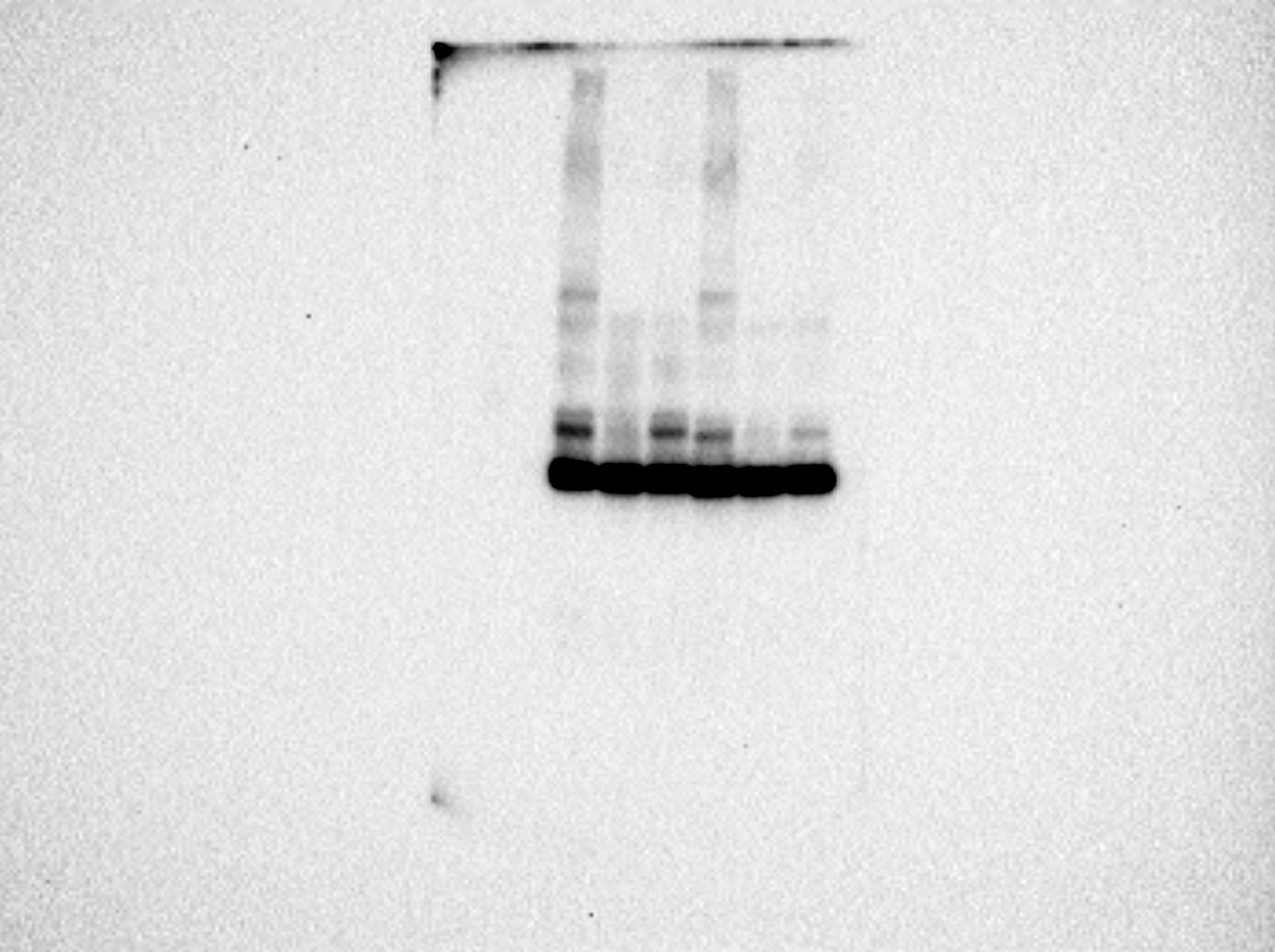

Supplement: Figure 1—figure supplement 1—source data 1. [file elife-89002-fig1-figsupp1-data1.zip › IP FLAG anti-FLAGm_Exposure_60.0sec.jpg]

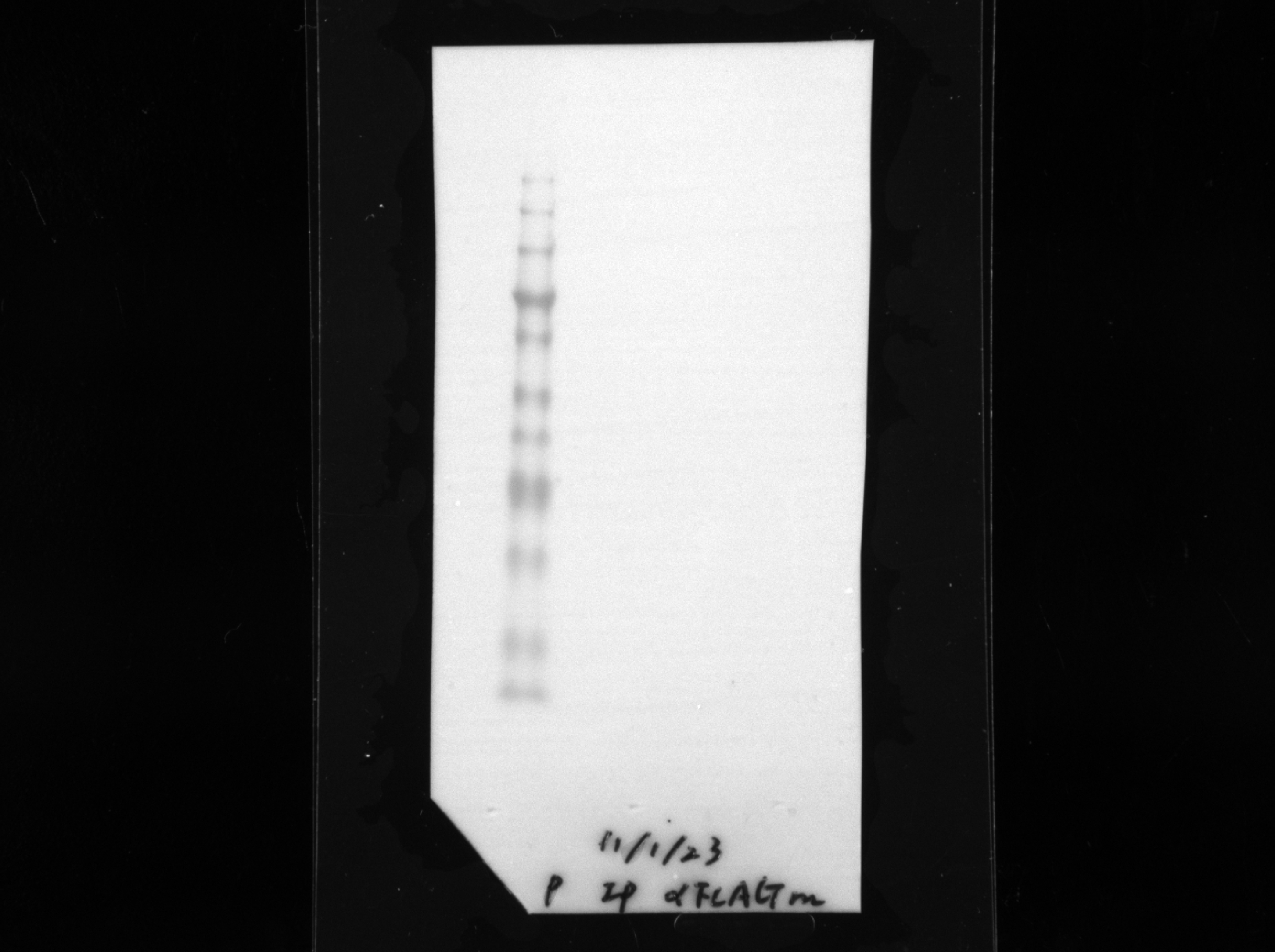

Supplement: Figure 1—figure supplement 1—source data 1. [file elife-89002-fig1-figsupp1-data1.zip › IP FLAG anti-FLAGm_Marker.jpg]

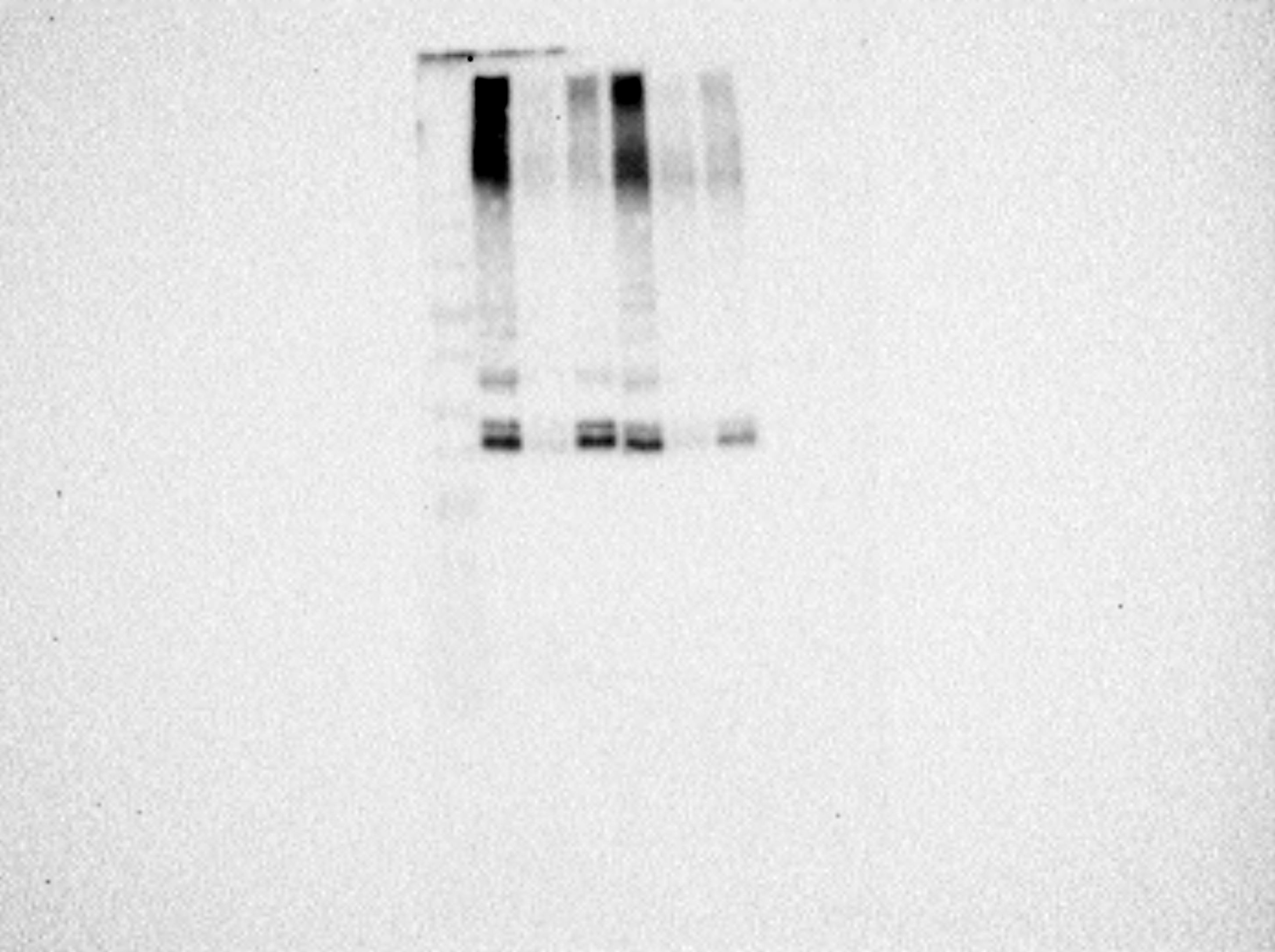

Supplement: Figure 1—figure supplement 1—source data 1. [file elife-89002-fig1-figsupp1-data1.zip › IP FLAG anti-HArb_Exposure_120.0sec.jpg]

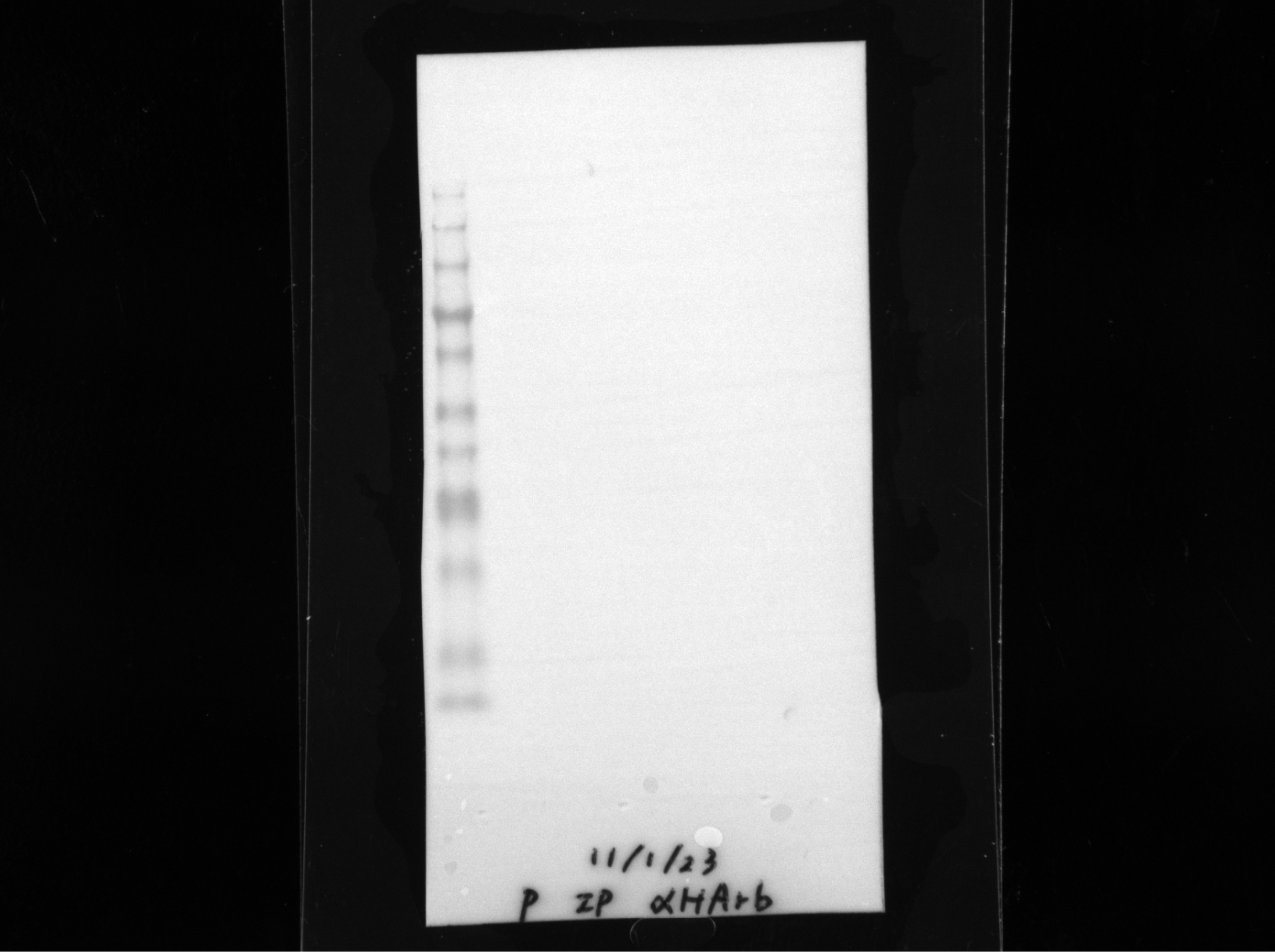

Supplement: Figure 1—figure supplement 1—source data 1. [file elife-89002-fig1-figsupp1-data1.zip › IP FLAG anti-HArb_Marker.jpg]

**a**

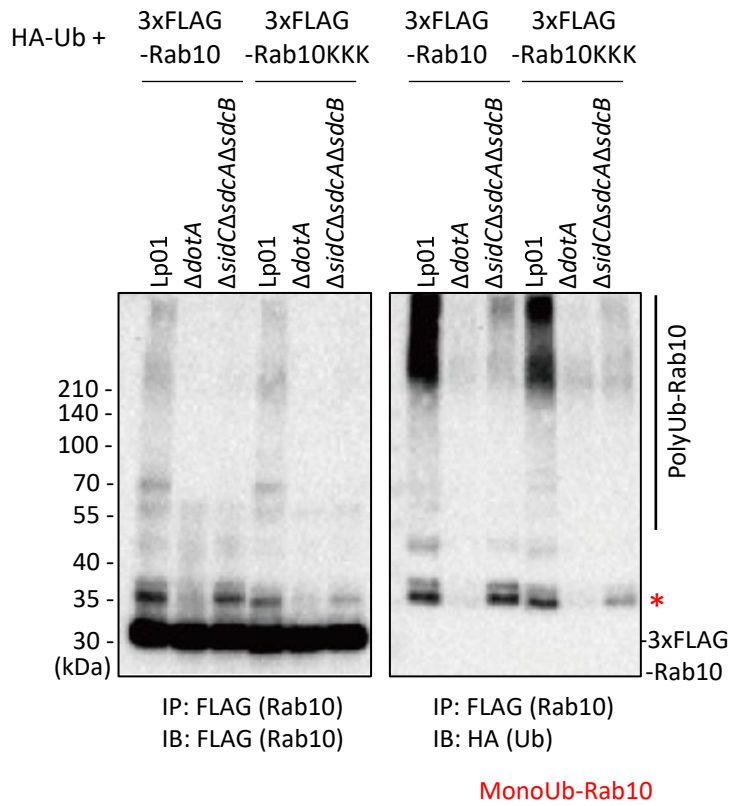

**Figure 1**  
– figure supplement 1a  
left

**Figure 1**  
– figure supplement 1a  
right

Supplement: Figure 1—figure supplement 1—source data 2. [file elife-89002-fig1-figsupp1-data2.pdf]

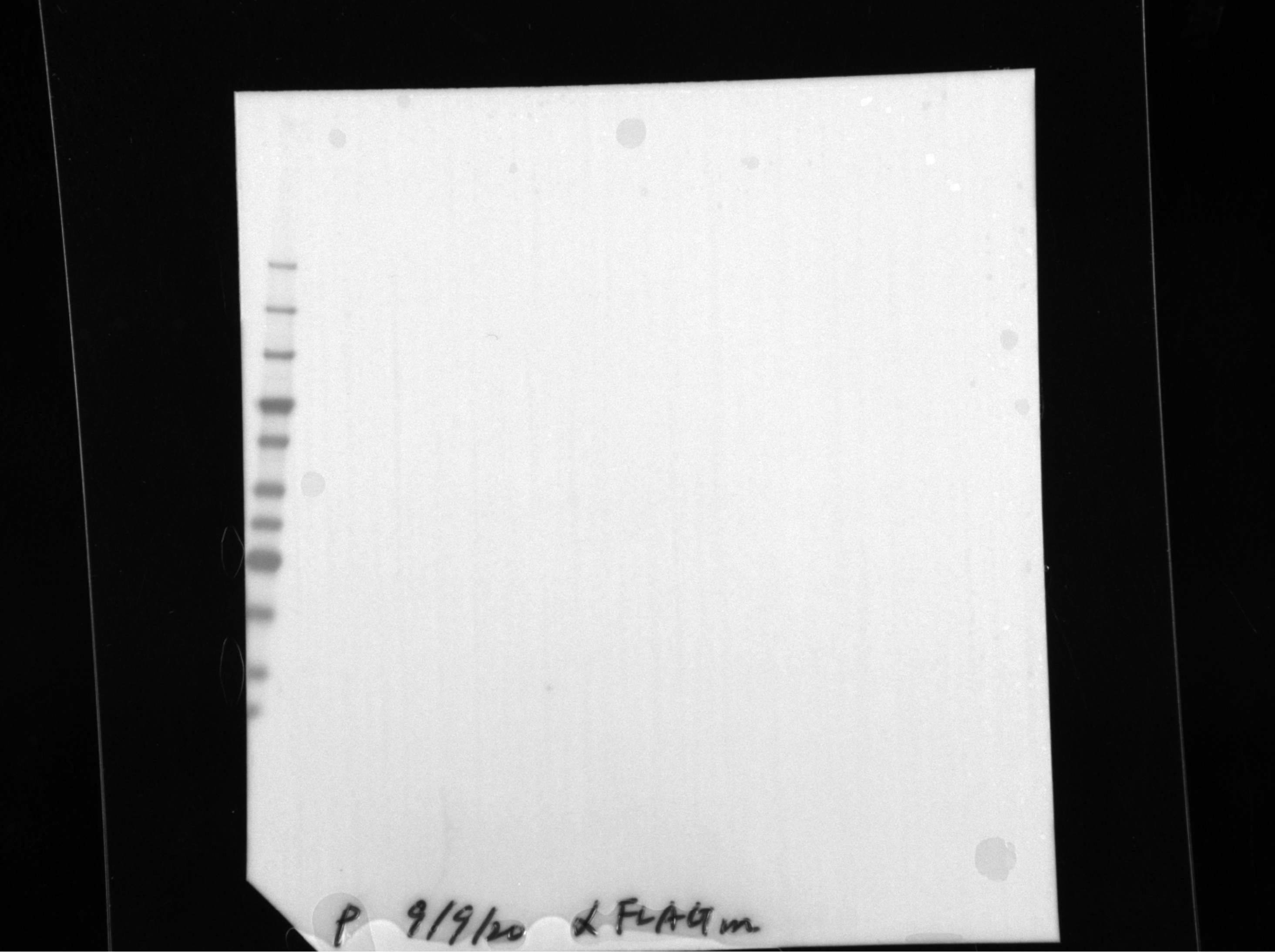

Supplement: Figure 1—figure supplement 1—source data 3. [file elife-89002-fig1-figsupp1-data3.zip › anti-FLAG Marker.jpg]

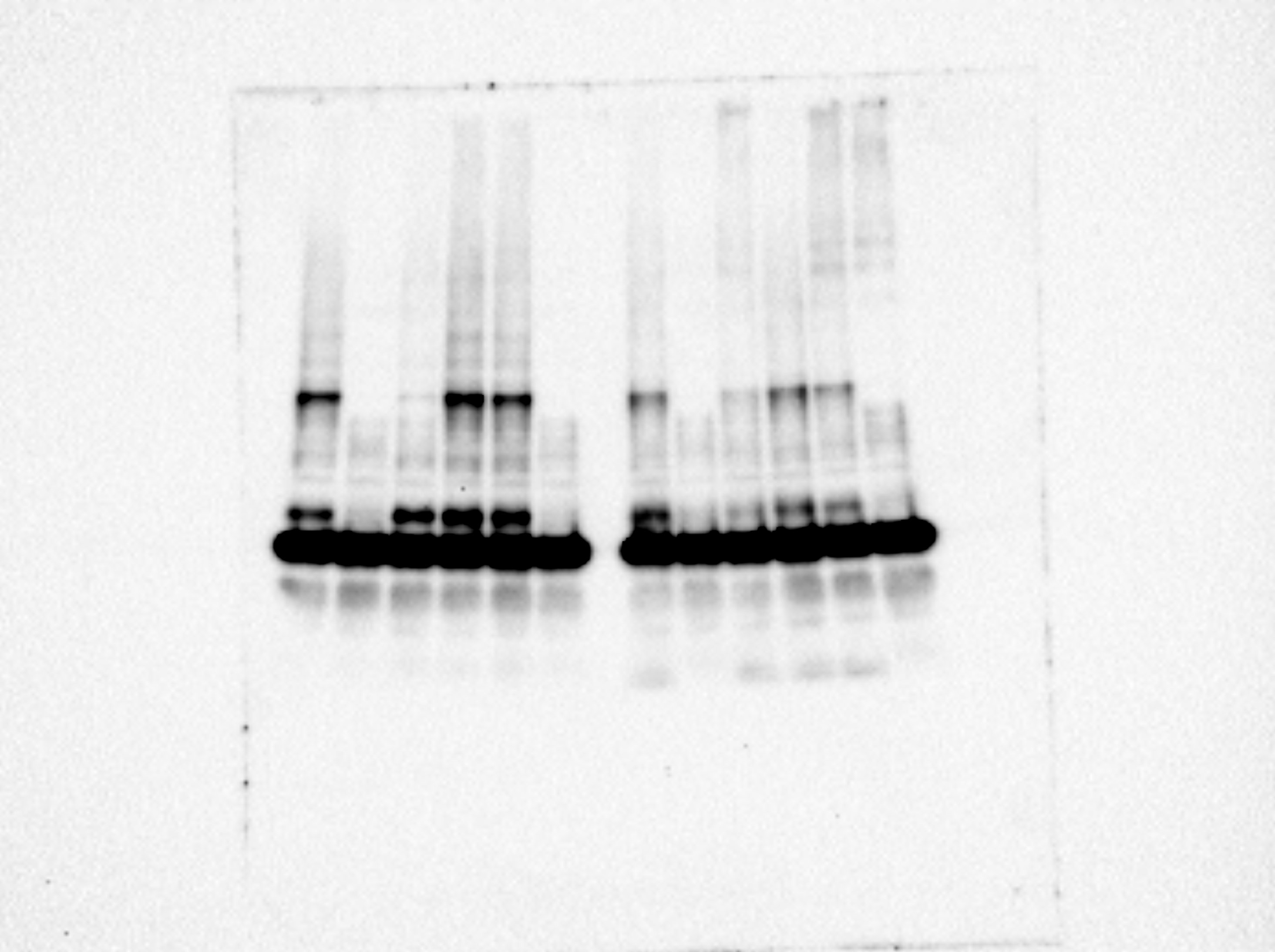

Supplement: Figure 1—figure supplement 1—source data 3. [file elife-89002-fig1-figsupp1-data3.zip › anti-FLAG_Exposure_100.7sec.jpg]

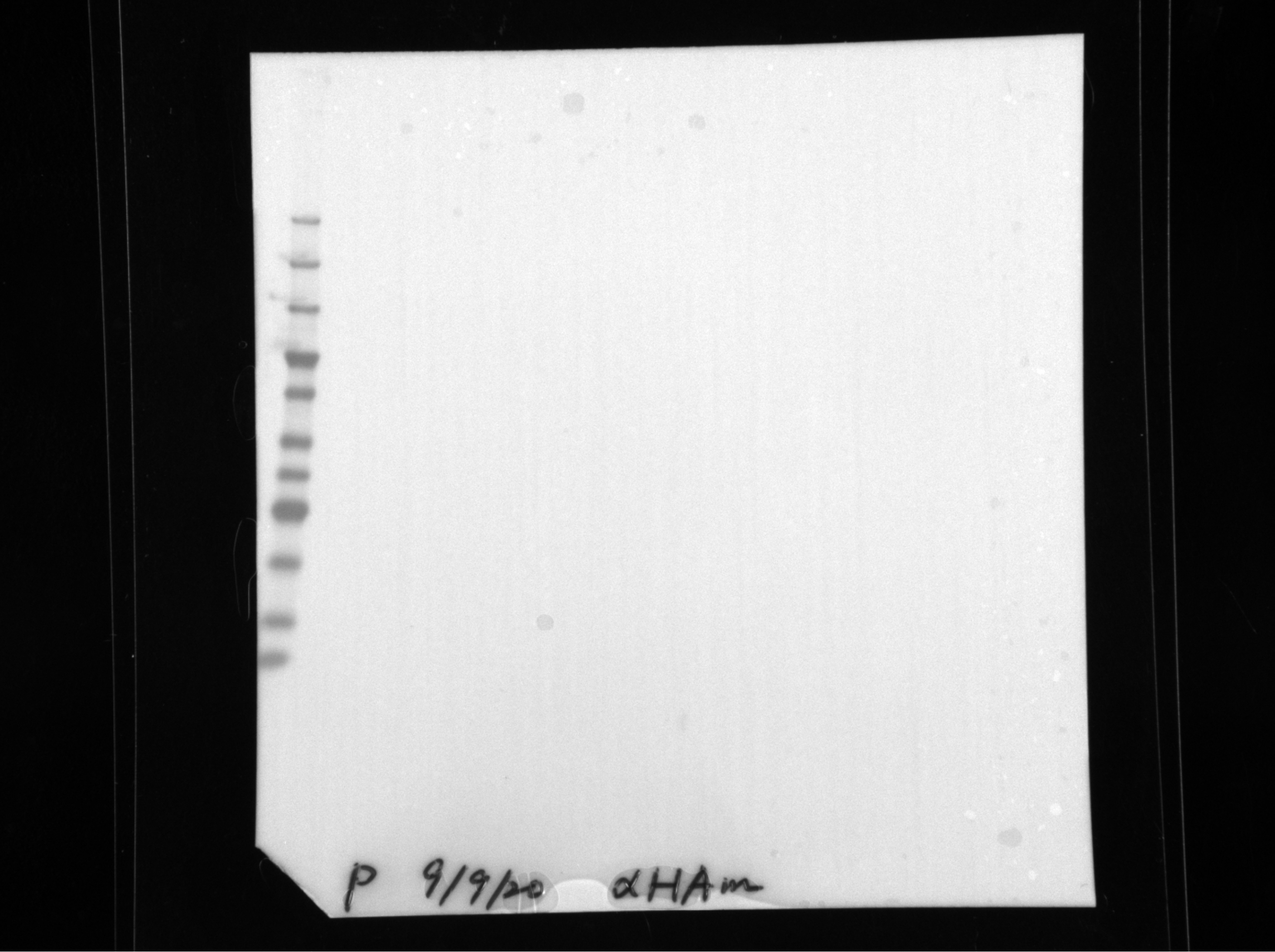

Supplement: Figure 1—figure supplement 1—source data 3. [file elife-89002-fig1-figsupp1-data3.zip › anti-HArb Marker.jpg]

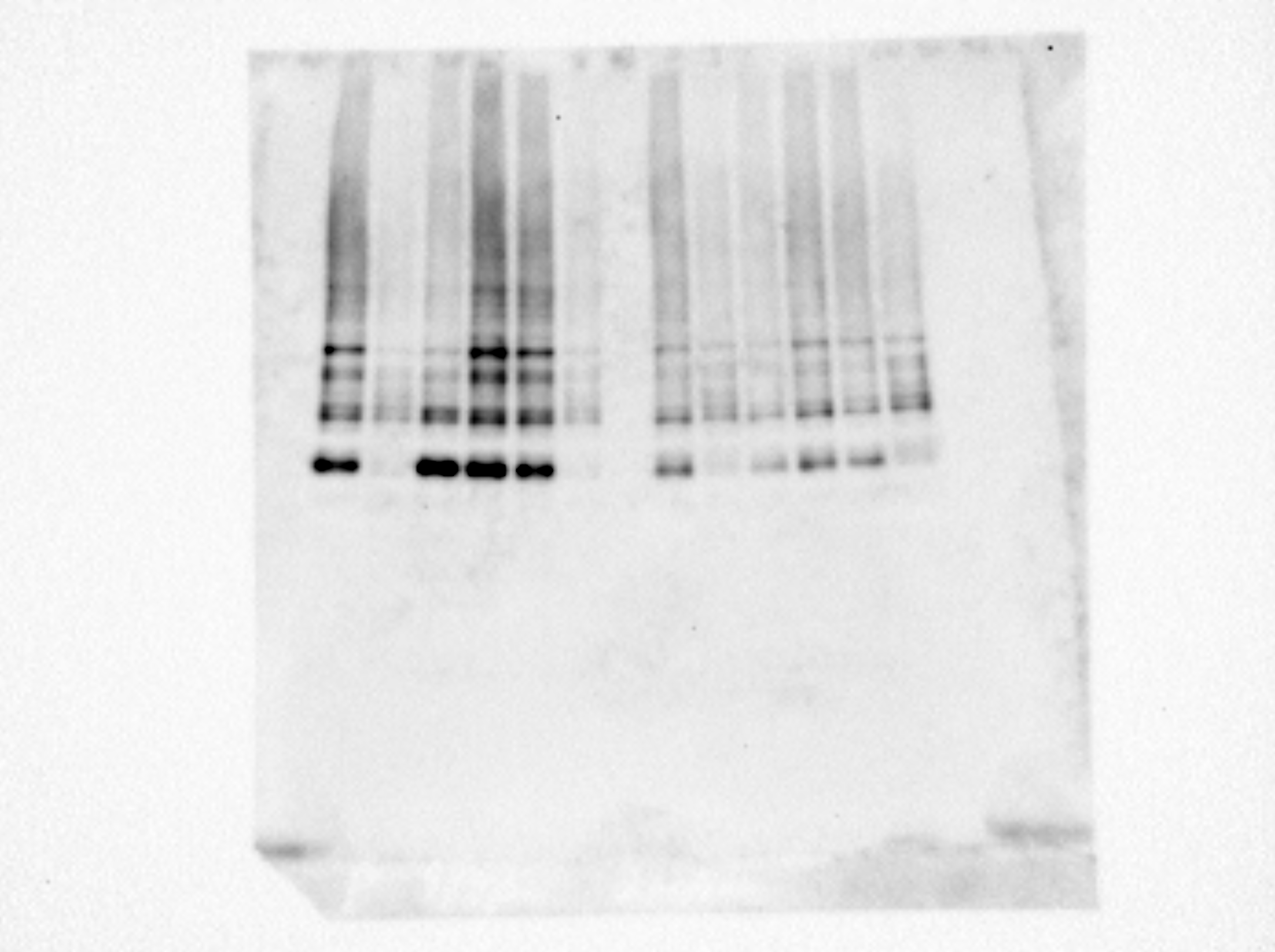

Supplement: Figure 1—figure supplement 1—source data 3. [file elife-89002-fig1-figsupp1-data3.zip › anti-HArb_Exposure_60.8sec.jpg]

**b**

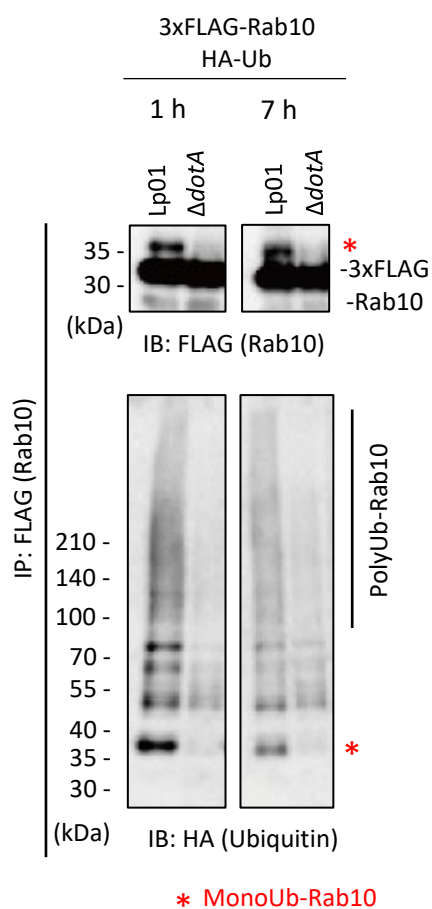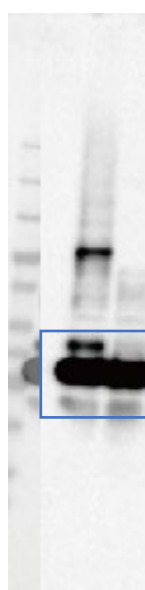

**Figure 1**  
– figure supplement 1b  
top

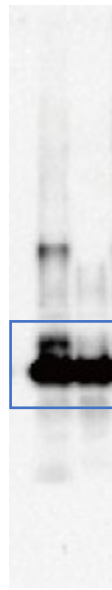

**Figure 1**  
– figure supplement 1b  
bottom

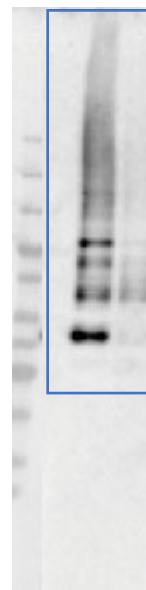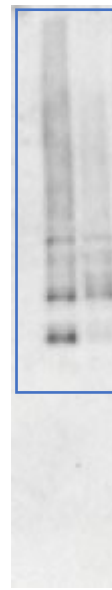

Supplement: Figure 1—figure supplement 1—source data 4. [file elife-89002-fig1-figsupp1-data4.pdf]

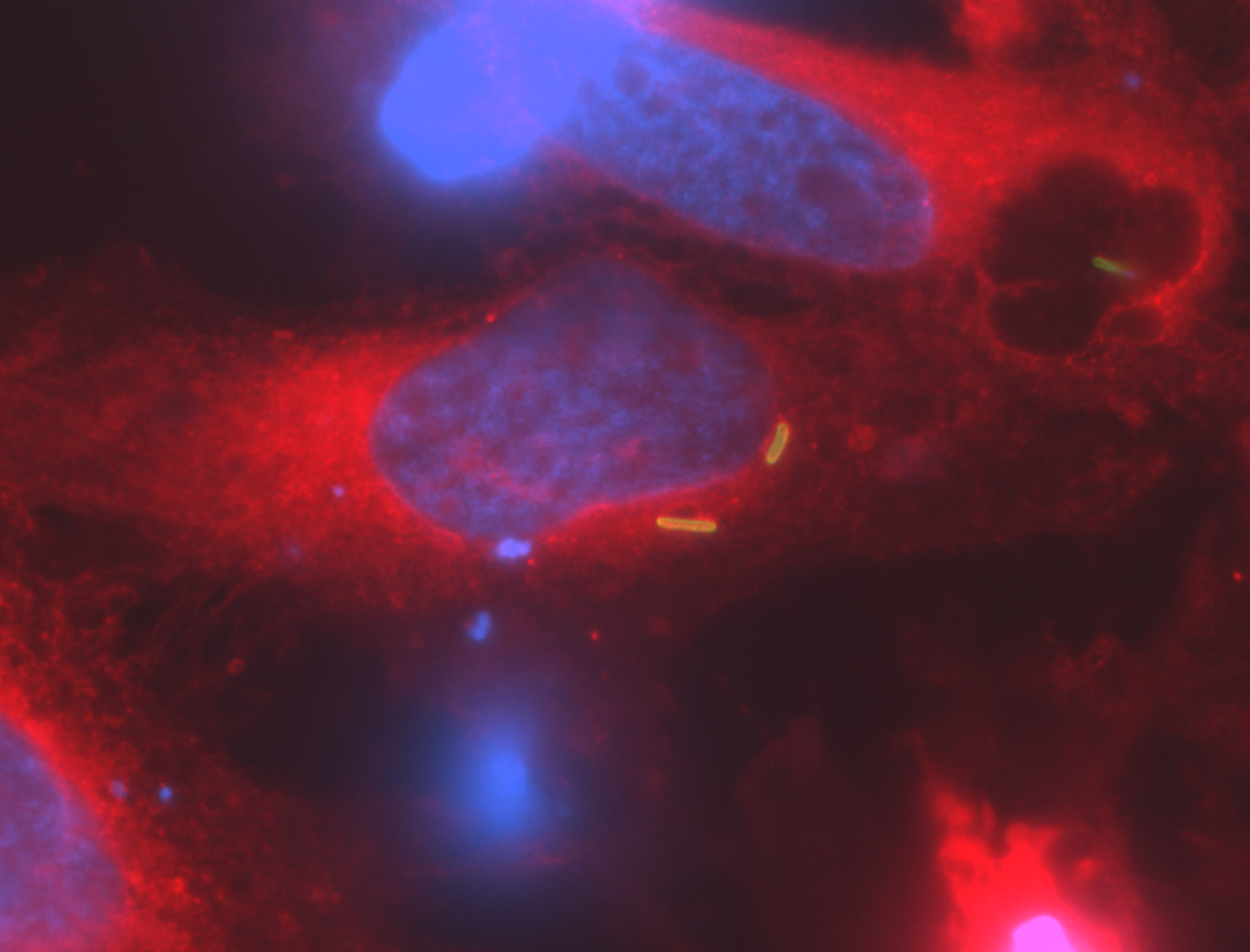

Supplement: Figure 2—source data 1. [file elife-89002-fig2-data1.zip › 4hr CR39 +1.tif]

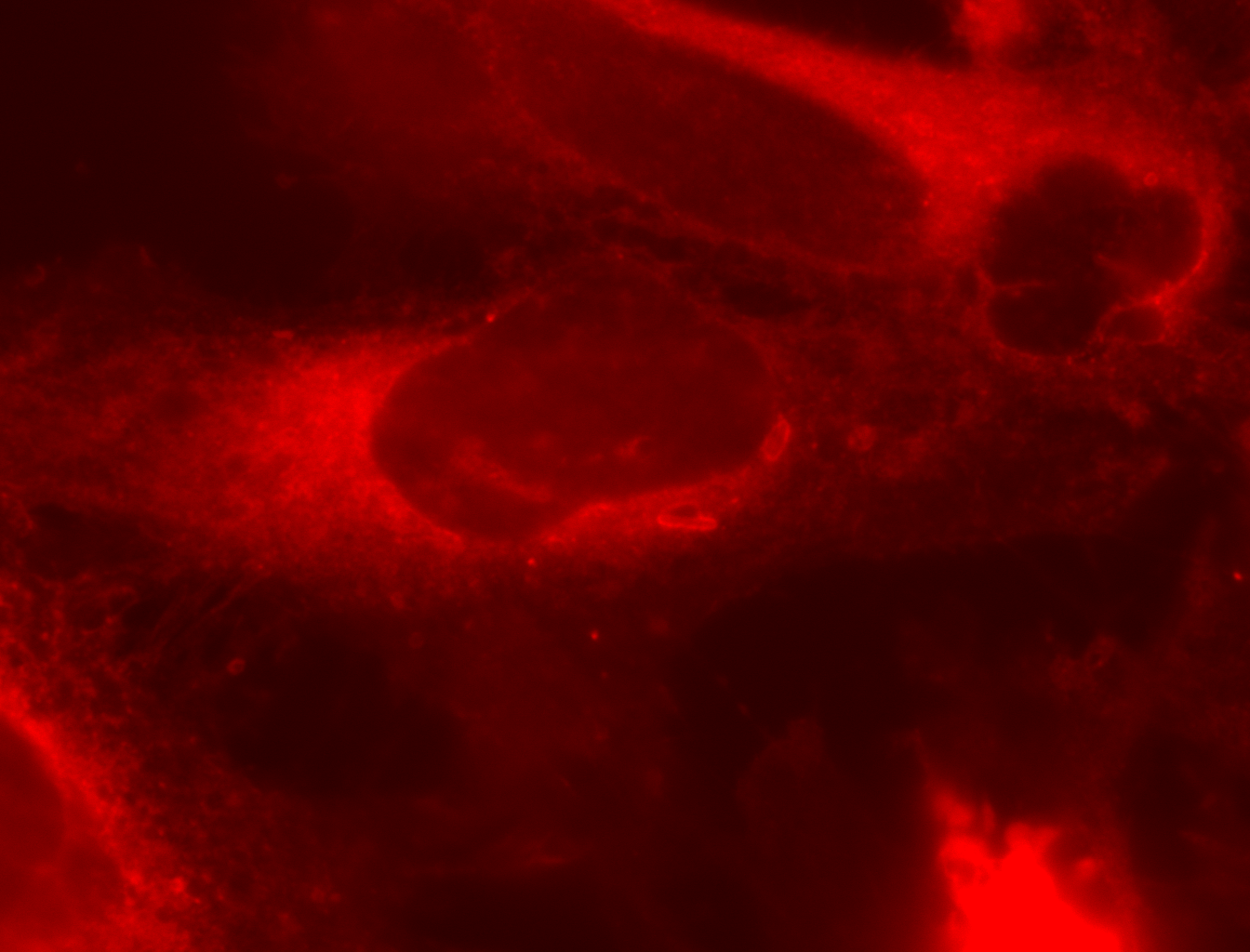

Supplement: Figure 2—source data 1. [file elife-89002-fig2-data1.zip › 4hr cr39 +1c1.tif]

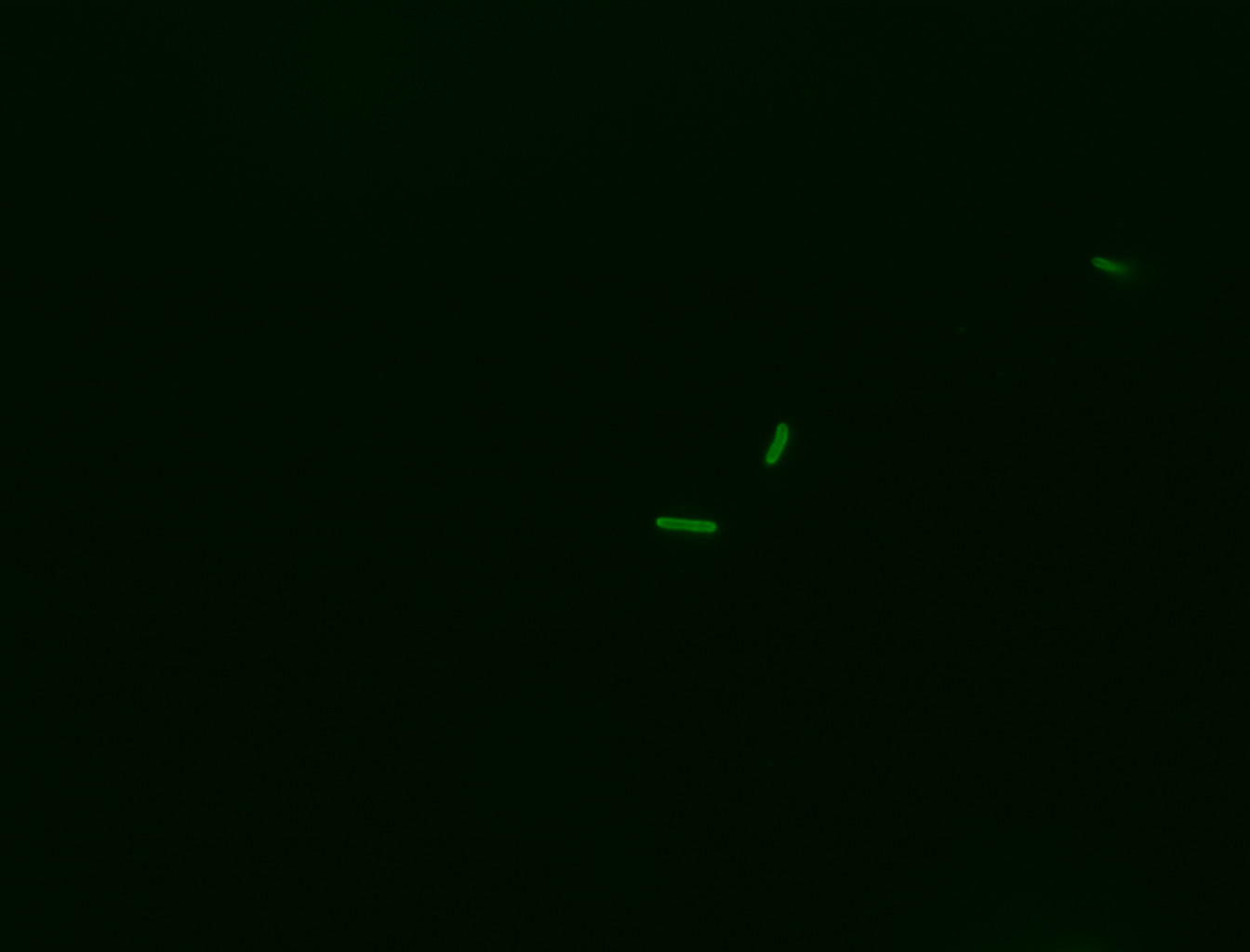

Supplement: Figure 2—source data 1. [file elife-89002-fig2-data1.zip › 4hr cr39 +1c2.tif]

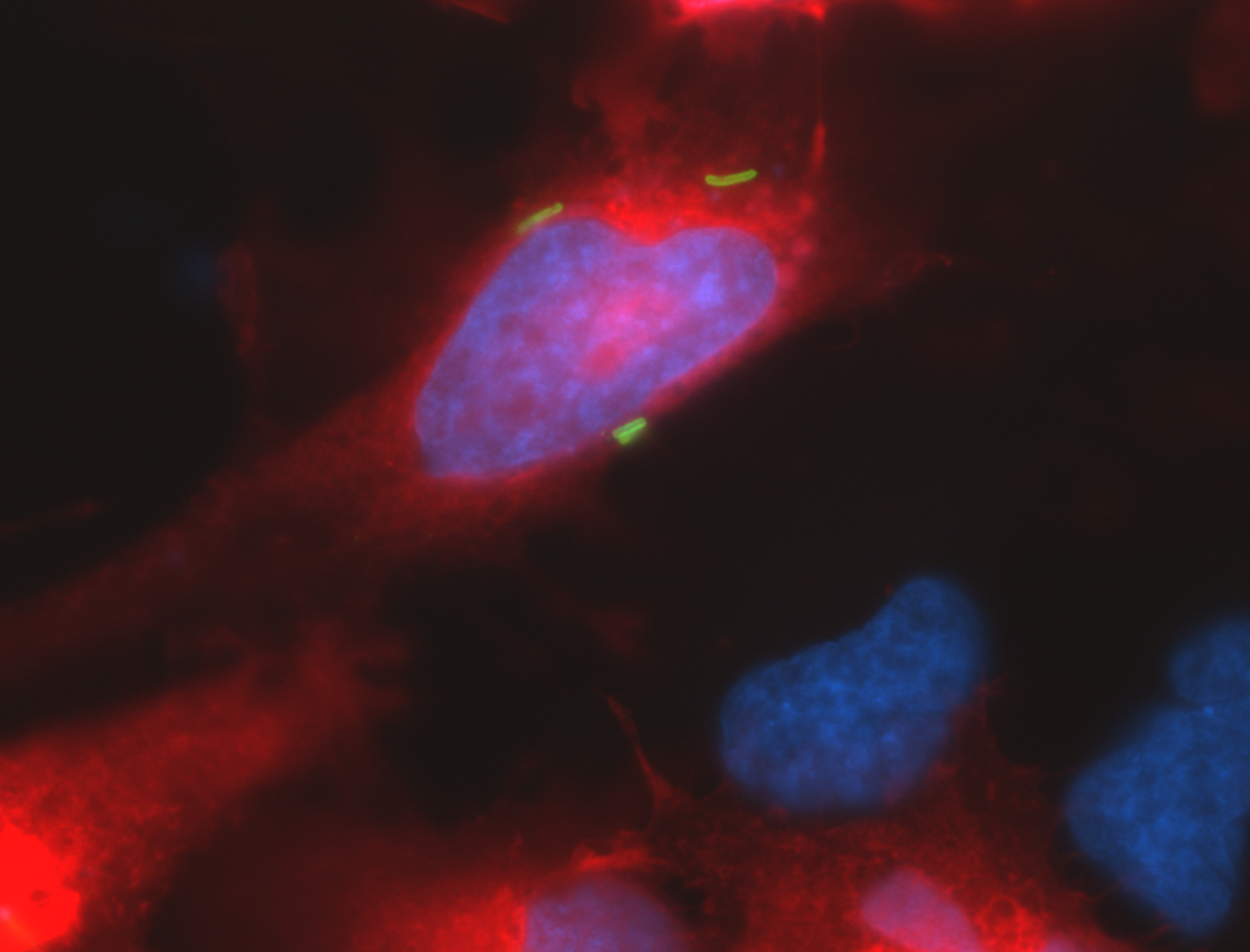

Supplement: Figure 2—source data 1. [file elife-89002-fig2-data1.zip › 4hr CR58 -2.tif]

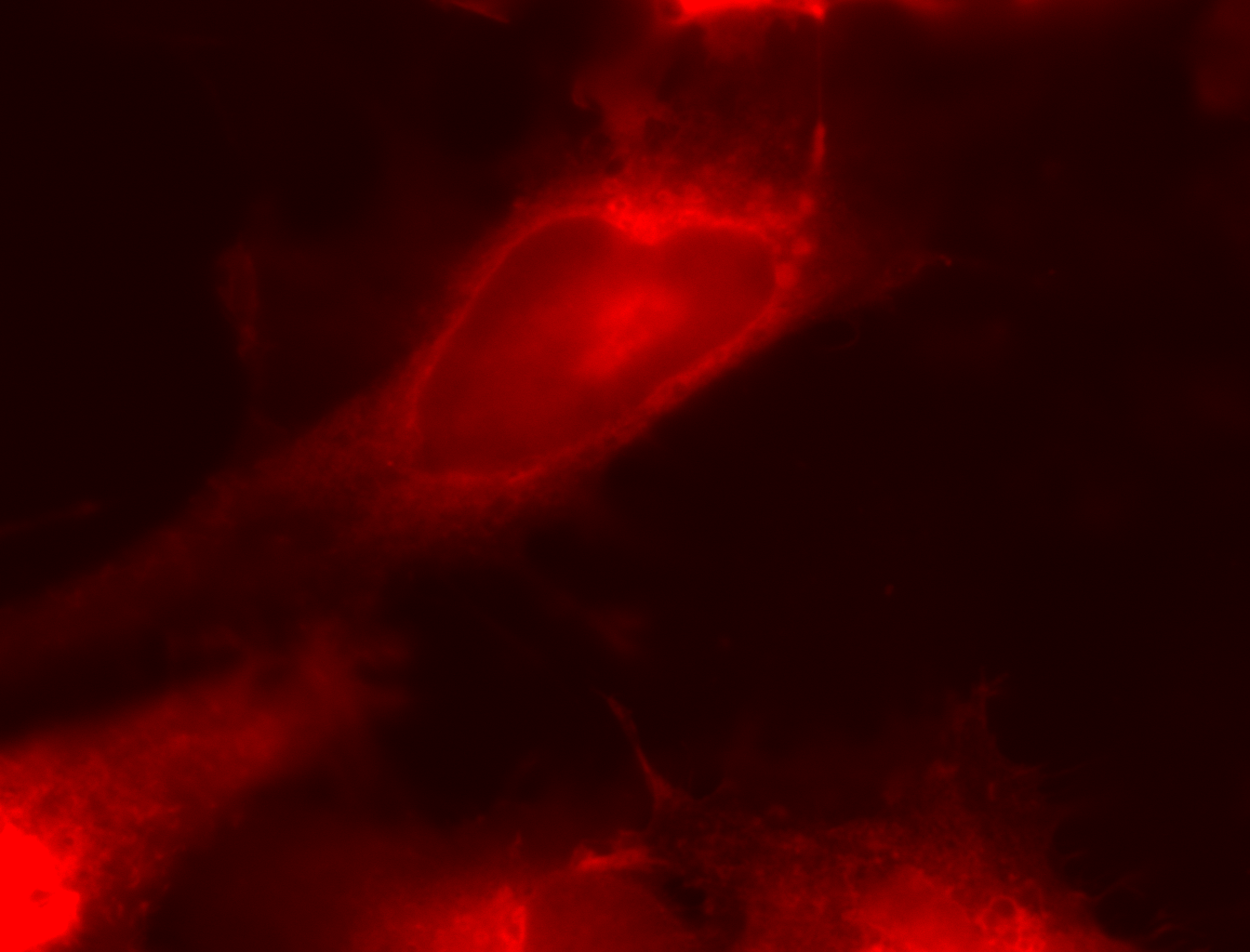

Supplement: Figure 2—source data 1. [file elife-89002-fig2-data1.zip › 4hr cr58 -2c1.tif]

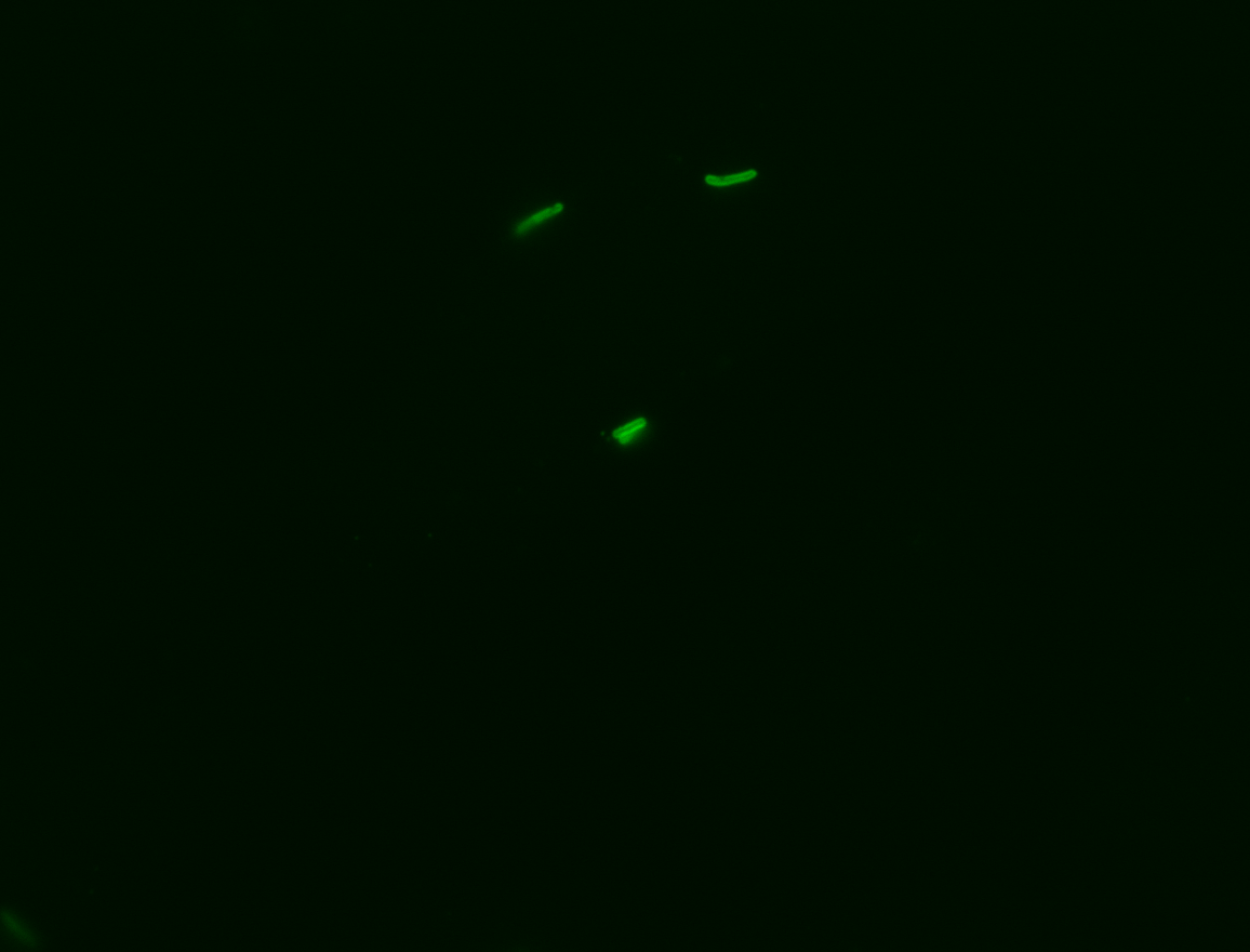

Supplement: Figure 2—source data 1. [file elife-89002-fig2-data1.zip › 4hr cr58 -2c2.tif]

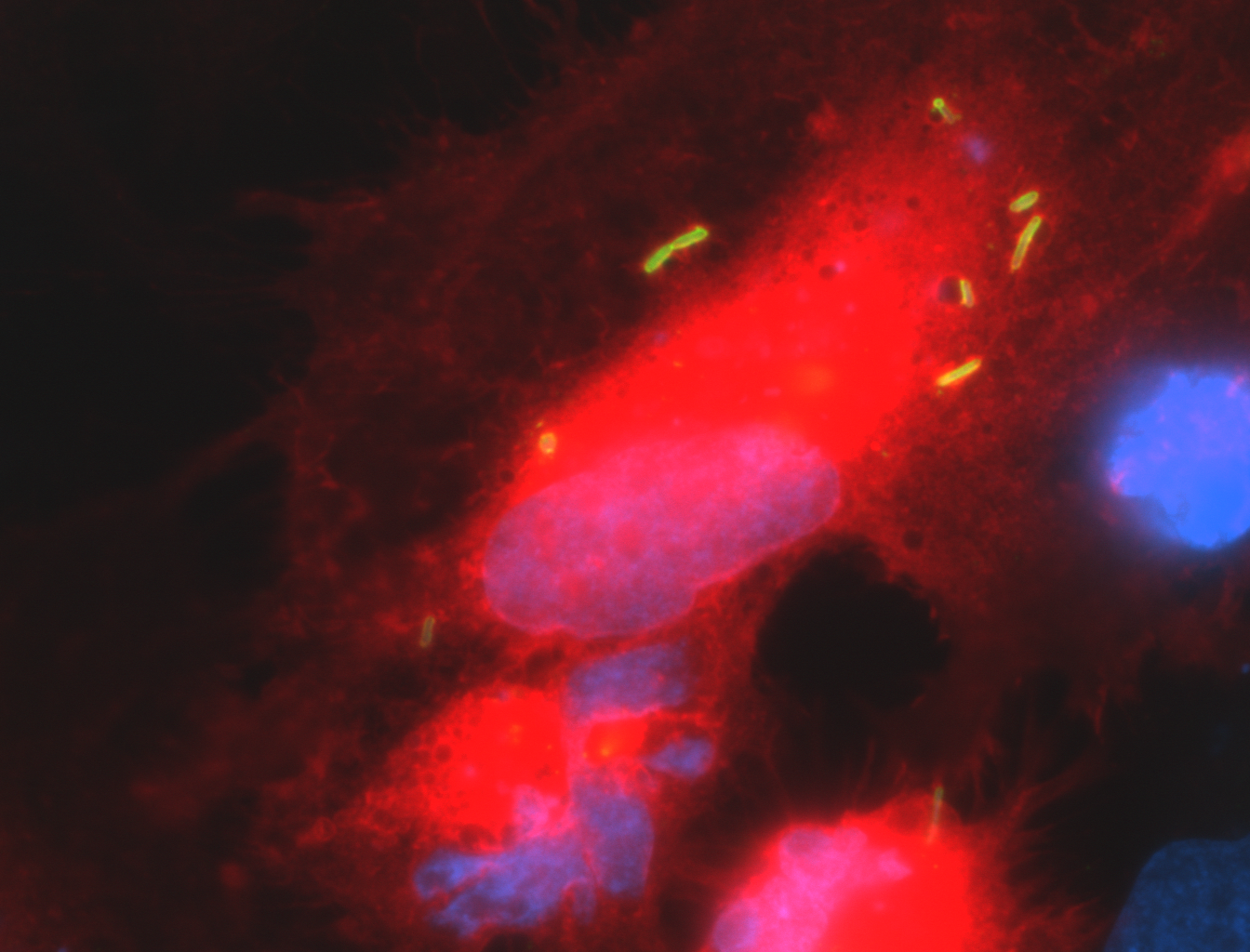

Supplement: Figure 2—source data 1. [file elife-89002-fig2-data1.zip › 4hr sidCsdcA +4.tif]

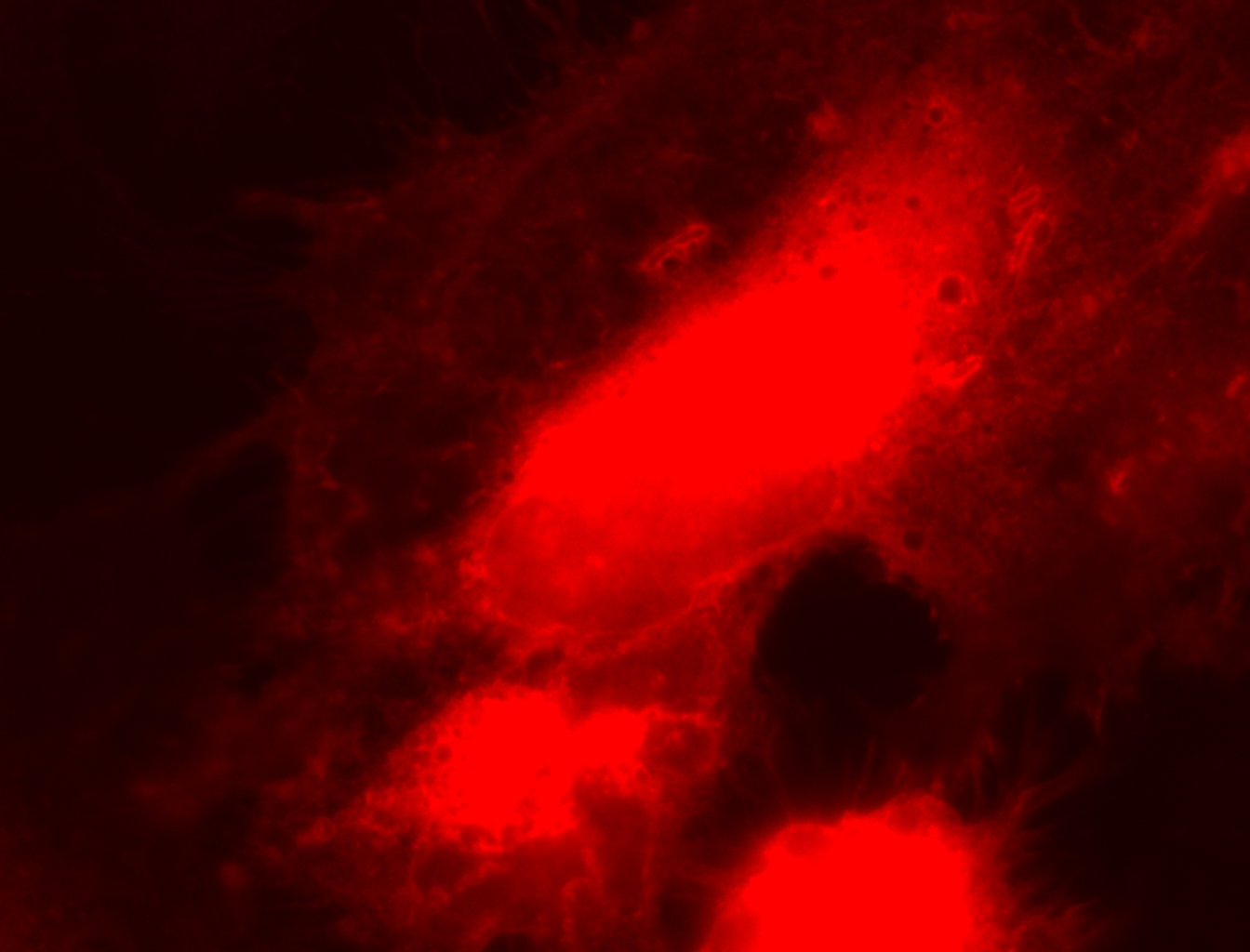

Supplement: Figure 2—source data 1. [file elife-89002-fig2-data1.zip › 4hr sidcsdca +4c1.tif]

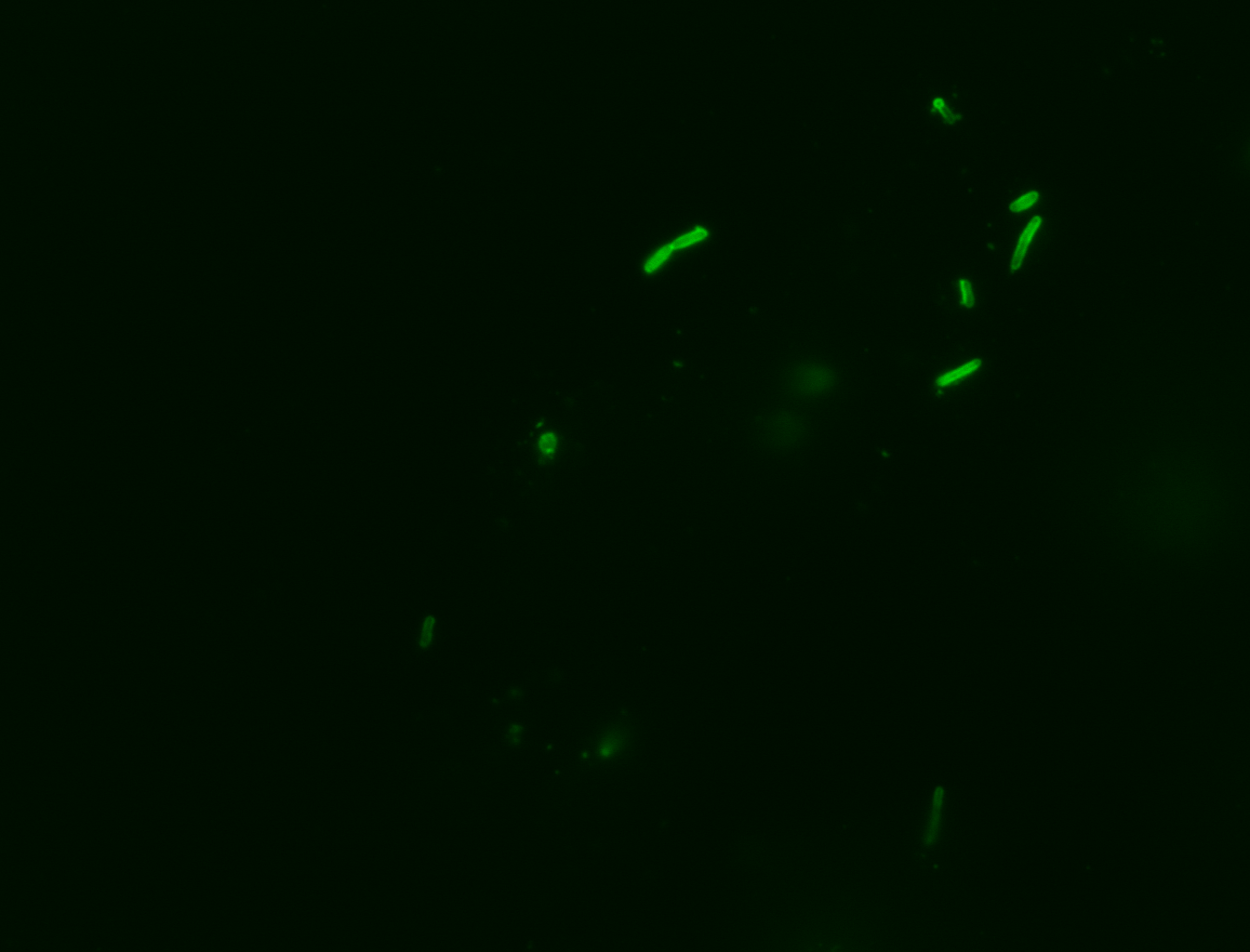

Supplement: Figure 2—source data 1. [file elife-89002-fig2-data1.zip › 4hr sidcsdca +4c2.tif]

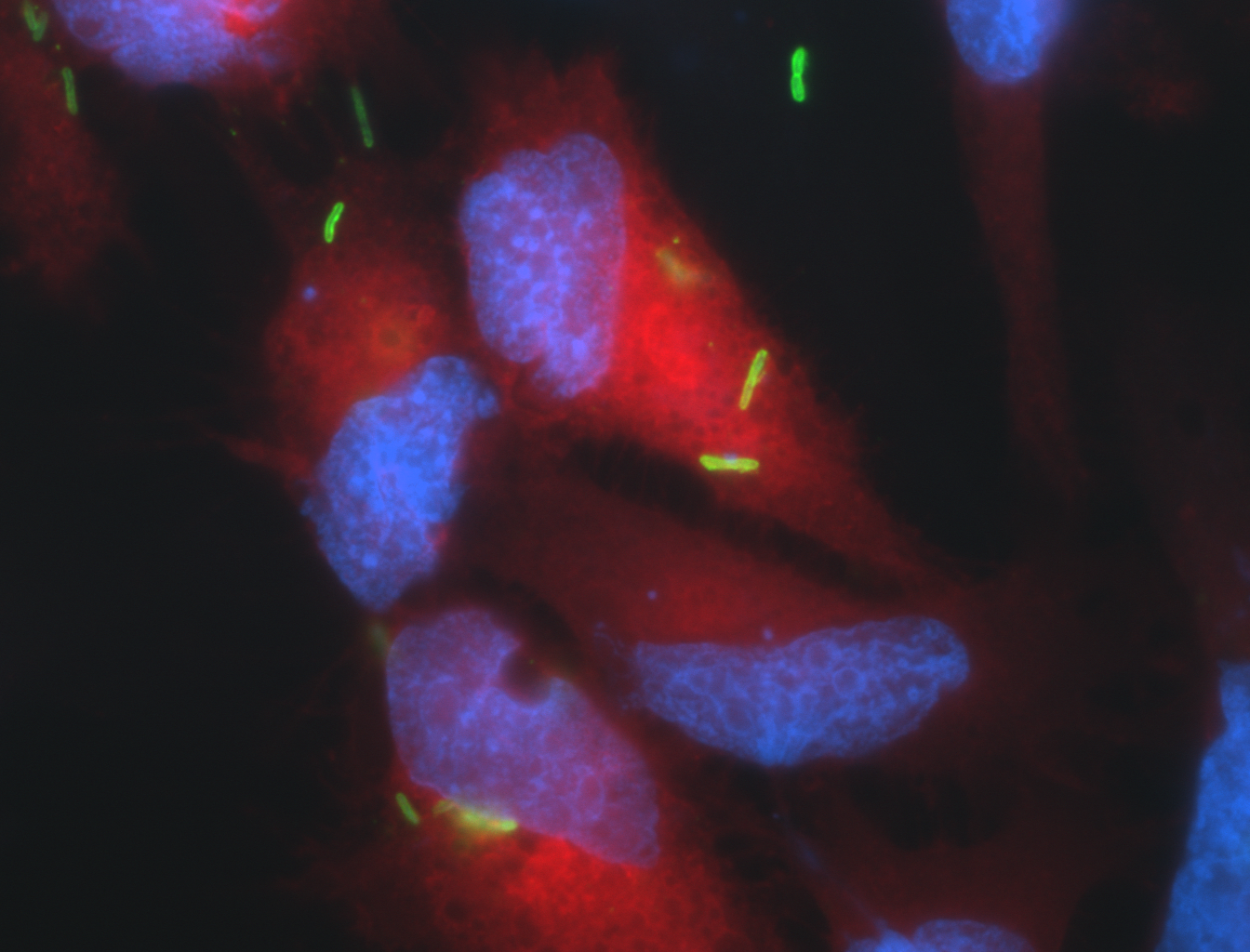

Supplement: Figure 2—source data 1. [file elife-89002-fig2-data1.zip › 4hr sidCsdcAsdcB -7.tif]

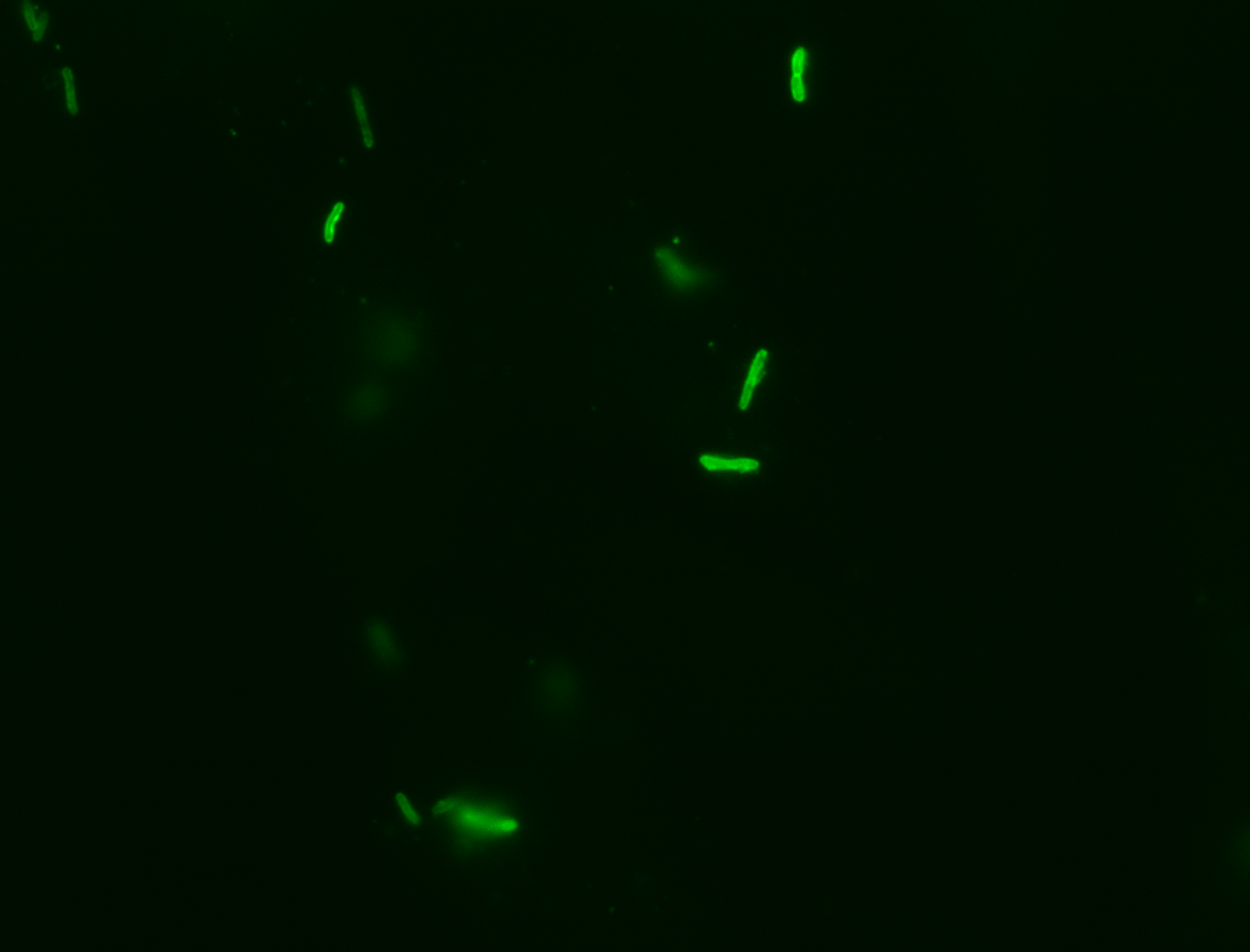

Supplement: Figure 2—source data 1. [file elife-89002-fig2-data1.zip › 4hr sidcsdcasdcb -7c1.tif]

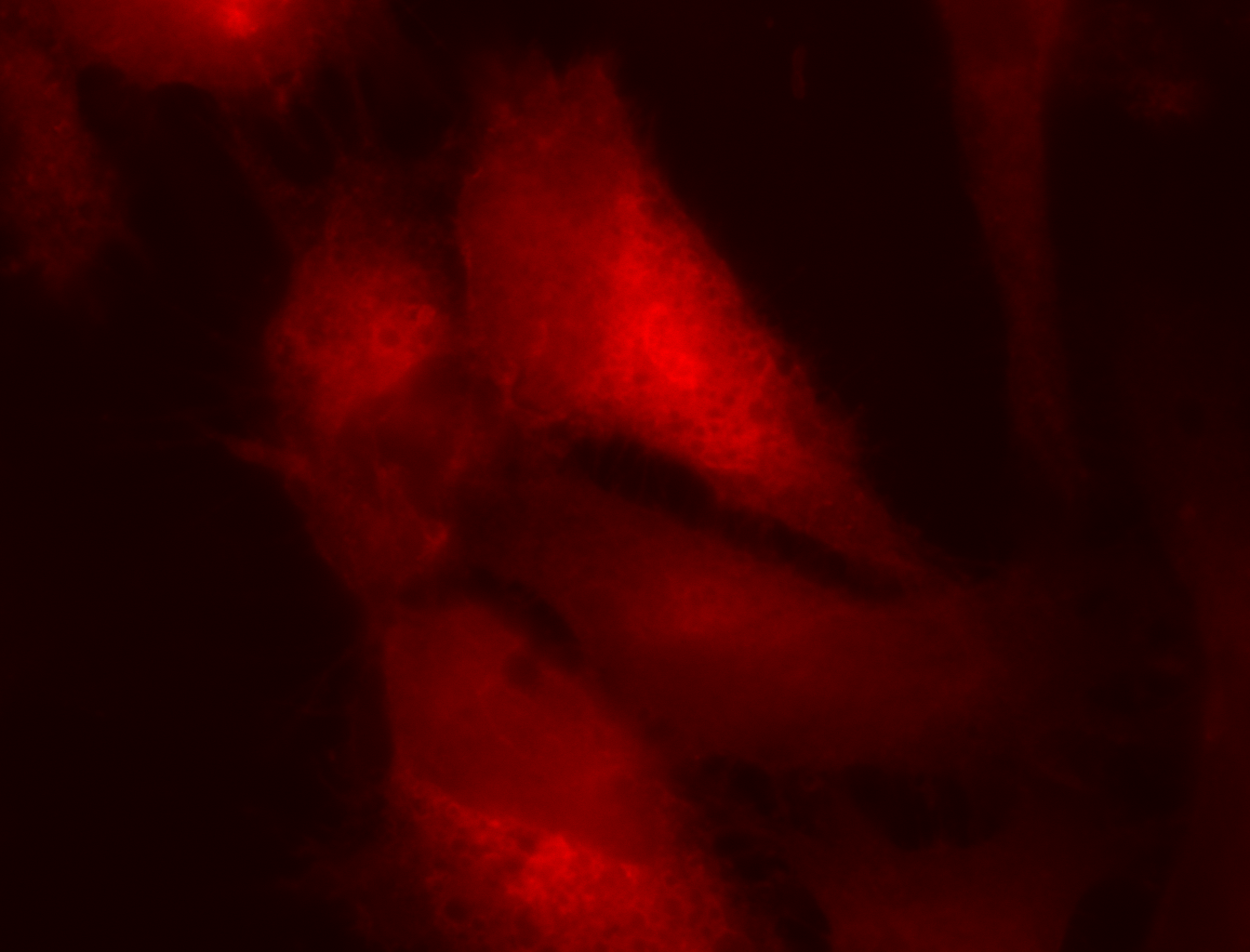

Supplement: Figure 2—source data 1. [file elife-89002-fig2-data1.zip › 4hr sidcsdcasdcb -7c2.tif]

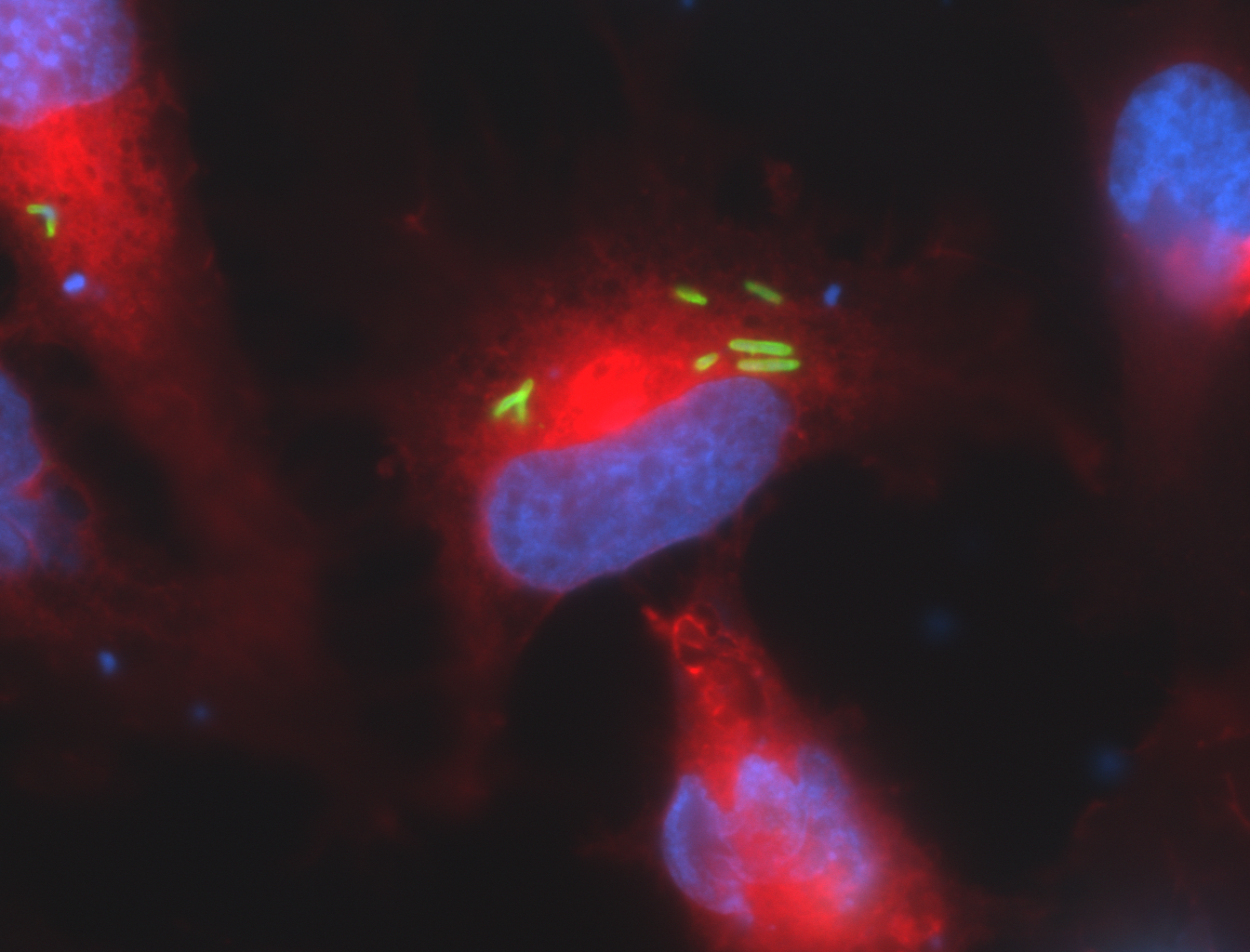

Supplement: Figure 2—source data 1. [file elife-89002-fig2-data1.zip › 4hr sidEs -3.tif]

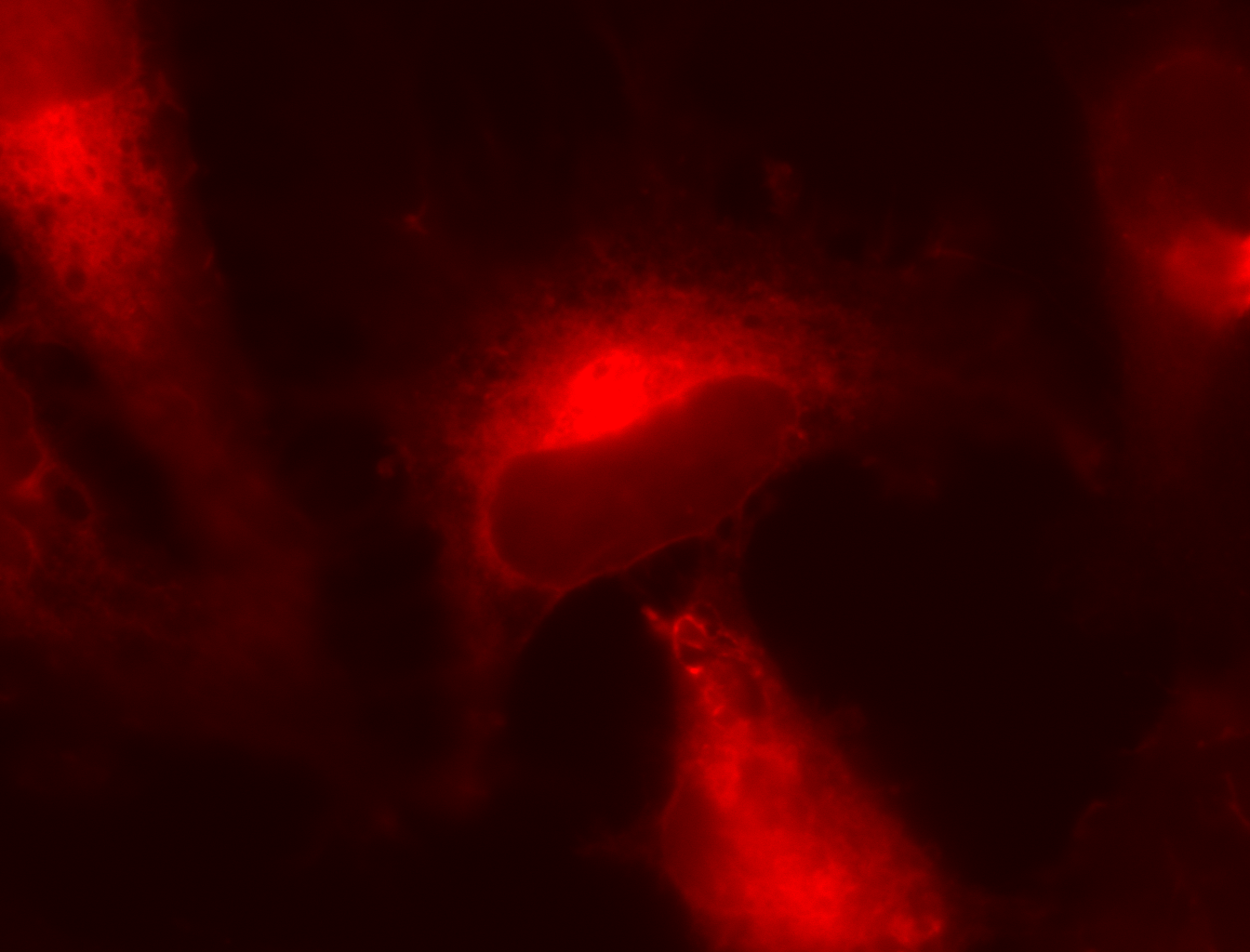

Supplement: Figure 2—source data 1. [file elife-89002-fig2-data1.zip › 4hr sides -3c1.tif]

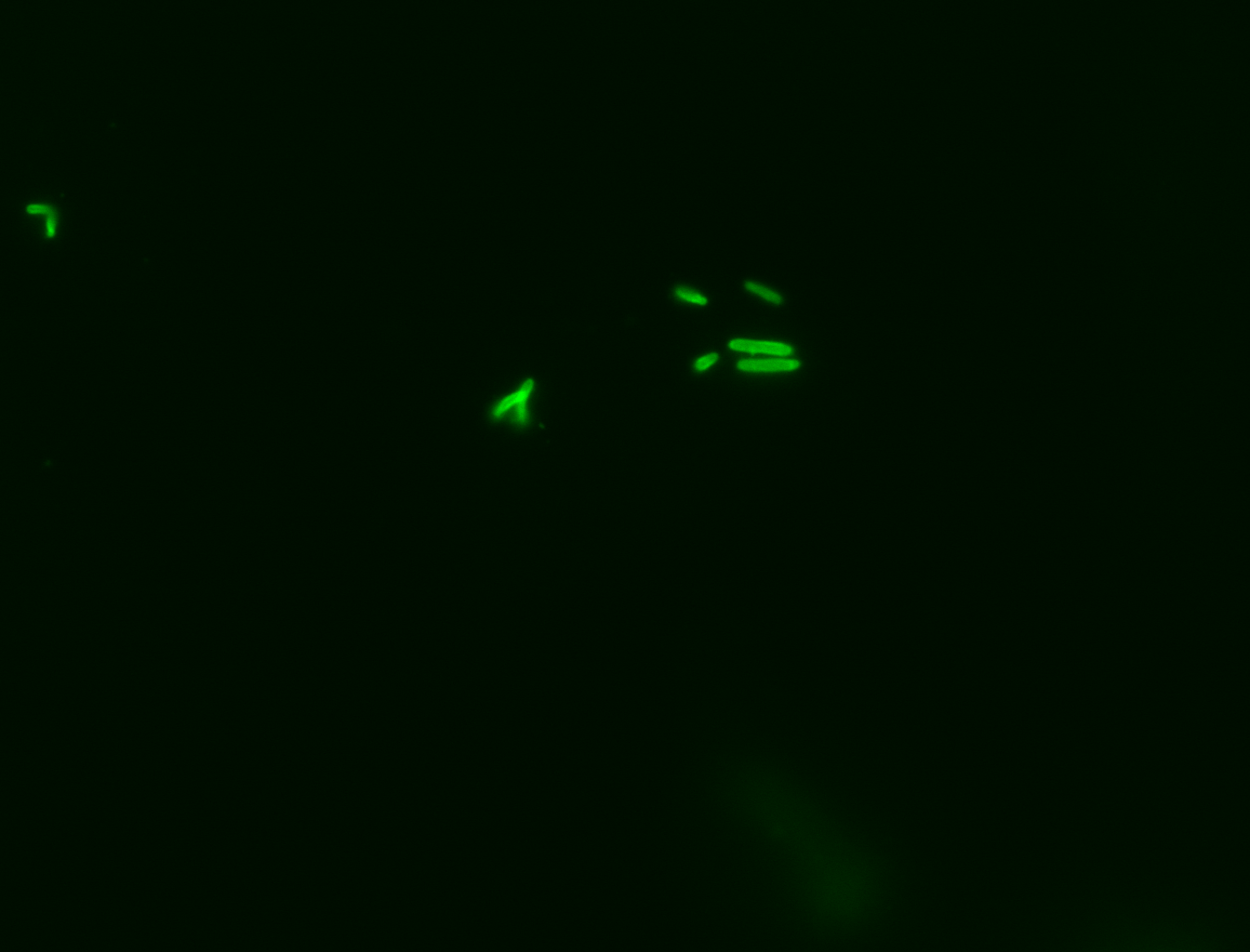

Supplement: Figure 2—source data 1. [file elife-89002-fig2-data1.zip › 4hr sides -3c2.tif]

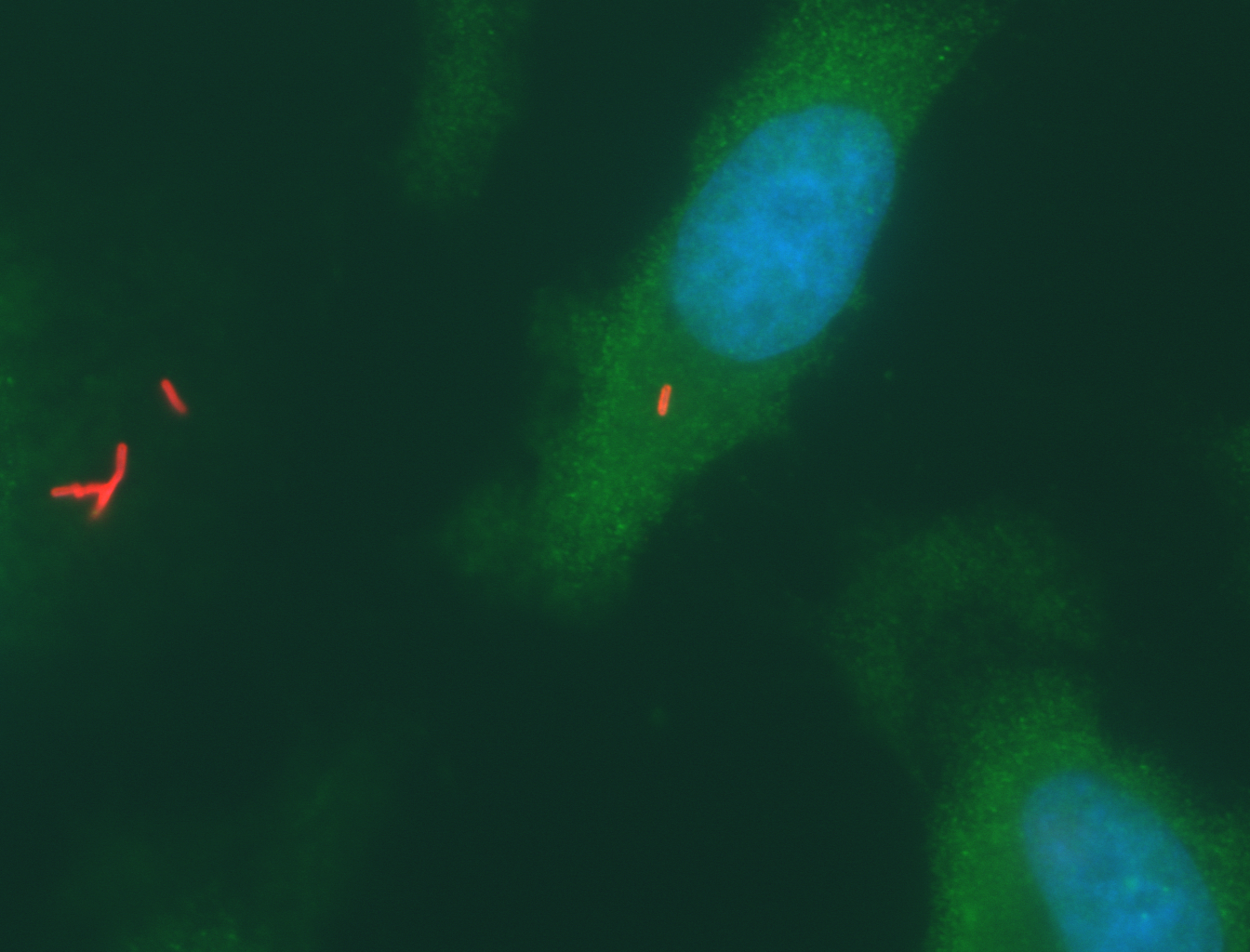

Supplement: Figure 3—source data 1. [file elife-89002-fig3-data1.zip › 1h anti-FK2 FLAG del sidcs 2-.tif]

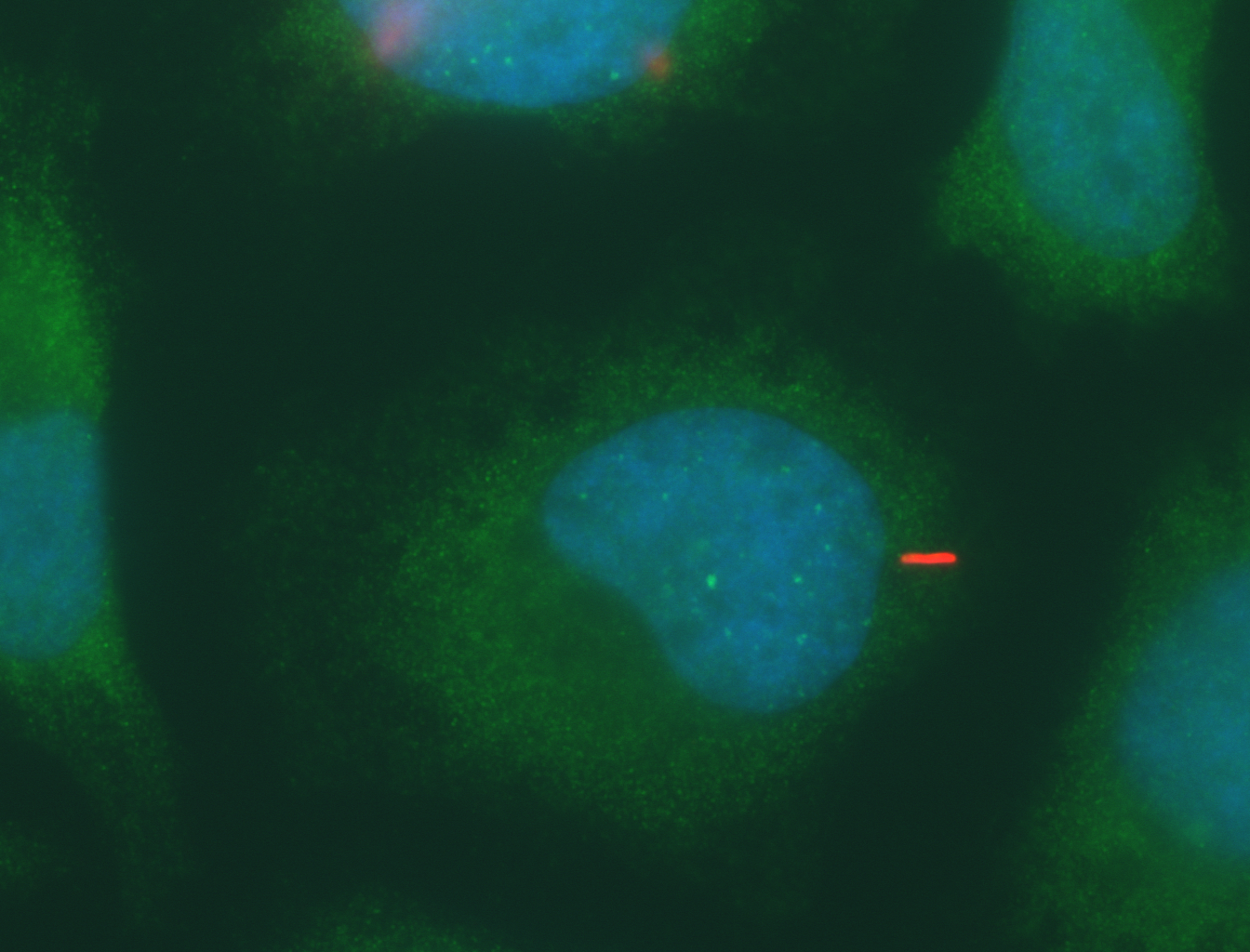

Supplement: Figure 3—source data 1. [file elife-89002-fig3-data1.zip › 1h anti-FK2 FLAG-SdcB C57A del sidcs 3-.tif]

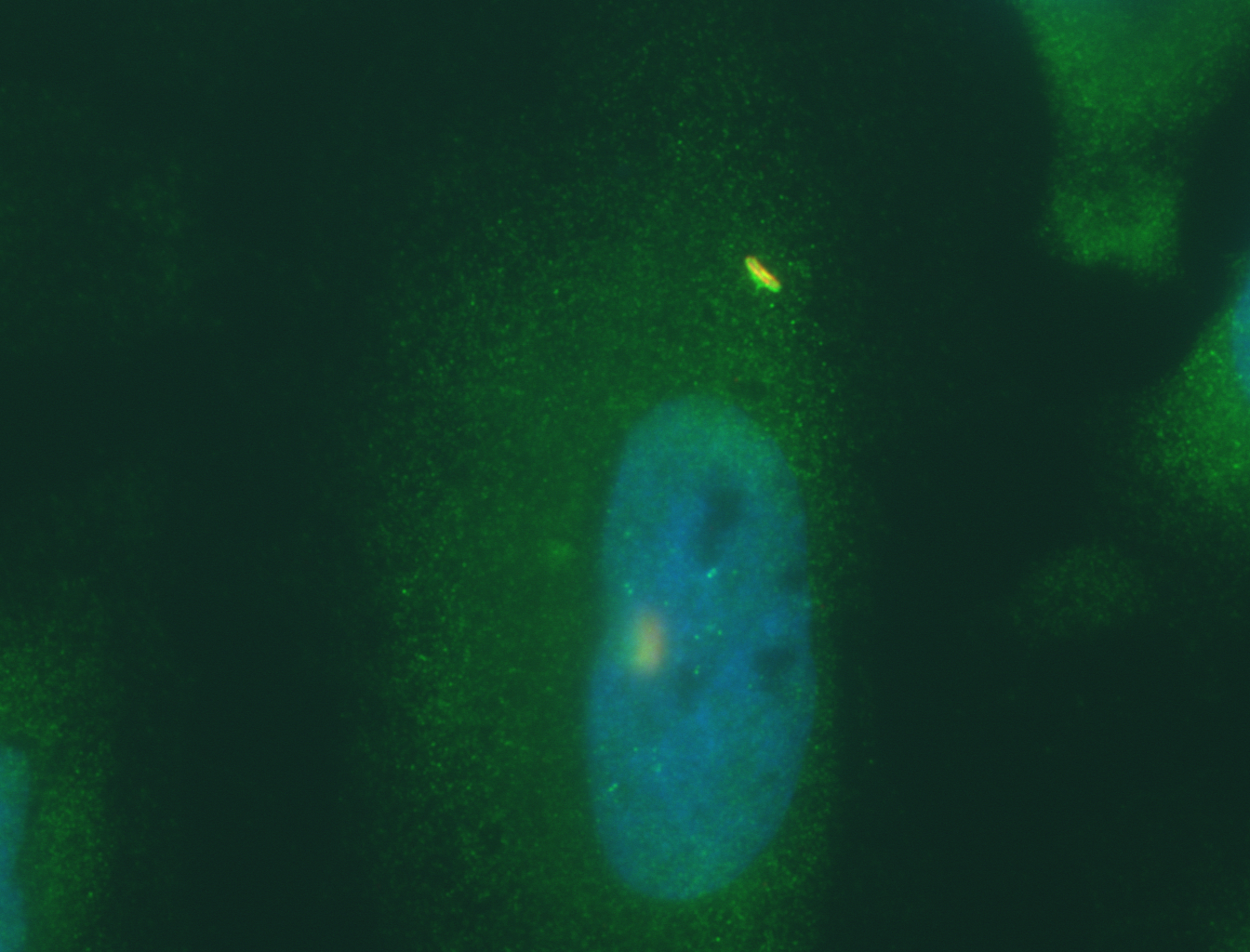

Supplement: Figure 3—source data 1. [file elife-89002-fig3-data1.zip › 1h anti-FK2 FLAG-SdcB del sidcs 5+.tif]

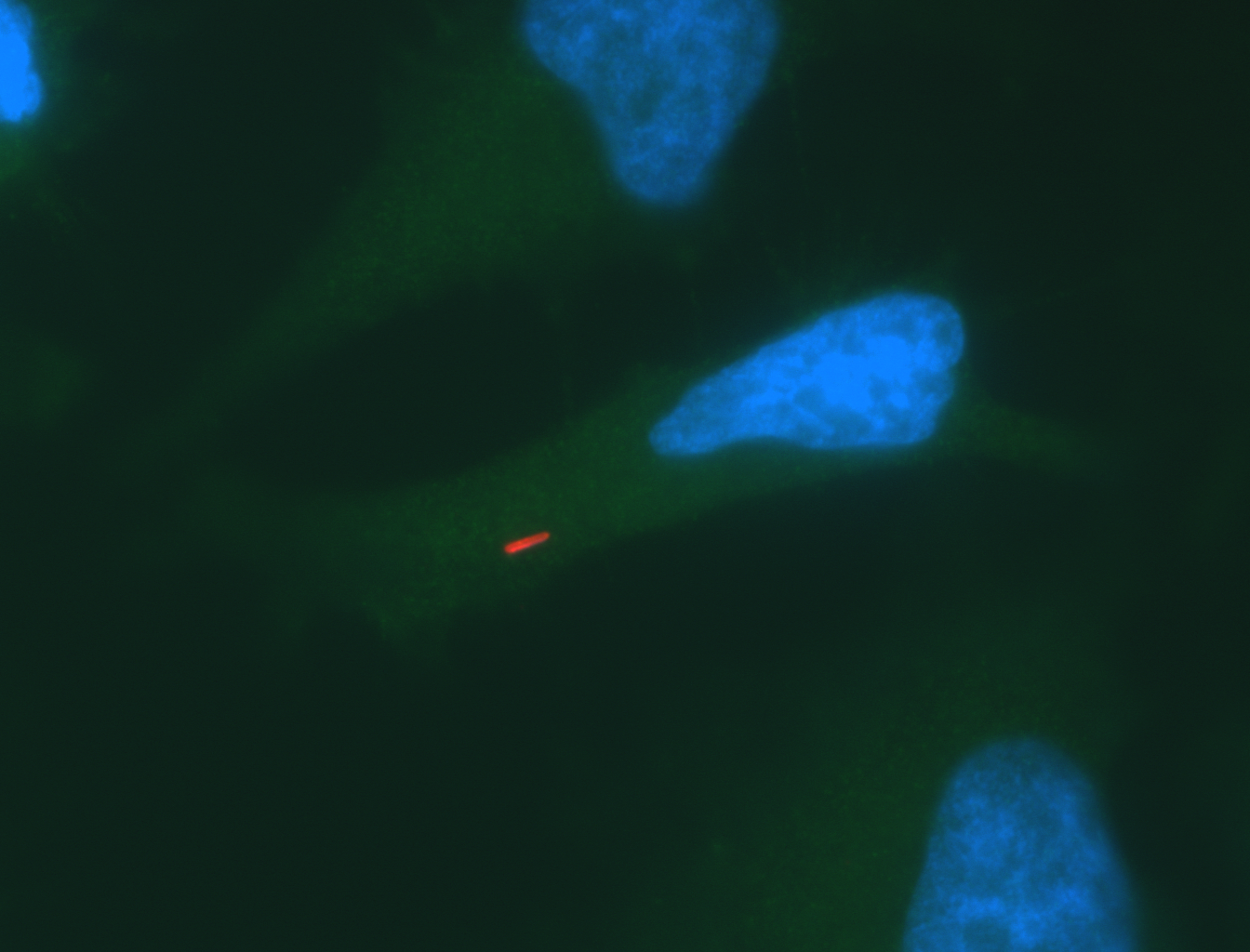

Supplement: Figure 3—source data 1. [file elife-89002-fig3-data1.zip › 1h anti-FLAG FLAG del sidcs 2-.tif]

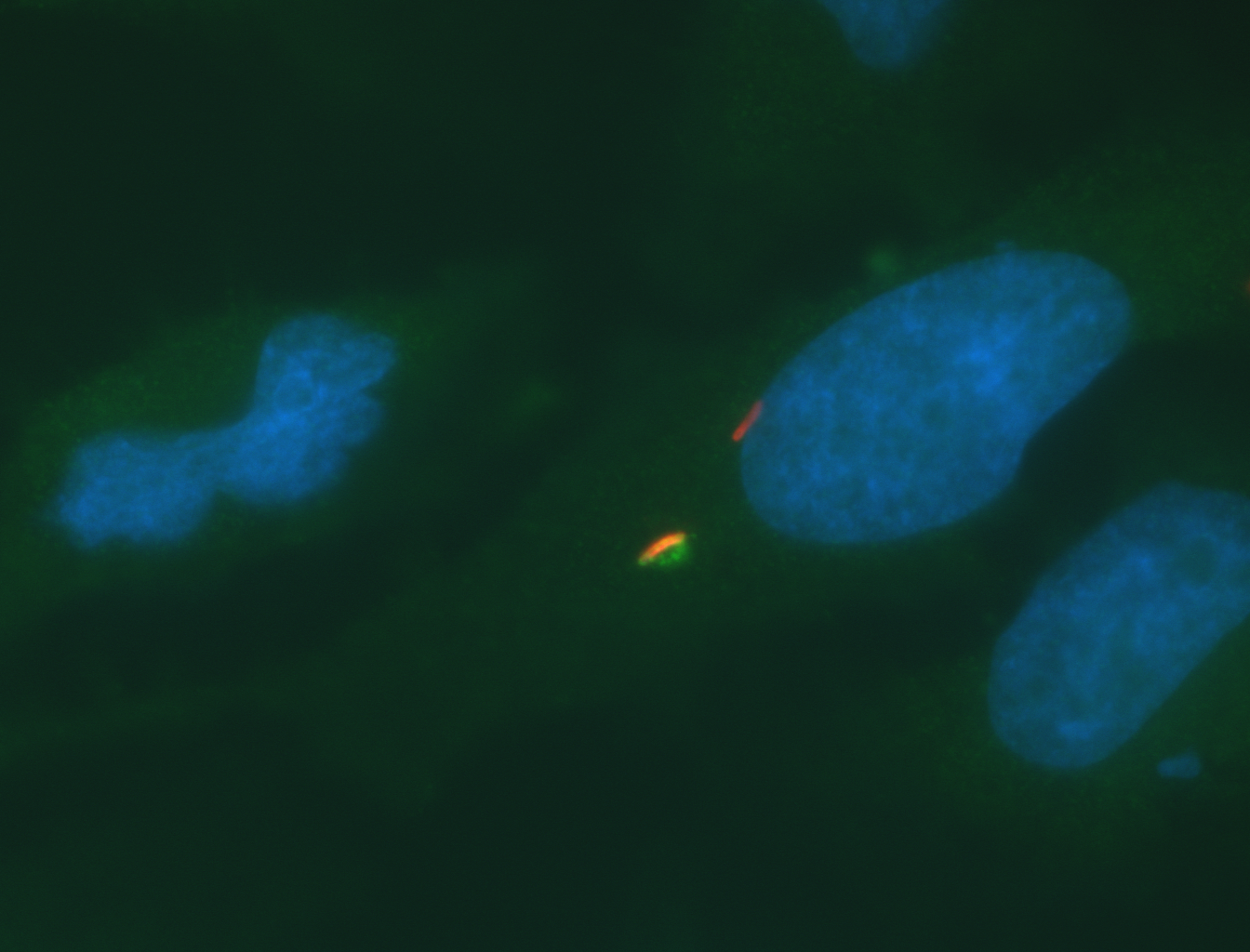

Supplement: Figure 3—source data 1. [file elife-89002-fig3-data1.zip › 1h anti-FLAG FLAG-SdcB C57A del sidcs 4+-.tif]

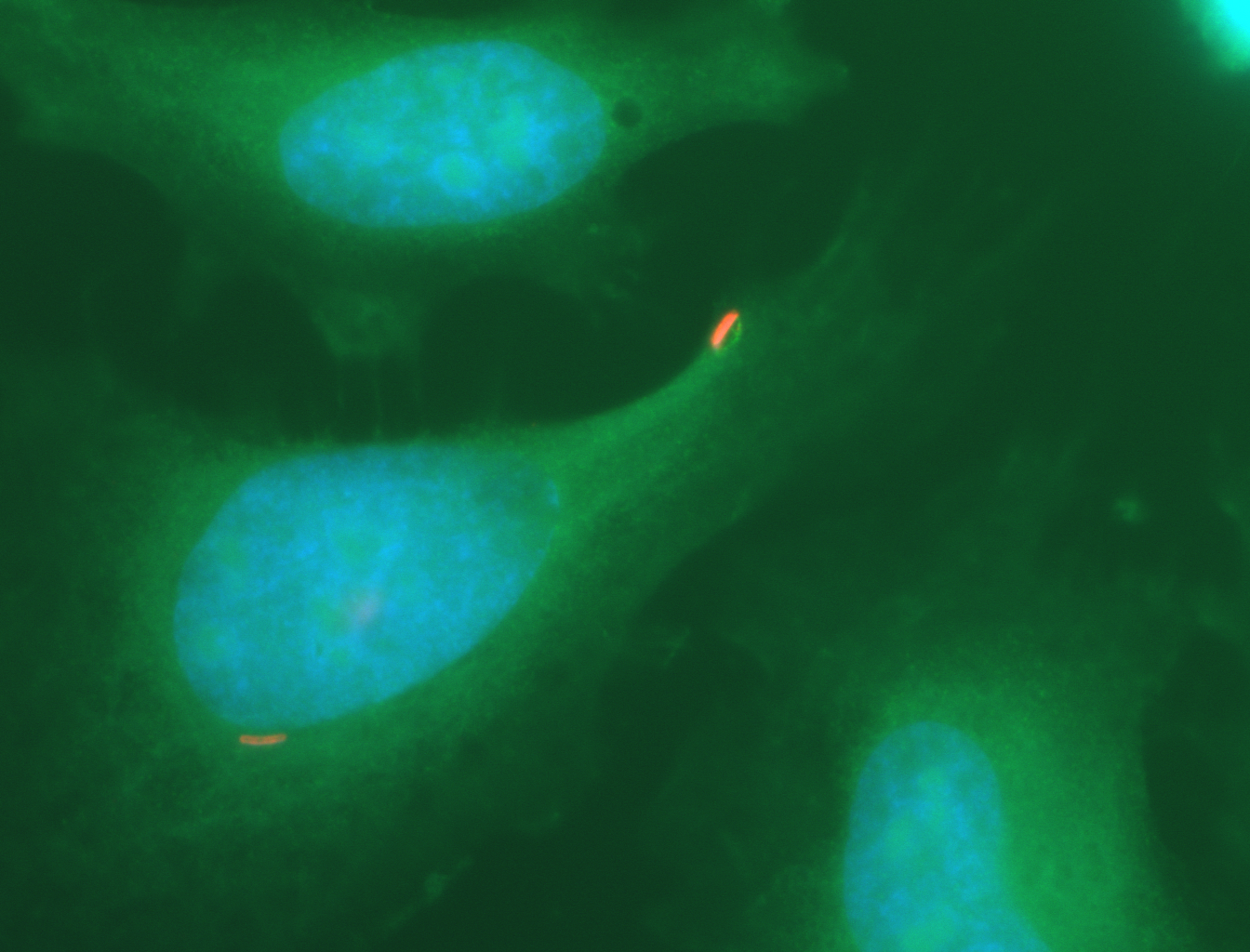

Supplement: Figure 3—source data 1. [file elife-89002-fig3-data1.zip › 1h anti-FLAG FLAG-SdcB del sidcs 1+.tif]

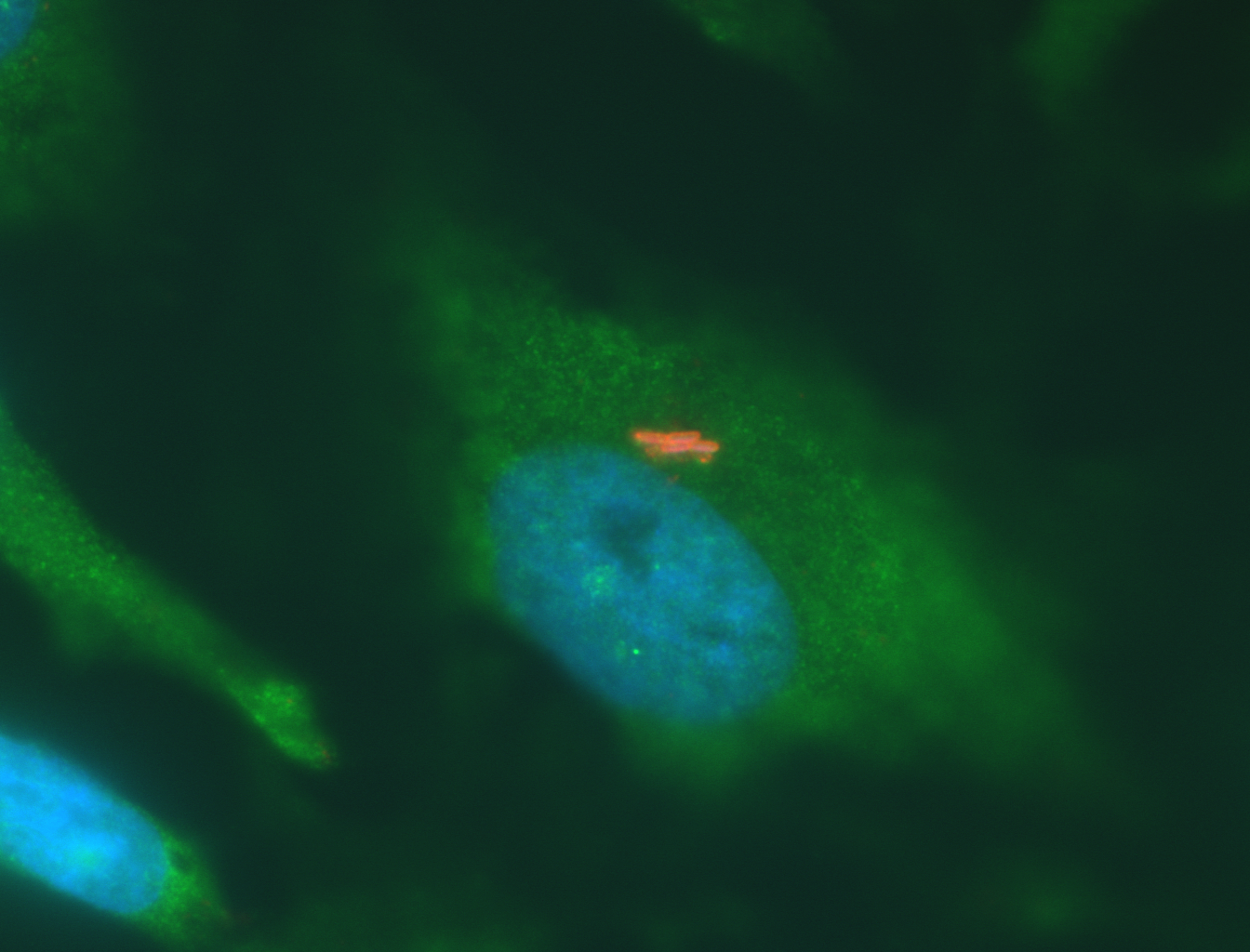

Supplement: Figure 3—source data 2. [file elife-89002-fig3-data2.zip › 7h anti-FK2 FLAG del sidcs 2-.tif]

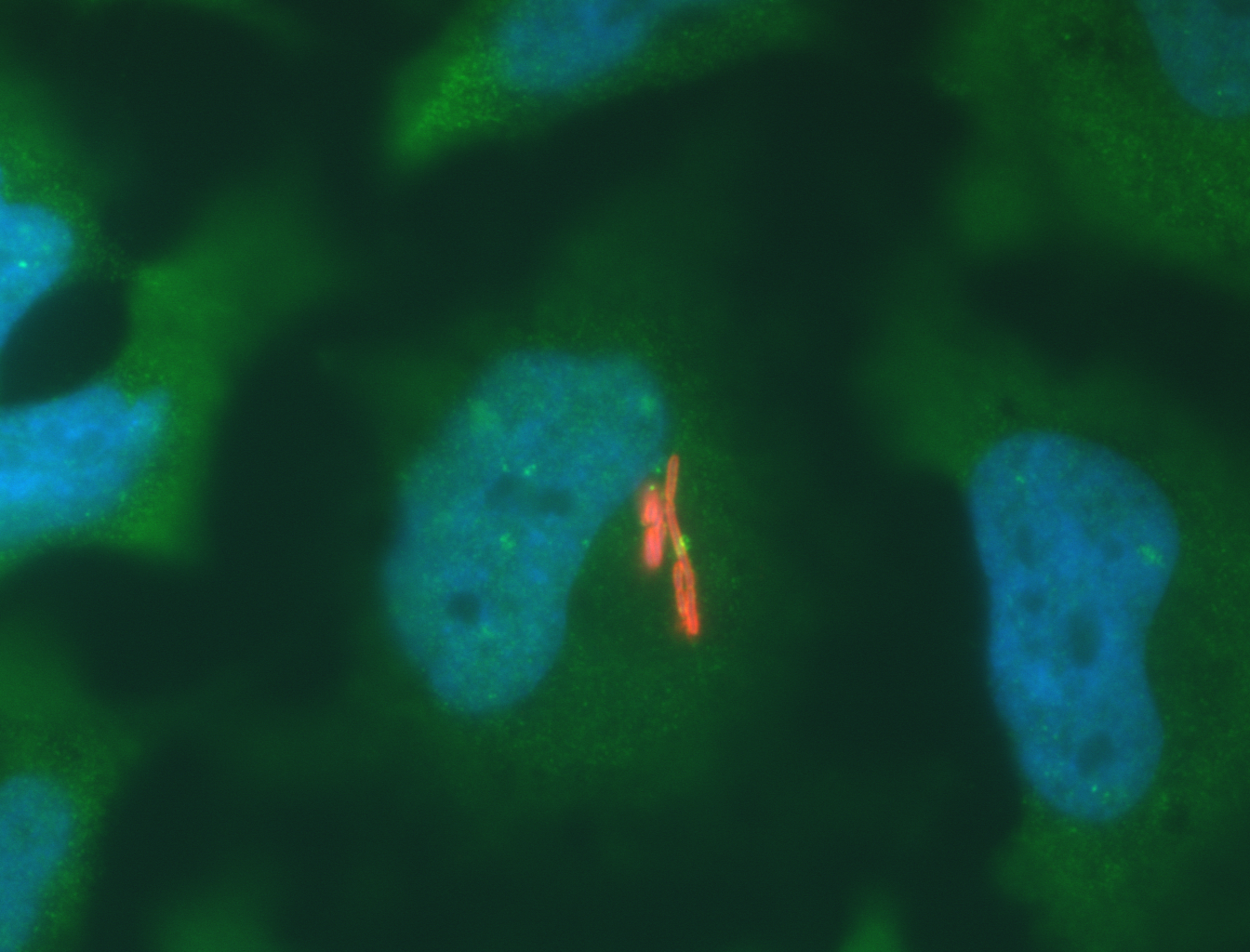

Supplement: Figure 3—source data 2. [file elife-89002-fig3-data2.zip › 7h anti-FK2 FLAG-SdcB C57A del sidcs 3-.tif]

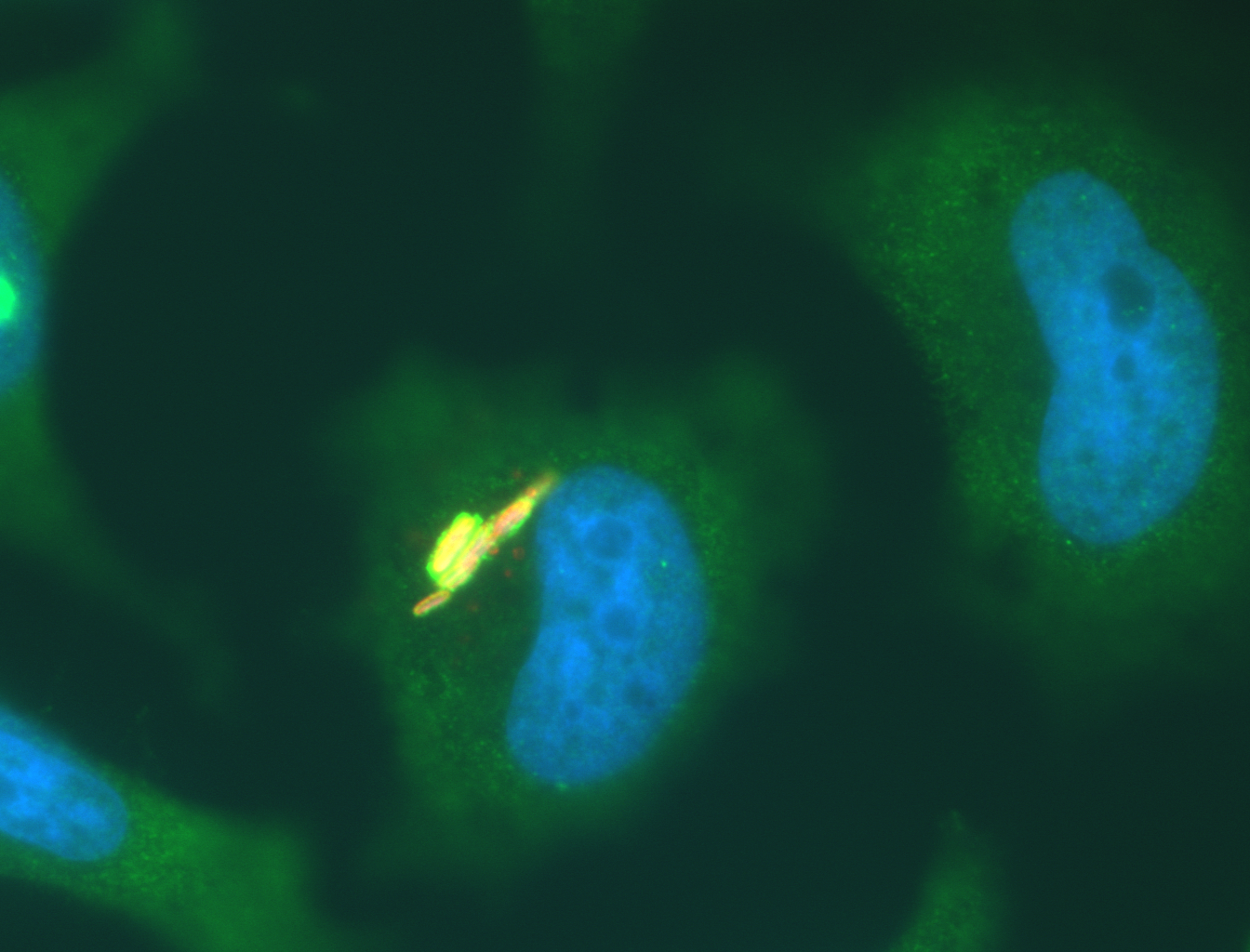

Supplement: Figure 3—source data 2. [file elife-89002-fig3-data2.zip › 7h anti-FK2 FLAG-SdcB del sidcs 2+.tif]

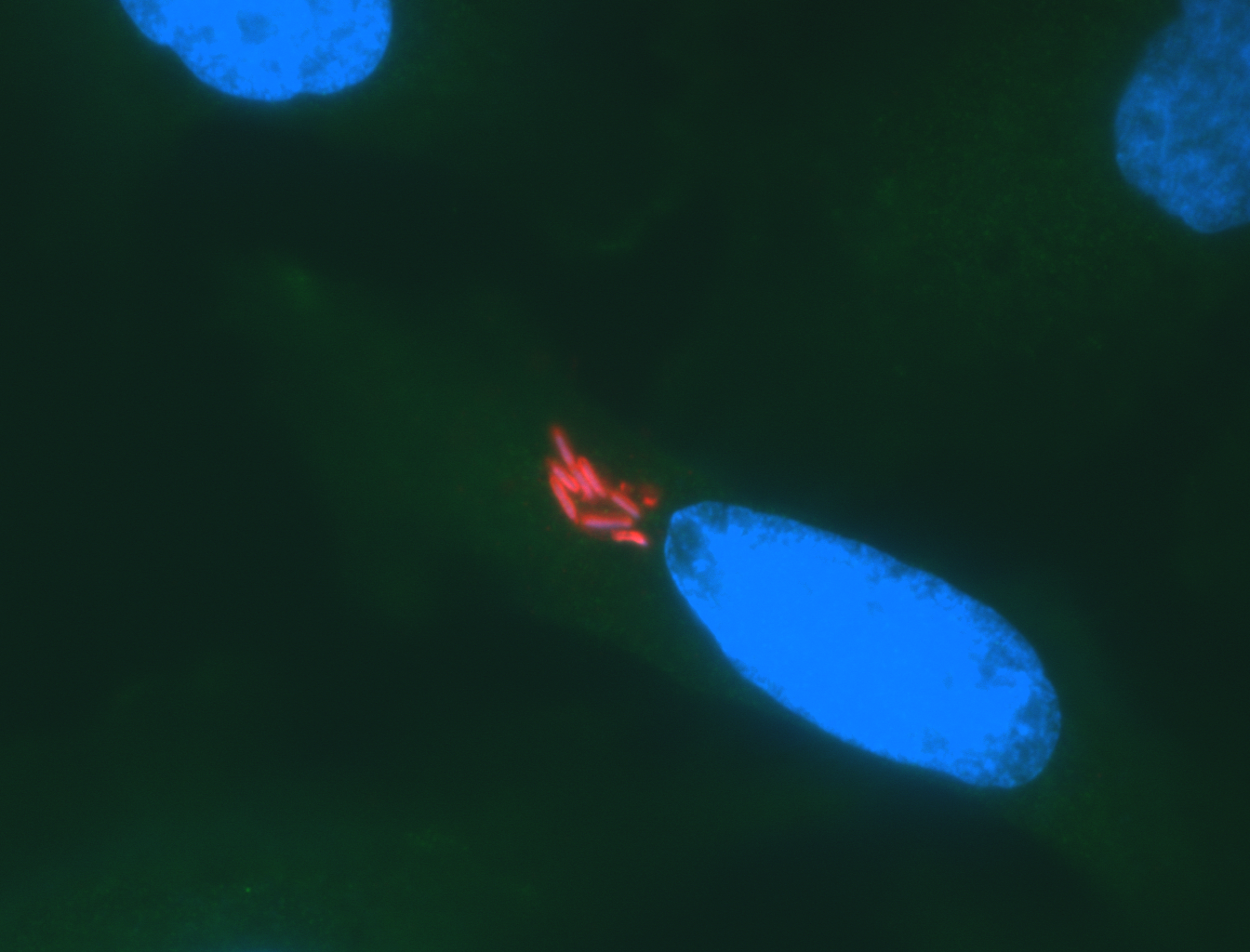

Supplement: Figure 3—source data 2. [file elife-89002-fig3-data2.zip › 7h anti-FLAG FLAG del sidcs 1-.tif]

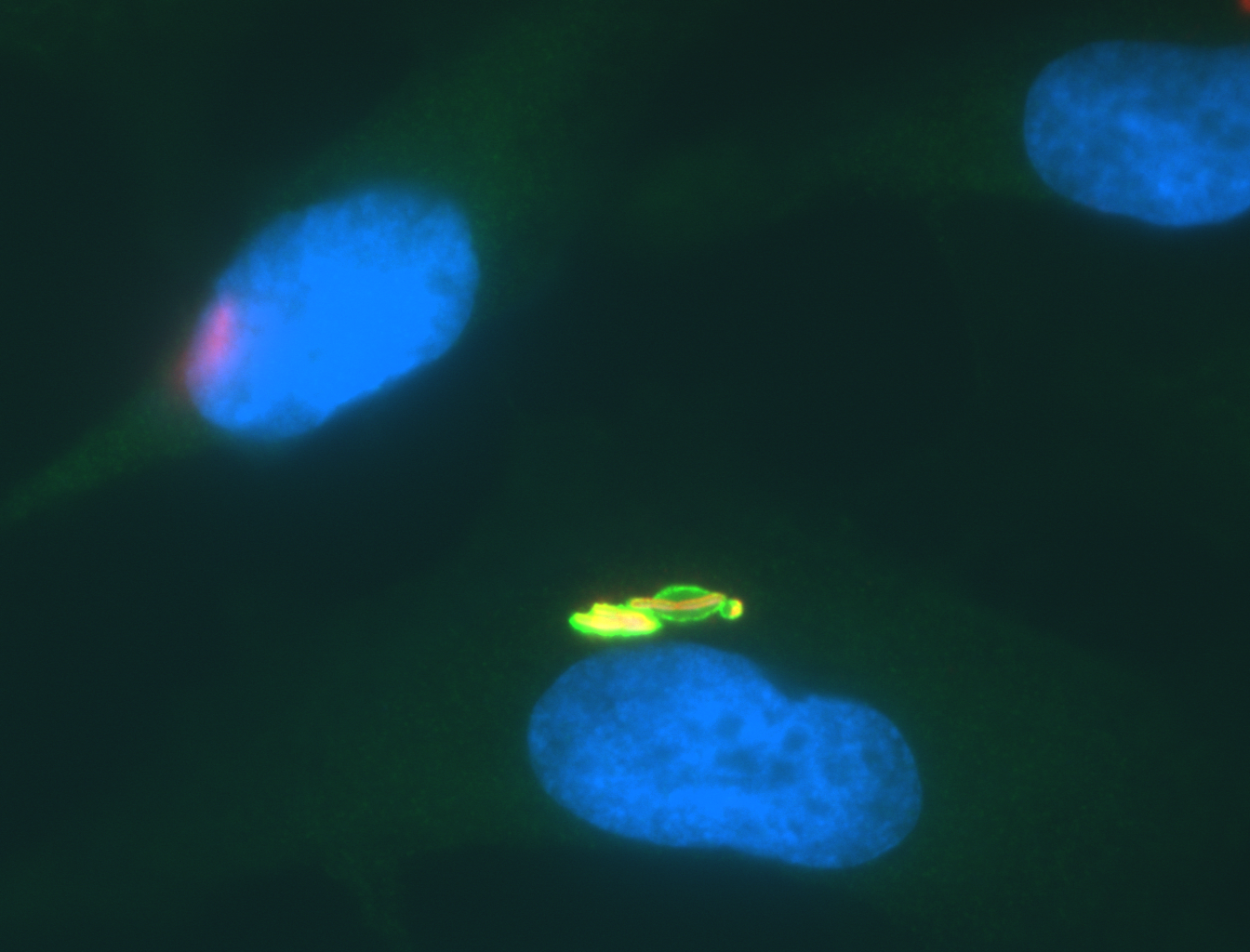

Supplement: Figure 3—source data 2. [file elife-89002-fig3-data2.zip › 7h anti-FLAG FLAG-SdcB C57A del sidcs 3+.tif]

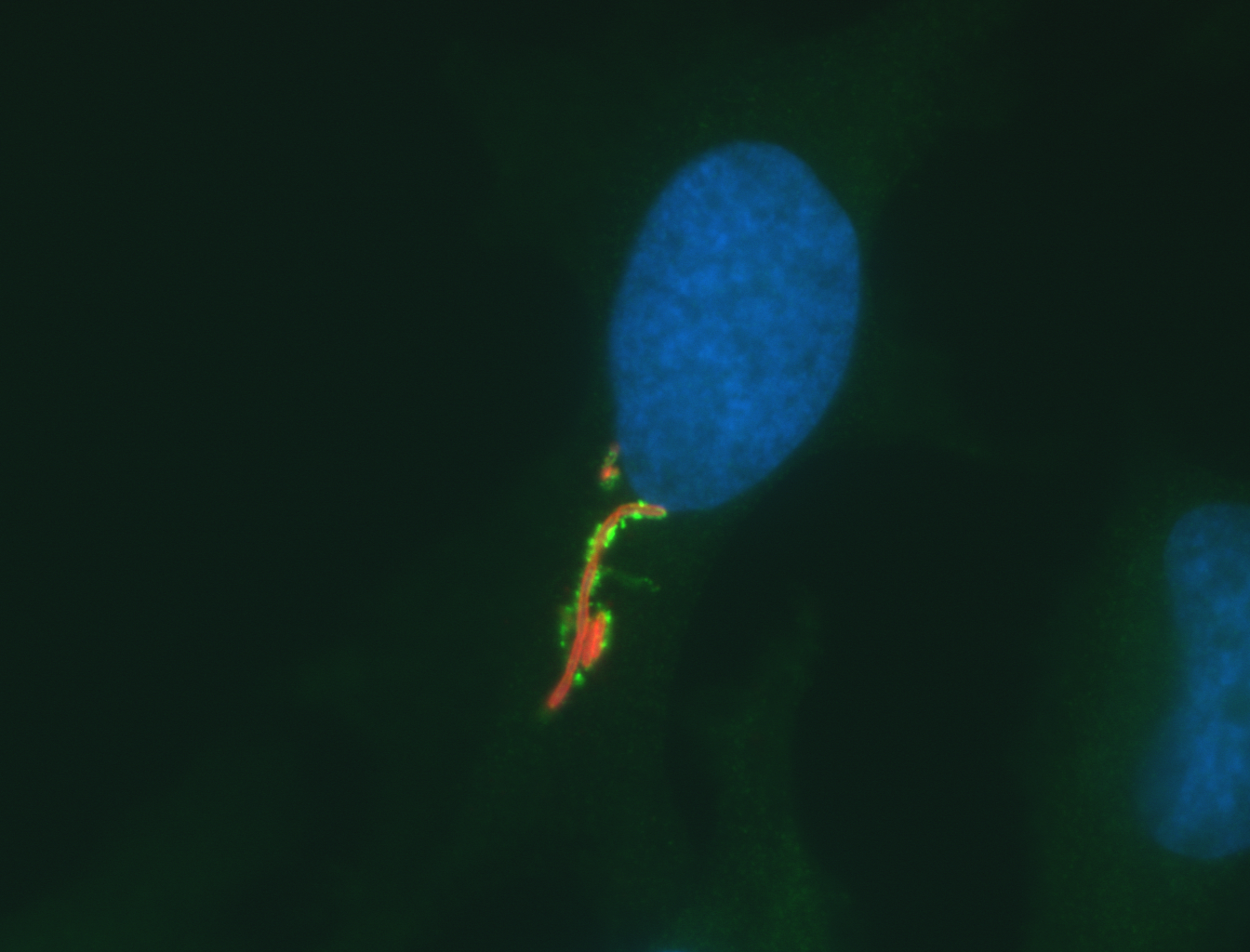

Supplement: Figure 3—source data 2. [file elife-89002-fig3-data2.zip › 7h anti-FLAG FLAG-SdcB del sidcs 2+.tif]

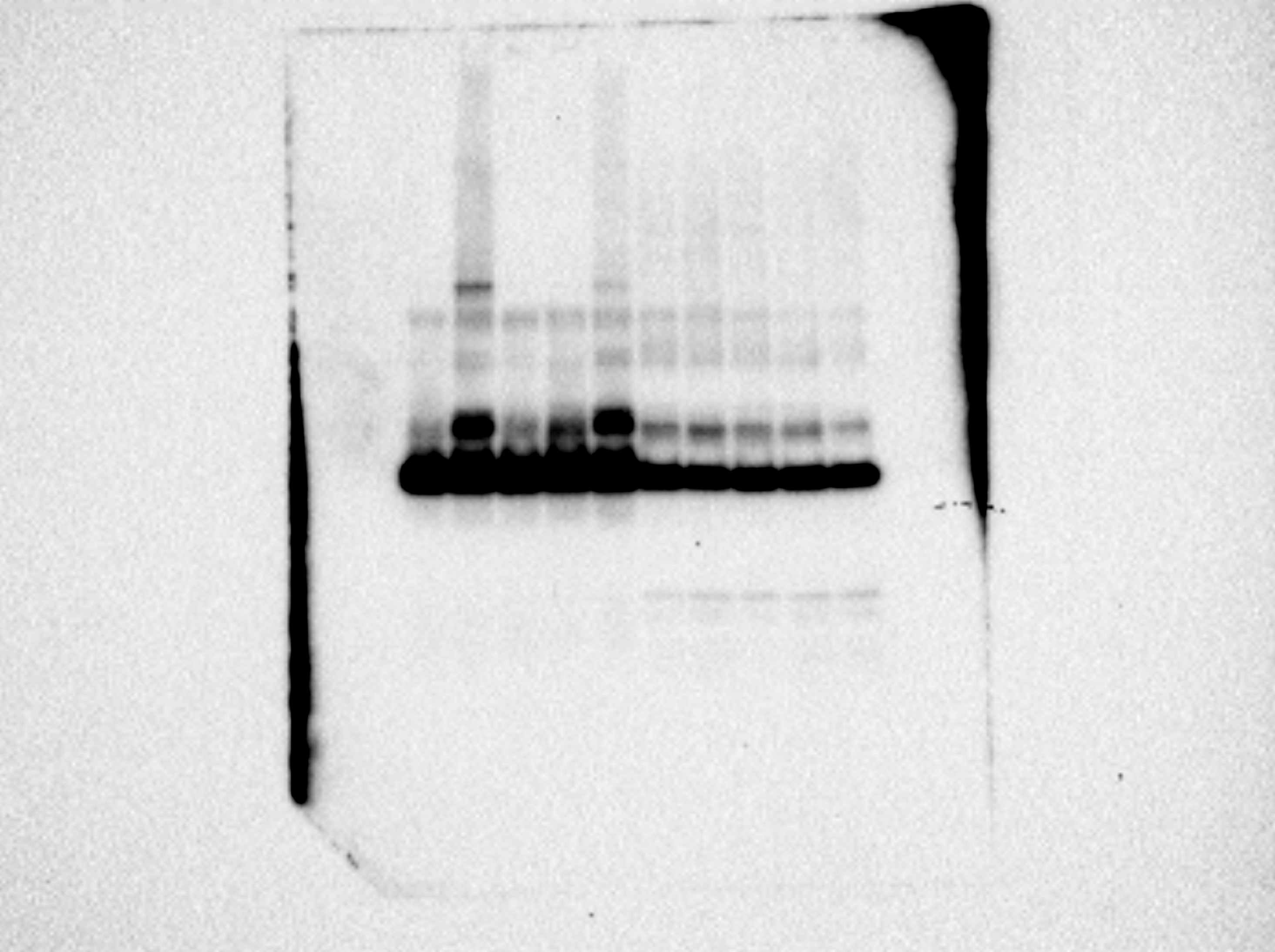

Supplement: Figure 4—source data 1. [file elife-89002-fig4-data1.zip › IP FLAG anti-FLAGm_Exposure_60.0sec.jpg]

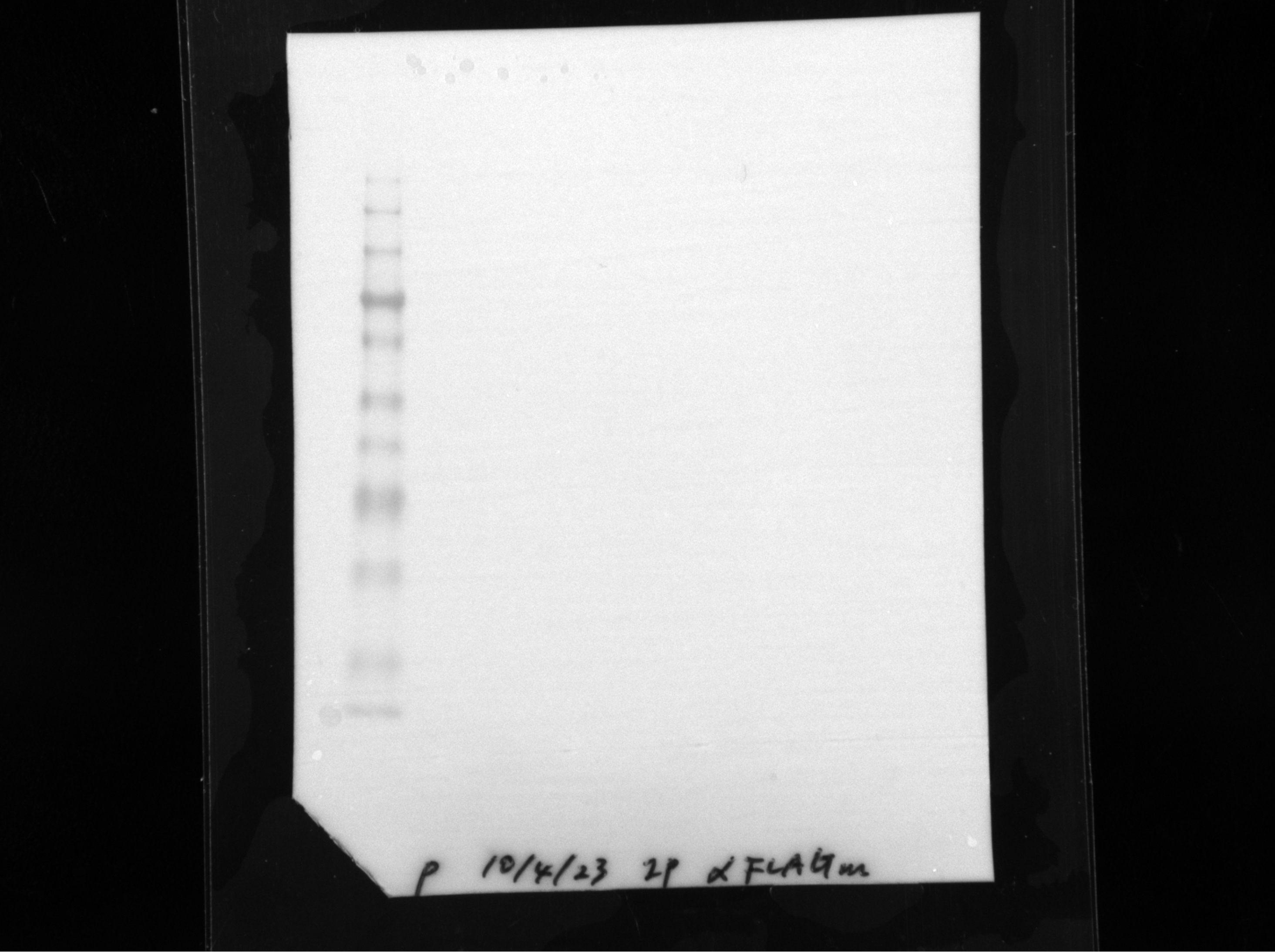

Supplement: Figure 4—source data 1. [file elife-89002-fig4-data1.zip › IP FLAG anti-FLAGm_Marker.jpg]

**a**

1 h infection

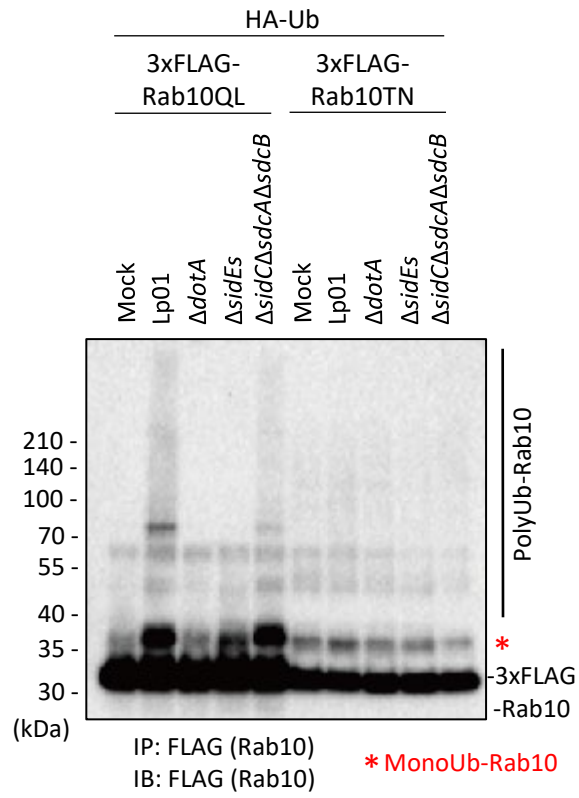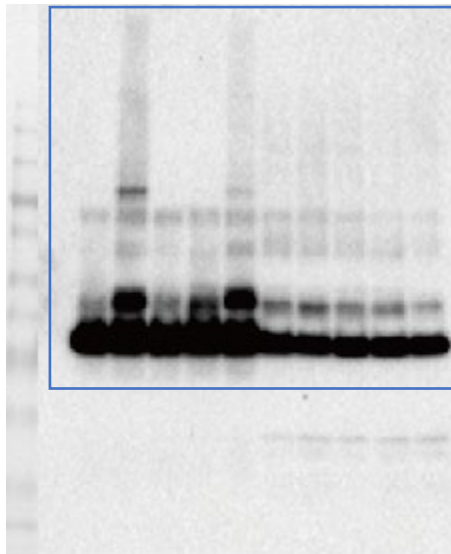

**Figure 4a**

Supplement: Figure 4—source data 2. [file elife-89002-fig4-data2.pdf]

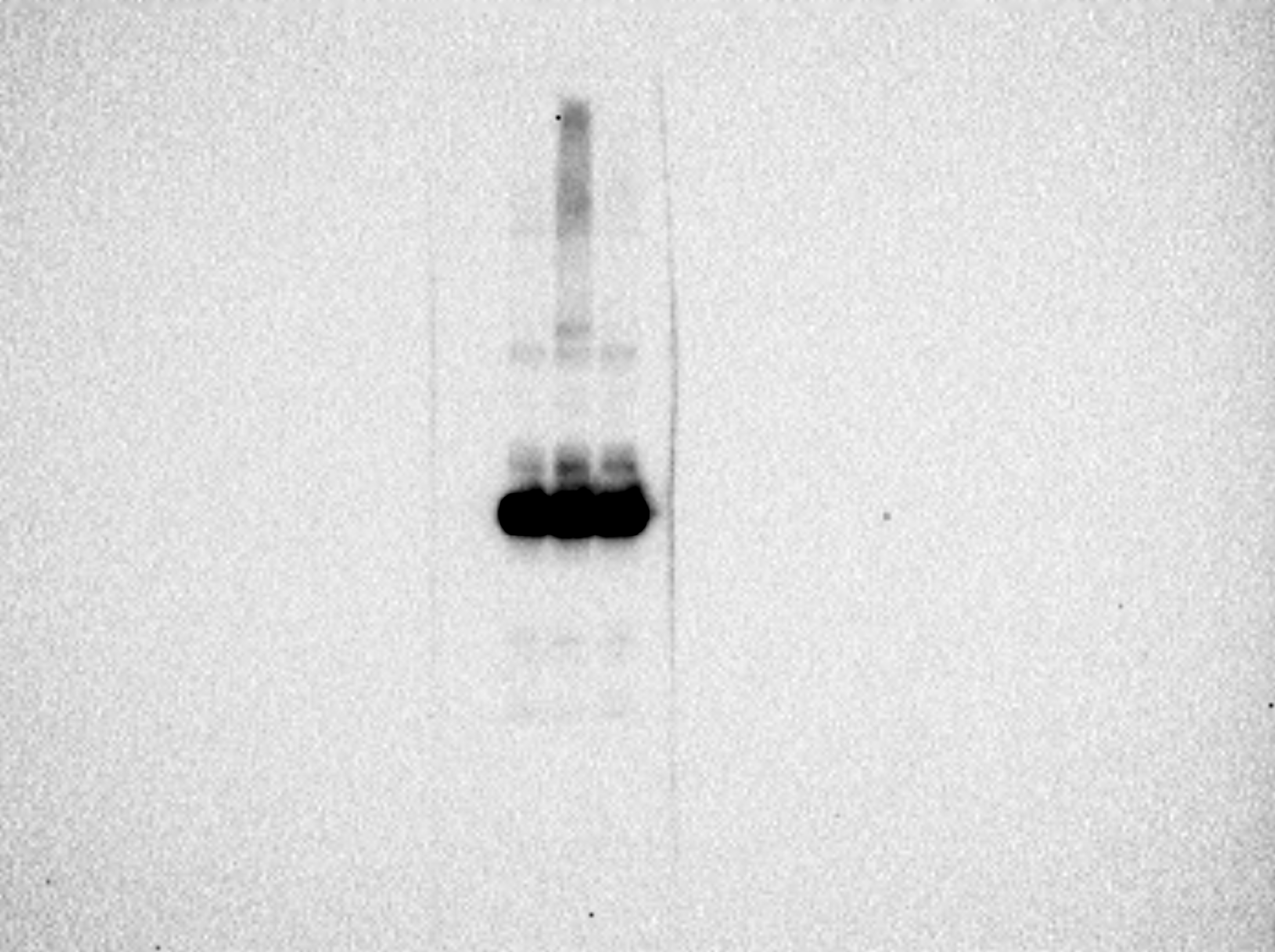

Supplement: Figure 4—source data 3. [file elife-89002-fig4-data3.zip › IP FLAG anti-FLAGm_Exposure_60.0sec.jpg]

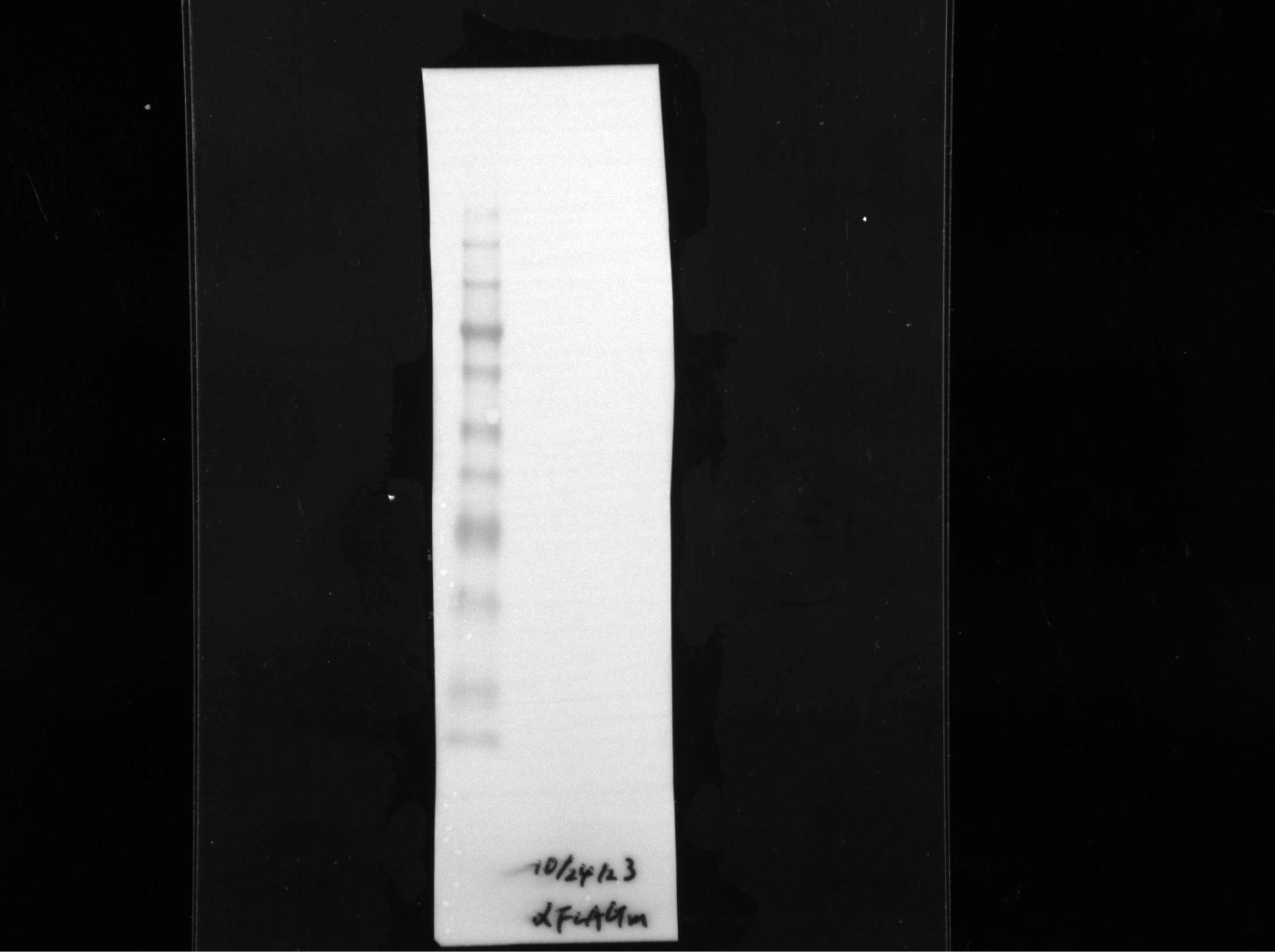

Supplement: Figure 4—source data 3. [file elife-89002-fig4-data3.zip › IP FLAG anti-FLAGm_Marker.jpg]

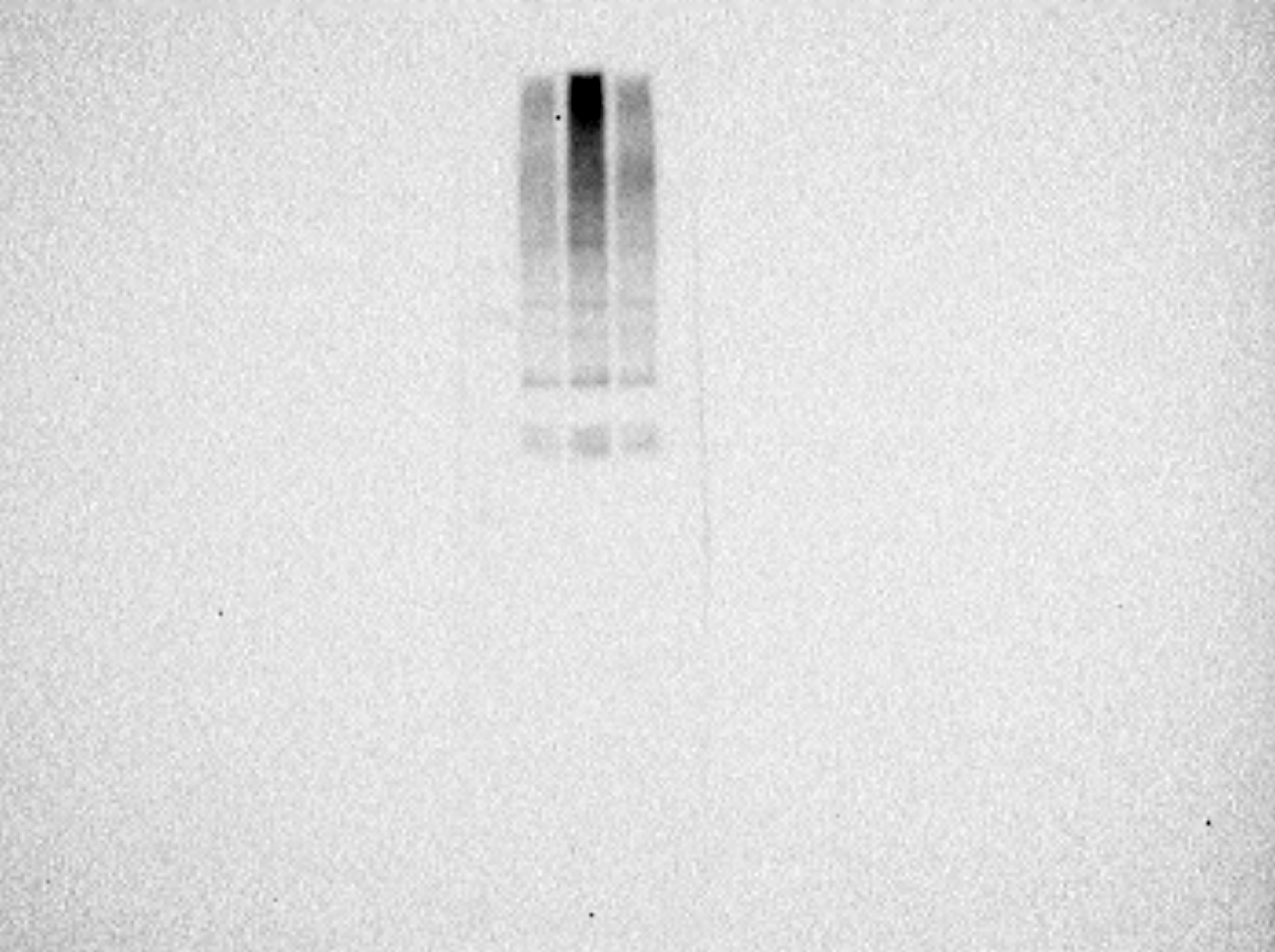

Supplement: Figure 4—source data 3. [file elife-89002-fig4-data3.zip › IP FLAG anti-HArb_Exposure_60.0sec.jpg]

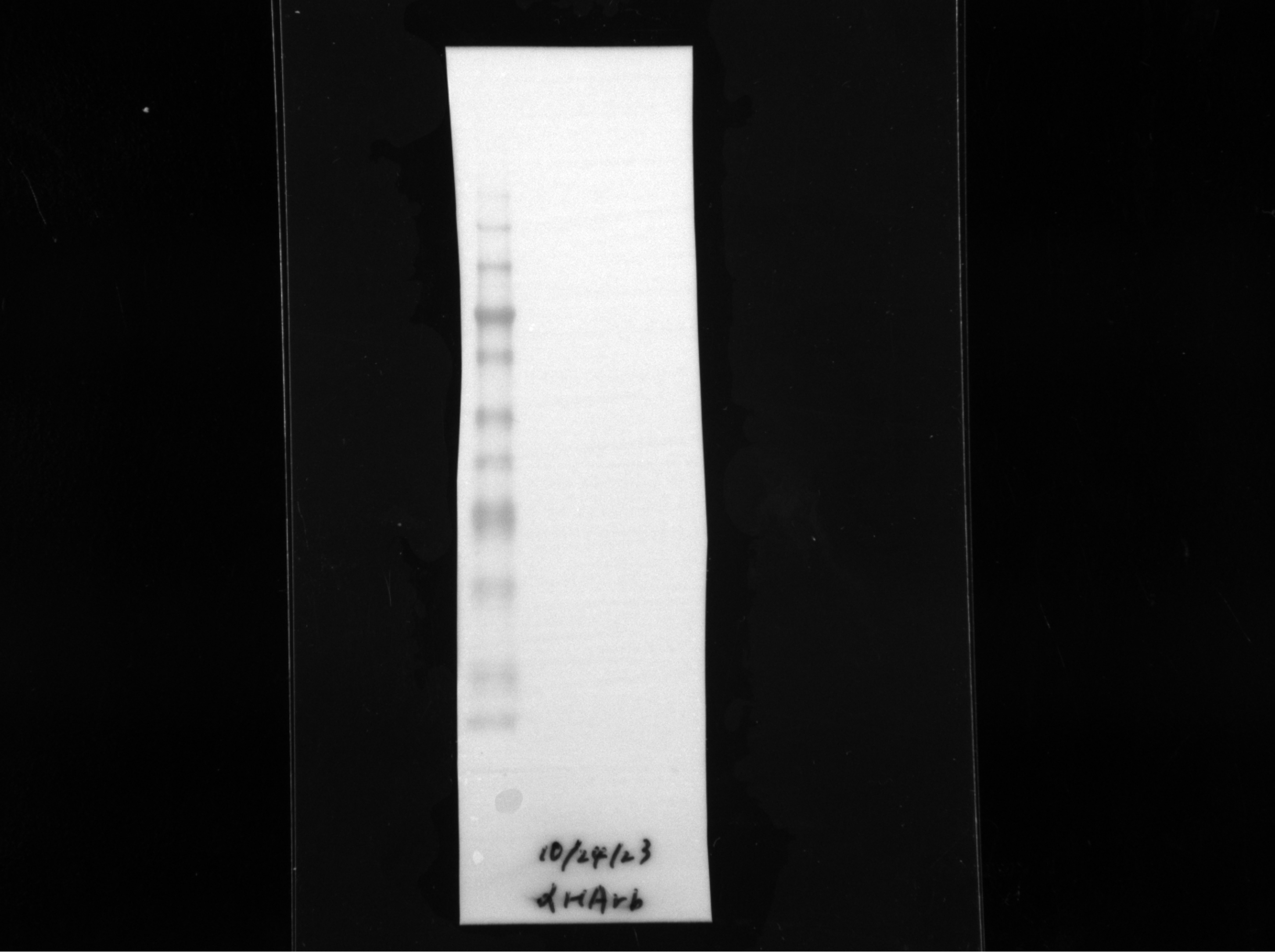

Supplement: Figure 4—source data 3. [file elife-89002-fig4-data3.zip › IP FLAG anti-HArb_Marker.jpg]

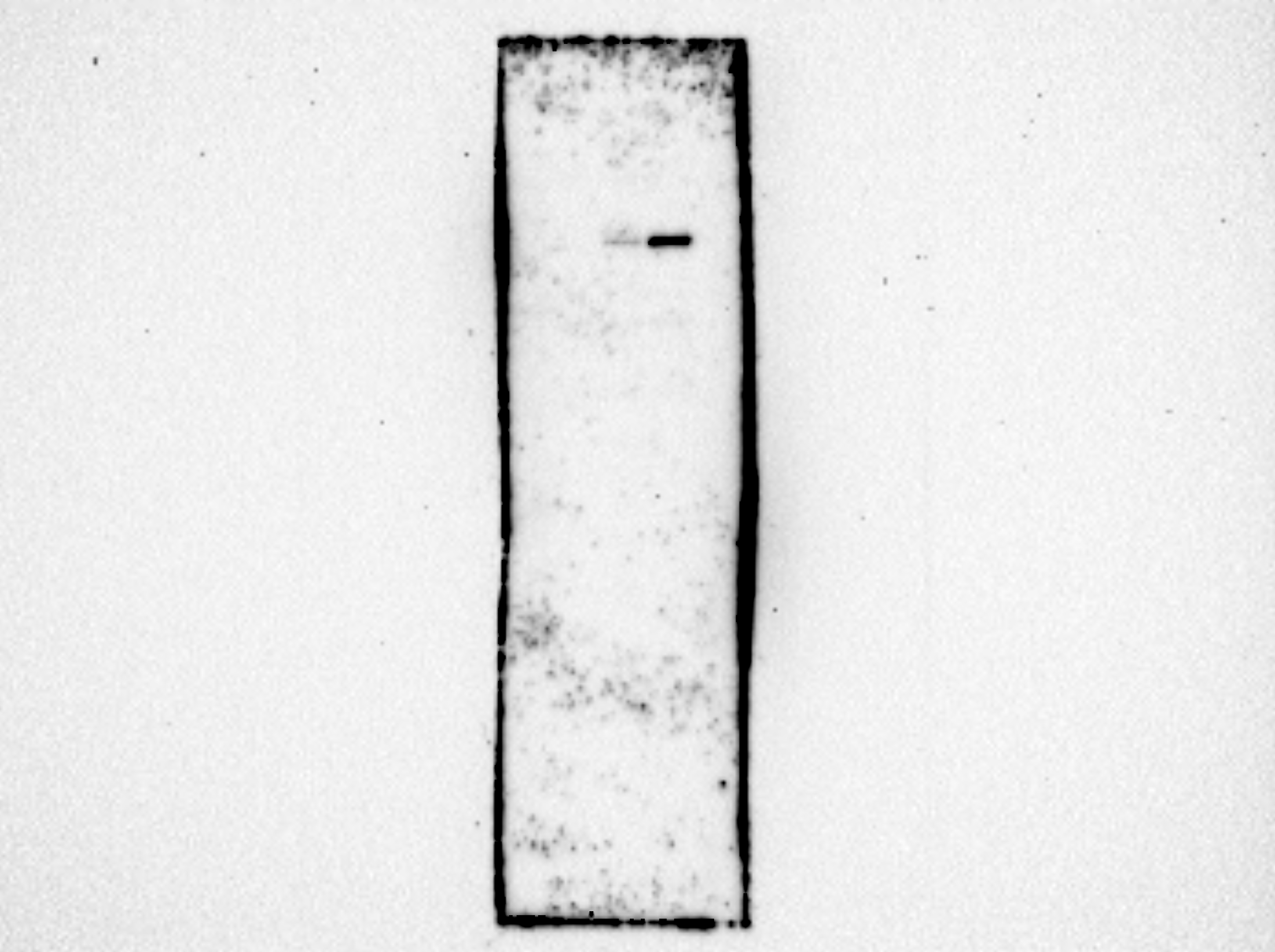

Supplement: Figure 4—source data 3. [file elife-89002-fig4-data3.zip › IP Myc anti-Myc super_Exposure_300.0sec.jpg]

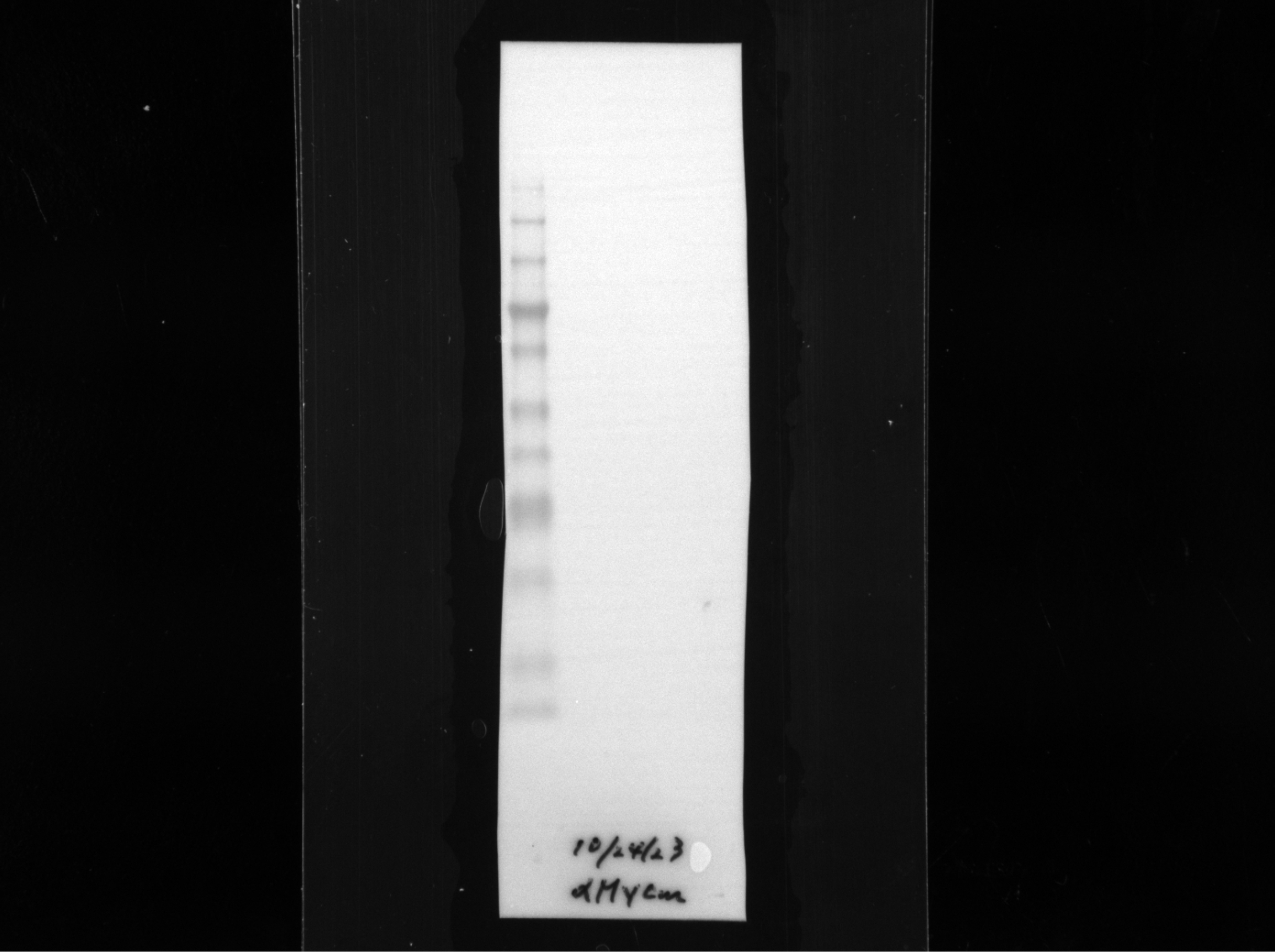

Supplement: Figure 4—source data 3. [file elife-89002-fig4-data3.zip › IP Myc anti-Myc super_Marker.jpg]

**C**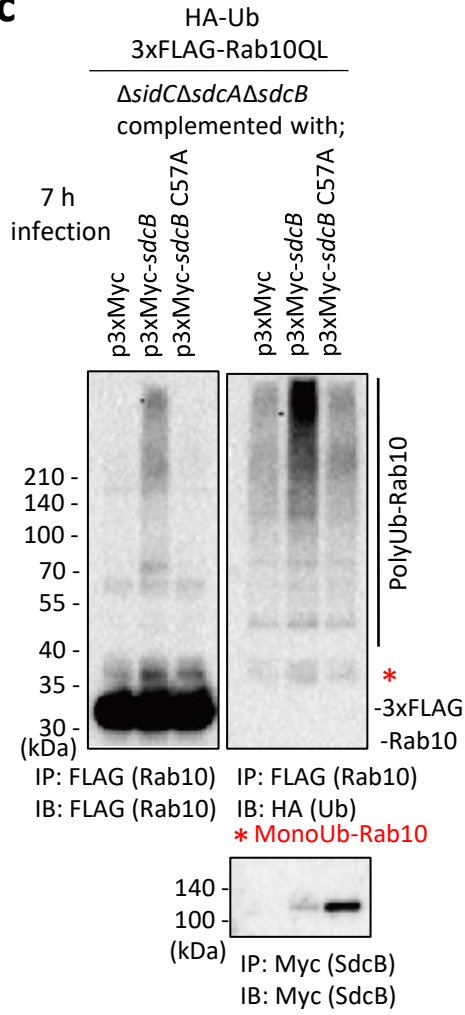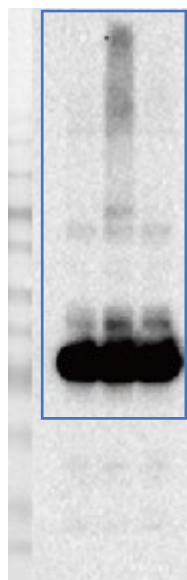

**Figure 4c**  
top left

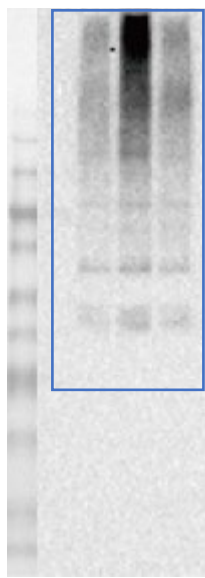

**Figure 4c**  
top right

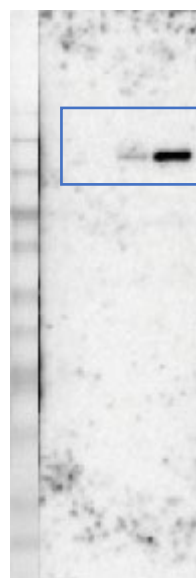

**Figure 4c**  
bottom

Supplement: Figure 4—source data 4. [file elife-89002-fig4-data4.pdf]

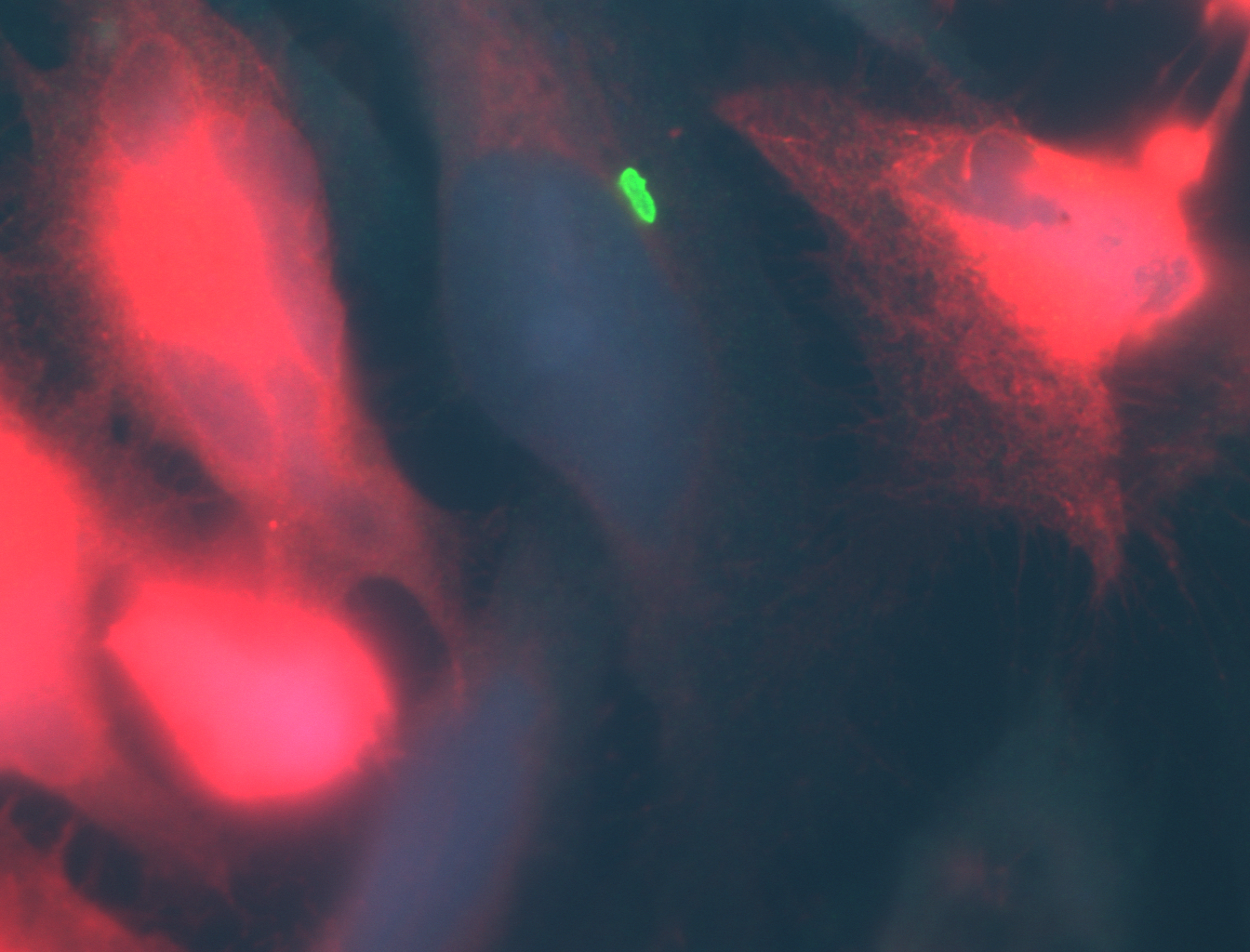

Supplement: Figure 4—source data 5. [file elife-89002-fig4-data5.zip › p3xFLAG-sdcB C57A del sidCs 9-.tif]

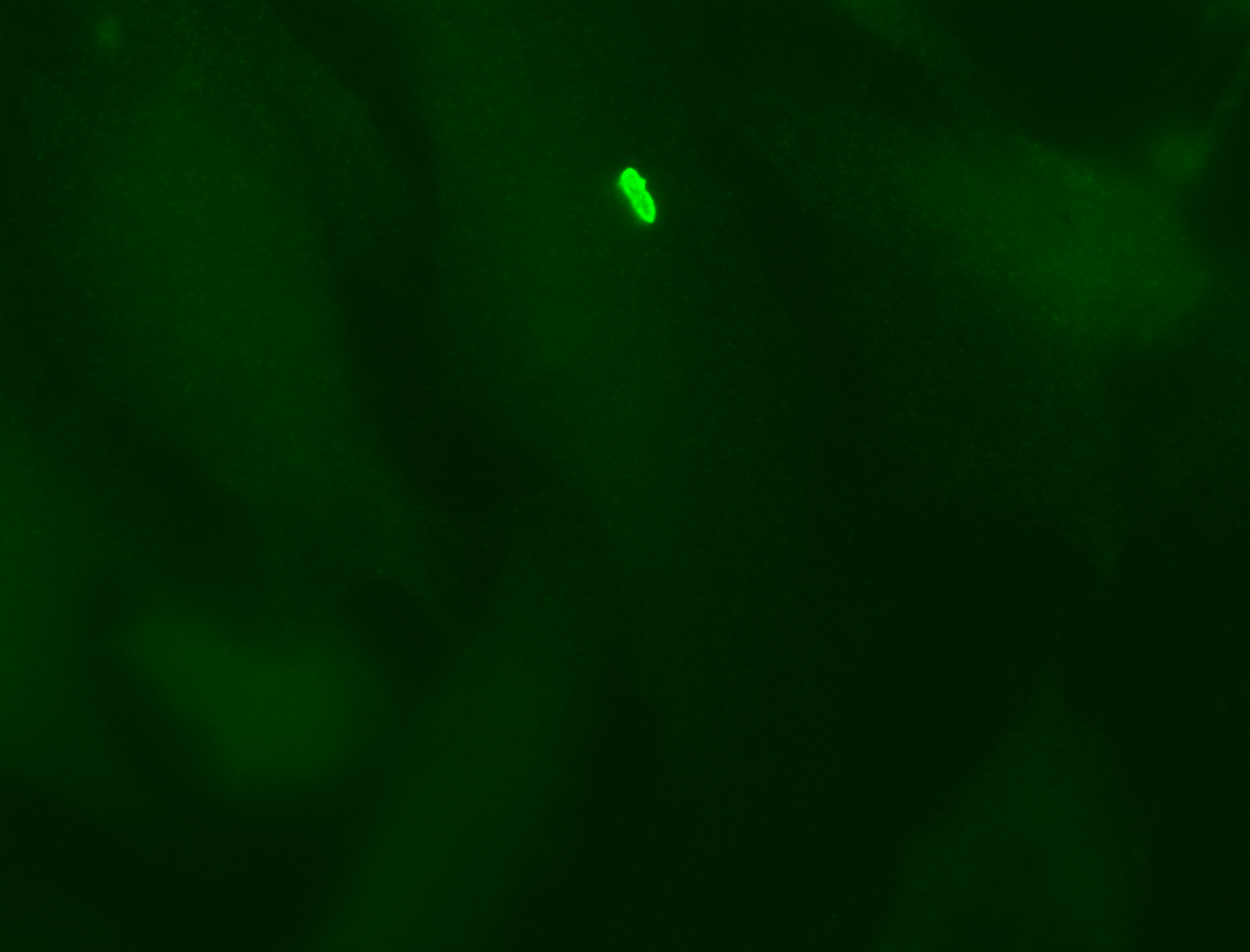

Supplement: Figure 4—source data 5. [file elife-89002-fig4-data5.zip › p3xflag-sdcb c57a del sidcs 9-c1.tif]

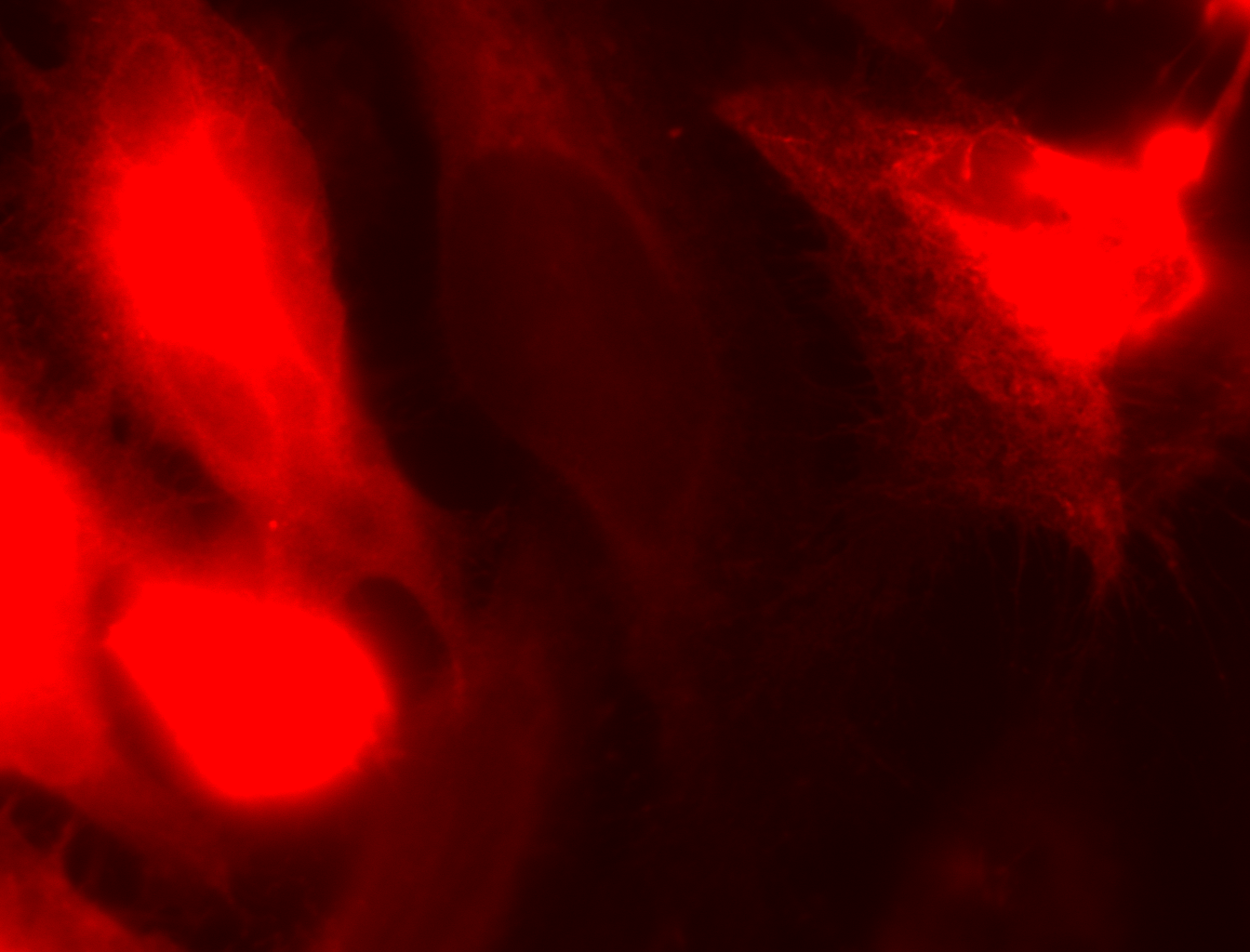

Supplement: Figure 4—source data 5. [file elife-89002-fig4-data5.zip › p3xflag-sdcb c57a del sidcs 9-c2.tif]

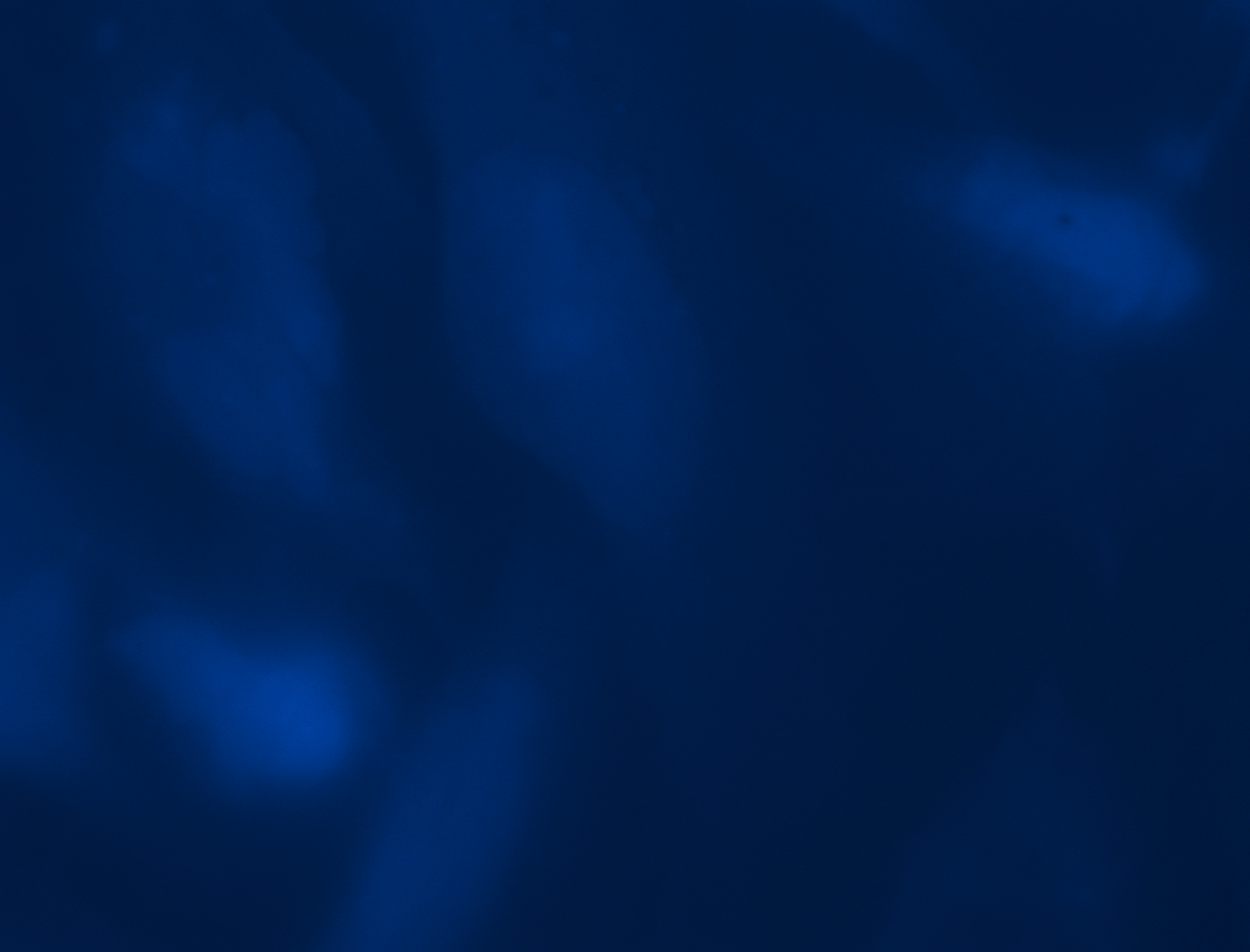

Supplement: Figure 4—source data 5. [file elife-89002-fig4-data5.zip › p3xflag-sdcb c57a del sidcs 9-c3.tif]

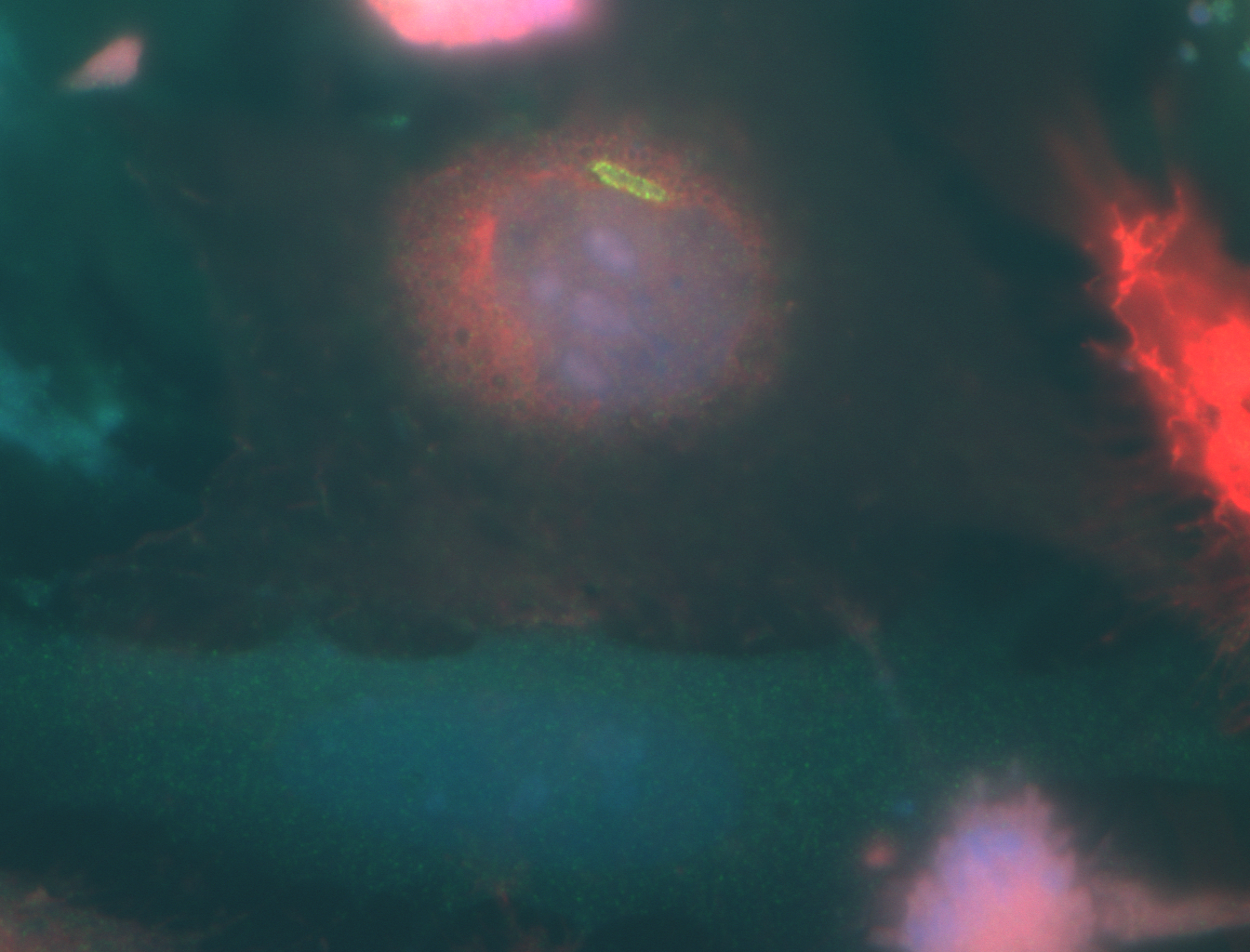

Supplement: Figure 4—source data 5. [file elife-89002-fig4-data5.zip › p3xFLAG-sdcB del sidCs 11+.tif]

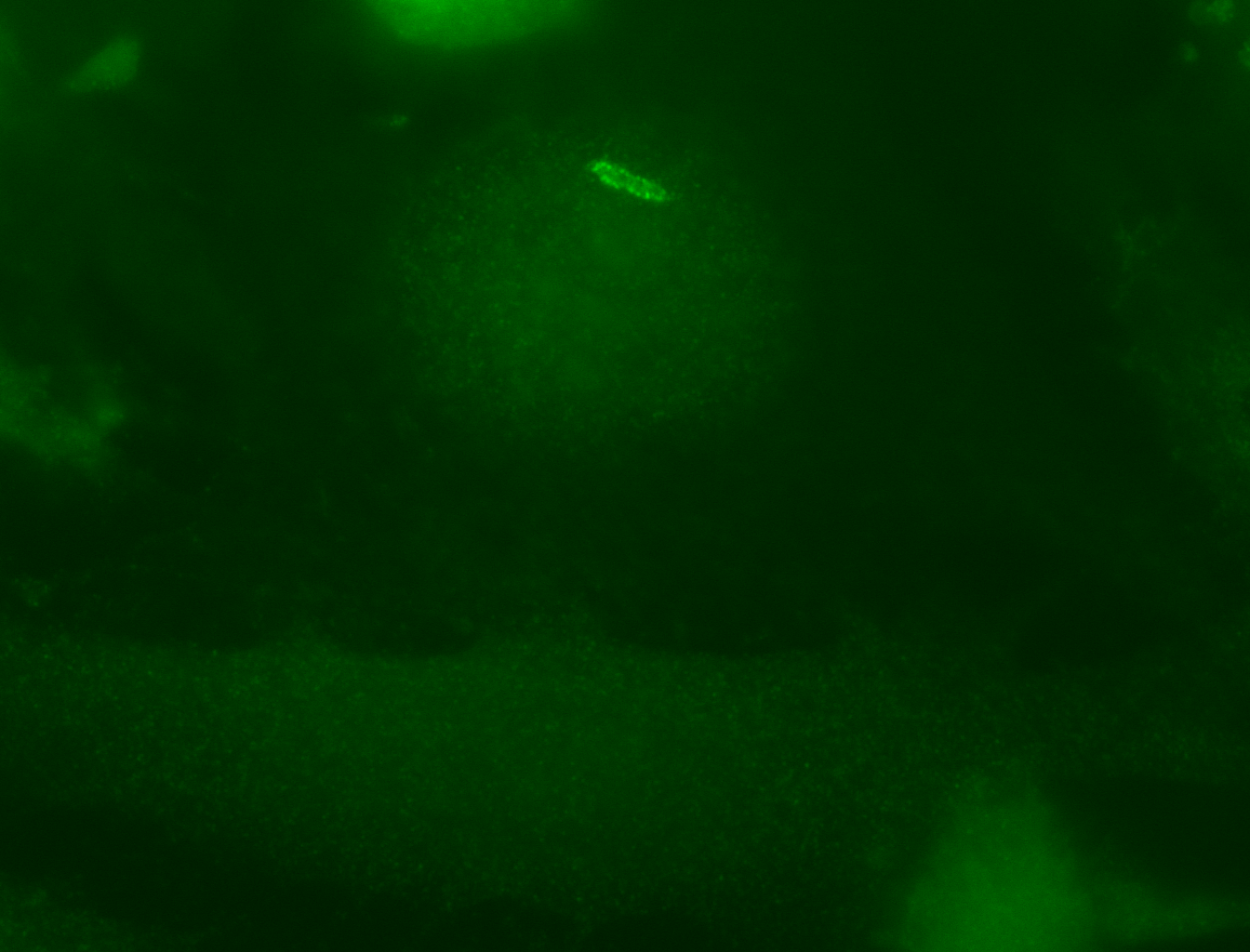

Supplement: Figure 4—source data 5. [file elife-89002-fig4-data5.zip › p3xflag-sdcb del sidcs 11+c1.tif]

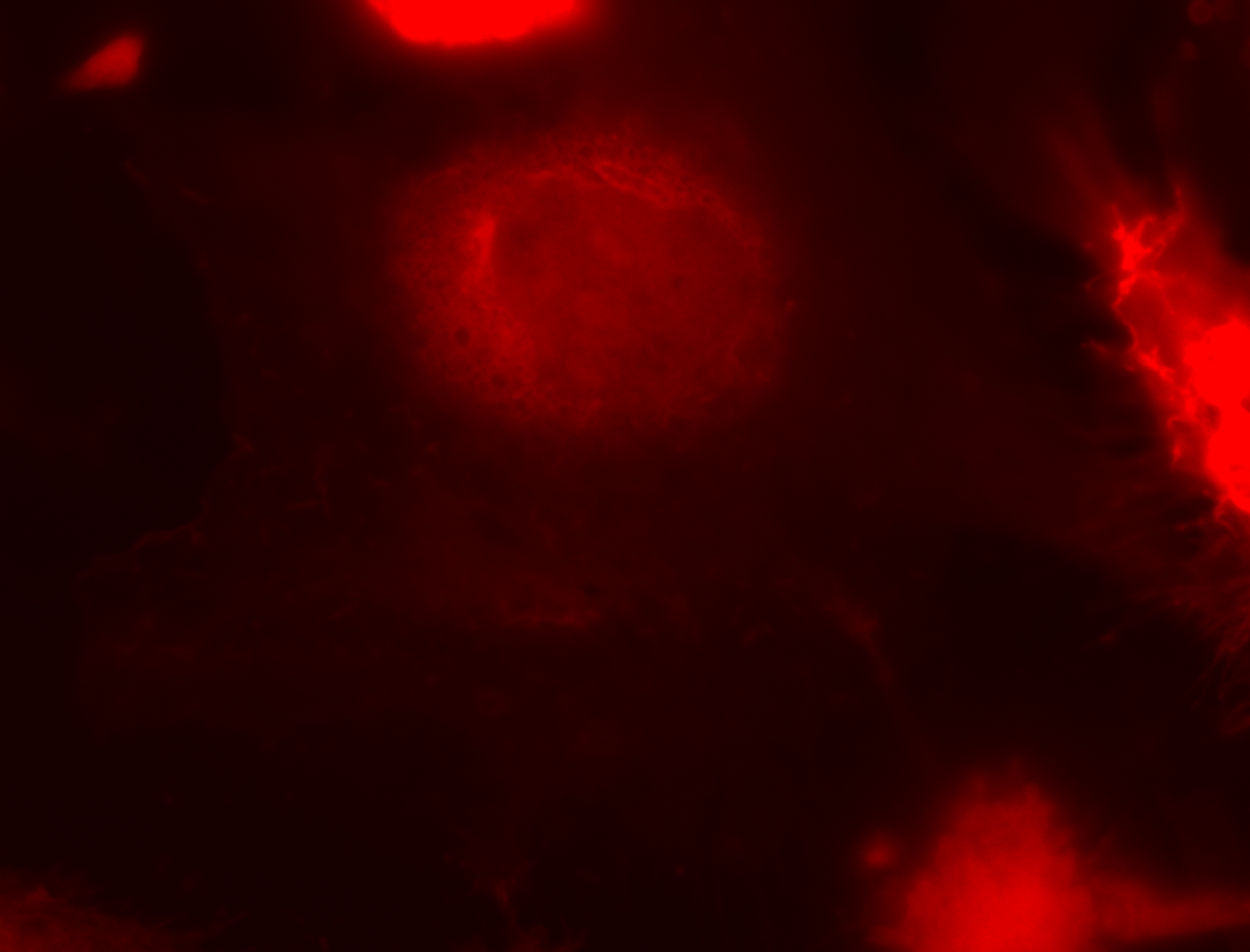

Supplement: Figure 4—source data 5. [file elife-89002-fig4-data5.zip › p3xflag-sdcb del sidcs 11+c2.tif]

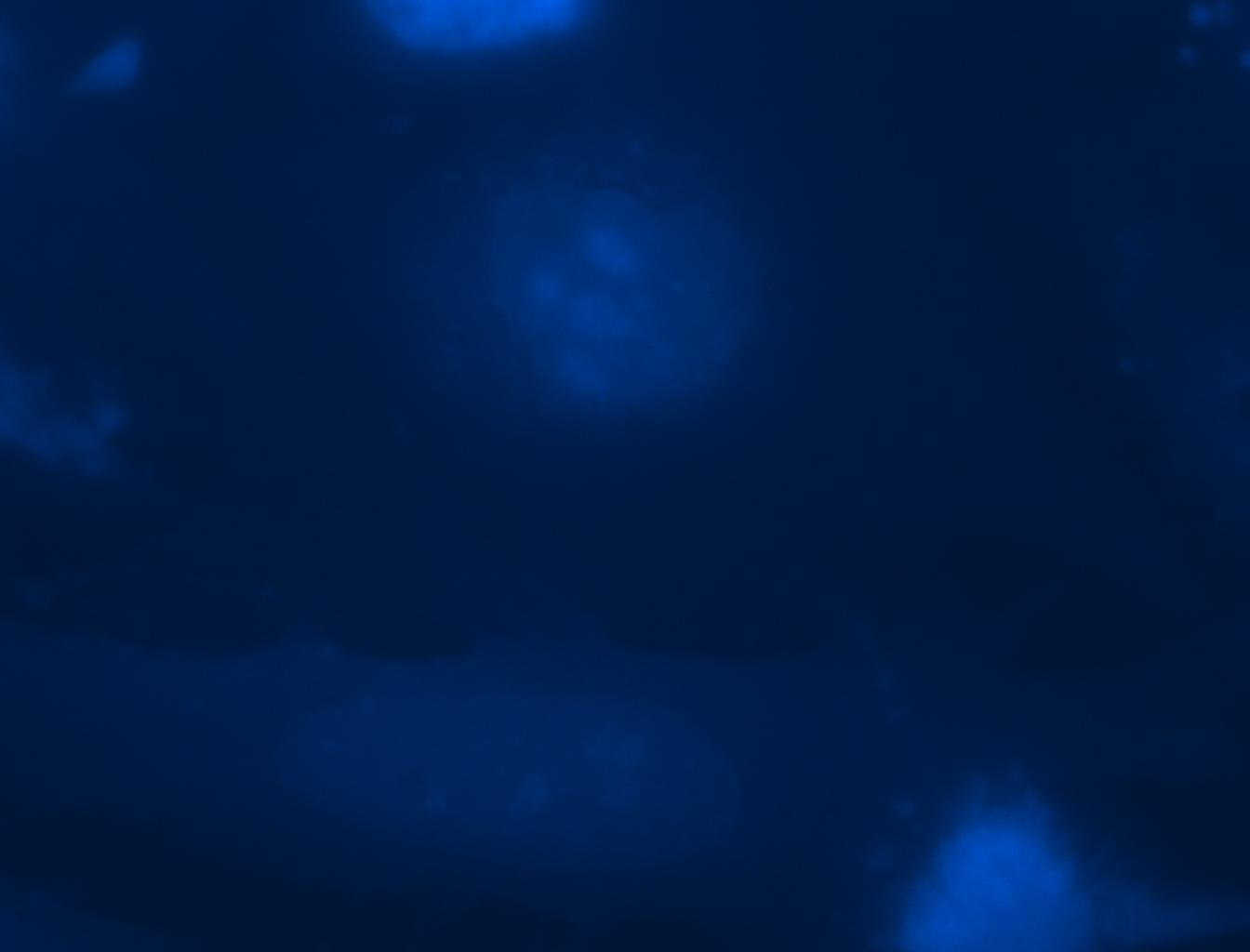

Supplement: Figure 4—source data 5. [file elife-89002-fig4-data5.zip › p3xflag-sdcb del sidcs 11+c3.tif]

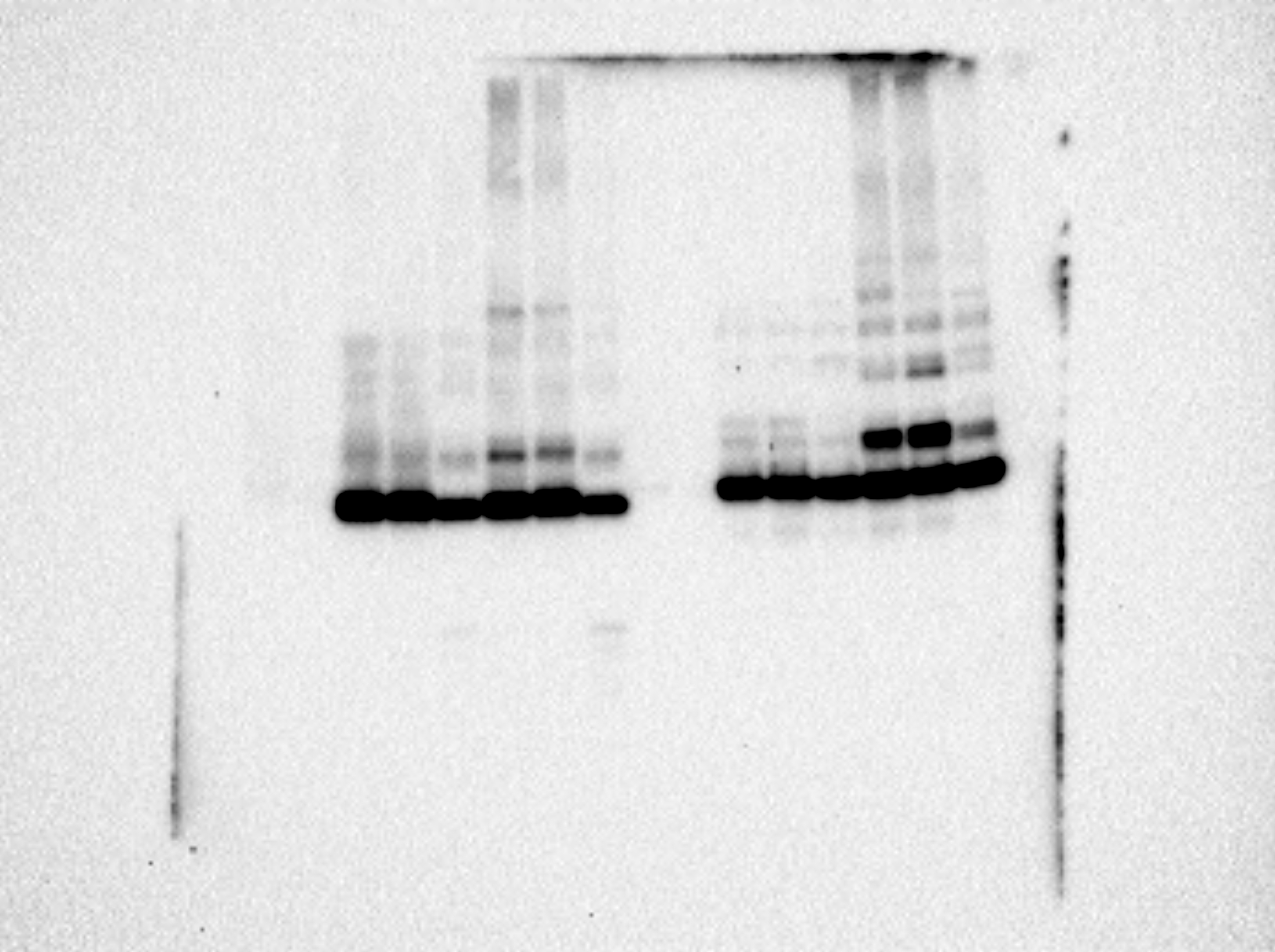

Supplement: Figure 4—figure supplement 1—source data 1. [file elife-89002-fig4-figsupp1-data1.zip › IP FLAG anti-FLAGm_Exposure_44.5sec.jpg]

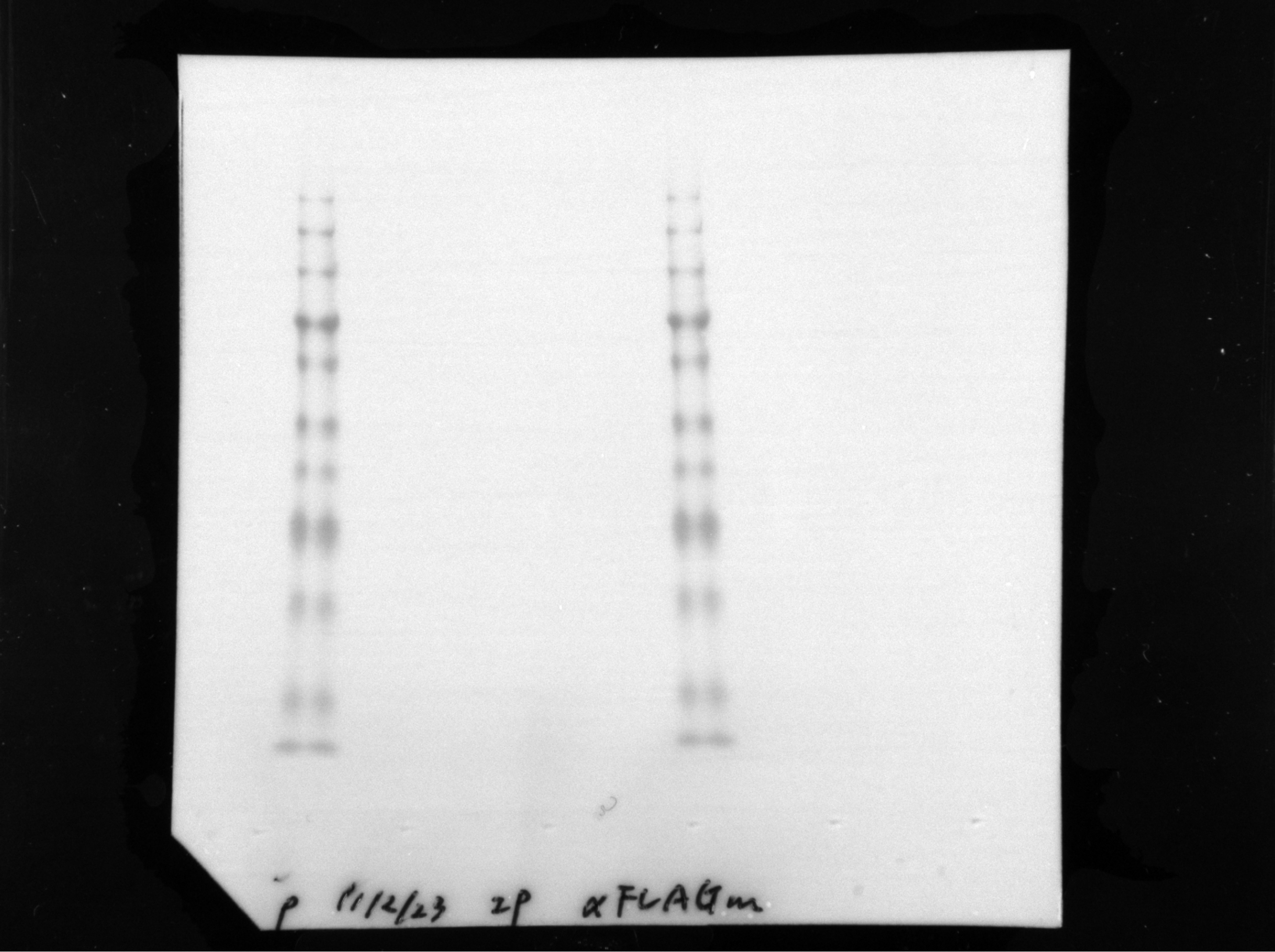

Supplement: Figure 4—figure supplement 1—source data 1. [file elife-89002-fig4-figsupp1-data1.zip › IP FLAG anti-FLAGm_Marker.jpg]

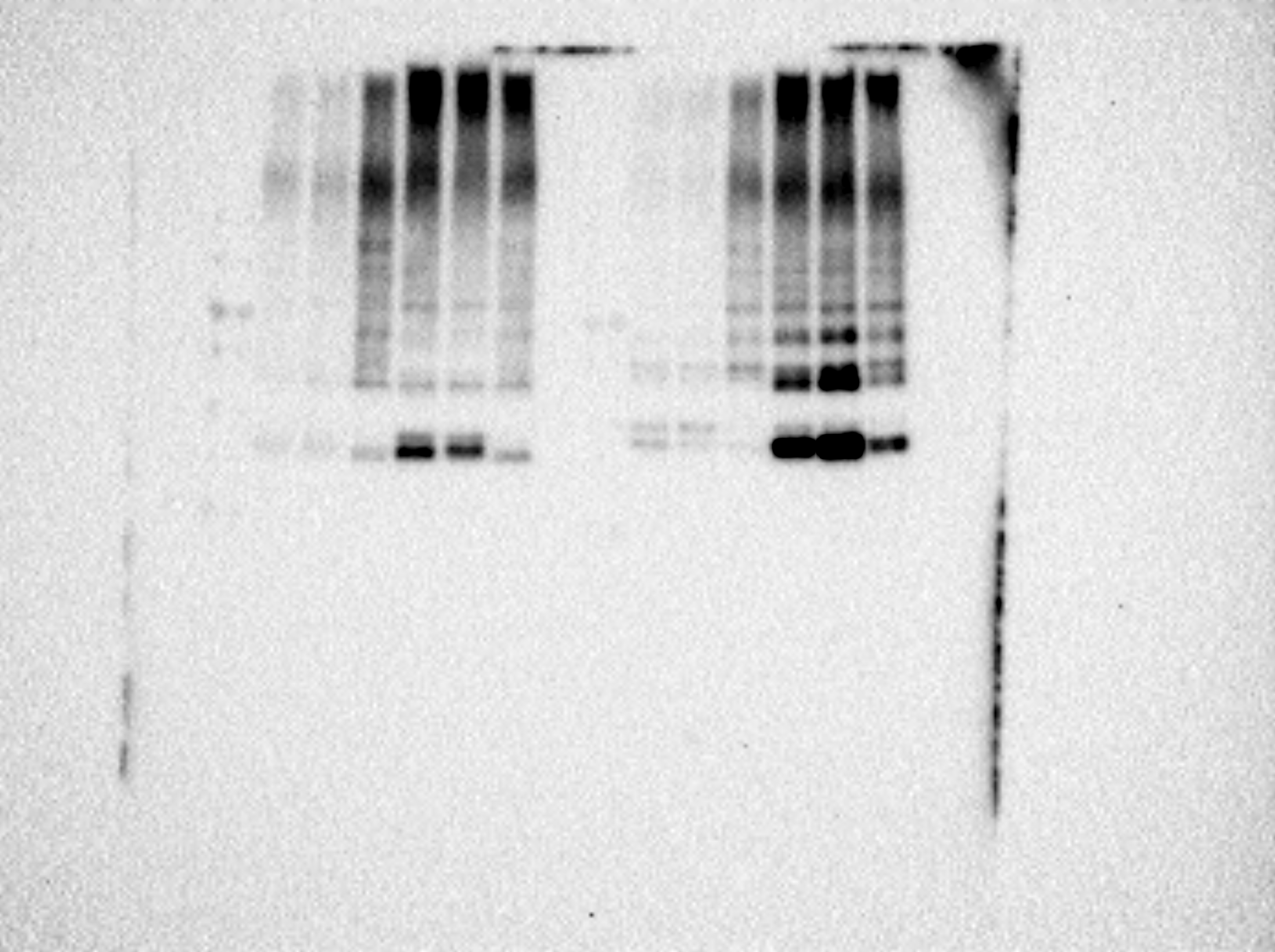

Supplement: Figure 4—figure supplement 1—source data 1. [file elife-89002-fig4-figsupp1-data1.zip › IP FLAG anti-HArb_Exposure_60.0sec.jpg]

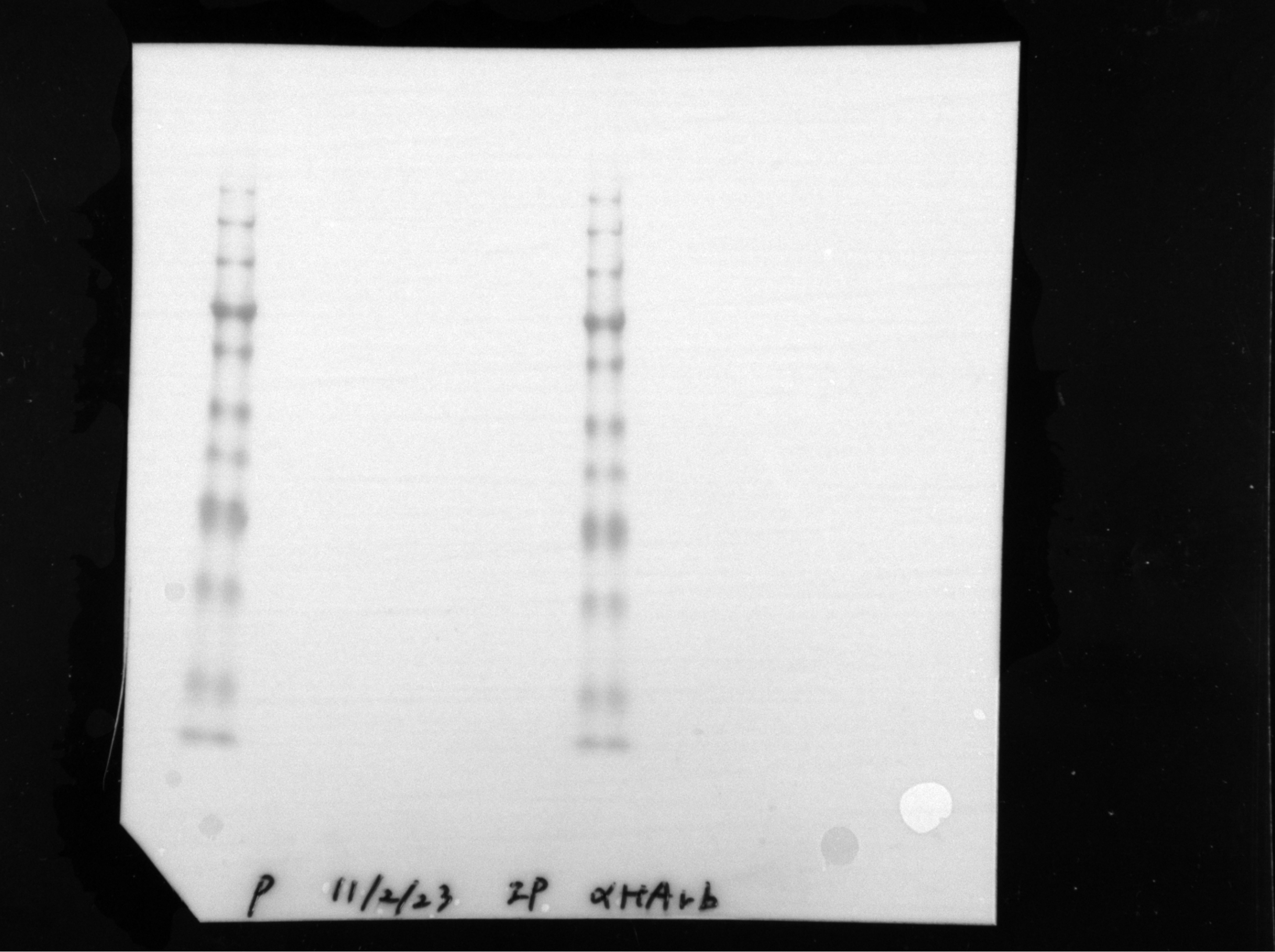

Supplement: Figure 4—figure supplement 1—source data 1. [file elife-89002-fig4-figsupp1-data1.zip › IP FLAG anti-HArb_Marker.jpg]

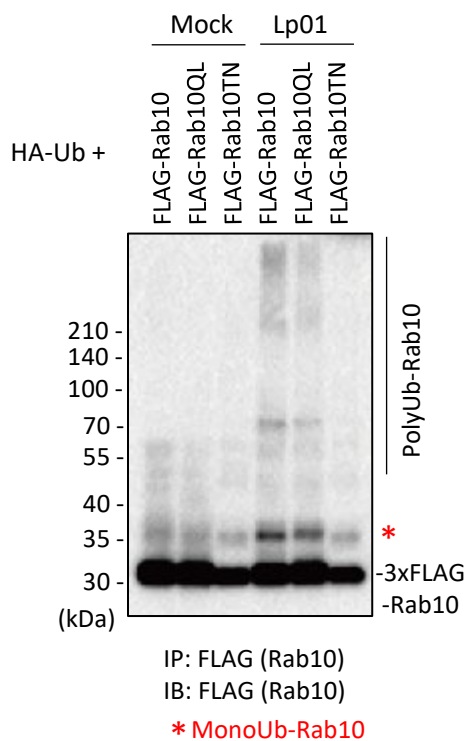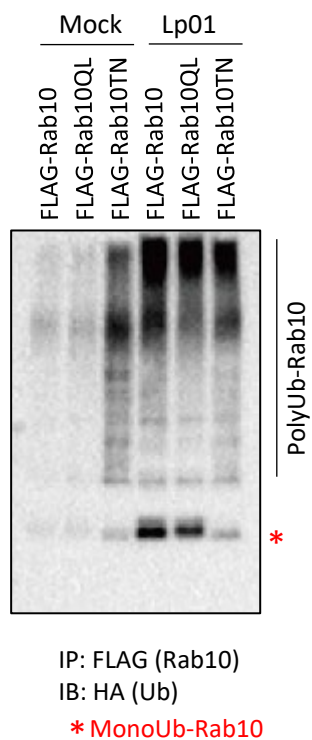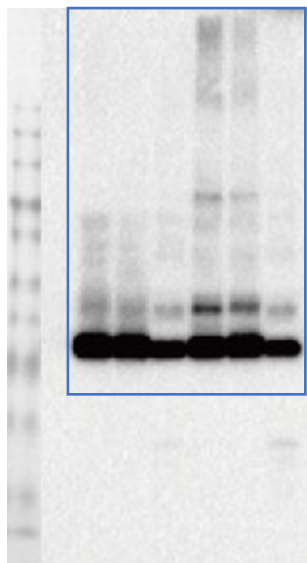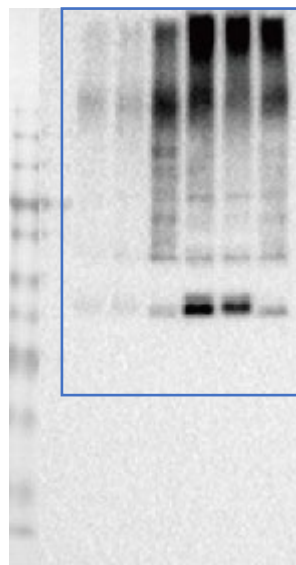

Figure 4 – figure supplement 1  
left

Figure 4 – figure supplement 1  
right

Supplement: Figure 4—figure supplement 1—source data 2. [file elife-89002-fig4-figsupp1-data2.pdf]

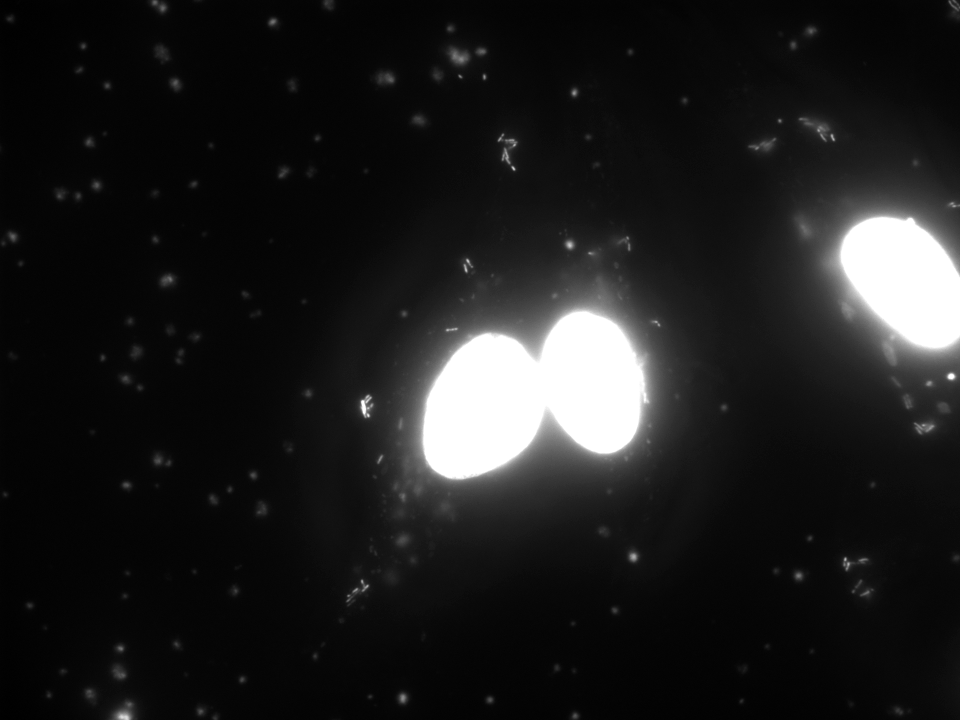

Supplement: Figure 4—figure supplement 2—source data 1. [file elife-89002-fig4-figsupp2-data1.zip › dotA_blue.tif]

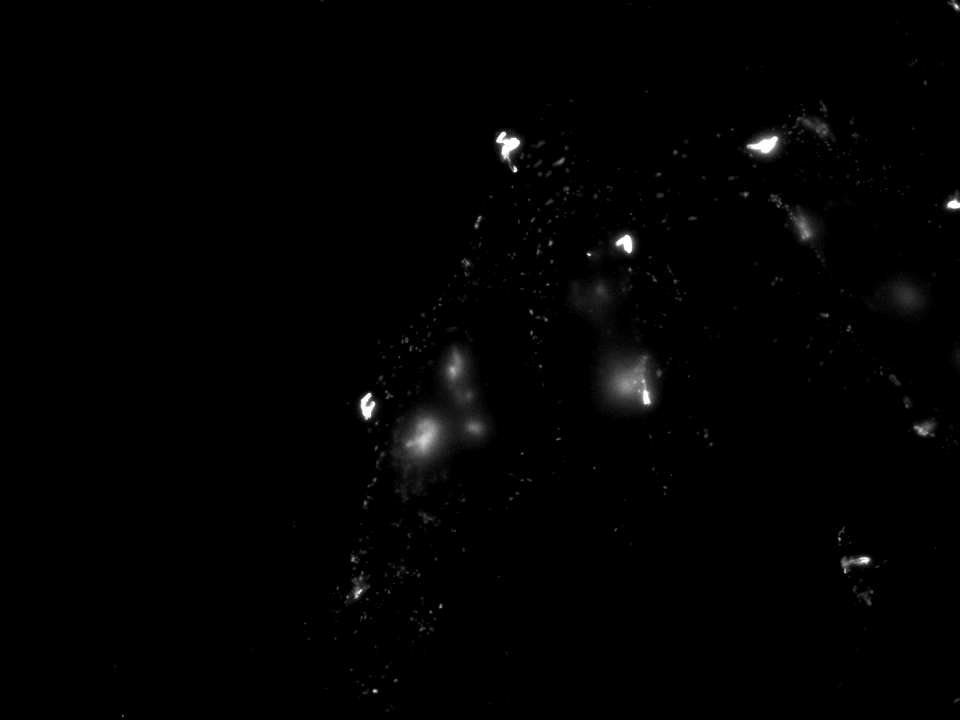

Supplement: Figure 4—figure supplement 2—source data 1. [file elife-89002-fig4-figsupp2-data1.zip › dotA_green.tif]

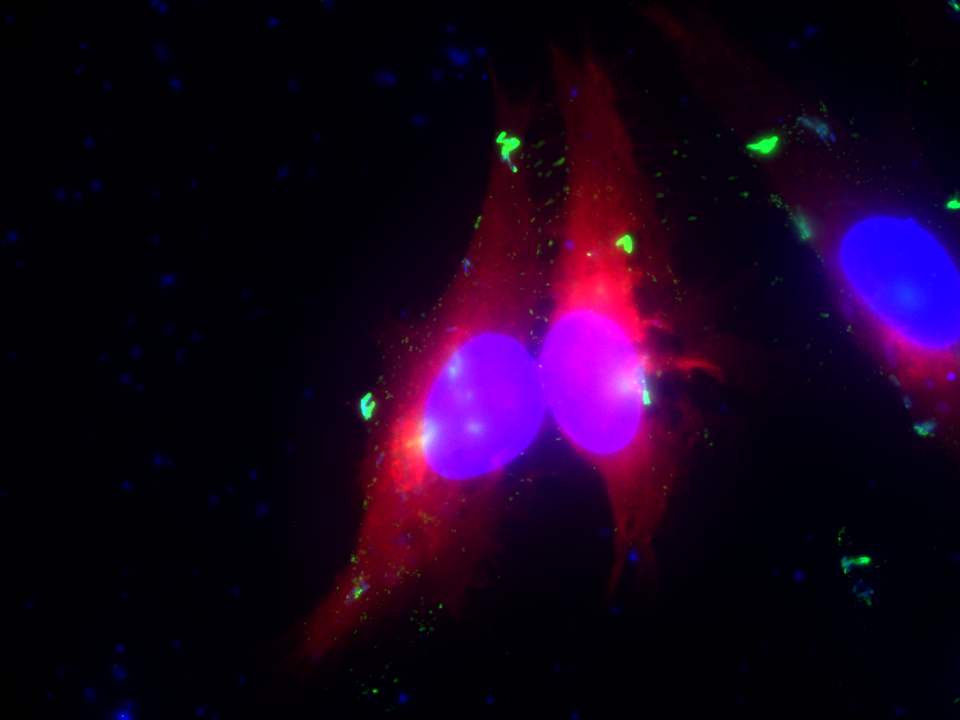

Supplement: Figure 4—figure supplement 2—source data 1. [file elife-89002-fig4-figsupp2-data1.zip › dotA_merge.tif]

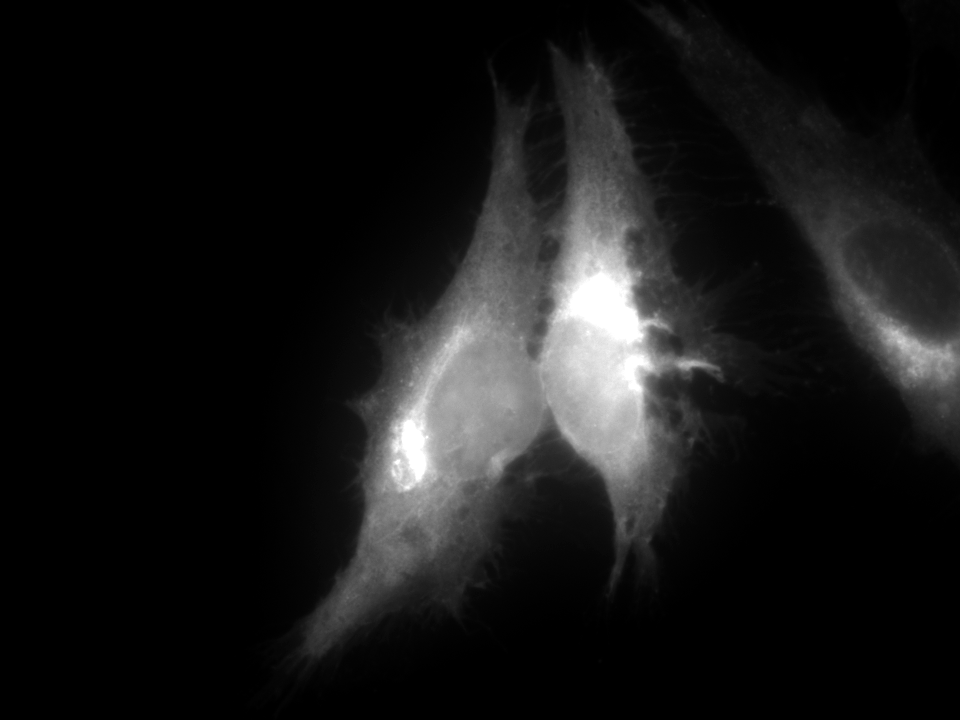

Supplement: Figure 4—figure supplement 2—source data 1. [file elife-89002-fig4-figsupp2-data1.zip › dotA_red.tif]

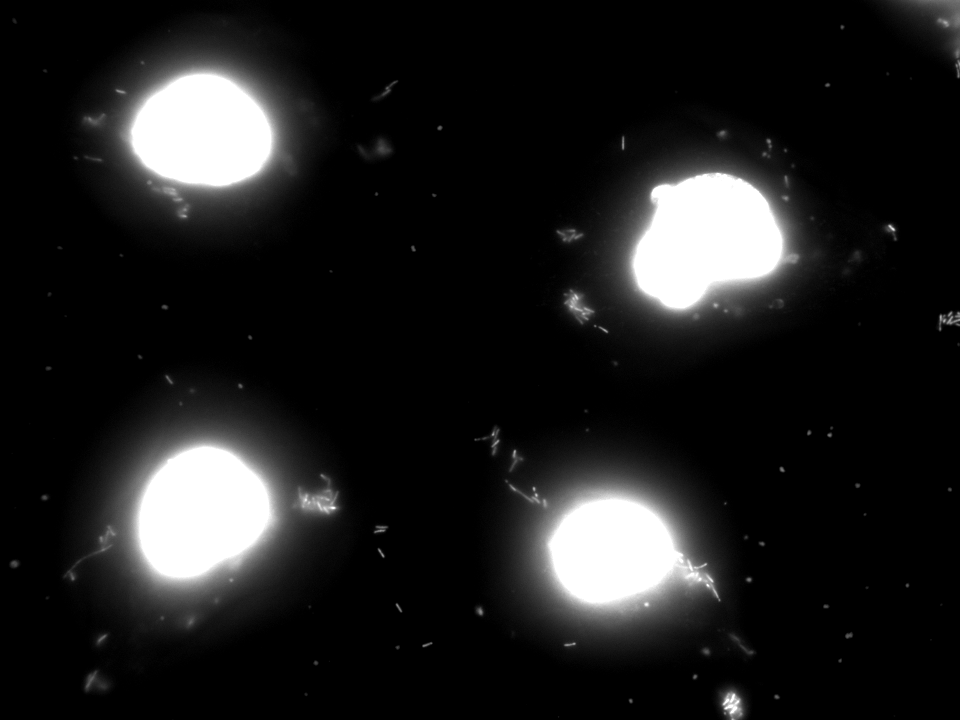

Supplement: Figure 4—figure supplement 2—source data 1. [file elife-89002-fig4-figsupp2-data1.zip › Lp01_blue.tif]

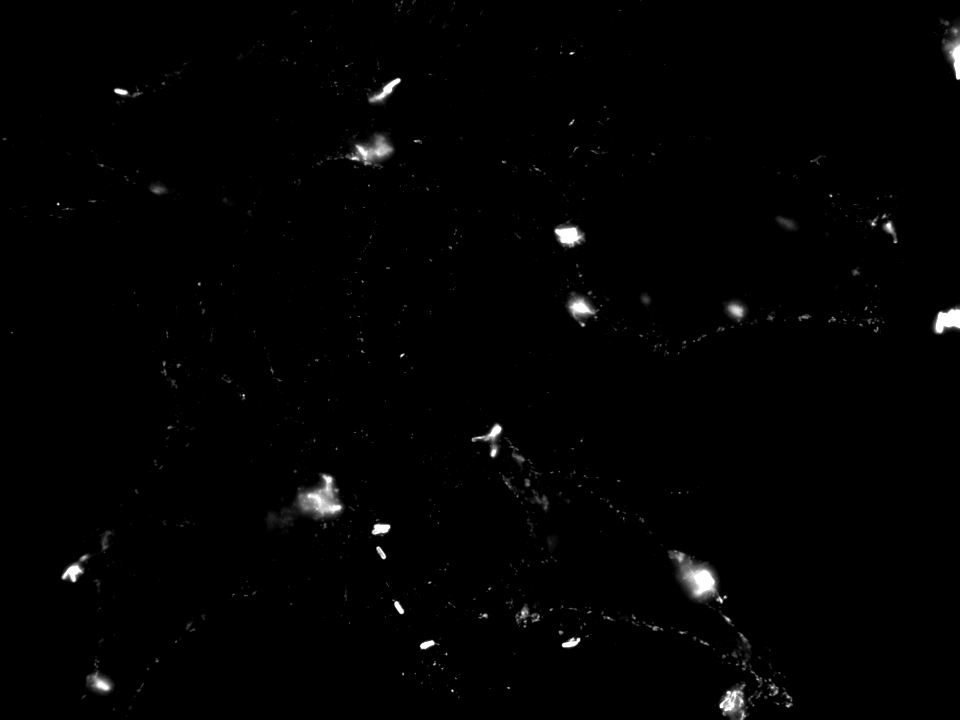

Supplement: Figure 4—figure supplement 2—source data 1. [file elife-89002-fig4-figsupp2-data1.zip › Lp01_green.tif]

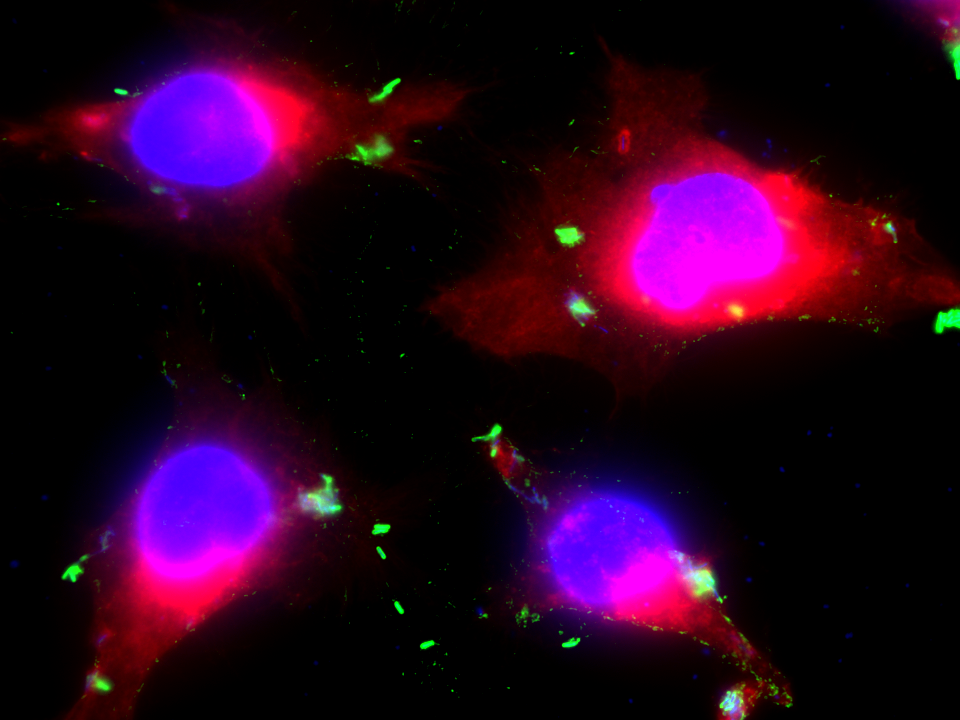

Supplement: Figure 4—figure supplement 2—source data 1. [file elife-89002-fig4-figsupp2-data1.zip › Lp01_merge.tif]

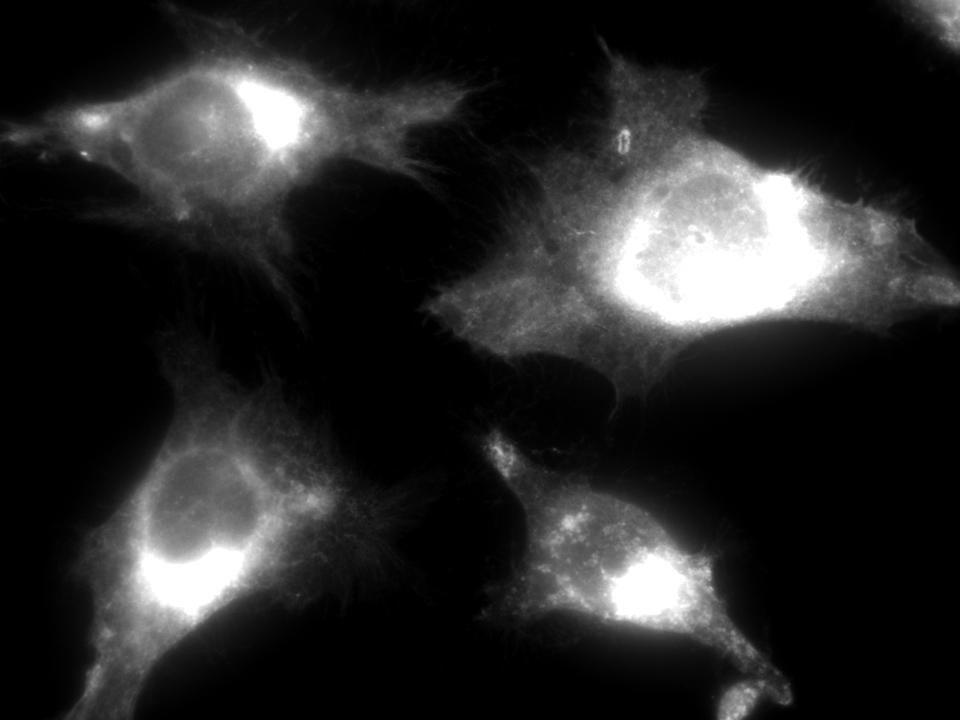

Supplement: Figure 4—figure supplement 2—source data 1. [file elife-89002-fig4-figsupp2-data1.zip › Lp01_red.tif]

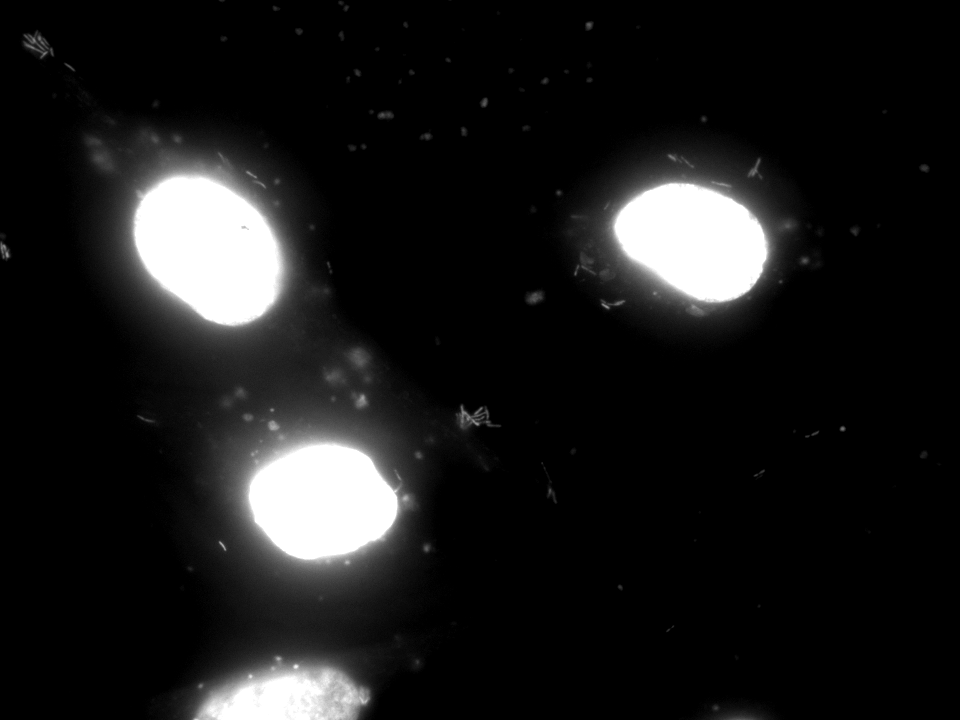

Supplement: Figure 4—figure supplement 2—source data 1. [file elife-89002-fig4-figsupp2-data1.zip › sidCsdcAsdcB_blue.tif]

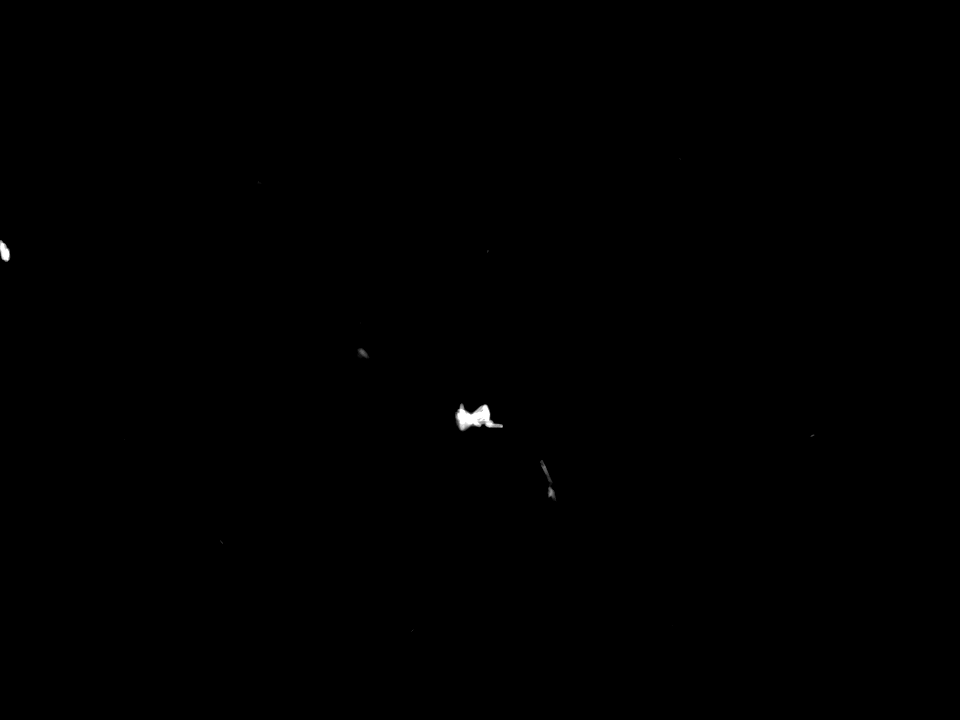

Supplement: Figure 4—figure supplement 2—source data 1. [file elife-89002-fig4-figsupp2-data1.zip › sidCsdcAsdcB_green.tif]

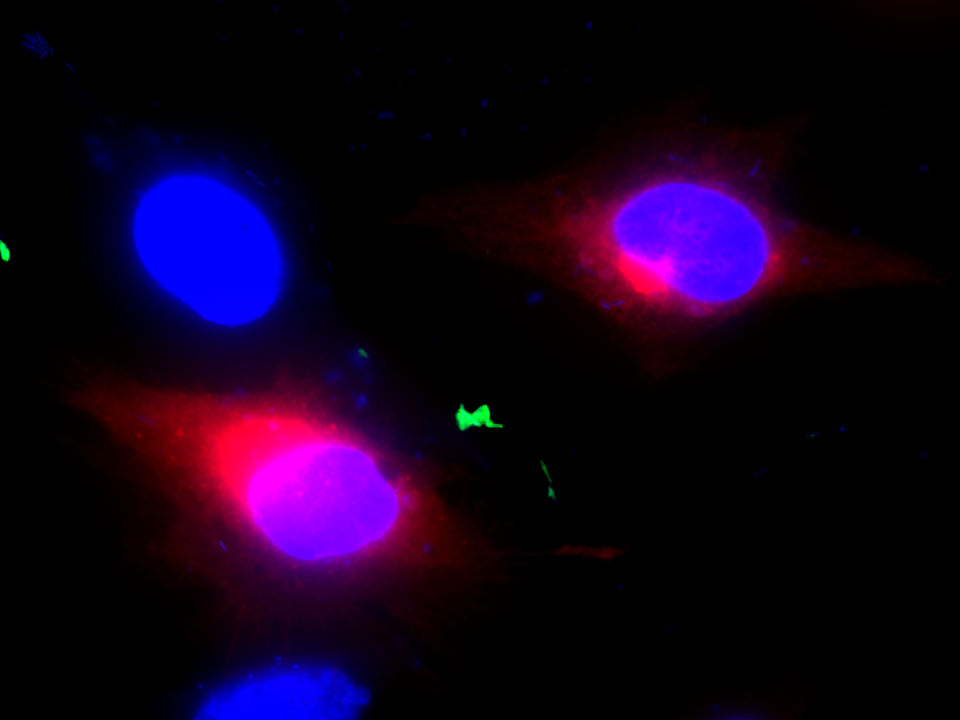

Supplement: Figure 4—figure supplement 2—source data 1. [file elife-89002-fig4-figsupp2-data1.zip › sidCsdcAsdcB_merge.tif]

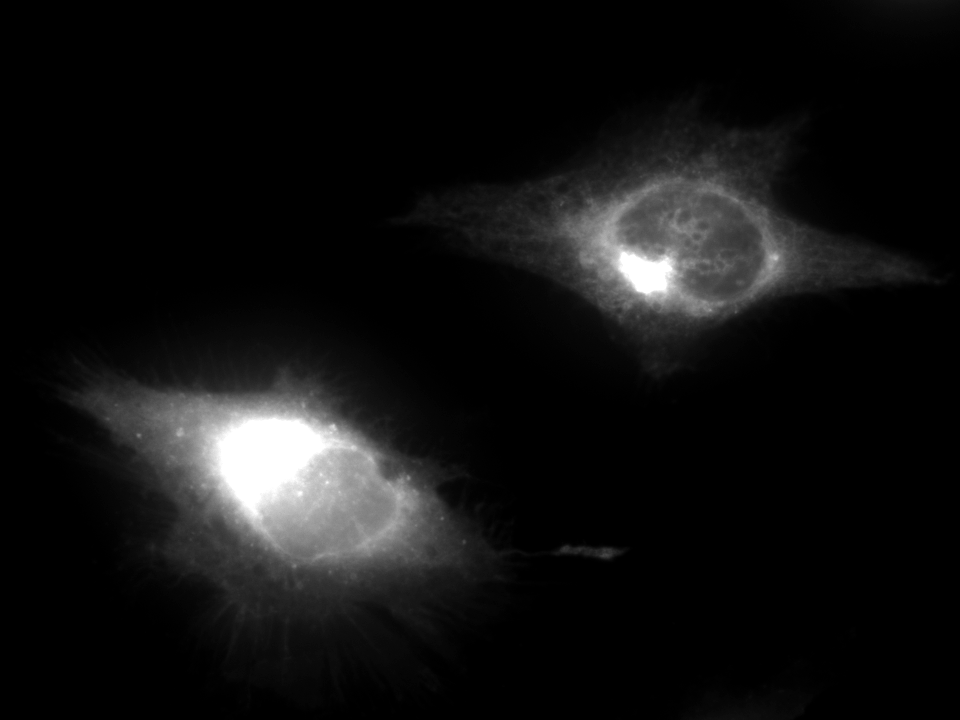

Supplement: Figure 4—figure supplement 2—source data 1. [file elife-89002-fig4-figsupp2-data1.zip › sidCsdcAsdcB_red.tif]

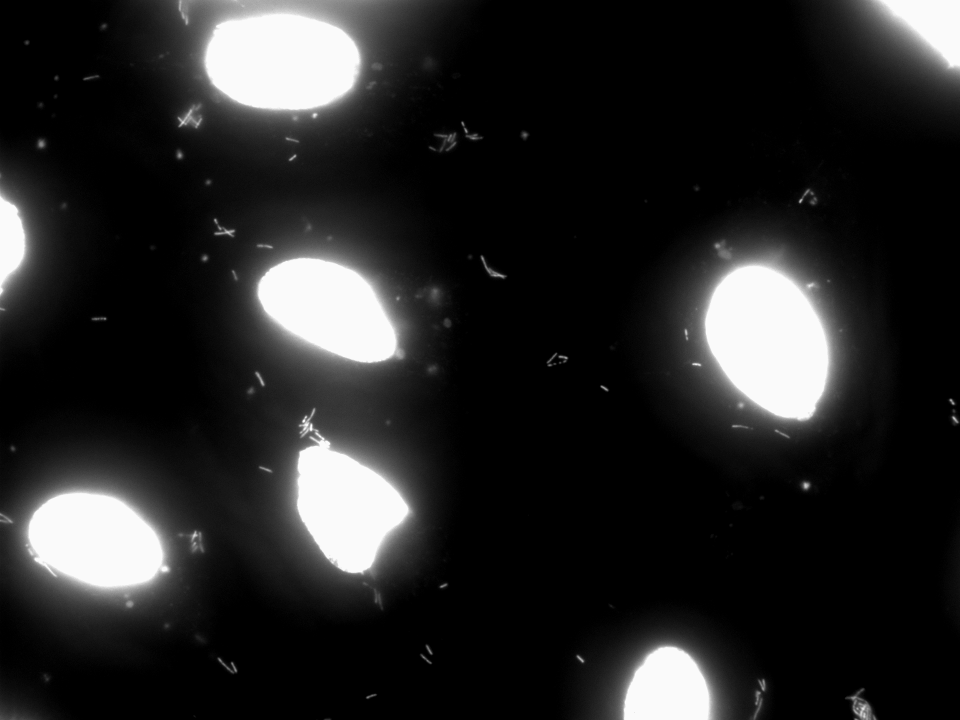

Supplement: Figure 4—figure supplement 2—source data 1. [file elife-89002-fig4-figsupp2-data1.zip › sidEs_blue.tif]

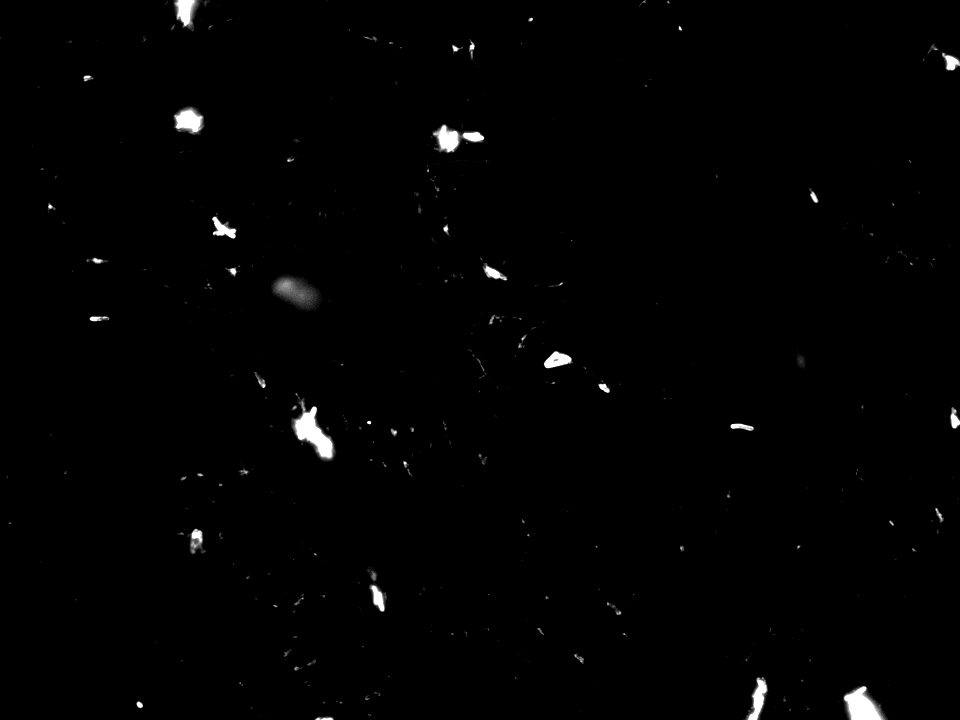

Supplement: Figure 4—figure supplement 2—source data 1. [file elife-89002-fig4-figsupp2-data1.zip › sidEs_green.tif]

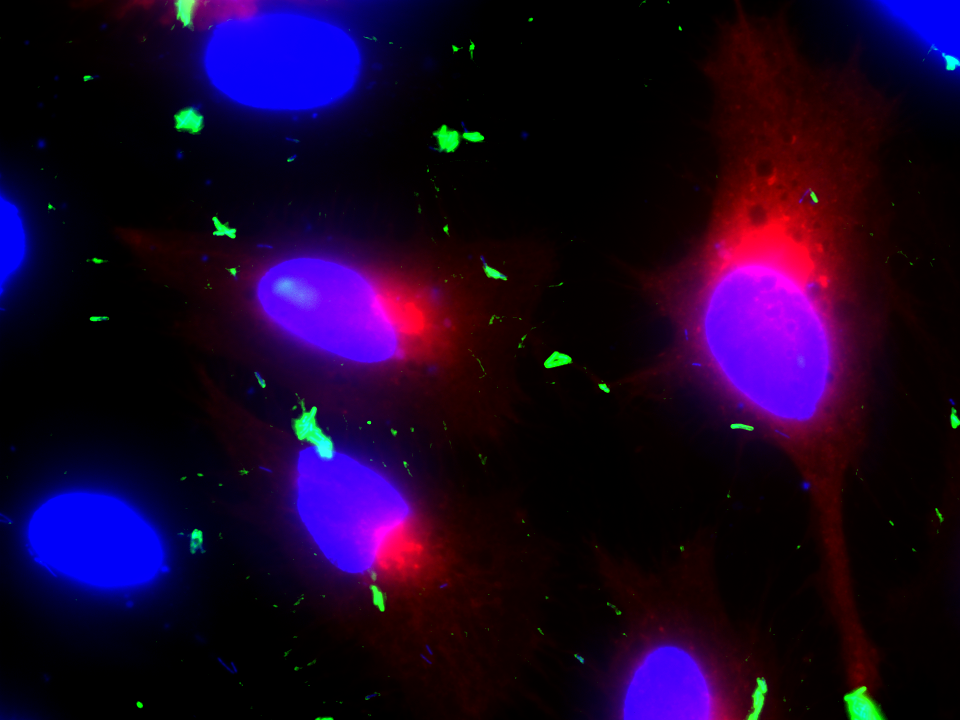

Supplement: Figure 4—figure supplement 2—source data 1. [file elife-89002-fig4-figsupp2-data1.zip › sidEs_merge.tif]

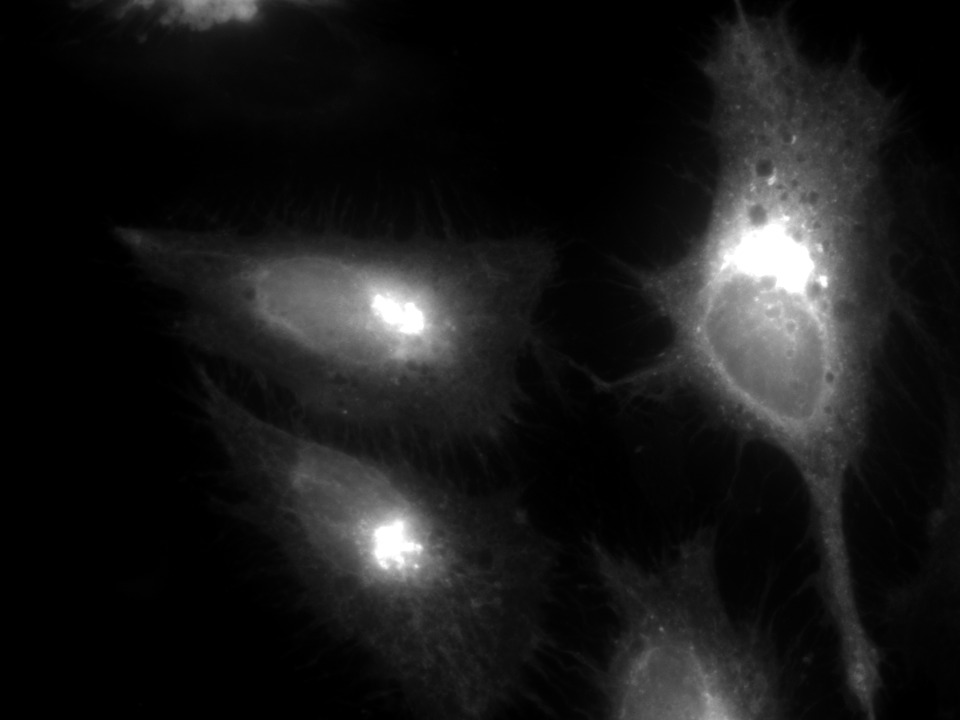

Supplement: Figure 4—figure supplement 2—source data 1. [file elife-89002-fig4-figsupp2-data1.zip › sidEs_red.tif]

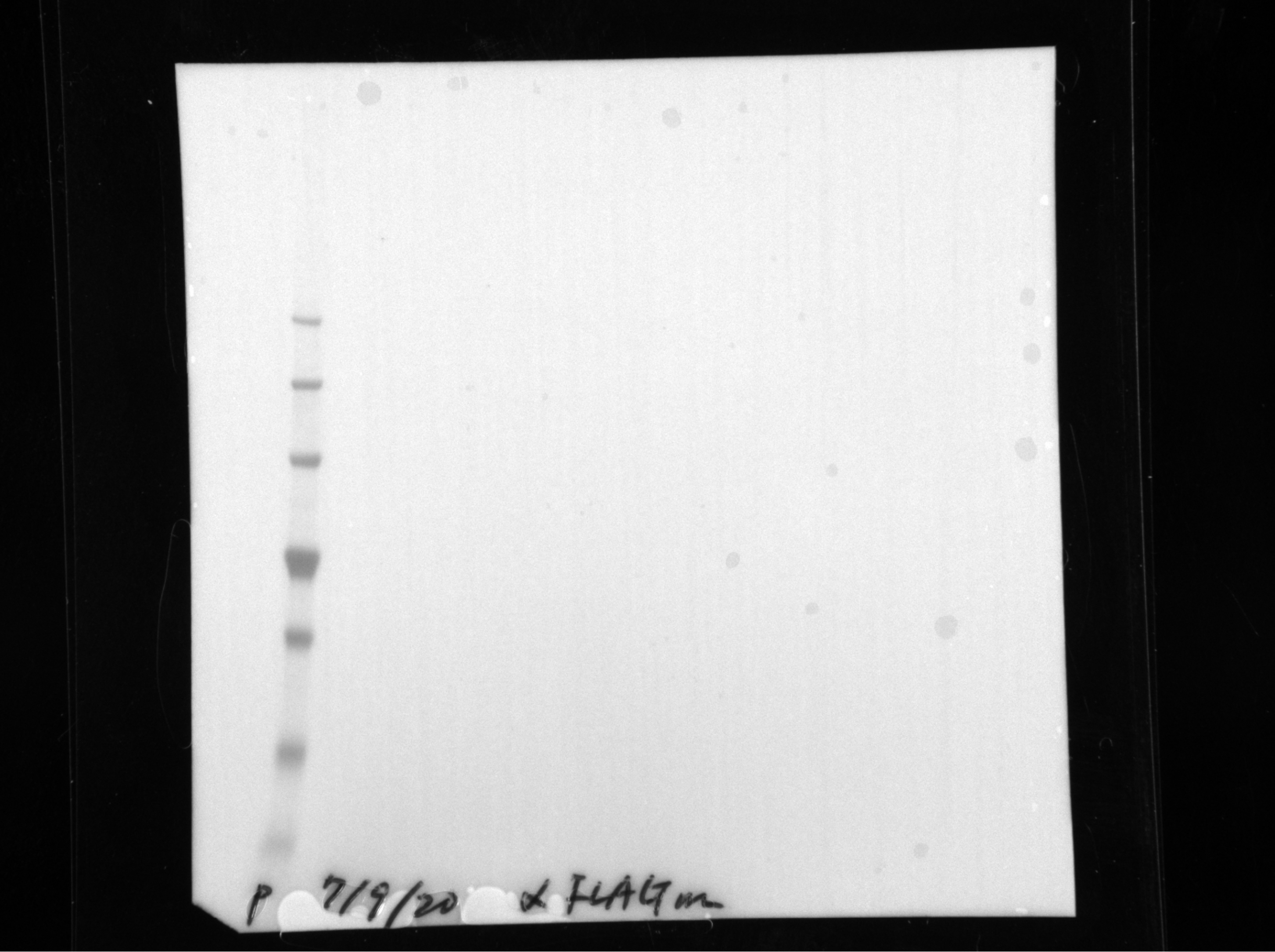

Supplement: Figure 5—source data 1. [file elife-89002-fig5-data1.zip › anti-FLAG Marker.jpg]

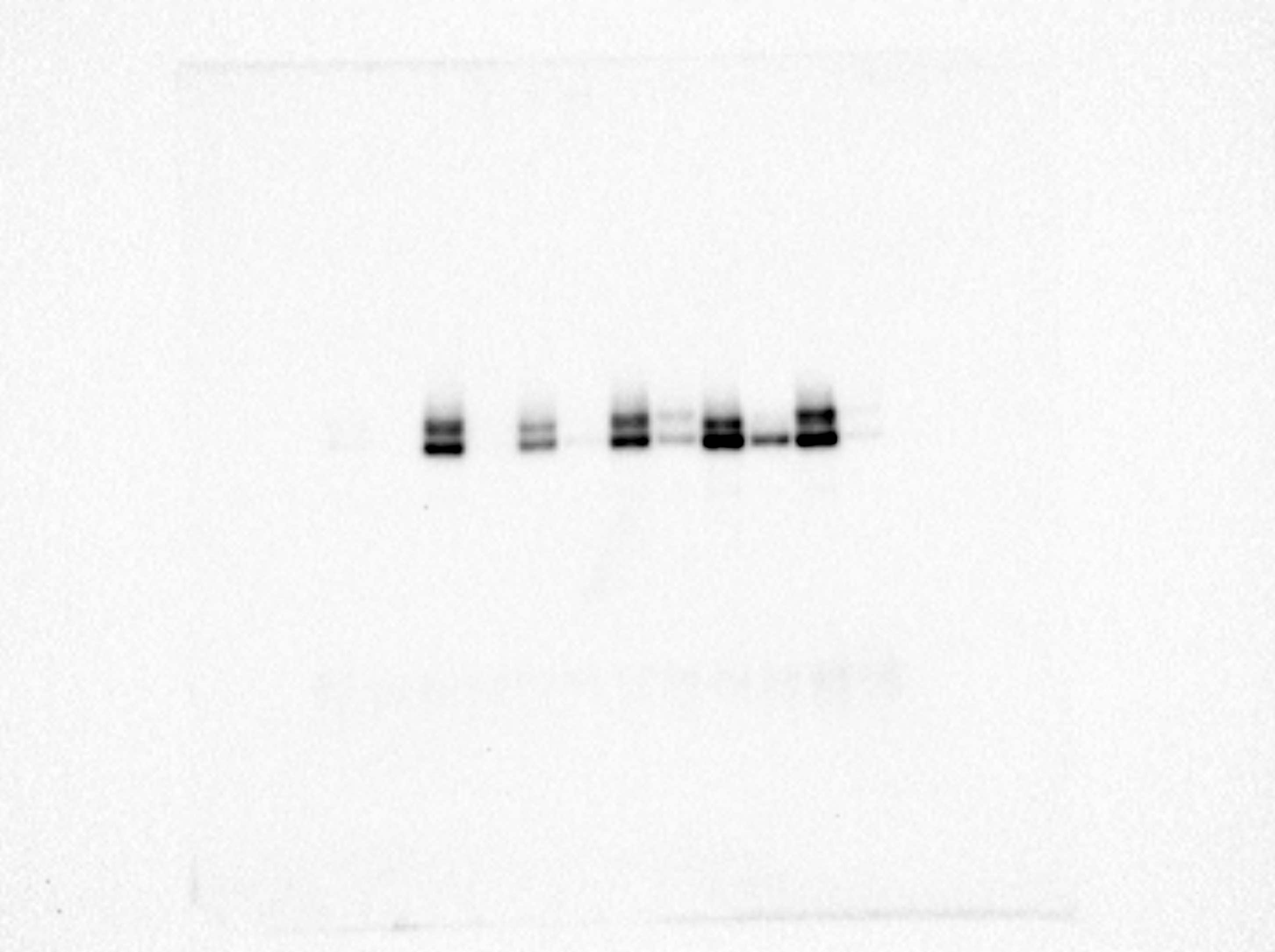

Supplement: Figure 5—source data 1. [file elife-89002-fig5-data1.zip › anti-FLAG_Exposure_60.8sec.jpg]

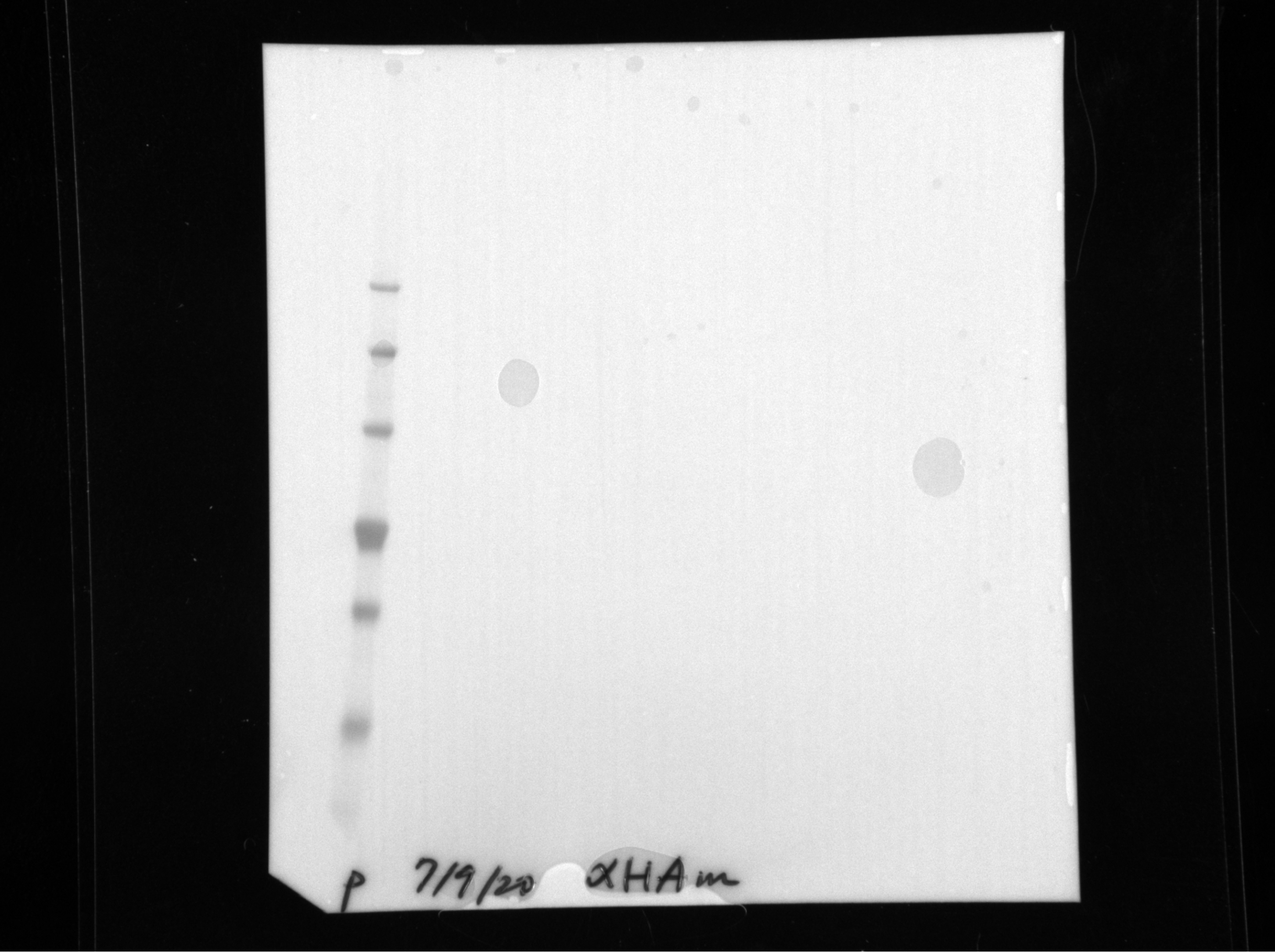

Supplement: Figure 5—source data 1. [file elife-89002-fig5-data1.zip › anti-HA pierce Marker.jpg]

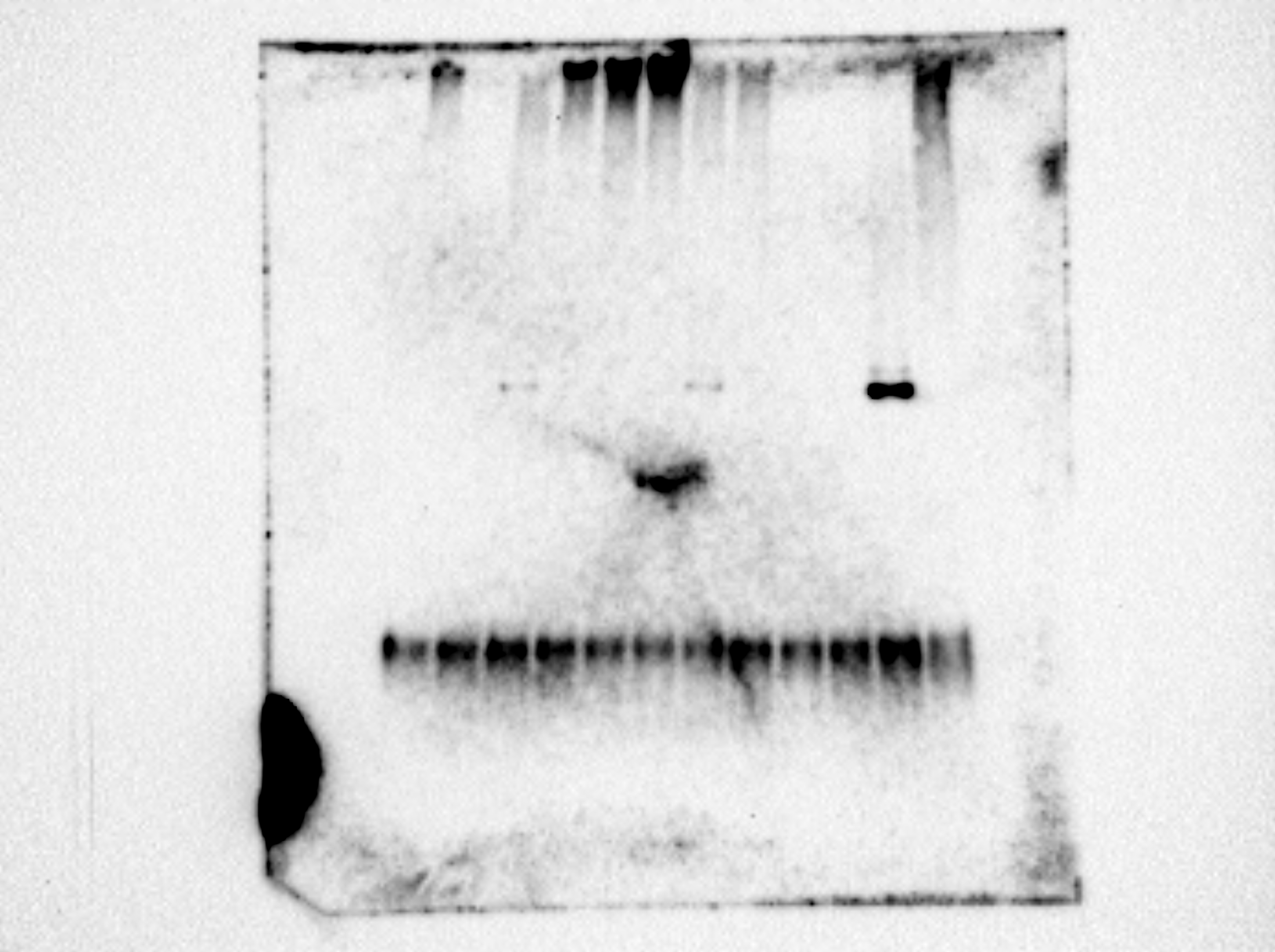

Supplement: Figure 5—source data 1. [file elife-89002-fig5-data1.zip › anti-HA pierce_Exposure_48.6sec.jpg]

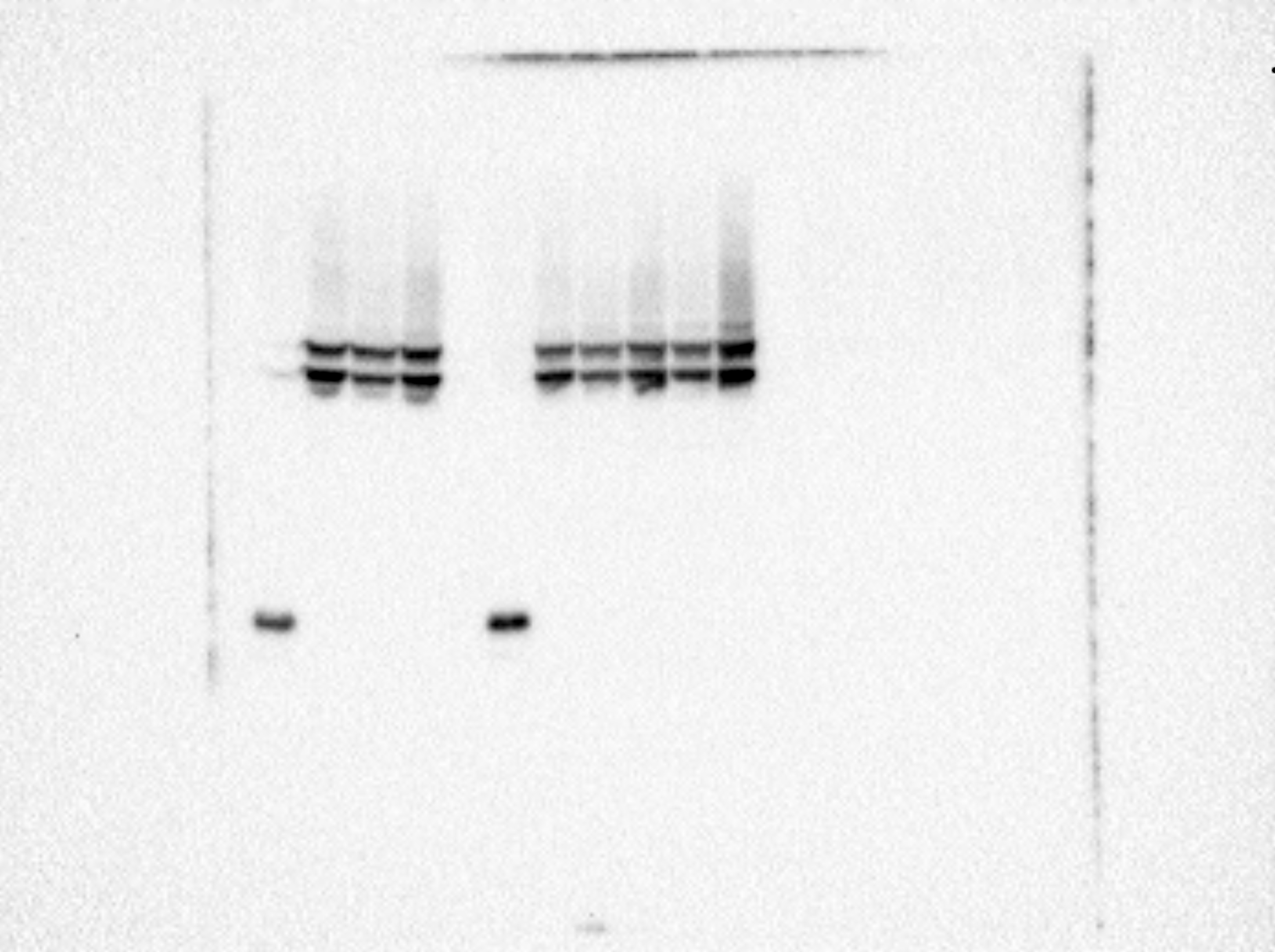

Supplement: Figure 5—source data 1. [file elife-89002-fig5-data1.zip › gel1 anti-GFPrb_Exposure_16.5sec.jpg]

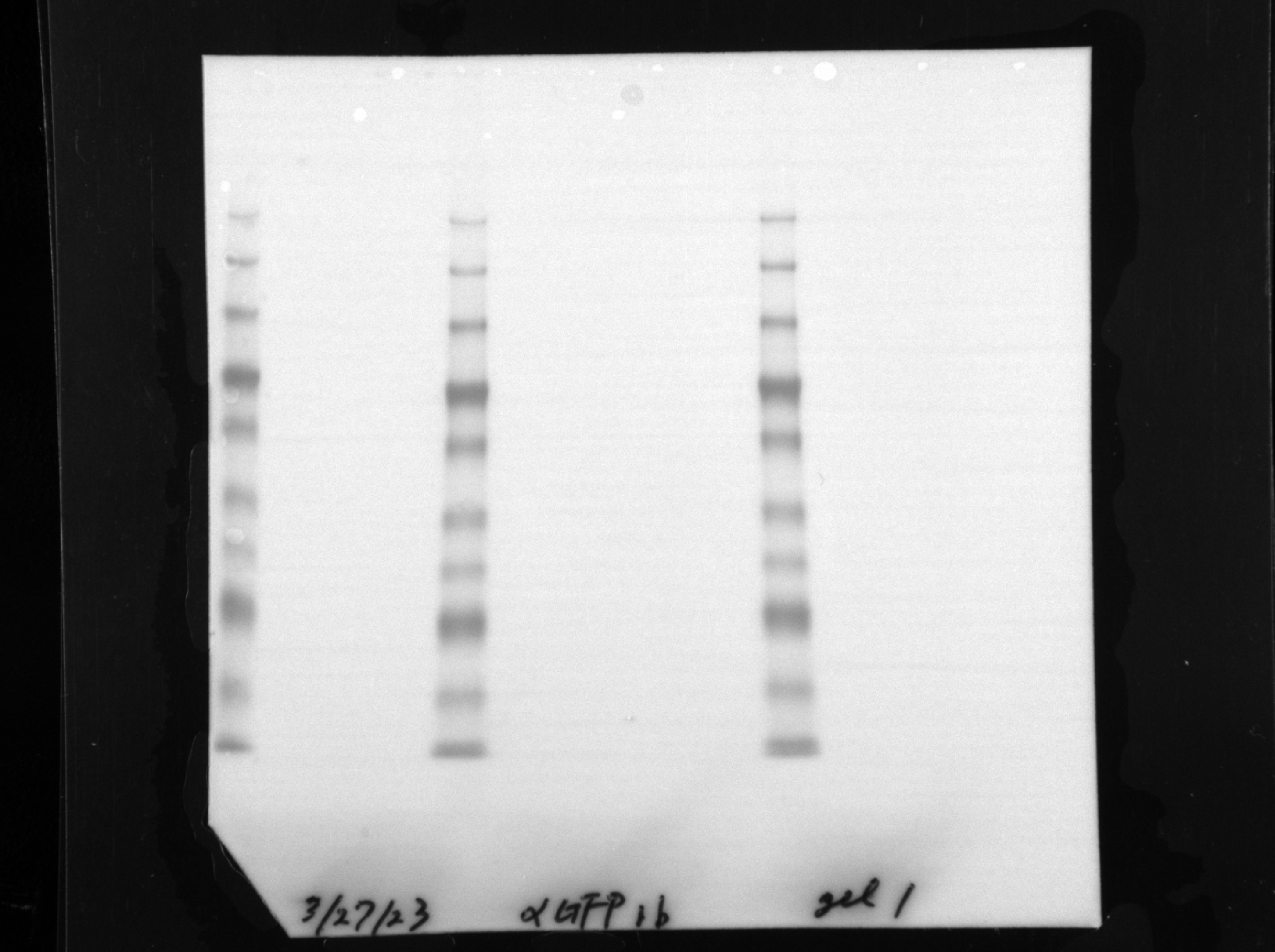

Supplement: Figure 5—source data 1. [file elife-89002-fig5-data1.zip › gel1 anti-GFPrb_Marker.jpg]

**a**

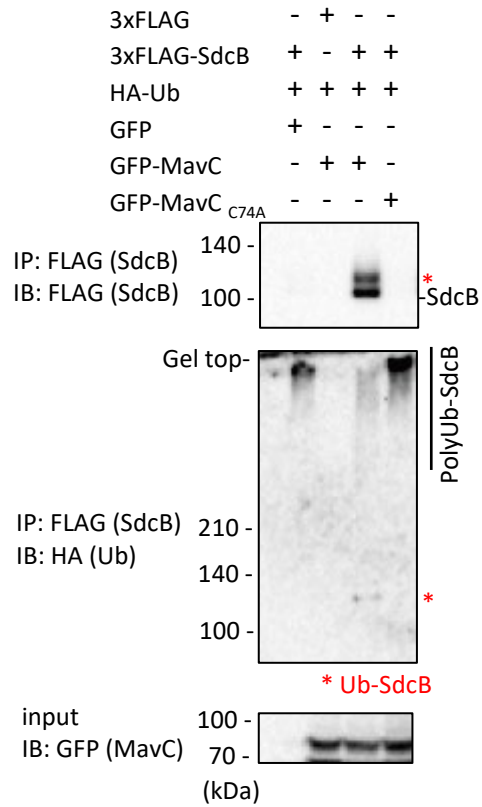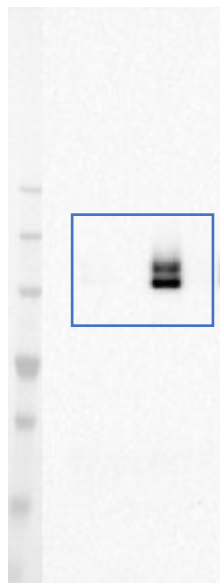

**Figure 5a  
top**

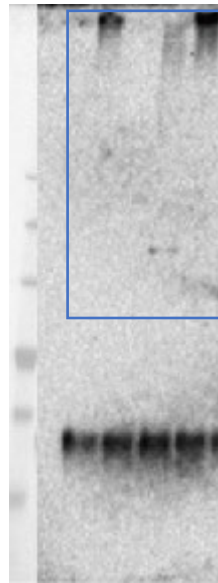

**Figure 5a  
middle**

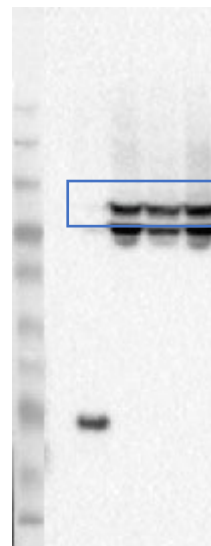

**Figure 5a  
bottom**

Supplement: Figure 5—source data 2. [file elife-89002-fig5-data2.pdf]
