# Supplementary figures and images for: Multi-tiered actions of Legionella effectors to modulate host Rab10 dynamics (part 2 of 2)
Source: eLife. 2024 May 21;12:RP89002. doi: 10.7554/eLife.89002 (PMC11108646; doi:10.7554/eLife.89002)

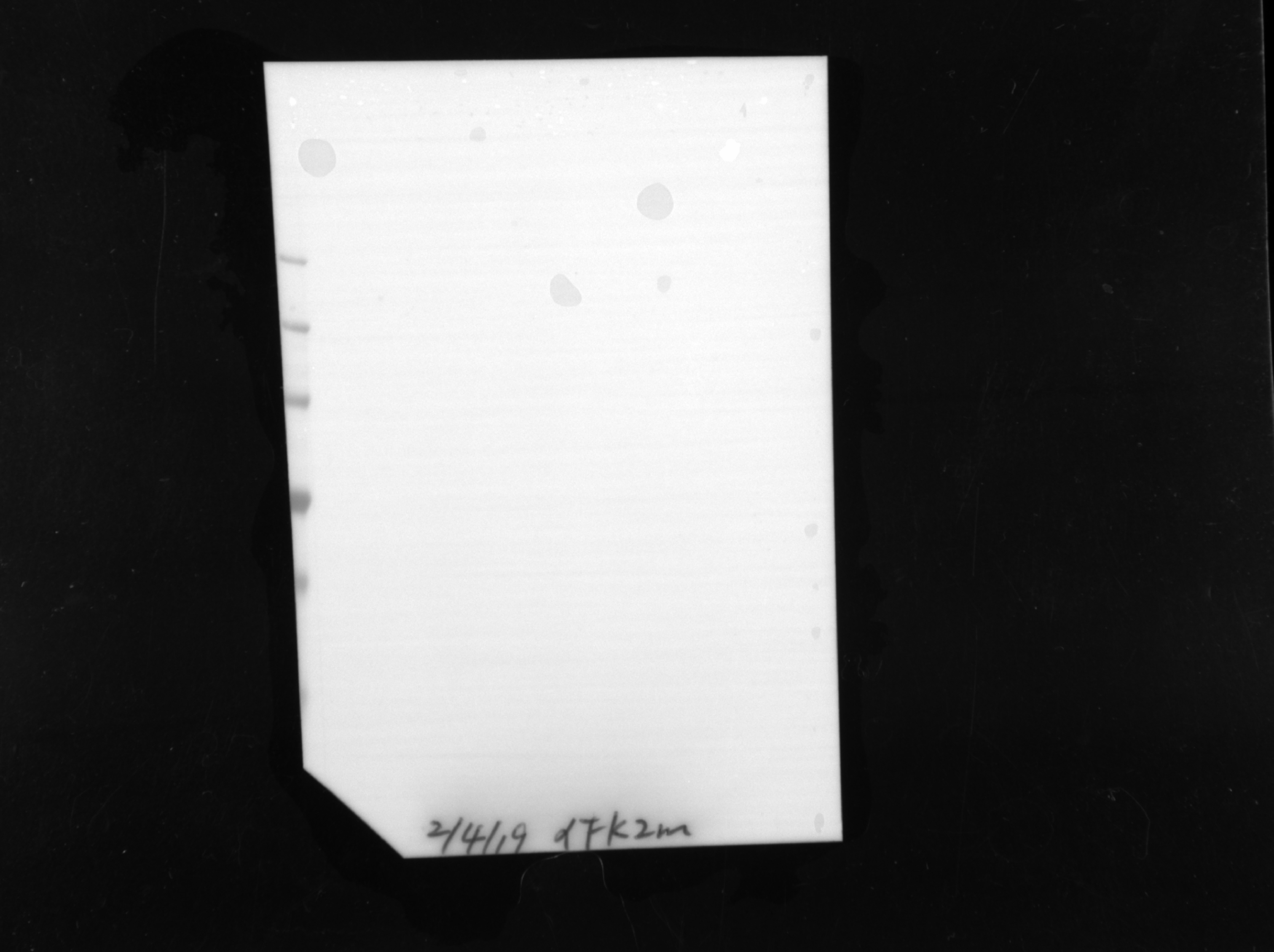

Supplement: Figure 5—source data 3. [file elife-89002-fig5-data3.zip › anti-FK2 Marker.jpg]

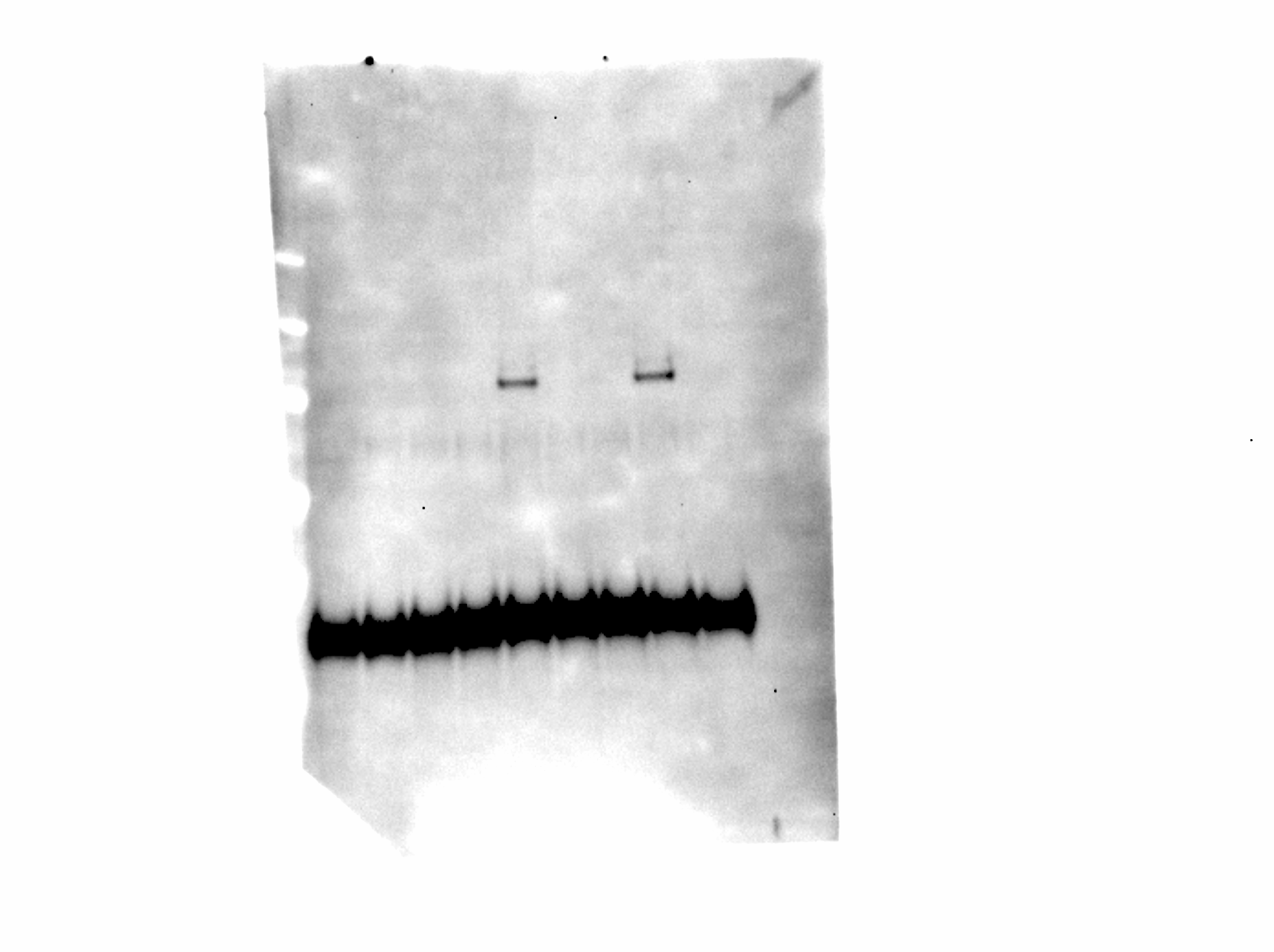

Supplement: Figure 5—source data 3. [file elife-89002-fig5-data3.zip › anti-FK2_Exposure_379.3sec.jpg]

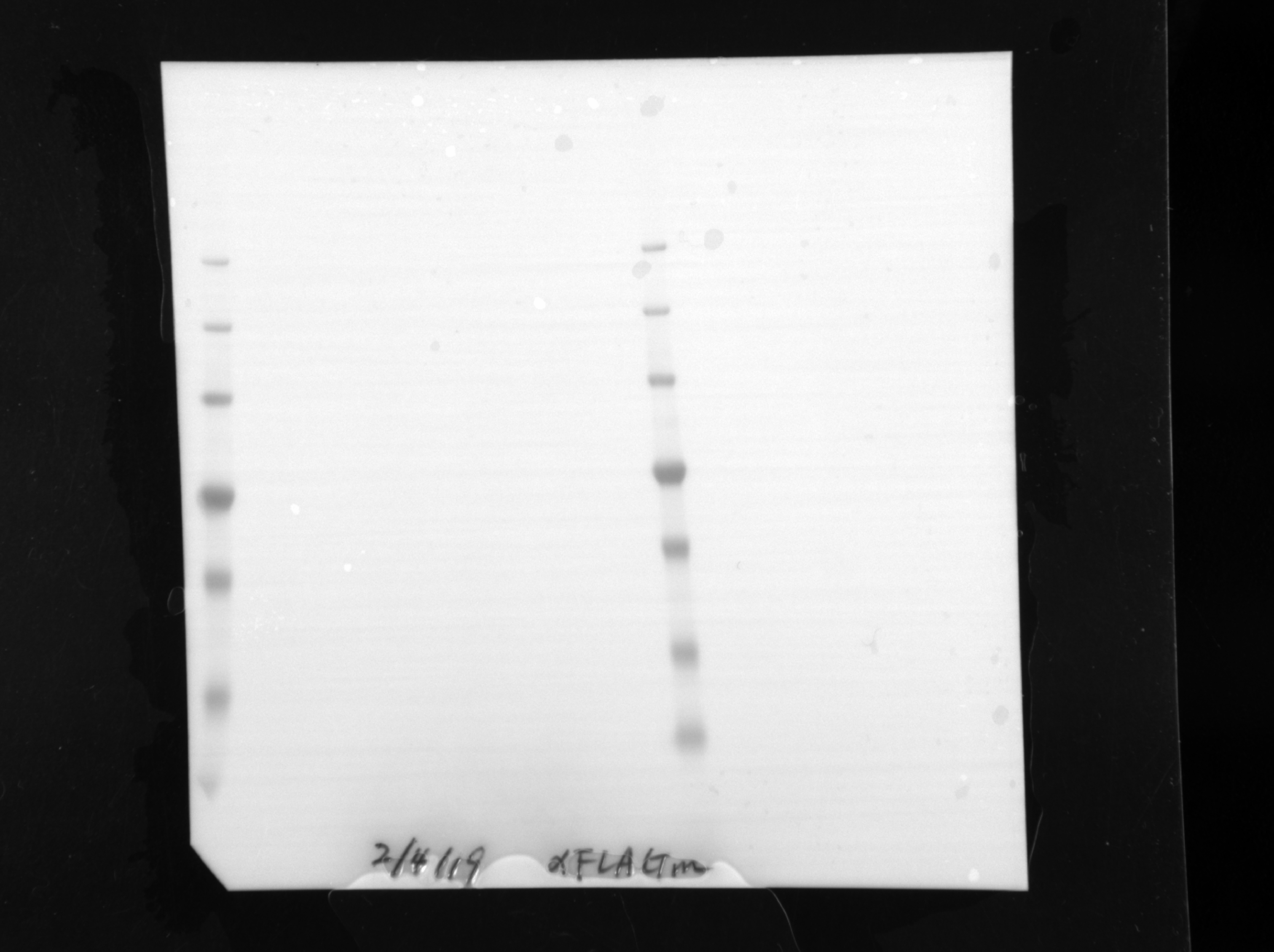

Supplement: Figure 5—source data 3. [file elife-89002-fig5-data3.zip › anti-FLAG Marker.jpg]

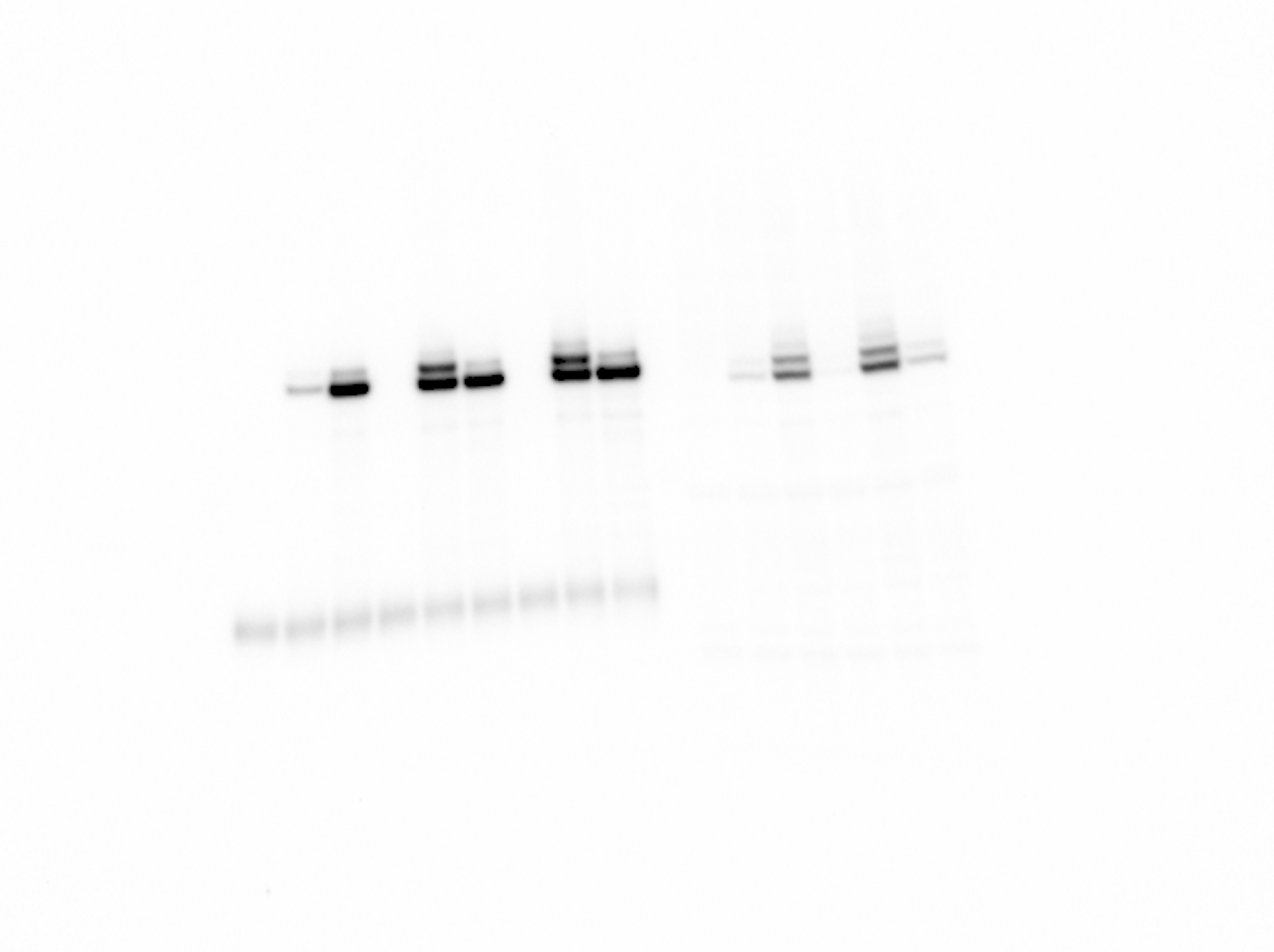

Supplement: Figure 5—source data 3. [file elife-89002-fig5-data3.zip › anti-FLAG_Exposure_28.2sec.jpg]

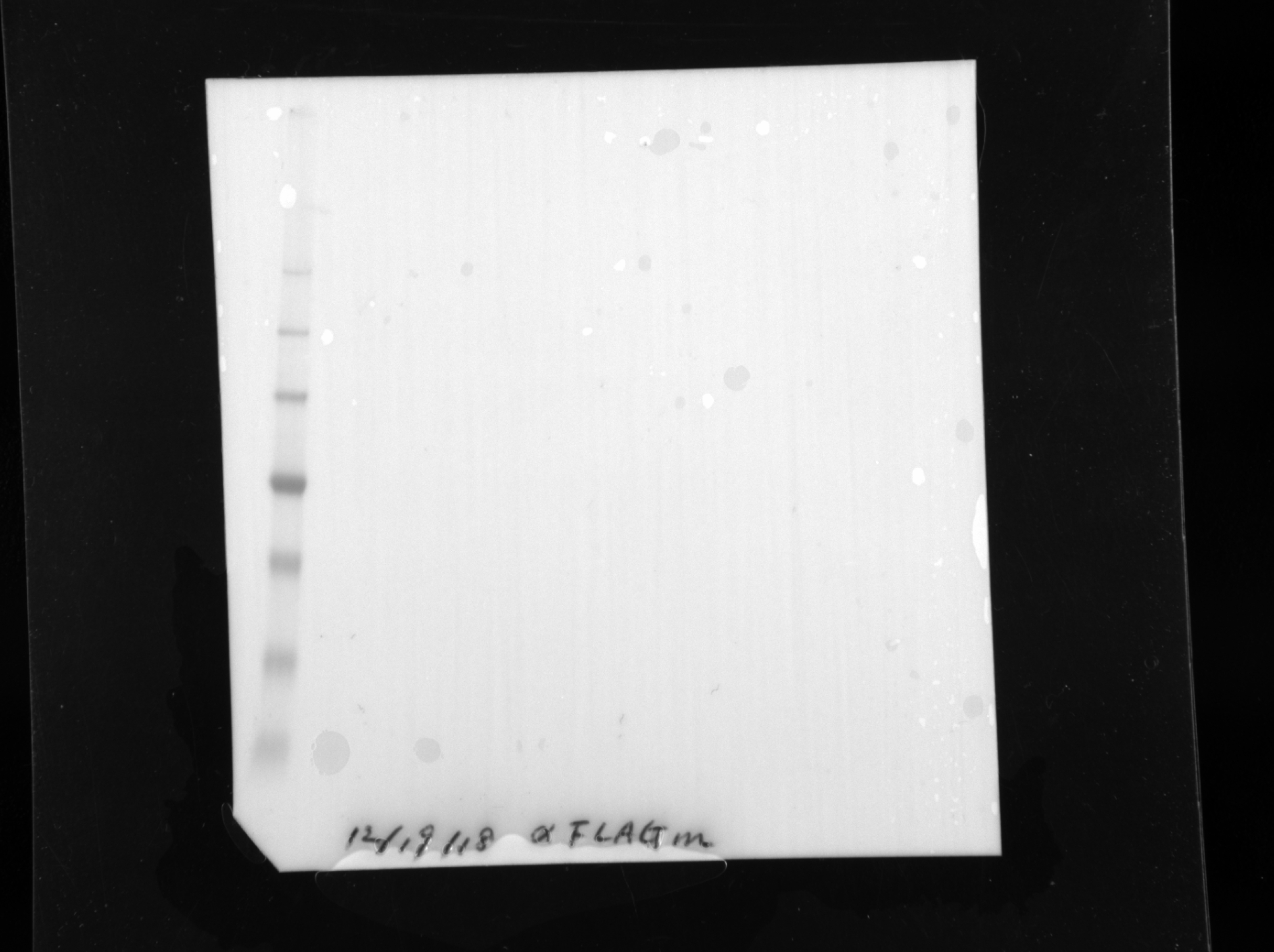

Supplement: Figure 5—source data 3. [file elife-89002-fig5-data3.zip › input anti-GFP Marker.jpg]

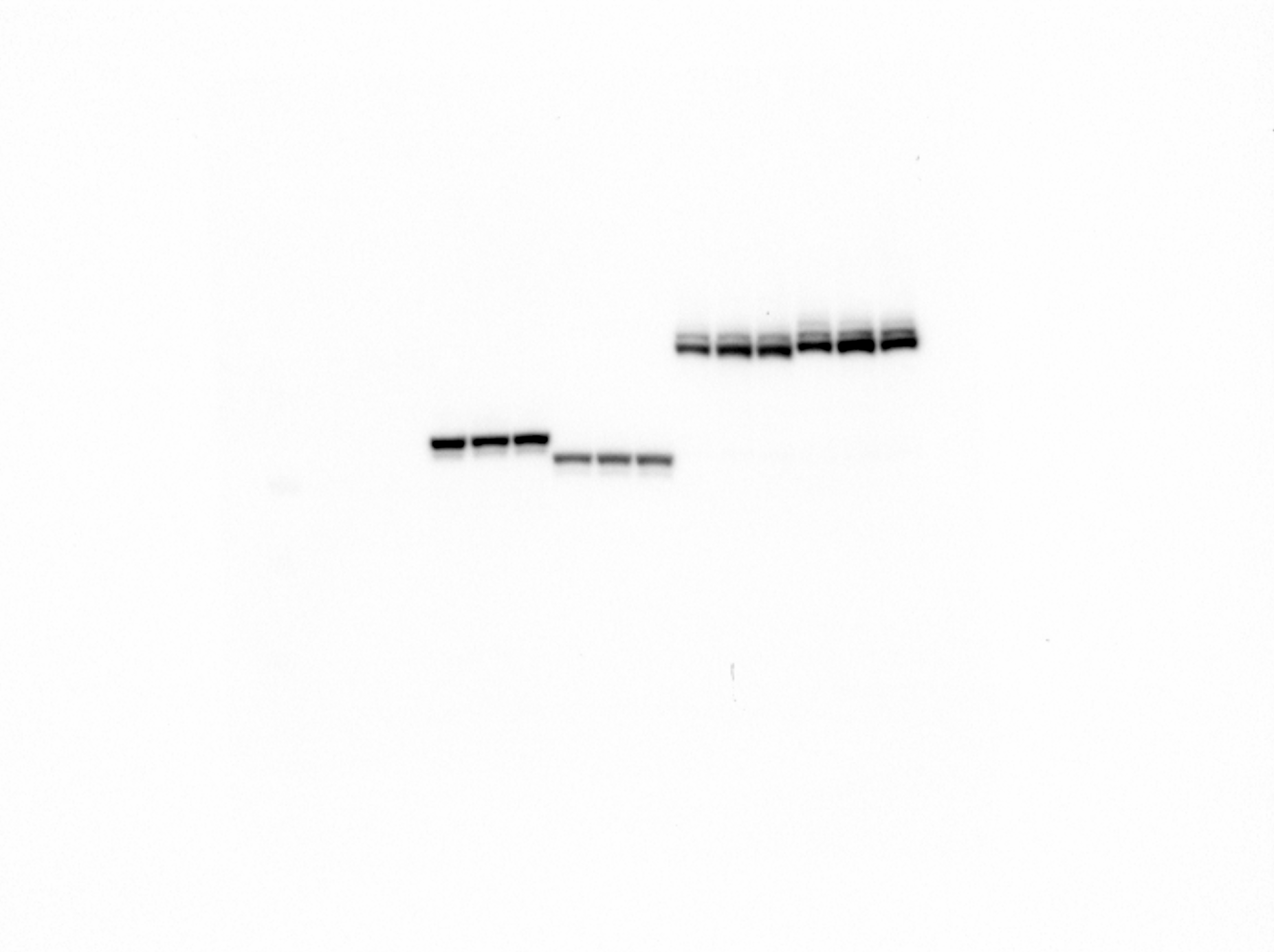

Supplement: Figure 5—source data 3. [file elife-89002-fig5-data3.zip › input anti-GFP_Exposure_133.9sec.jpg]

**b**

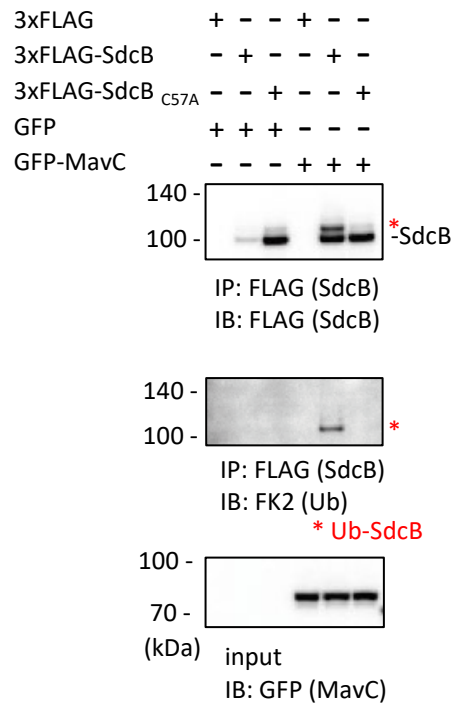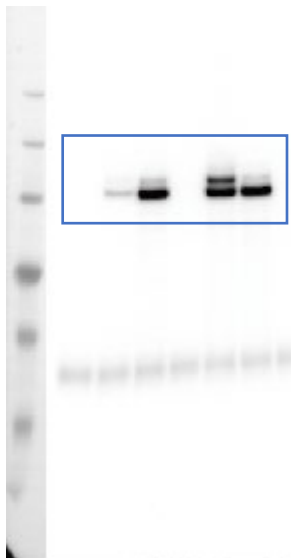

**Figure 5b  
top**

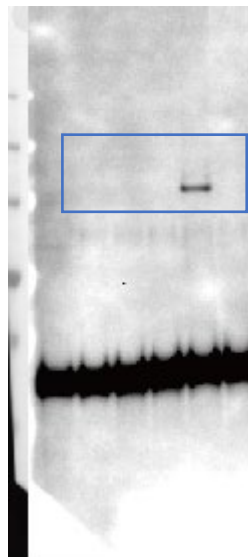

**Figure 5b  
middle**

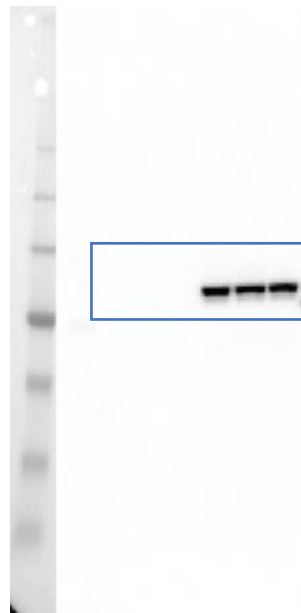

**Figure 5b  
bottom**

Supplement: Figure 5—source data 4. [file elife-89002-fig5-data4.pdf]

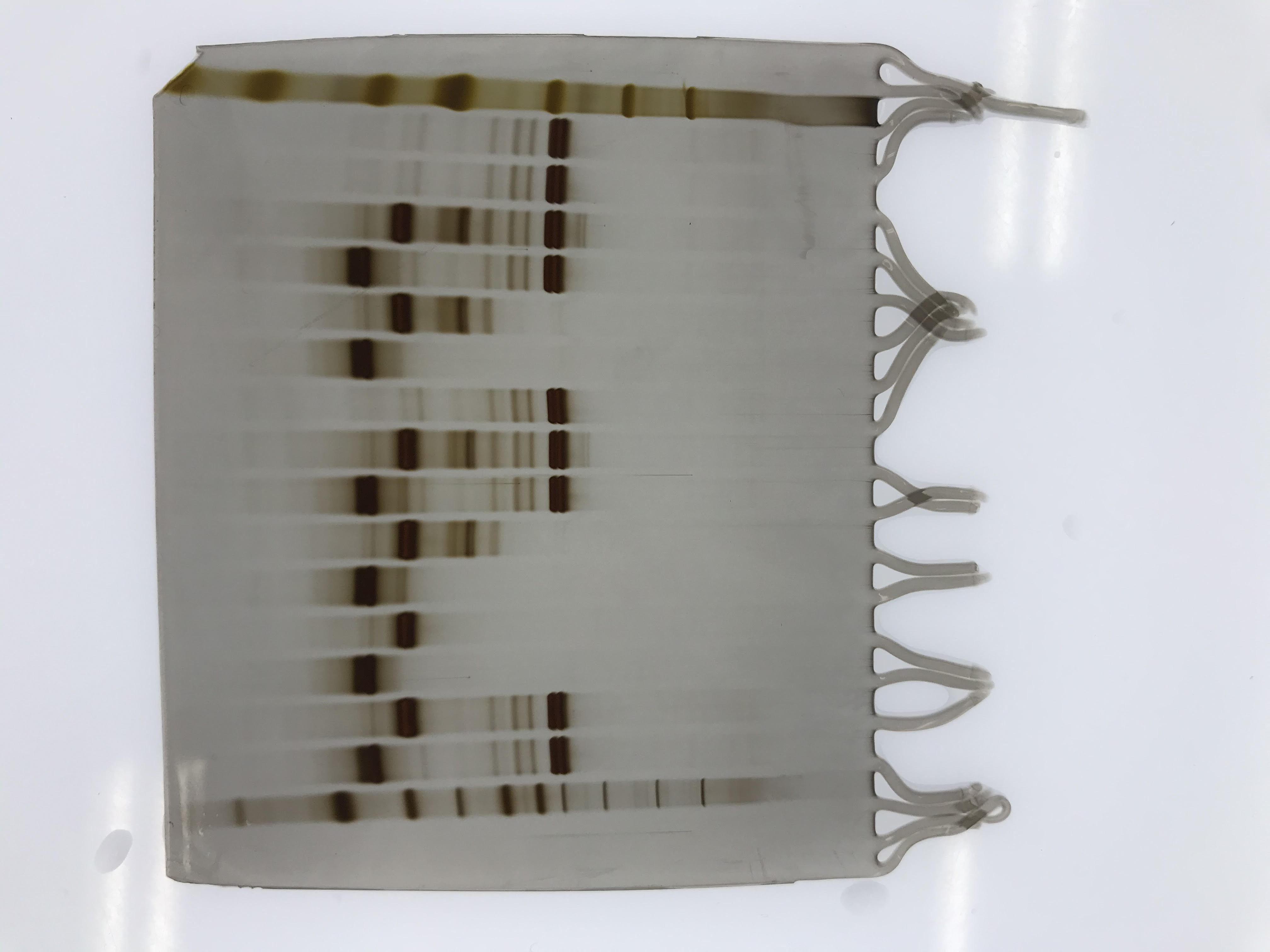

Supplement: Figure 5—source data 5. [file elife-89002-fig5-data5.zip › 200721 in vitro transglutaminase assay silverstaining.jpg]

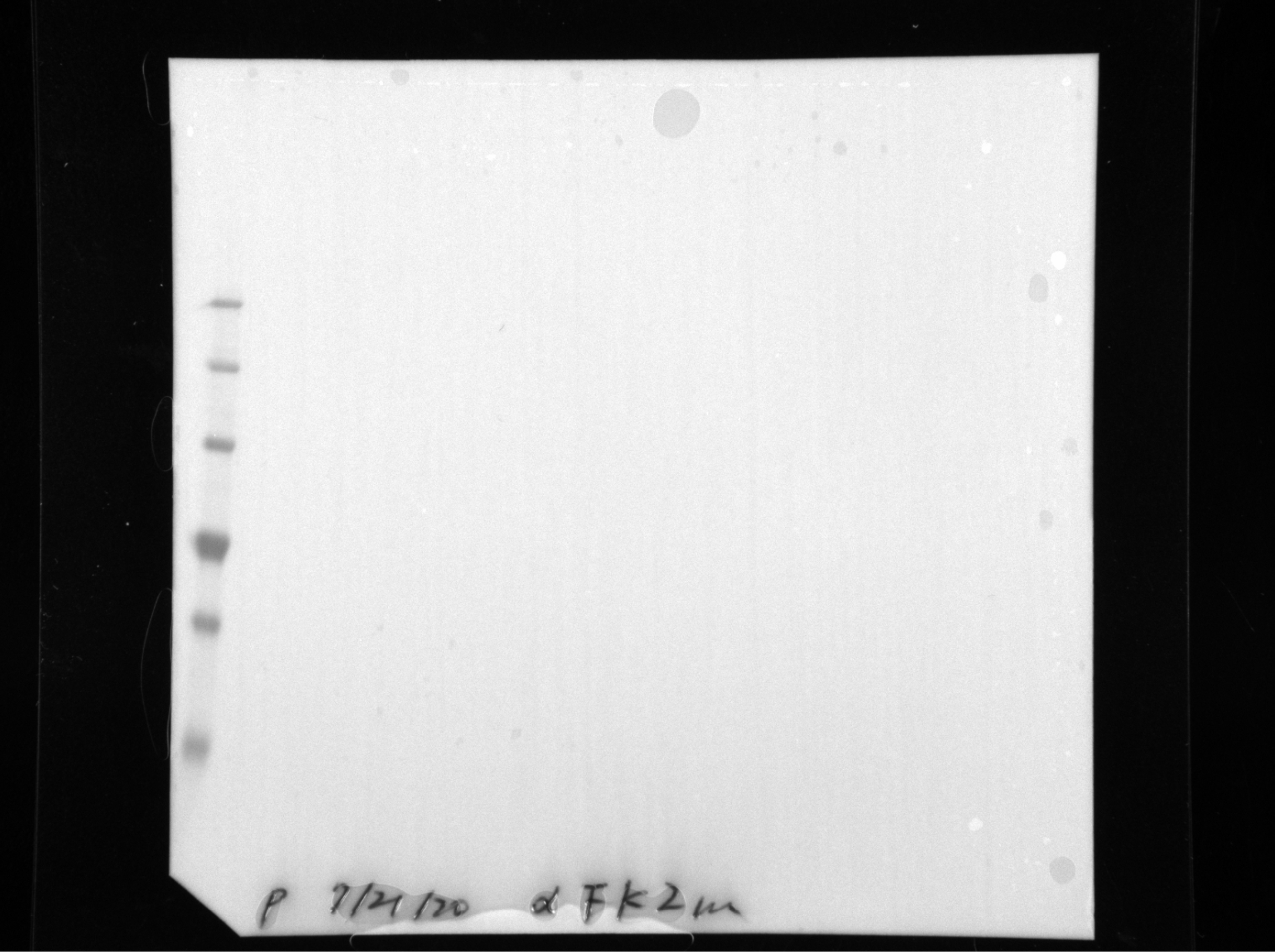

Supplement: Figure 5—source data 5. [file elife-89002-fig5-data5.zip › anti-FK2 Marker.jpg]

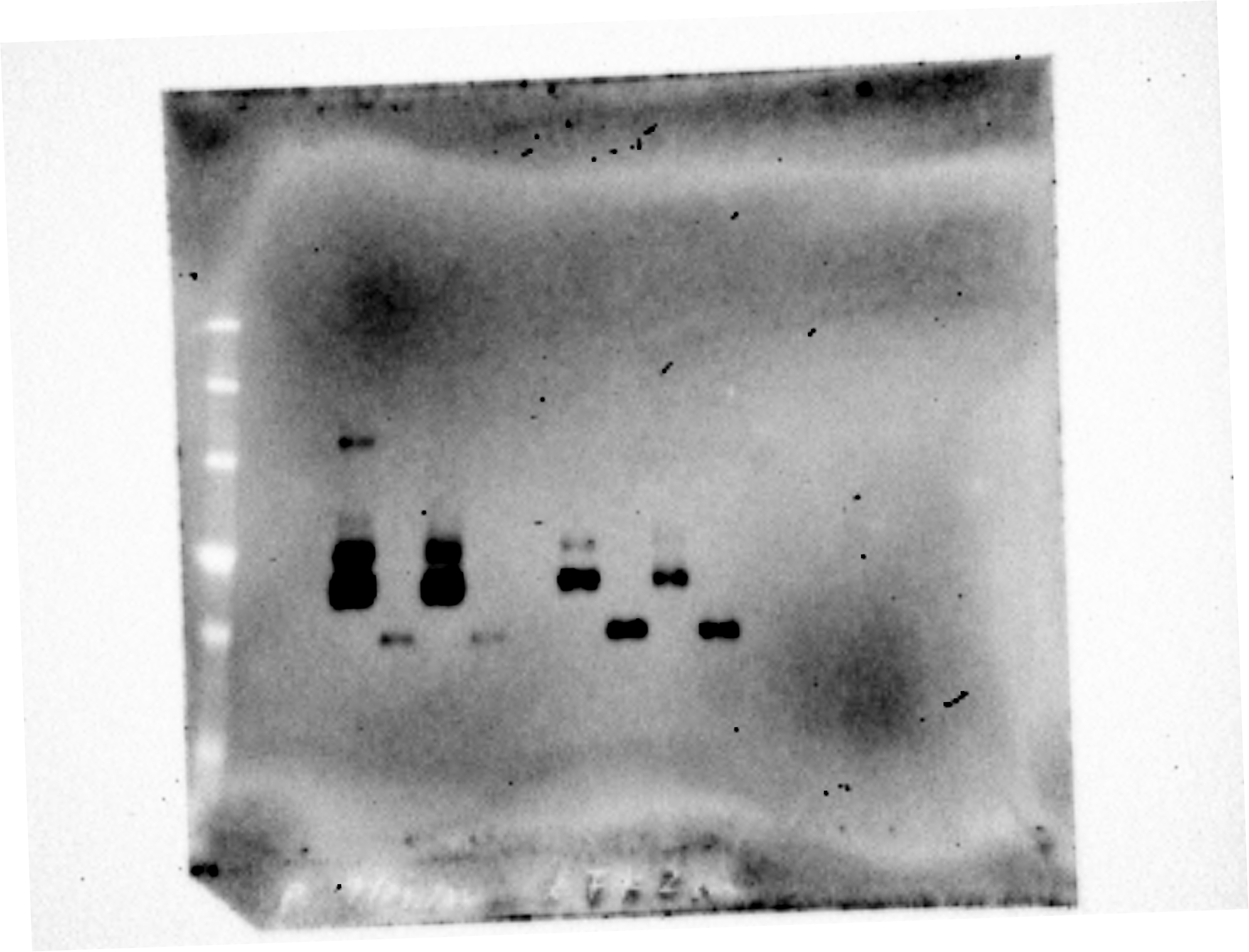

Supplement: Figure 5—source data 5. [file elife-89002-fig5-data5.zip › anti-FK2_Exposure_810.3sec.jpg]

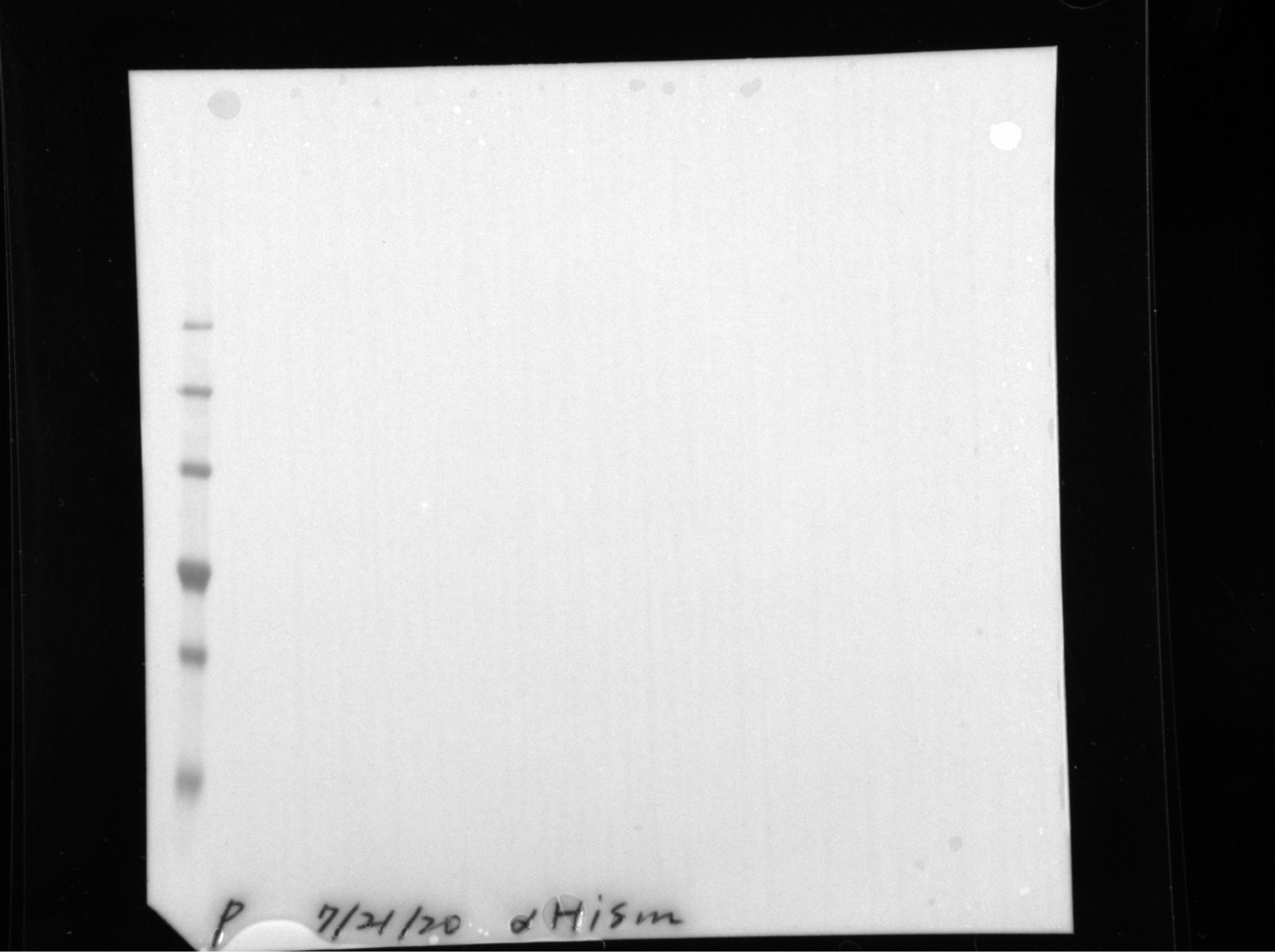

Supplement: Figure 5—source data 5. [file elife-89002-fig5-data5.zip › anti-His Marker.jpg]

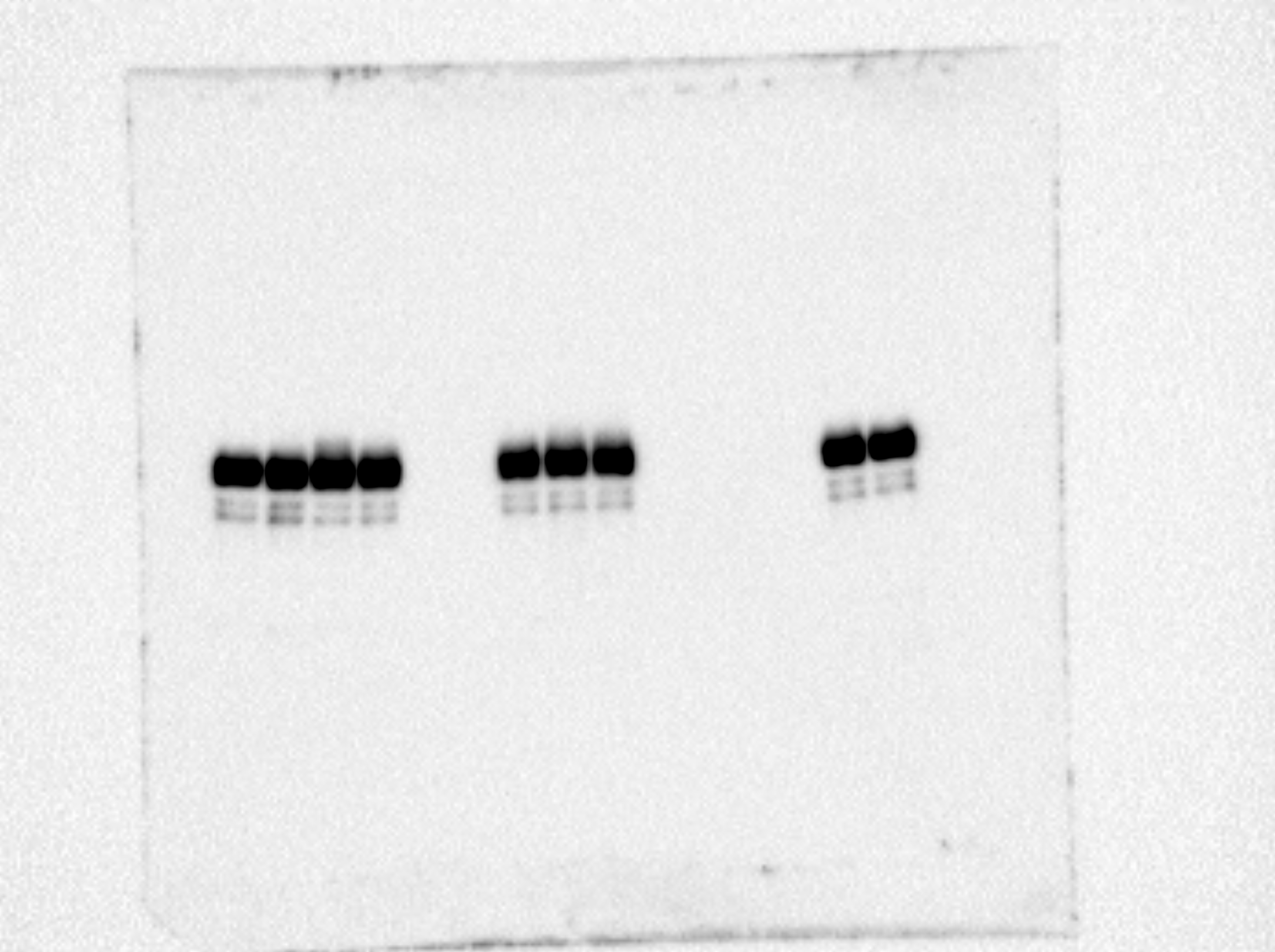

Supplement: Figure 5—source data 5. [file elife-89002-fig5-data5.zip › anti-His_Exposure_90.7sec.jpg]

**C**

|          |   |   |   |   |   |   |
|----------|---|---|---|---|---|---|
| Ub       | - | + | + | + | + | + |
| His-SdcB | + | + | + | + | - | - |
| MavC     | - | - | + | - | + | - |
| MvcA     | - | - | - | + | - | + |

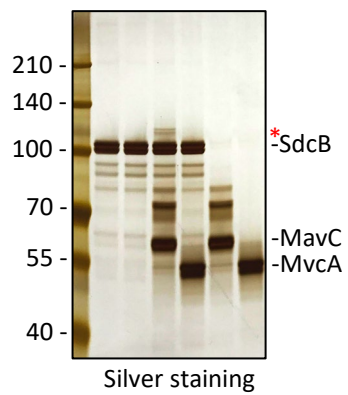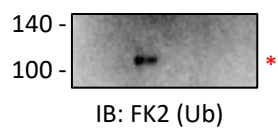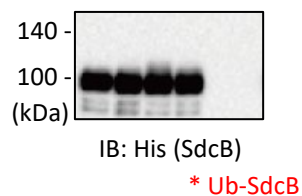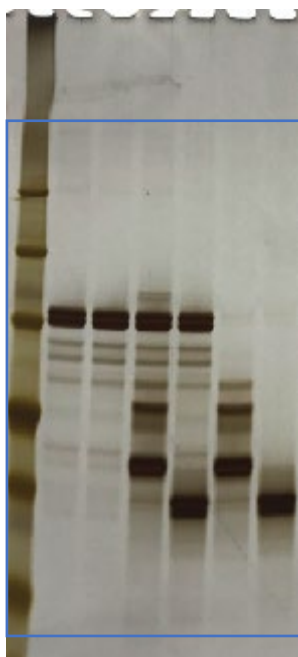

**Figure 5c**  
**top**

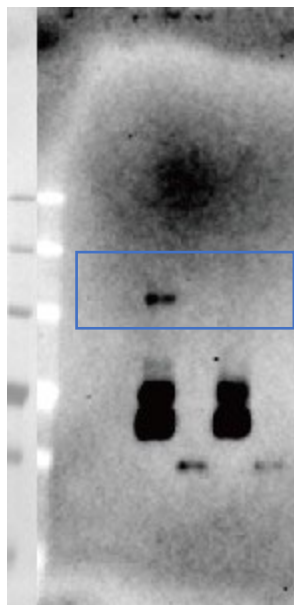

**Figure 5c**  
**middle**

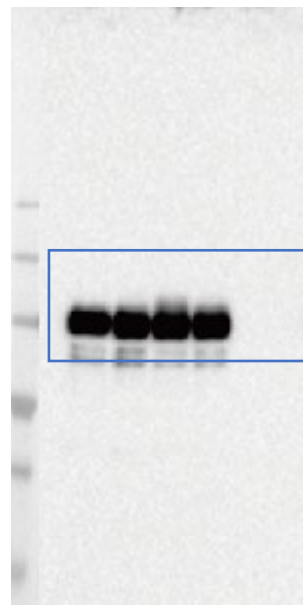

**Figure 5c**  
**bottom**

Supplement: Figure 5—source data 6. [file elife-89002-fig5-data6.pdf]

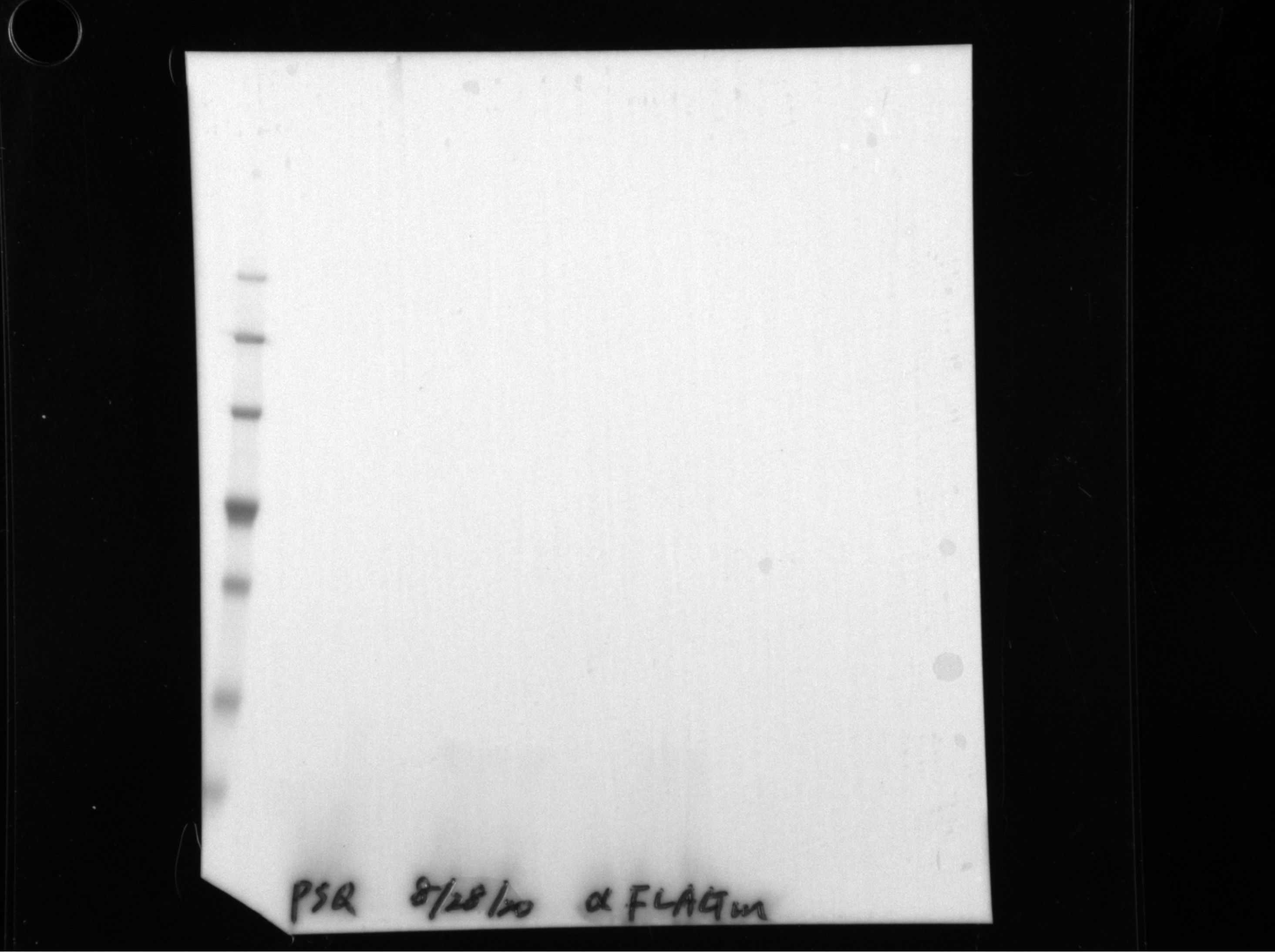

Supplement: Figure 5—source data 7. [file elife-89002-fig5-data7.zip › anti-FLAG Marker.jpg]

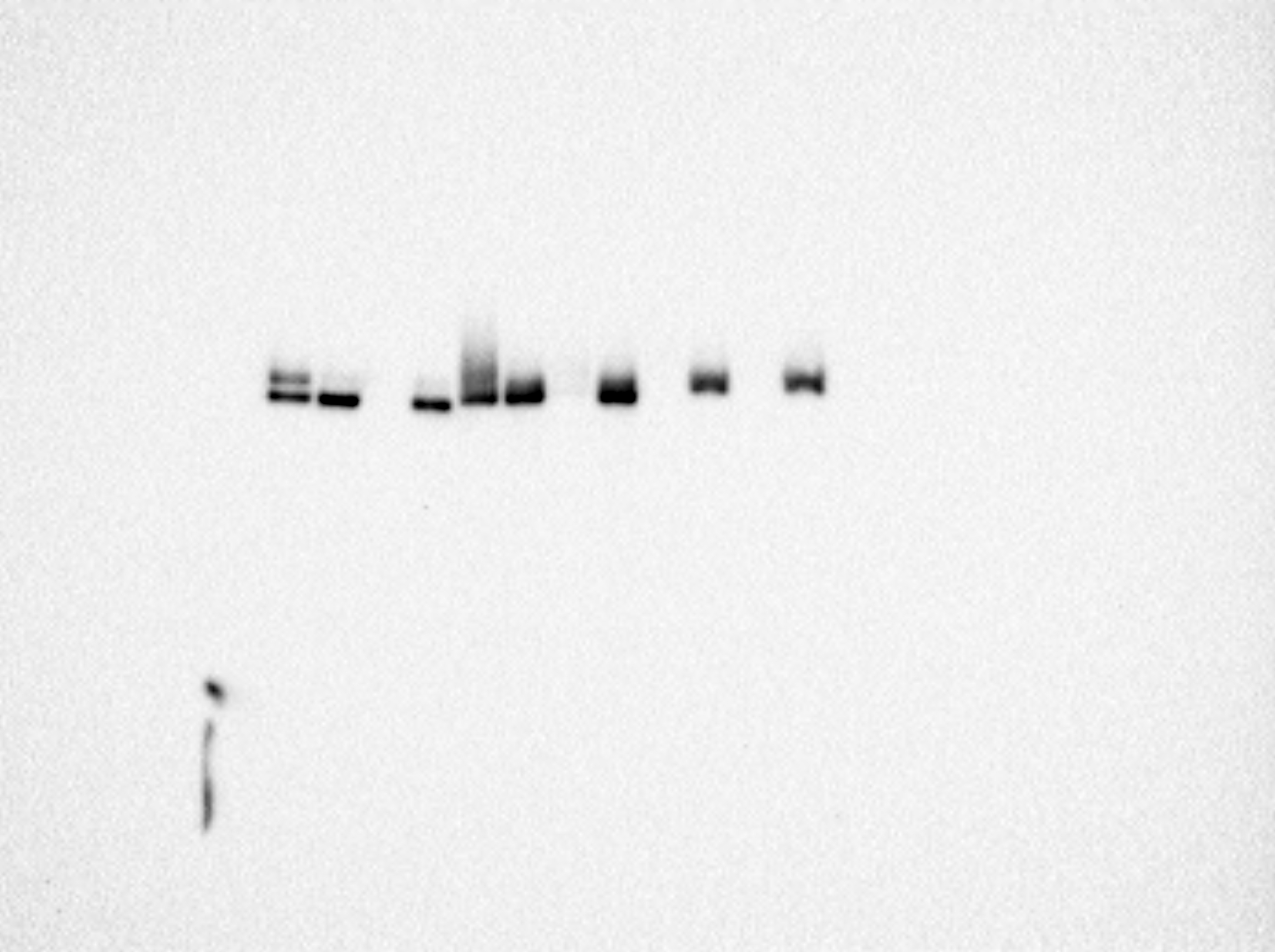

Supplement: Figure 5—source data 7. [file elife-89002-fig5-data7.zip › anti-FLAG_Exposure_42.3sec.jpg]

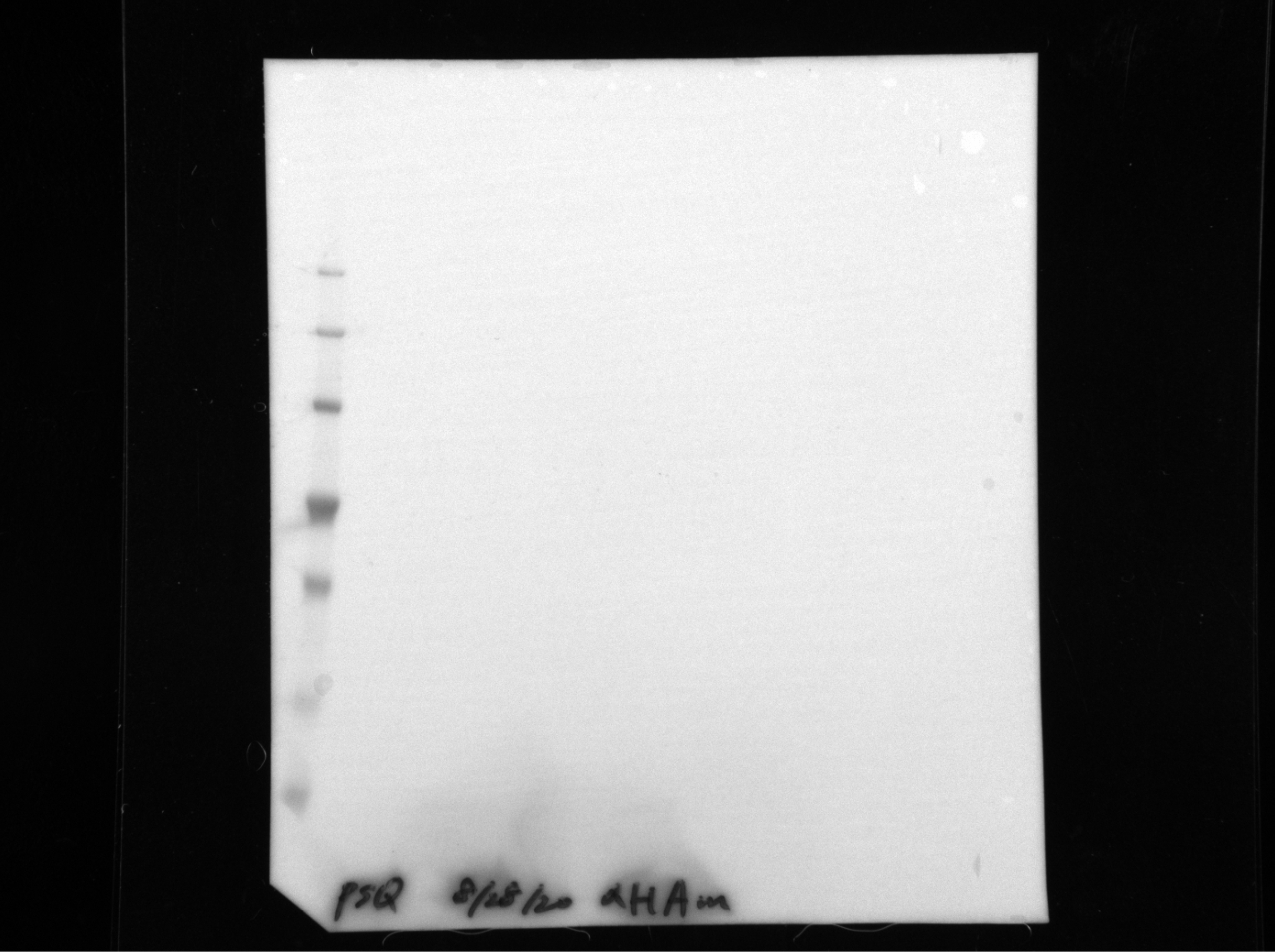

Supplement: Figure 5—source data 7. [file elife-89002-fig5-data7.zip › anti-HA pierce Marker.jpg]

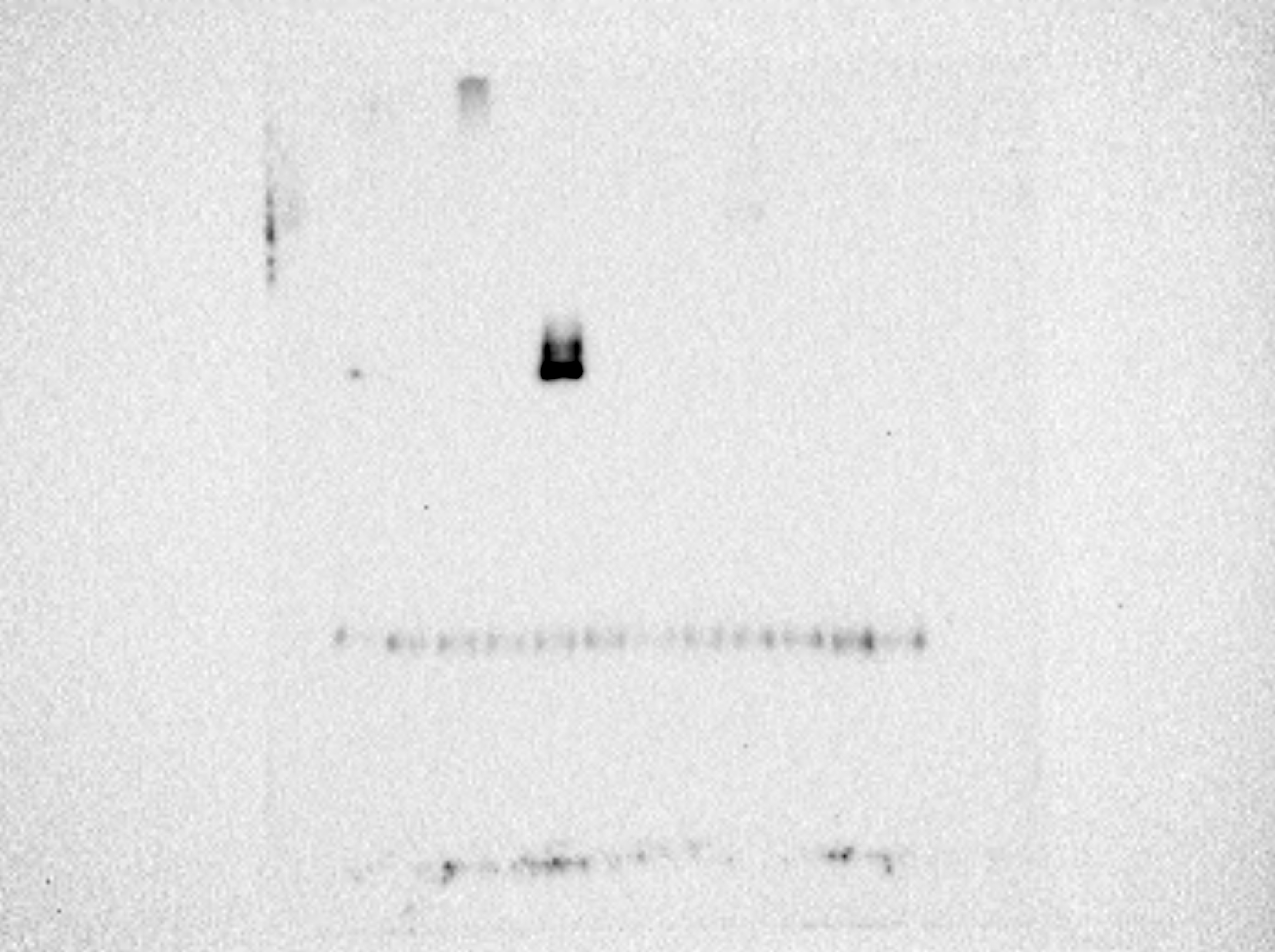

Supplement: Figure 5—source data 7. [file elife-89002-fig5-data7.zip › anti-HA pierce_58.8sec.jpg]

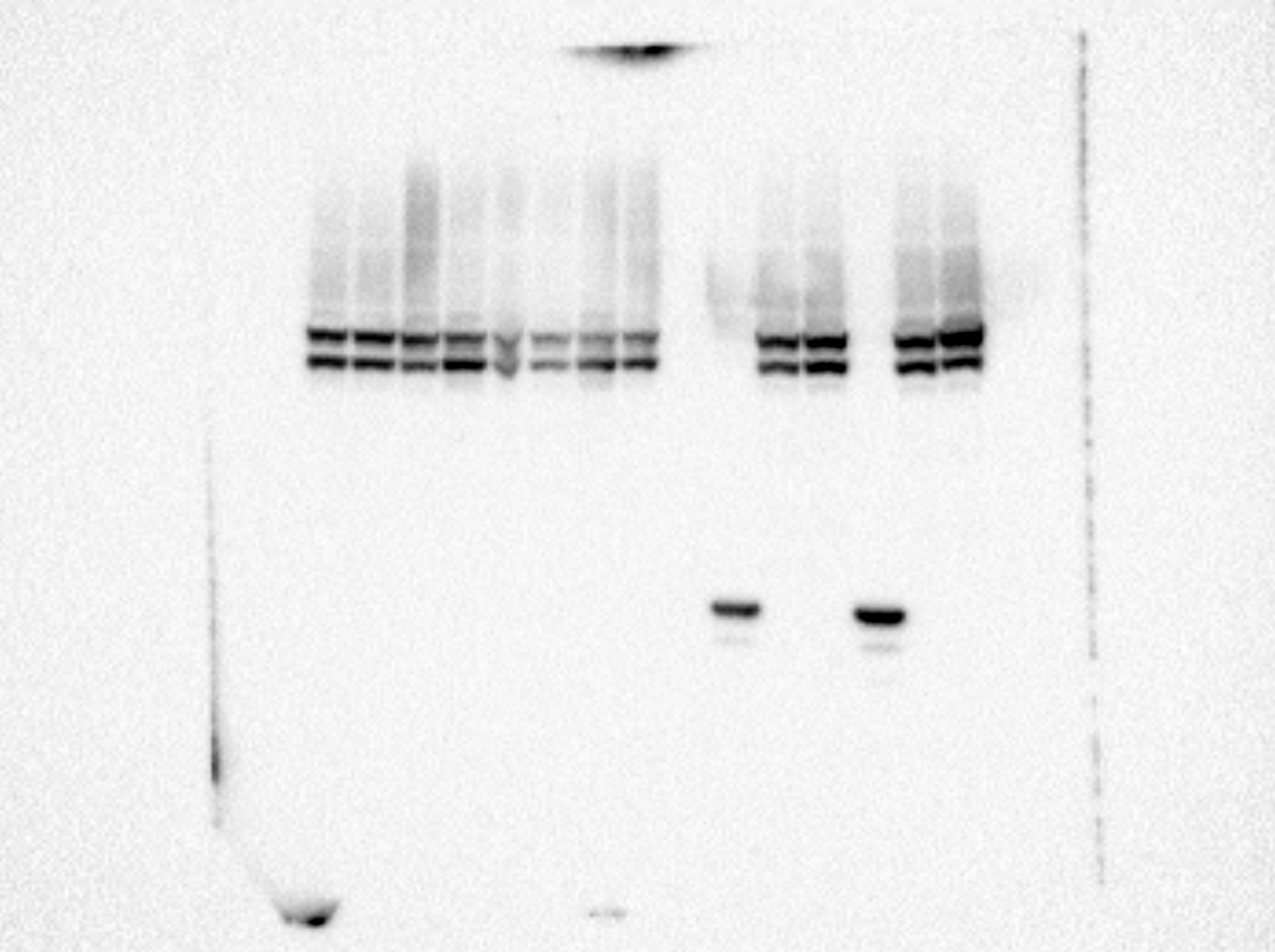

Supplement: Figure 5—source data 7. [file elife-89002-fig5-data7.zip › gel2 anti-GFPrb_Exposure_16.5sec.jpg]

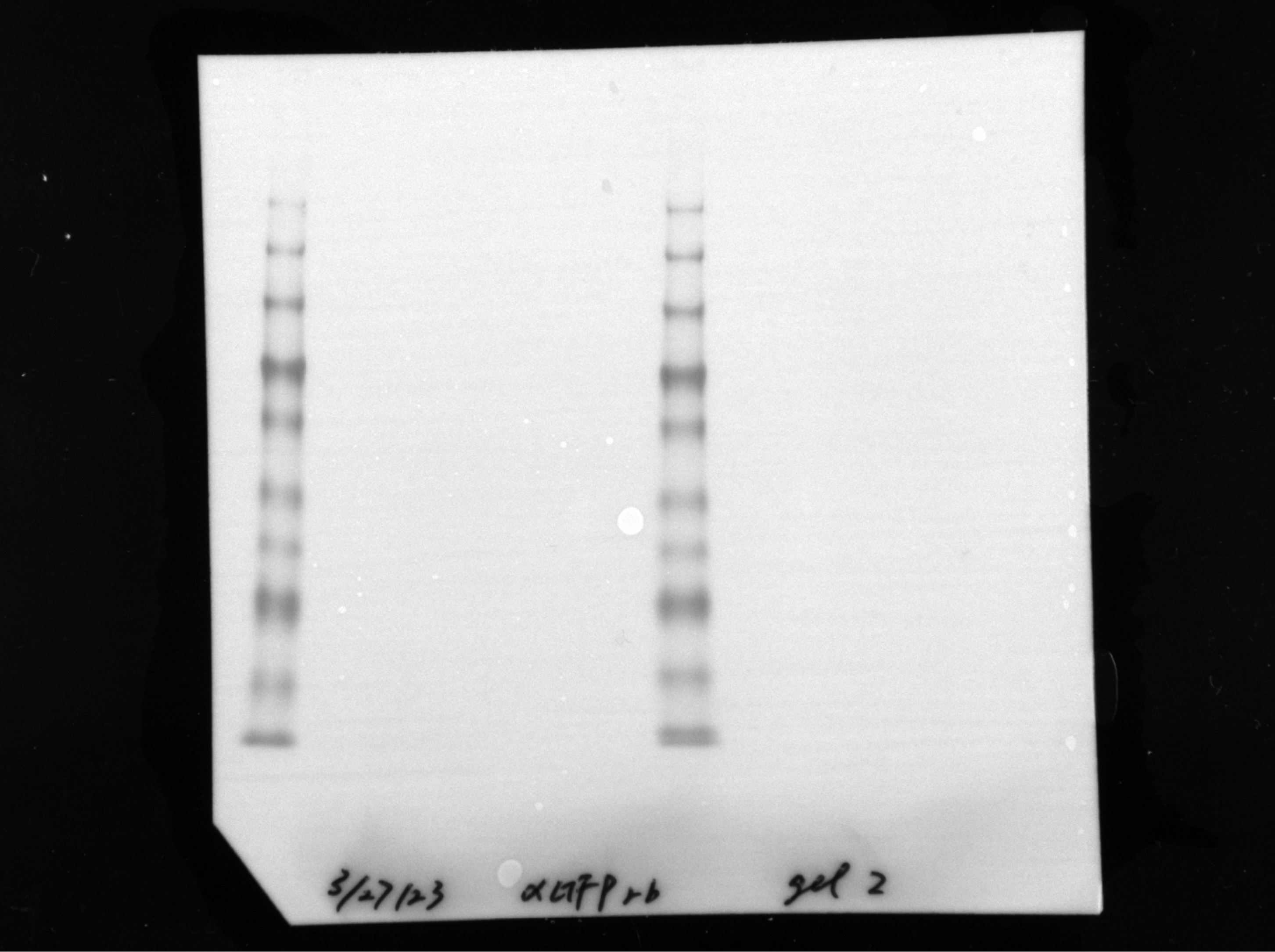

Supplement: Figure 5—source data 7. [file elife-89002-fig5-data7.zip › gel2 anti-GFPrb_Marker.jpg]

**d**

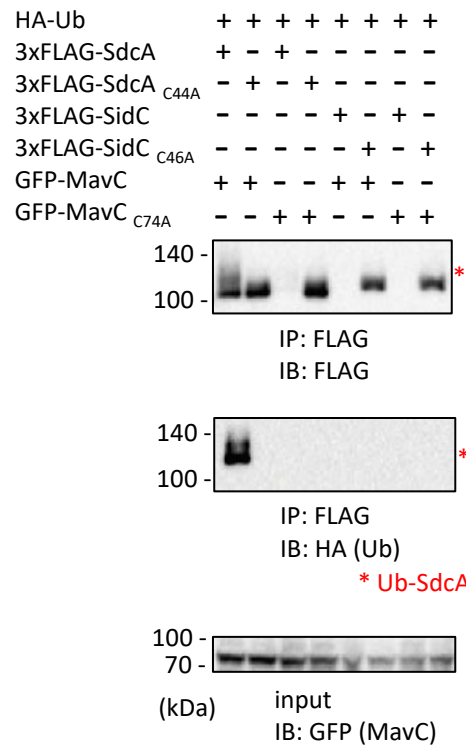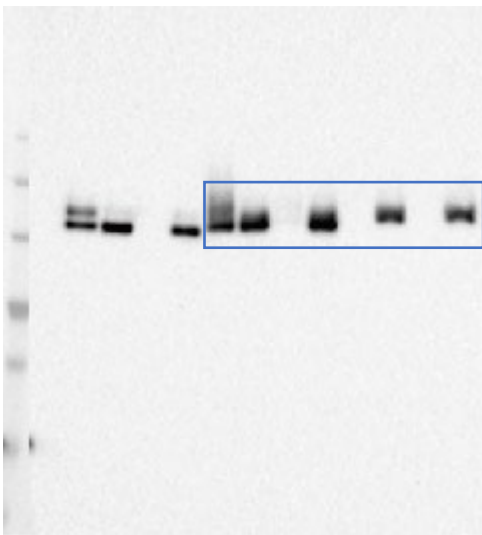

**Figure 5d**  
**top**

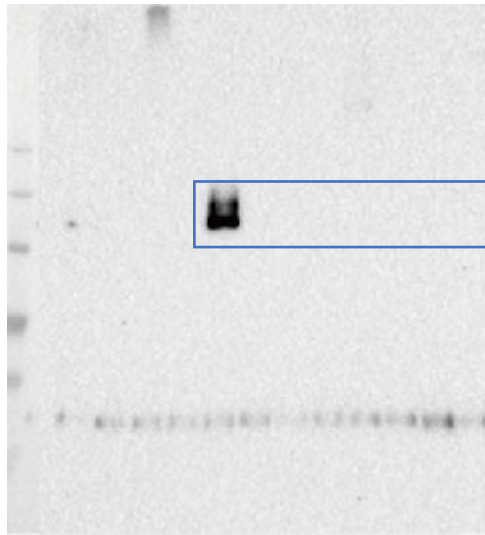

**Figure 5d**  
**middle**

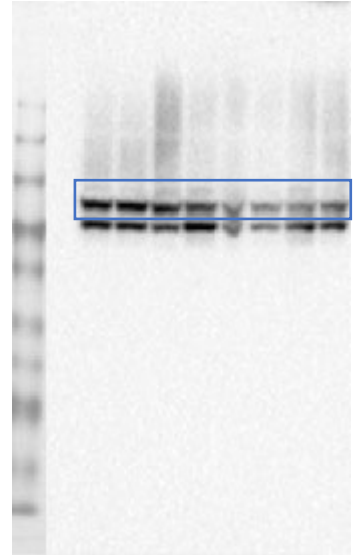

**Figure 5d**  
**bottom**

Supplement: Figure 5—source data 8. [file elife-89002-fig5-data8.pdf]

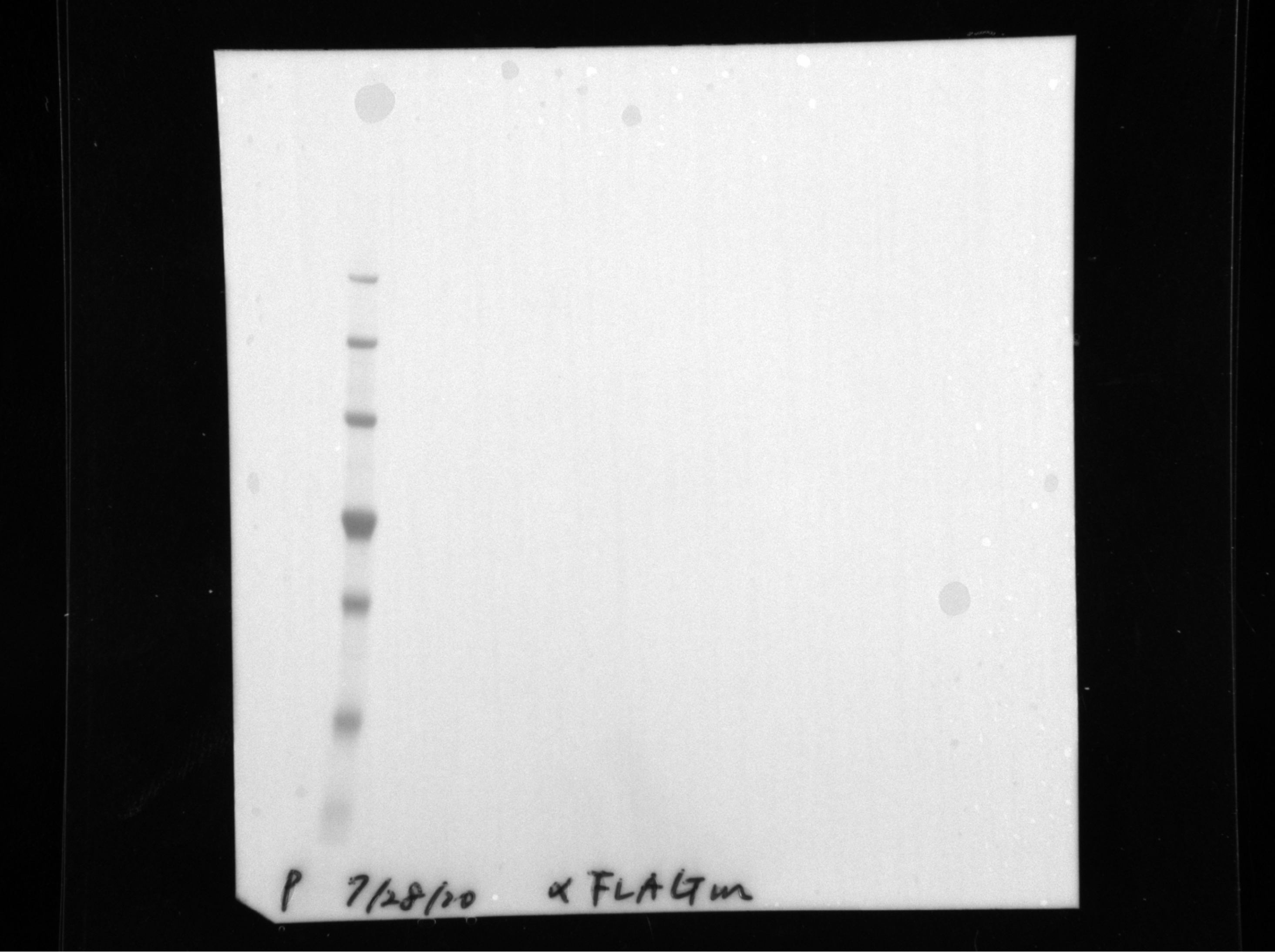

Supplement: Figure 5—source data 9. [file elife-89002-fig5-data9.zip › anti-FLAG Marker.jpg]

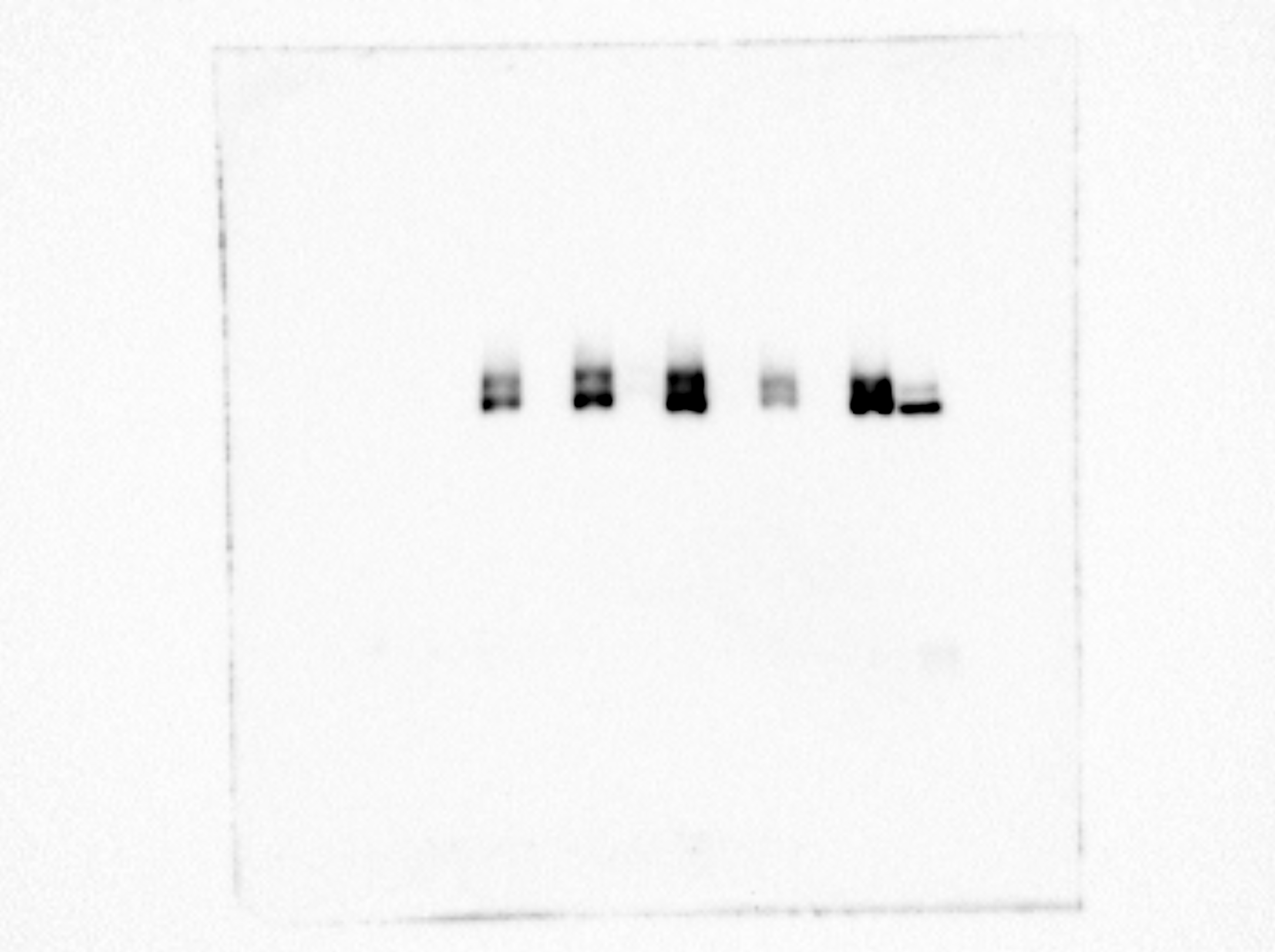

Supplement: Figure 5—source data 9. [file elife-89002-fig5-data9.zip › anti-FLAG_Exposure_90.7sec.jpg]

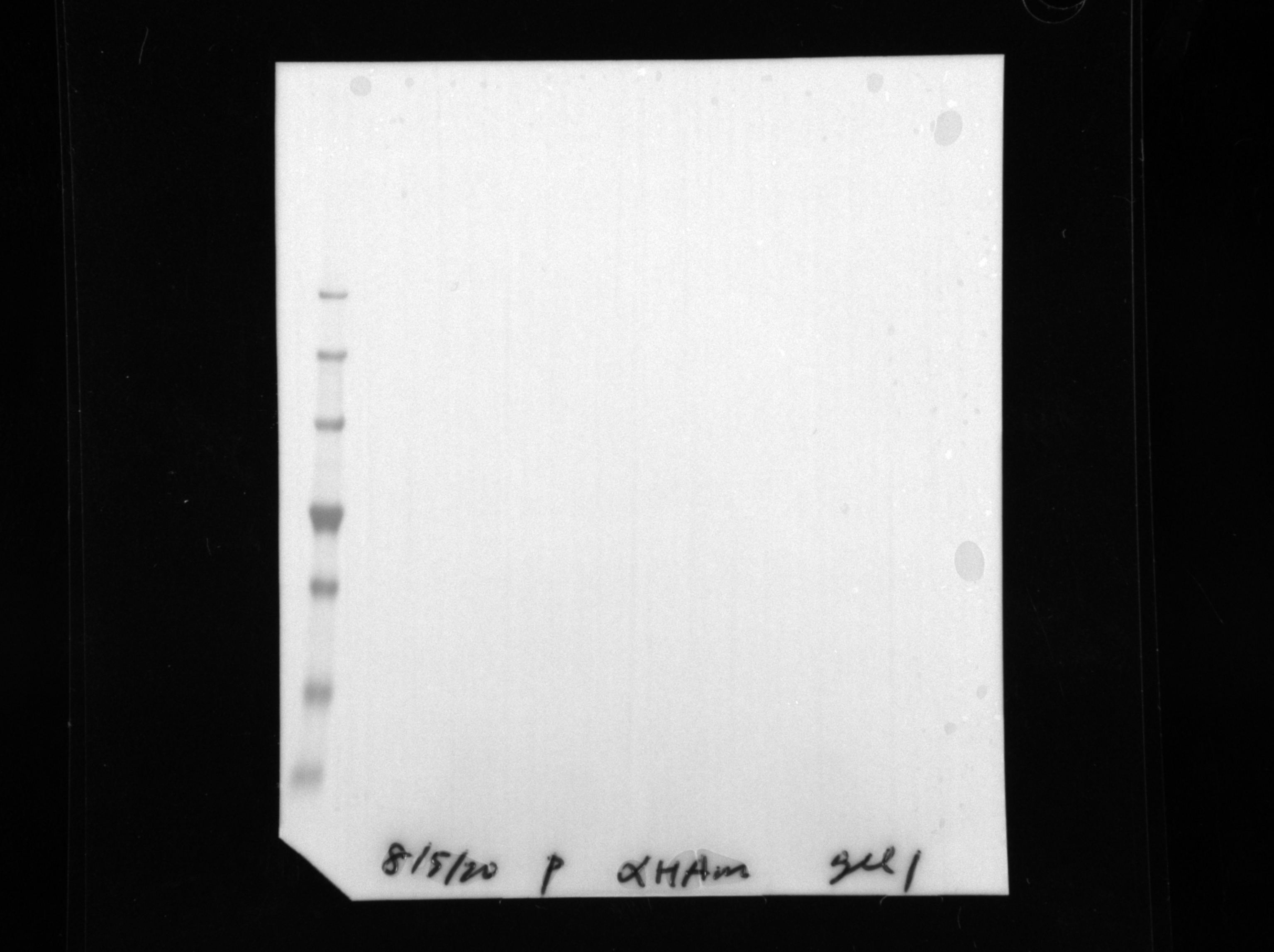

Supplement: Figure 5—source data 9. [file elife-89002-fig5-data9.zip › anti-HA immobilon P Marker.jpg]

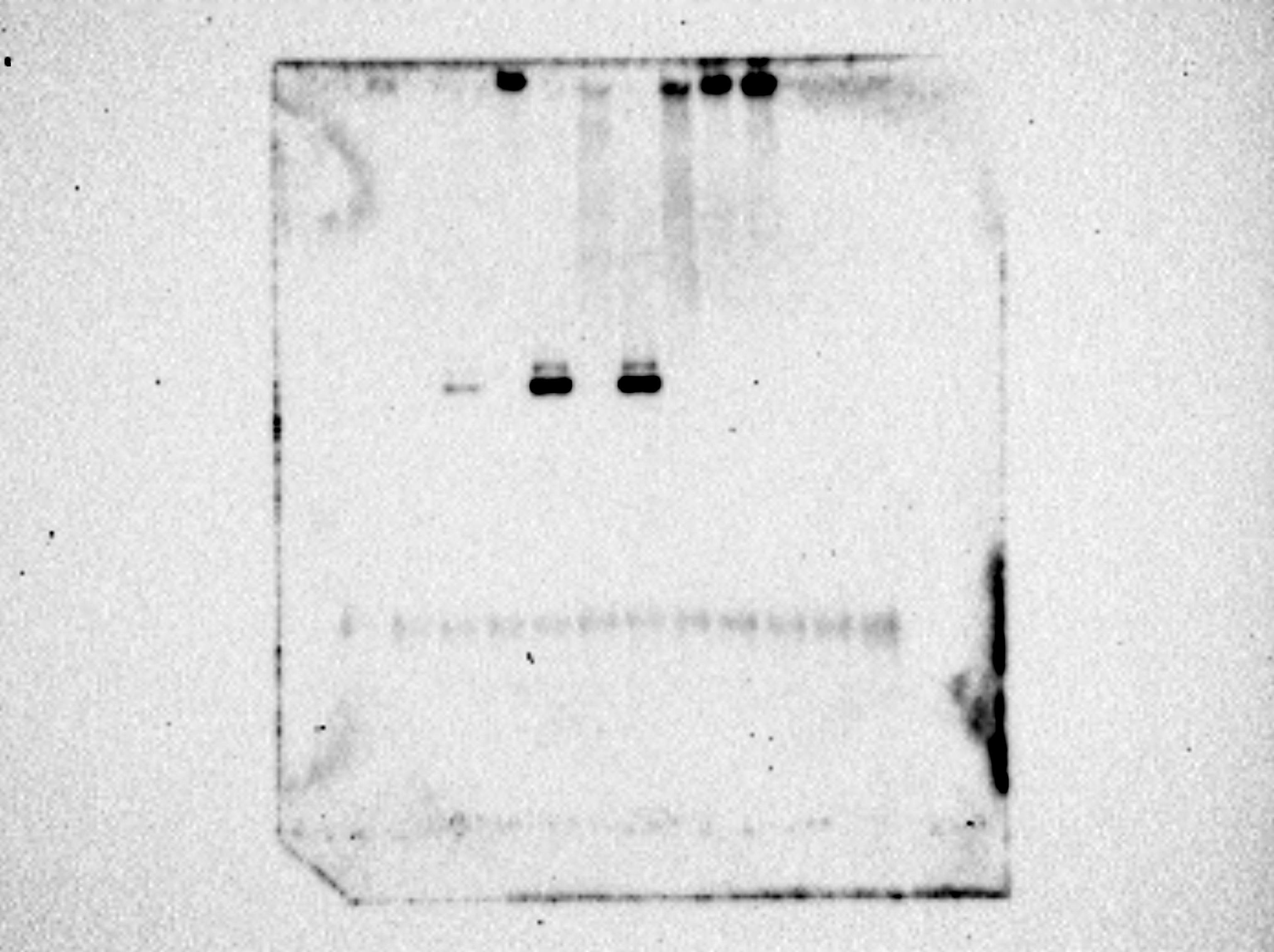

Supplement: Figure 5—source data 9. [file elife-89002-fig5-data9.zip › anti-HA immobilon P_Exposure_600.0sec.jpg]

**e**

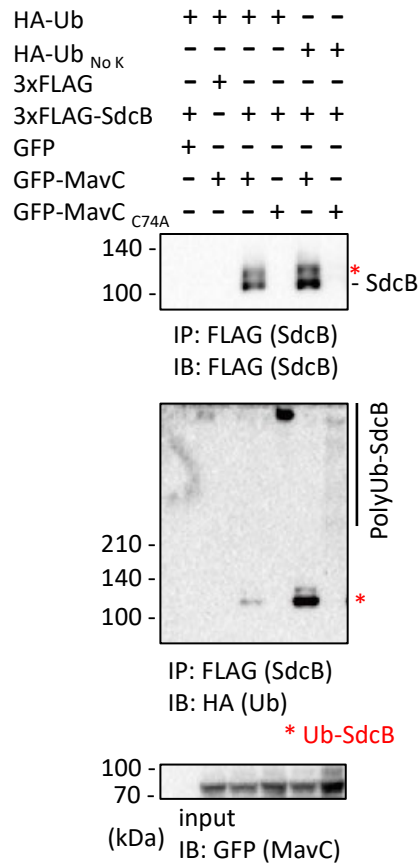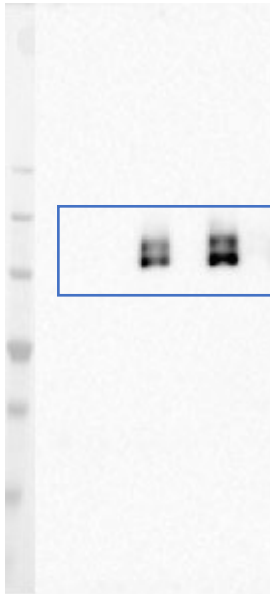

**Figure 5e**  
top

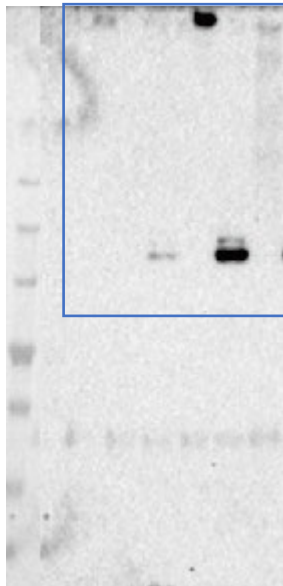

**Figure 5e**  
middle

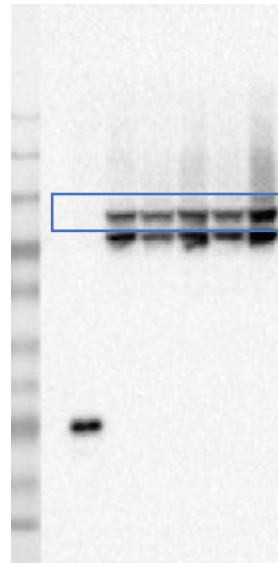

**Figure 5e**  
bottom

Supplement: Figure 5—source data 10. [file elife-89002-fig5-data10.pdf]

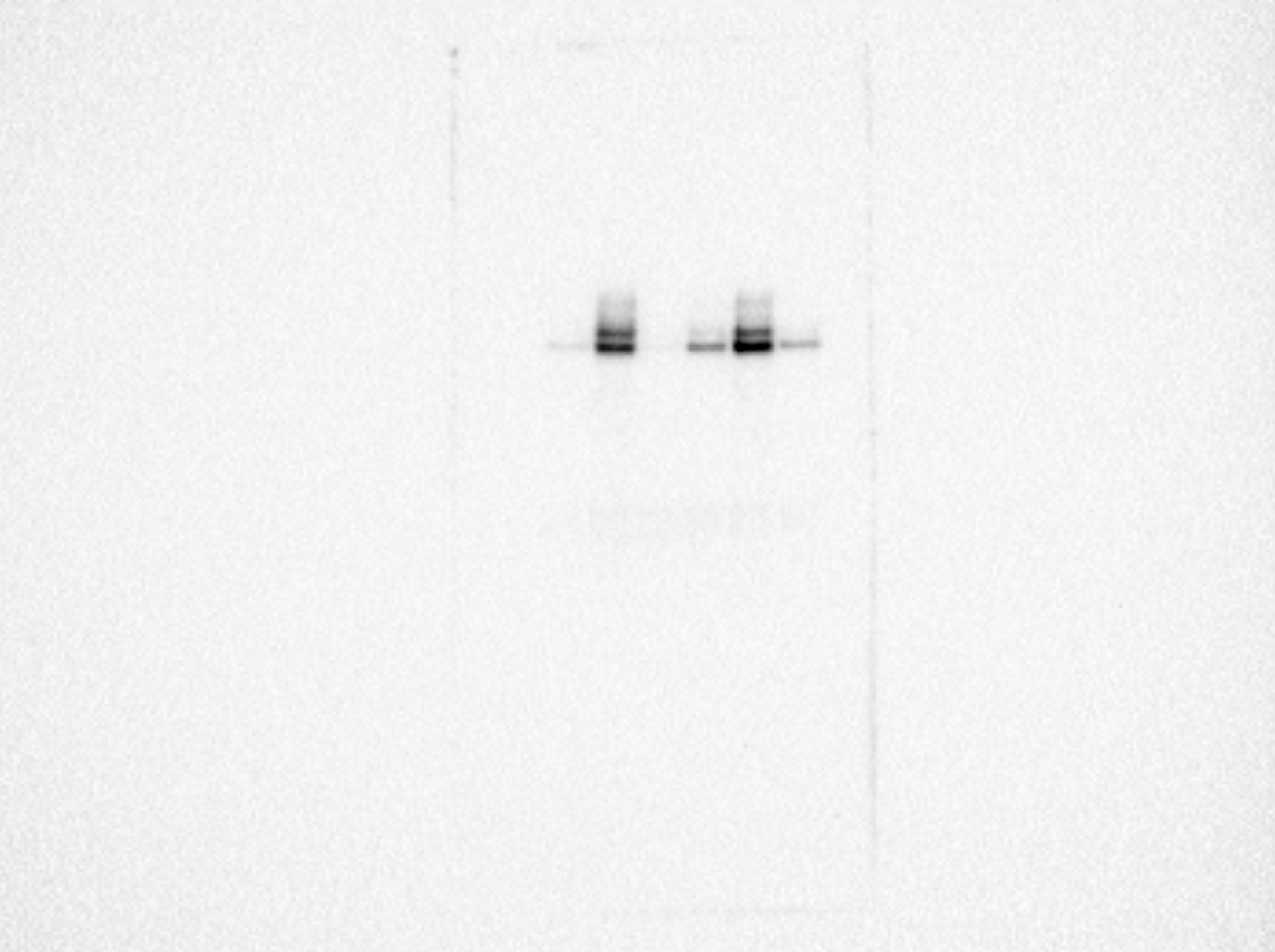

Supplement: Figure 5—source data 11. [file elife-89002-fig5-data11.zip › IP FLAG anti-FLAGm_Exposure_38.3sec.jpg]

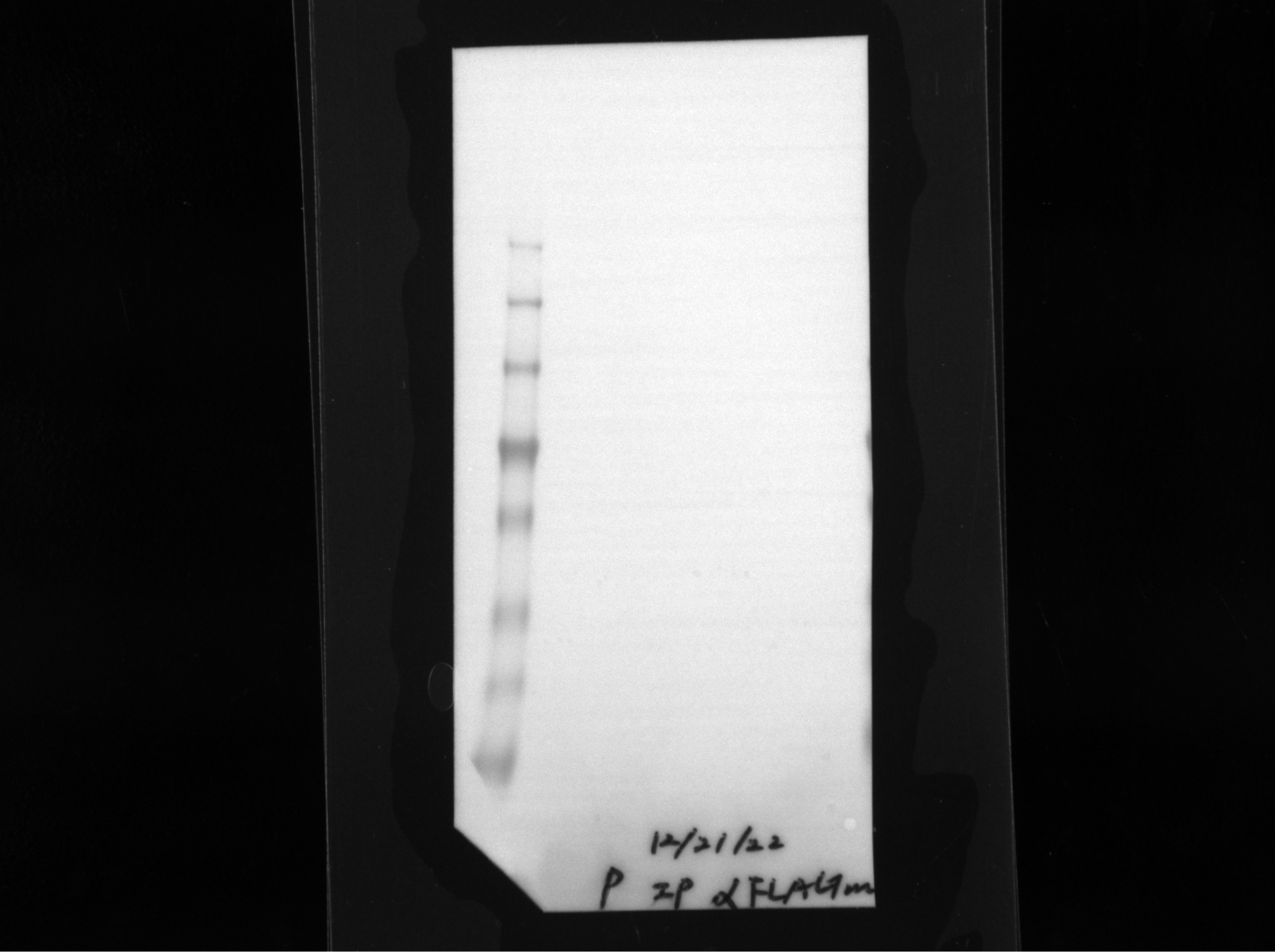

Supplement: Figure 5—source data 11. [file elife-89002-fig5-data11.zip › IP FLAG anti-FLAGm_Marker.jpg]

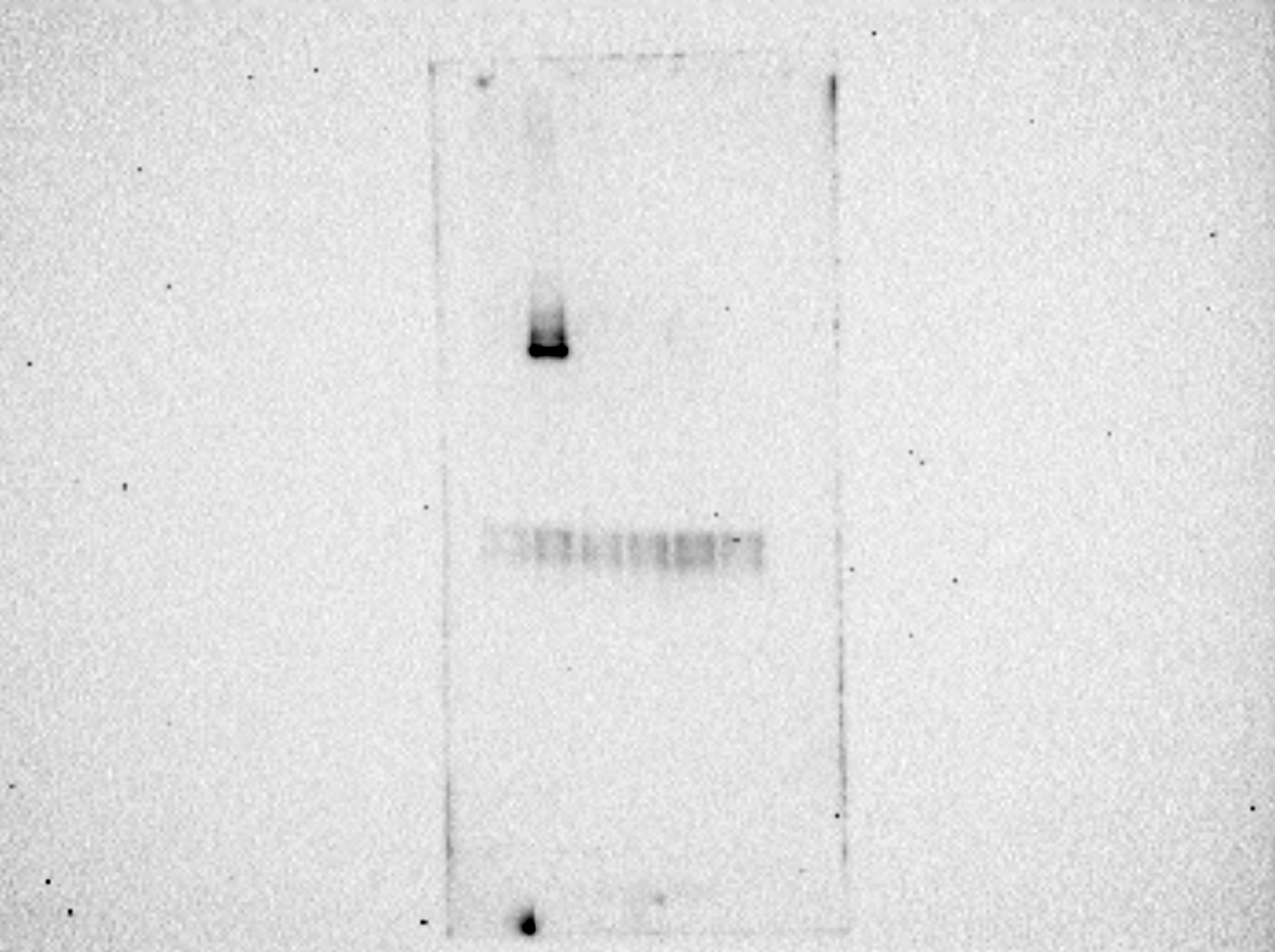

Supplement: Figure 5—source data 11. [file elife-89002-fig5-data11.zip › IP FLAG anti-HAm_Exposure_300.0sec.jpg]

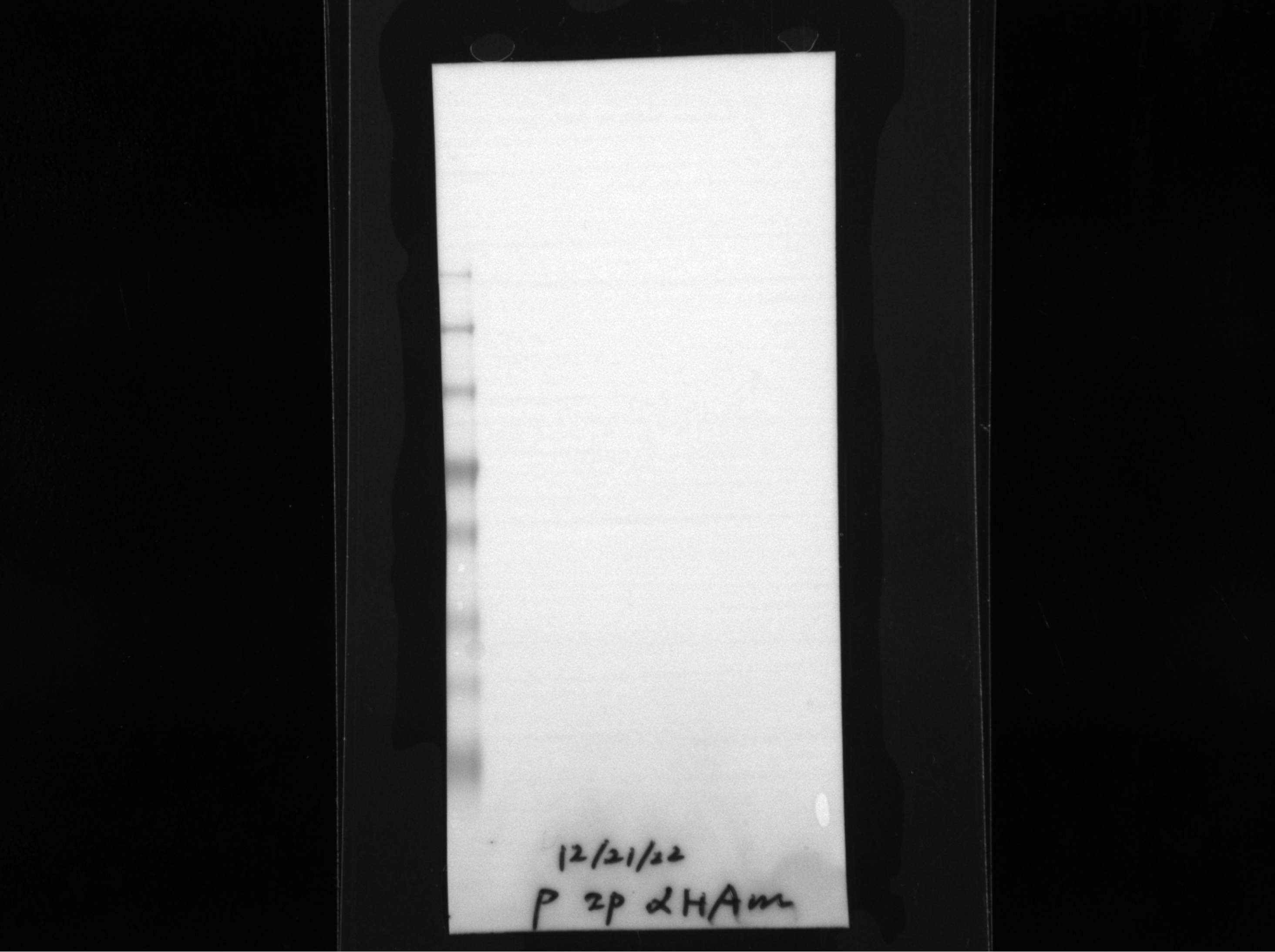

Supplement: Figure 5—source data 11. [file elife-89002-fig5-data11.zip › IP FLAG anti-HAm_Marker.jpg]

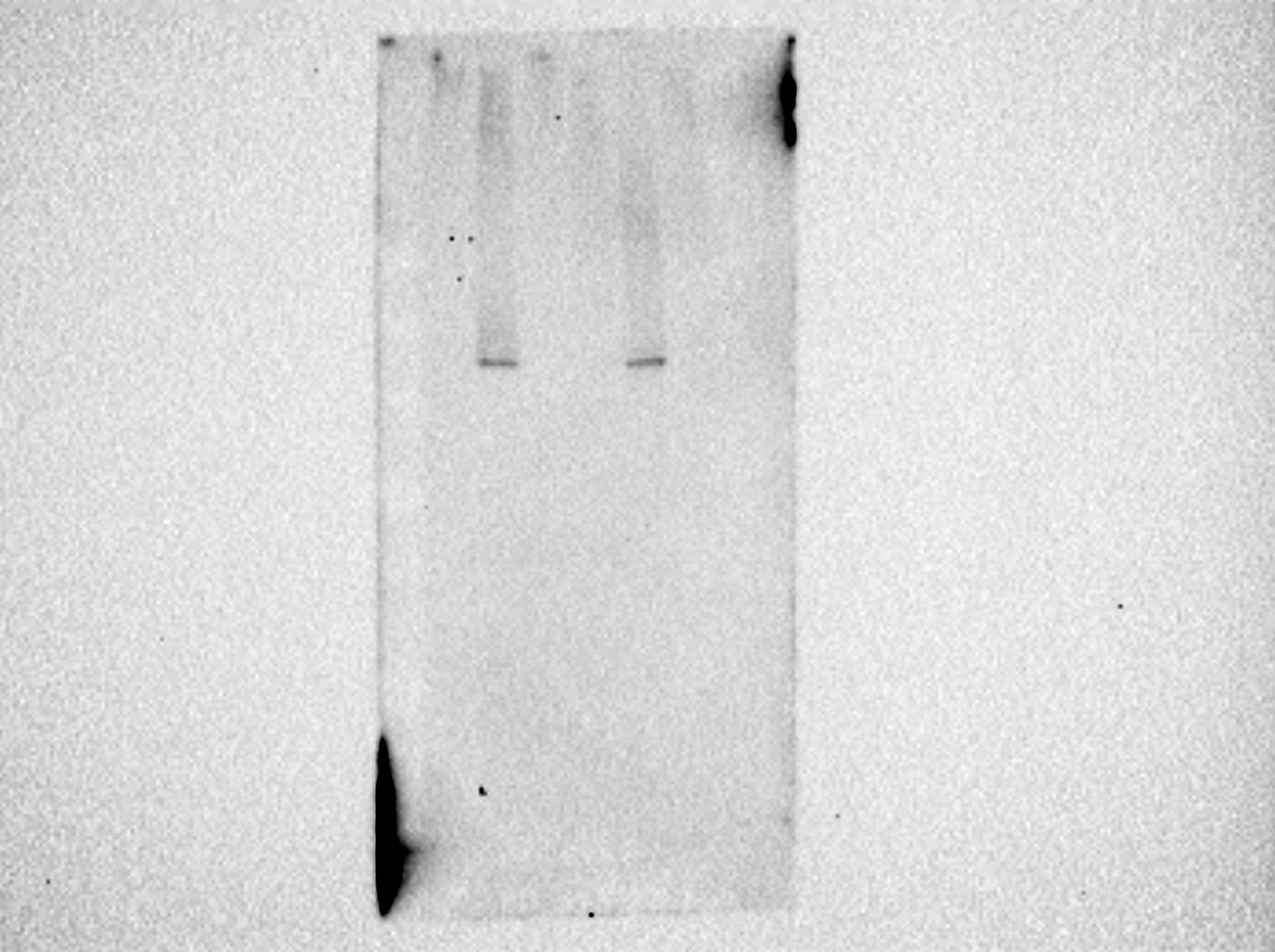

Supplement: Figure 5—source data 11. [file elife-89002-fig5-data11.zip › IP FLAG anti-P4D1m_Exposure_120.0sec.jpg]

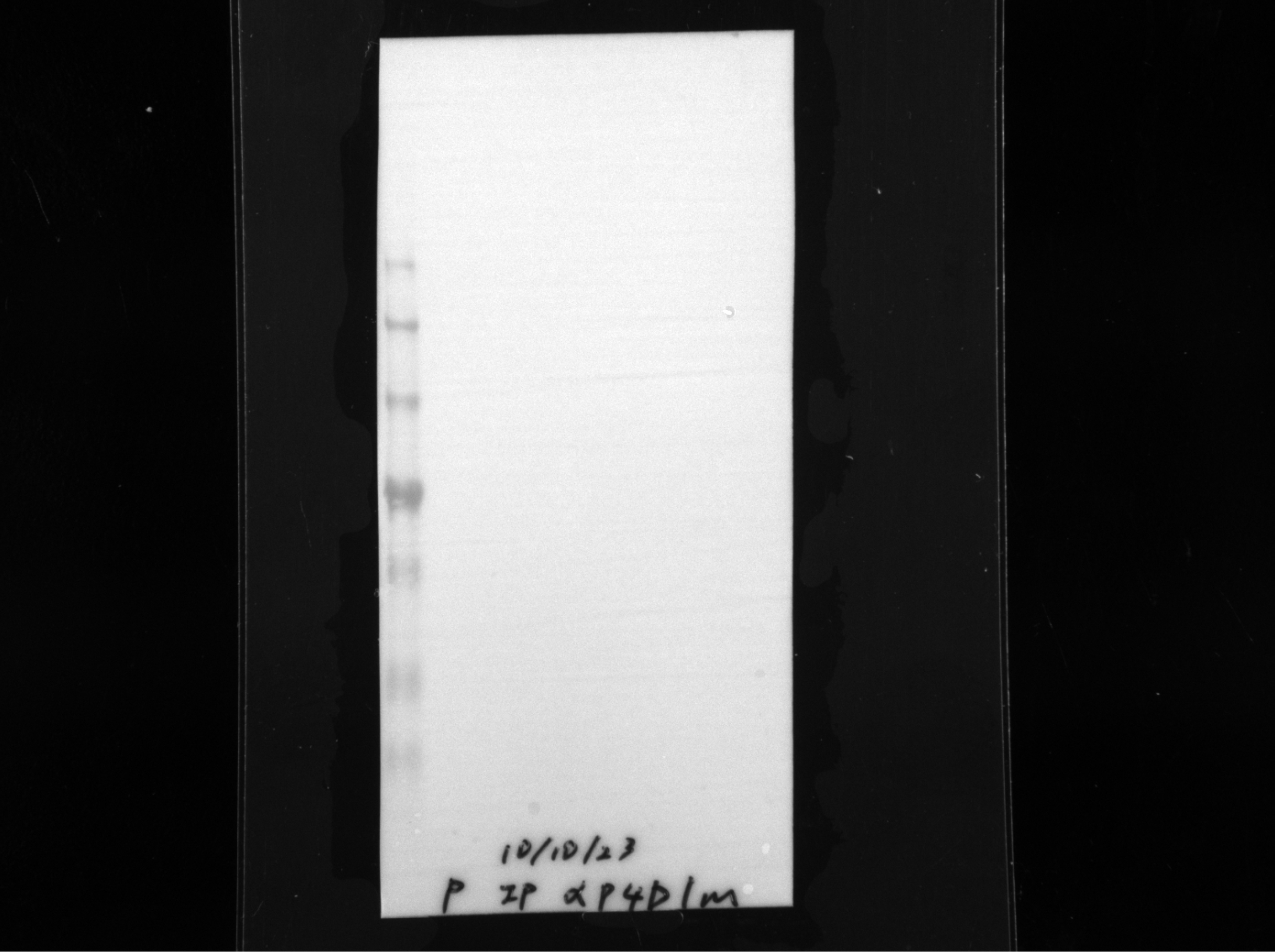

Supplement: Figure 5—source data 11. [file elife-89002-fig5-data11.zip › IP FLAG anti-P4D1m_Marker.jpg]

**f**

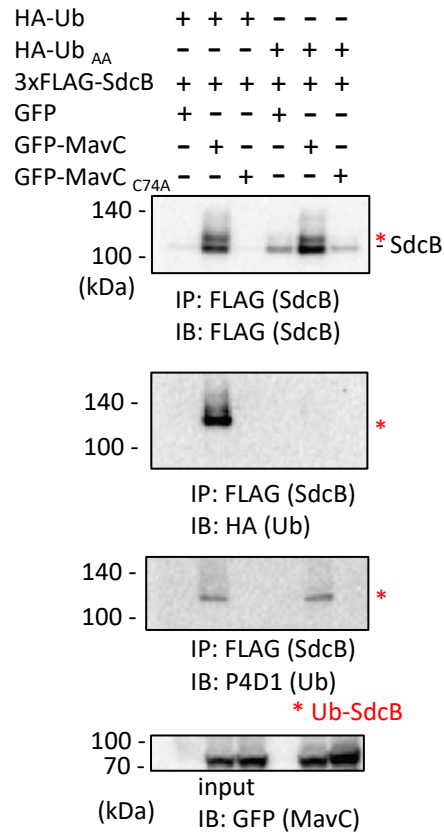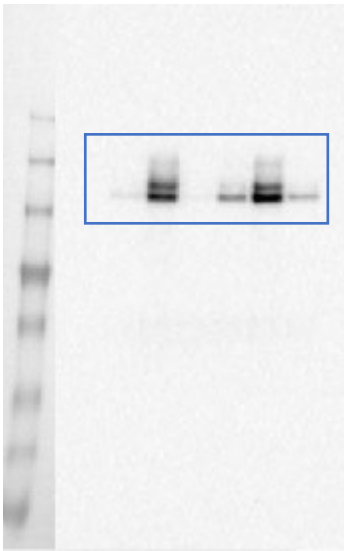

**Figure 5f**  
**top**

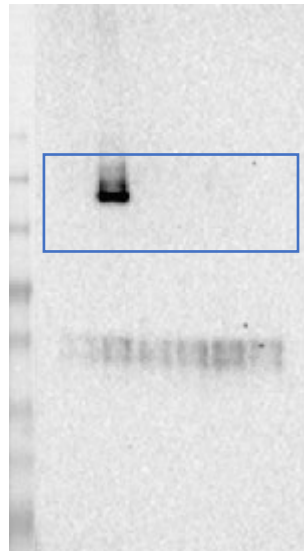

**Figure 5f**  
**2nd**

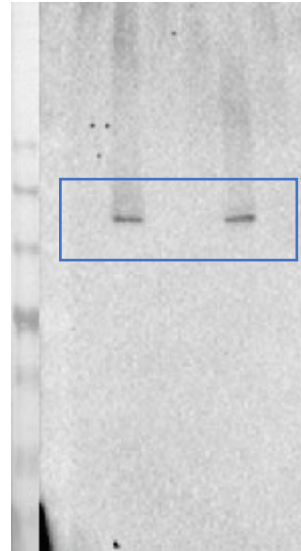

**Figure 5f**  
**3rd**

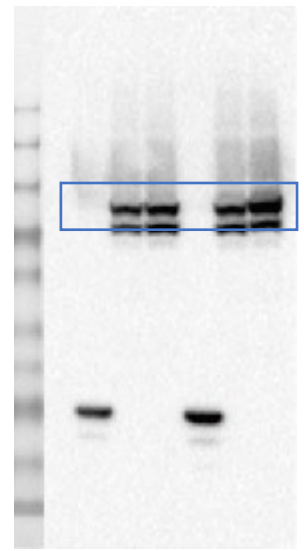

**Figure 5f**  
**bottom**

Supplement: Figure 5—source data 12. [file elife-89002-fig5-data12.pdf]

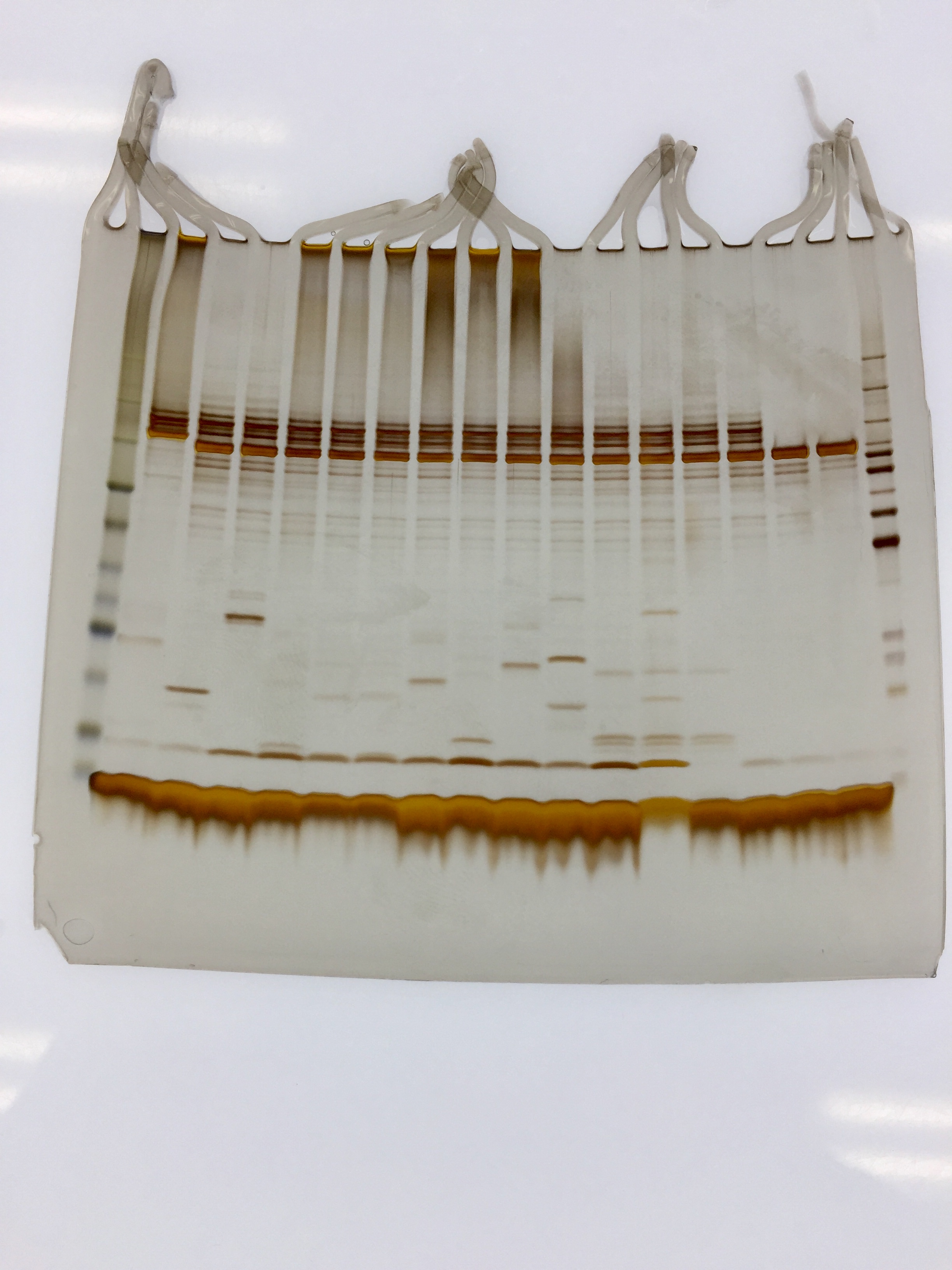

Supplement: Figure 5—figure supplement 1—source data 1. [file elife-89002-fig5-figsupp1-data1.zip › 181121 Ub ligation assay silverstain.jpg]

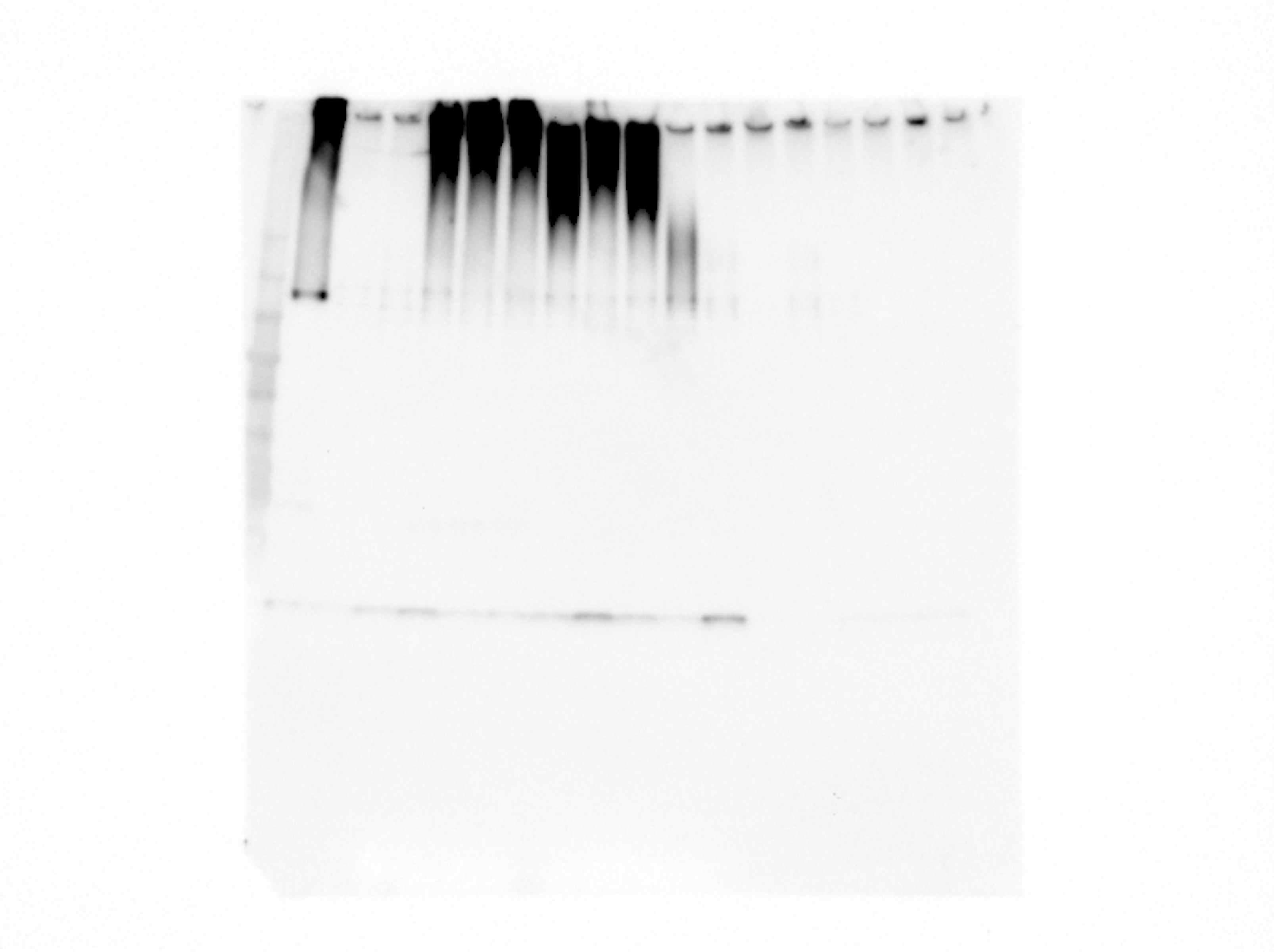

Supplement: Figure 5—figure supplement 1—source data 1. [file elife-89002-fig5-figsupp1-data1.zip › 181121 WB anti-FK2 Exposure_86.0sec.jpg]

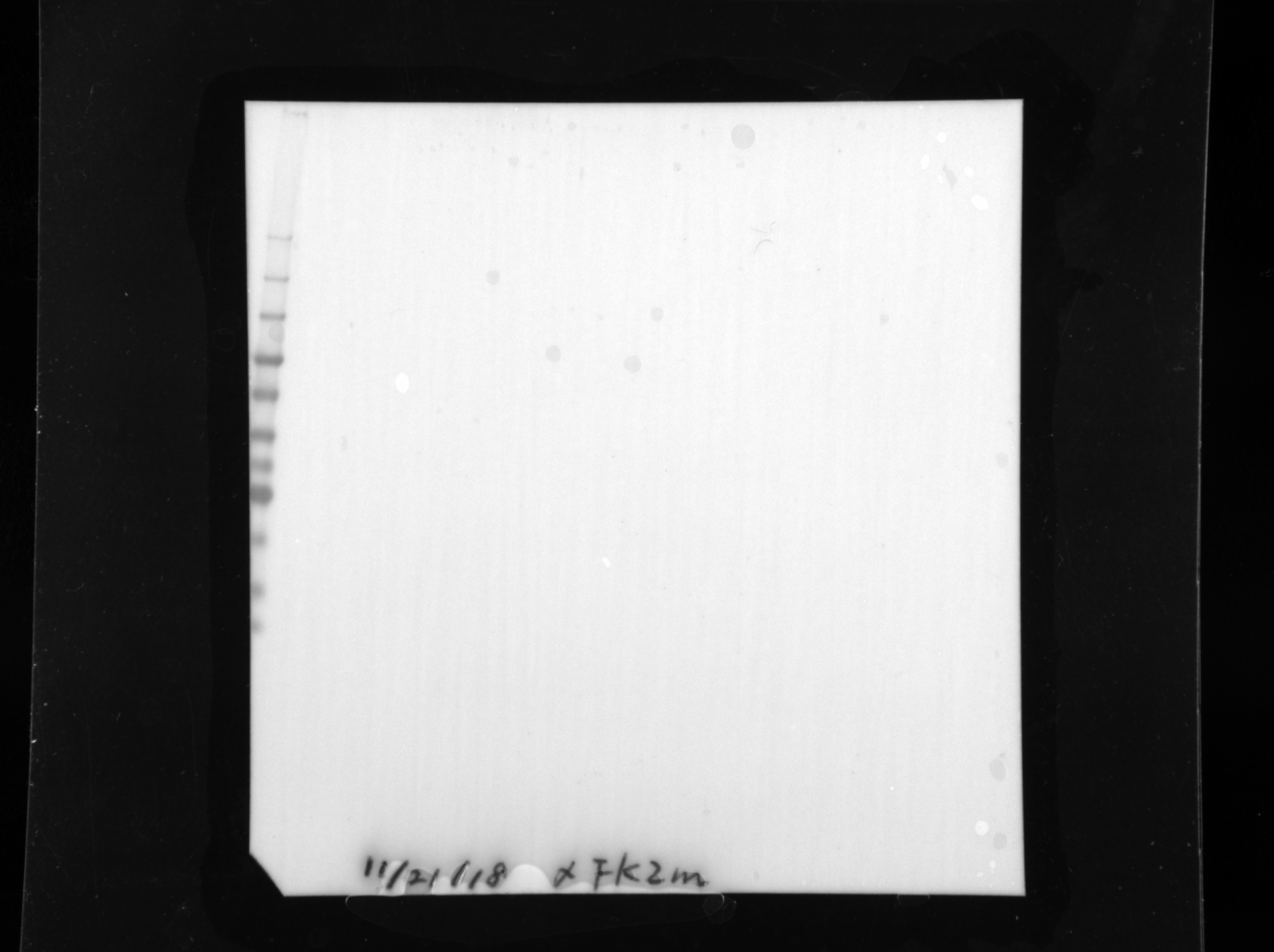

Supplement: Figure 5—figure supplement 1—source data 1. [file elife-89002-fig5-figsupp1-data1.zip › Marker anti-FK2.jpg]

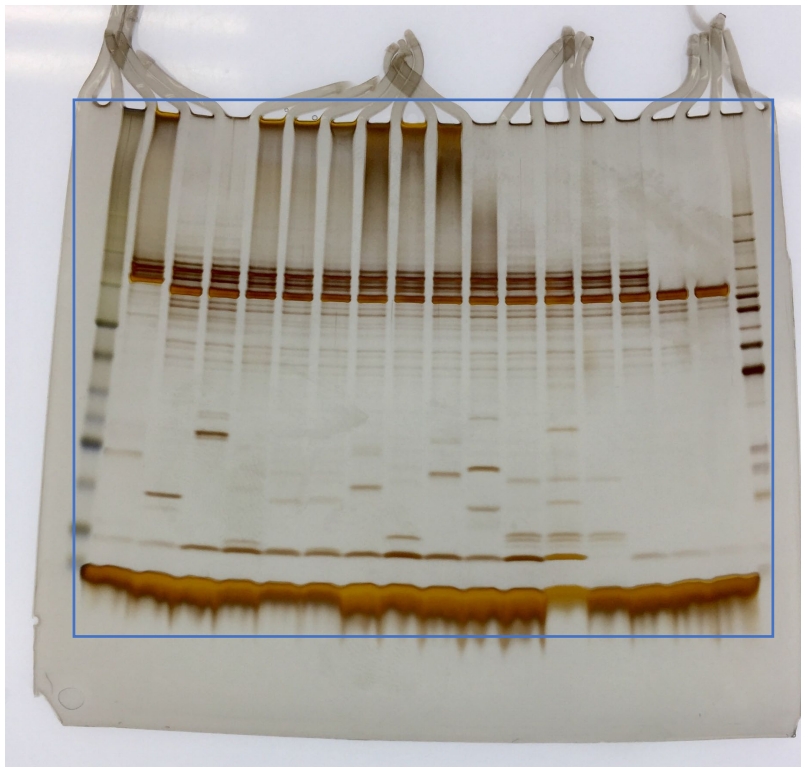

**Figure 5– figure supplement 1a**  
(silverstaining)

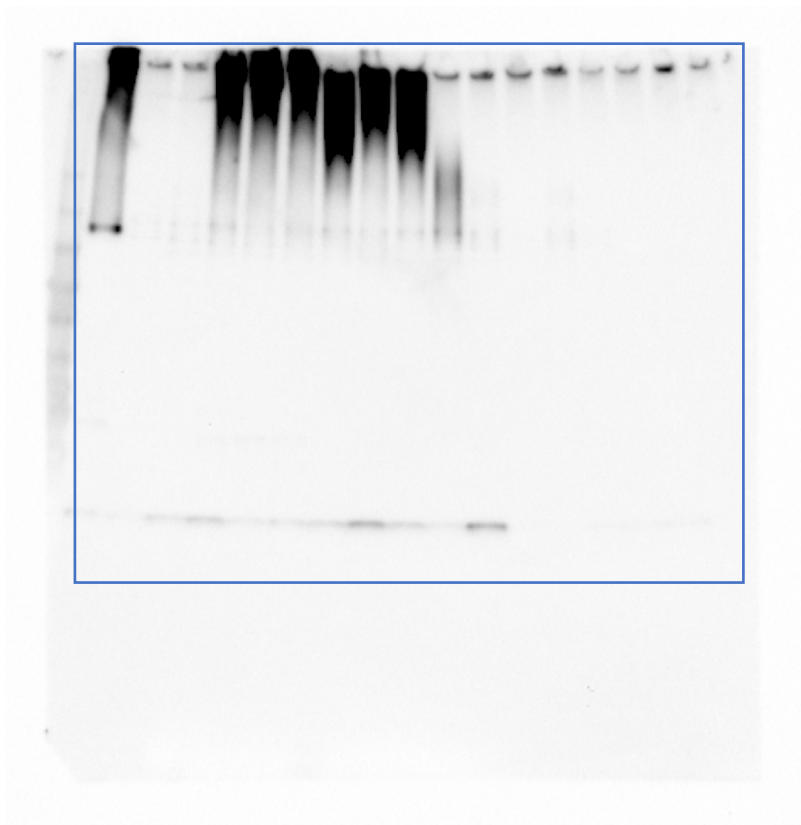

**Figure 5– figure supplement 1b**  
(anti-FK2)

Supplement: Figure 5—figure supplement 1—source data 2. [file elife-89002-fig5-figsupp1-data2.pdf]

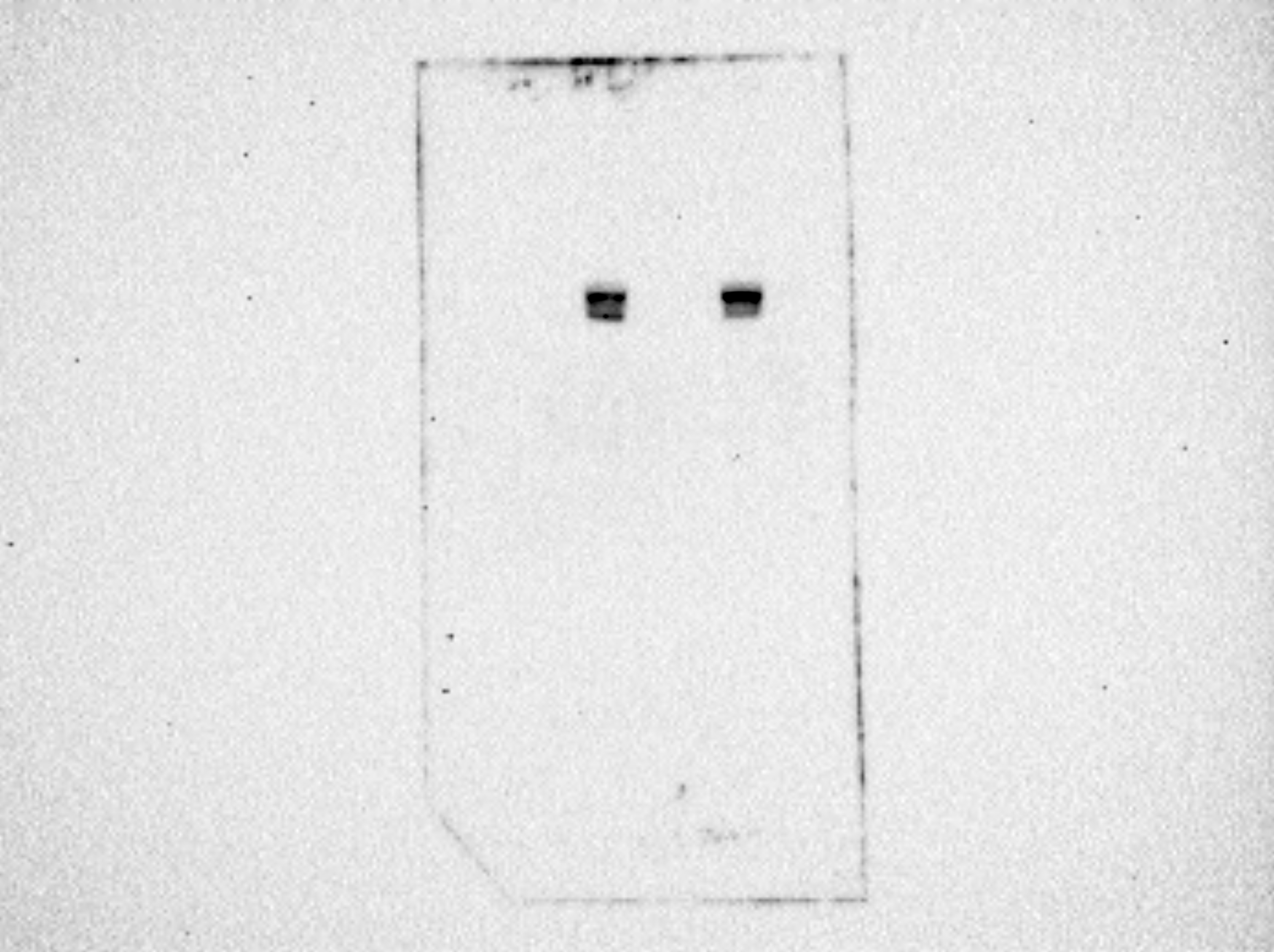

Supplement: Figure 5—figure supplement 2—source data 1. [file elife-89002-fig5-figsupp2-data1.zip › anti-FLAGm_Exposure_300.0sec.jpg]

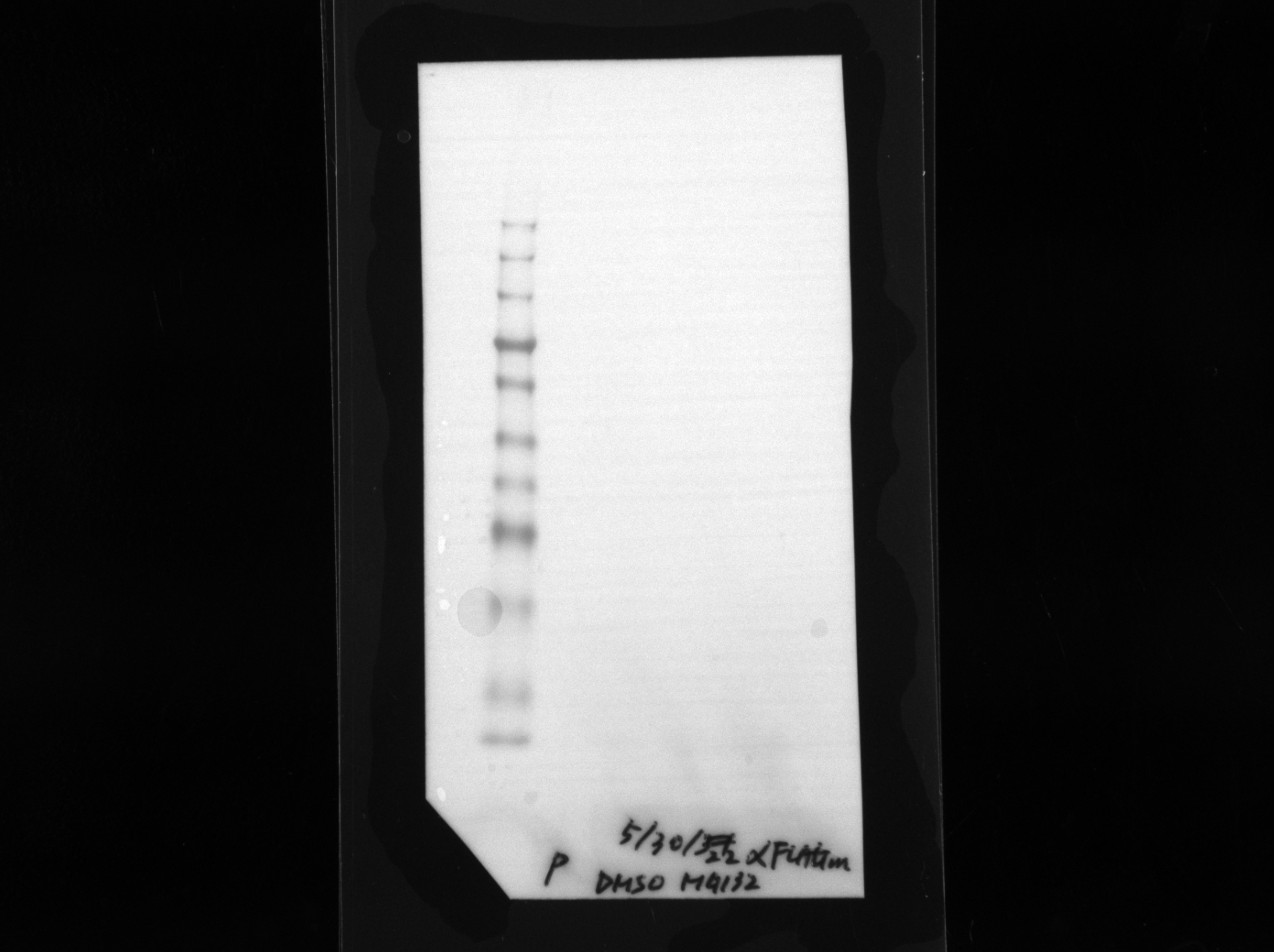

Supplement: Figure 5—figure supplement 2—source data 1. [file elife-89002-fig5-figsupp2-data1.zip › anti-FLAGm_Marker.jpg]

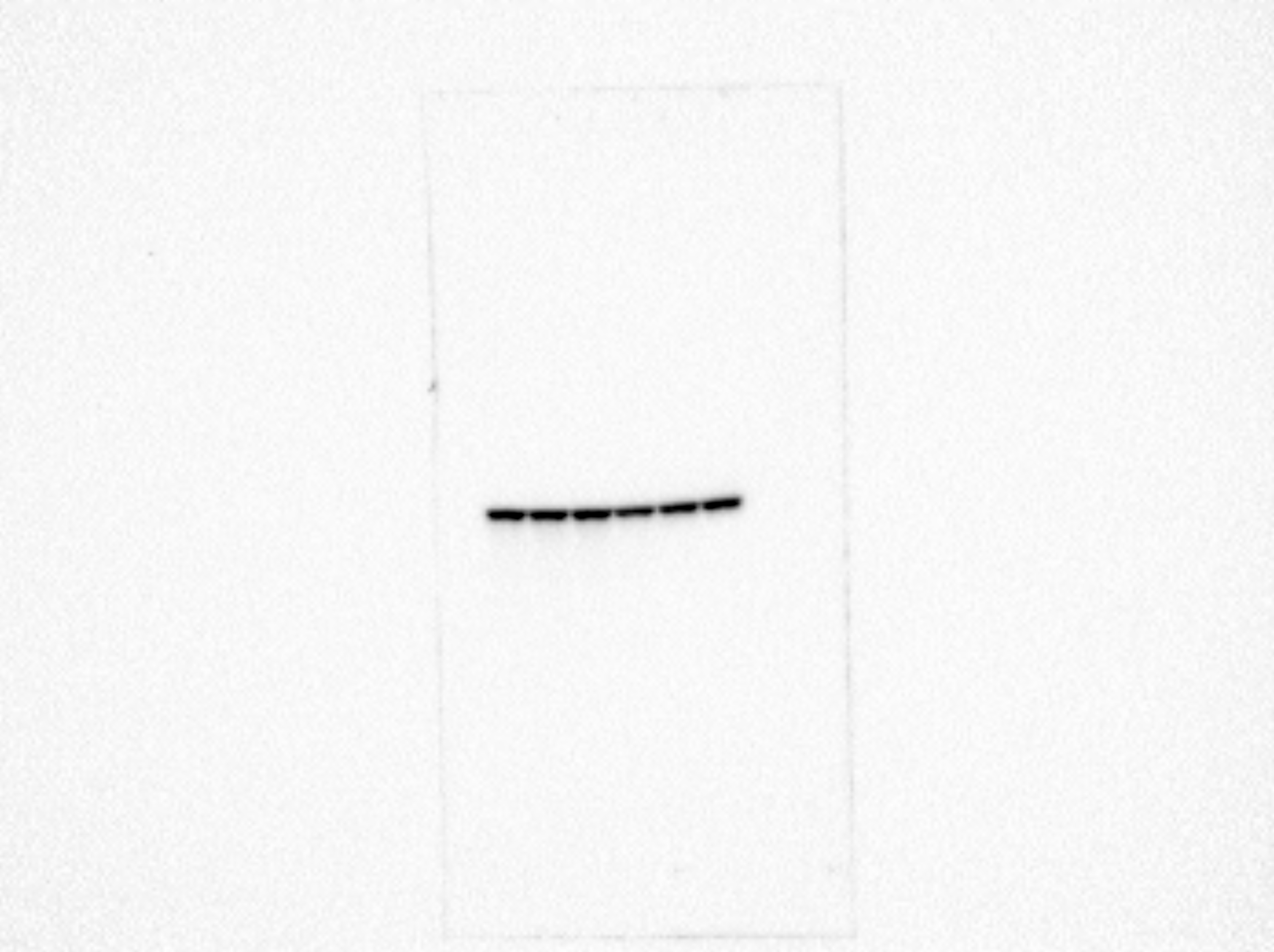

Supplement: Figure 5—figure supplement 2—source data 1. [file elife-89002-fig5-figsupp2-data1.zip › anti-GAPDH_Exposure_13.4sec.jpg]

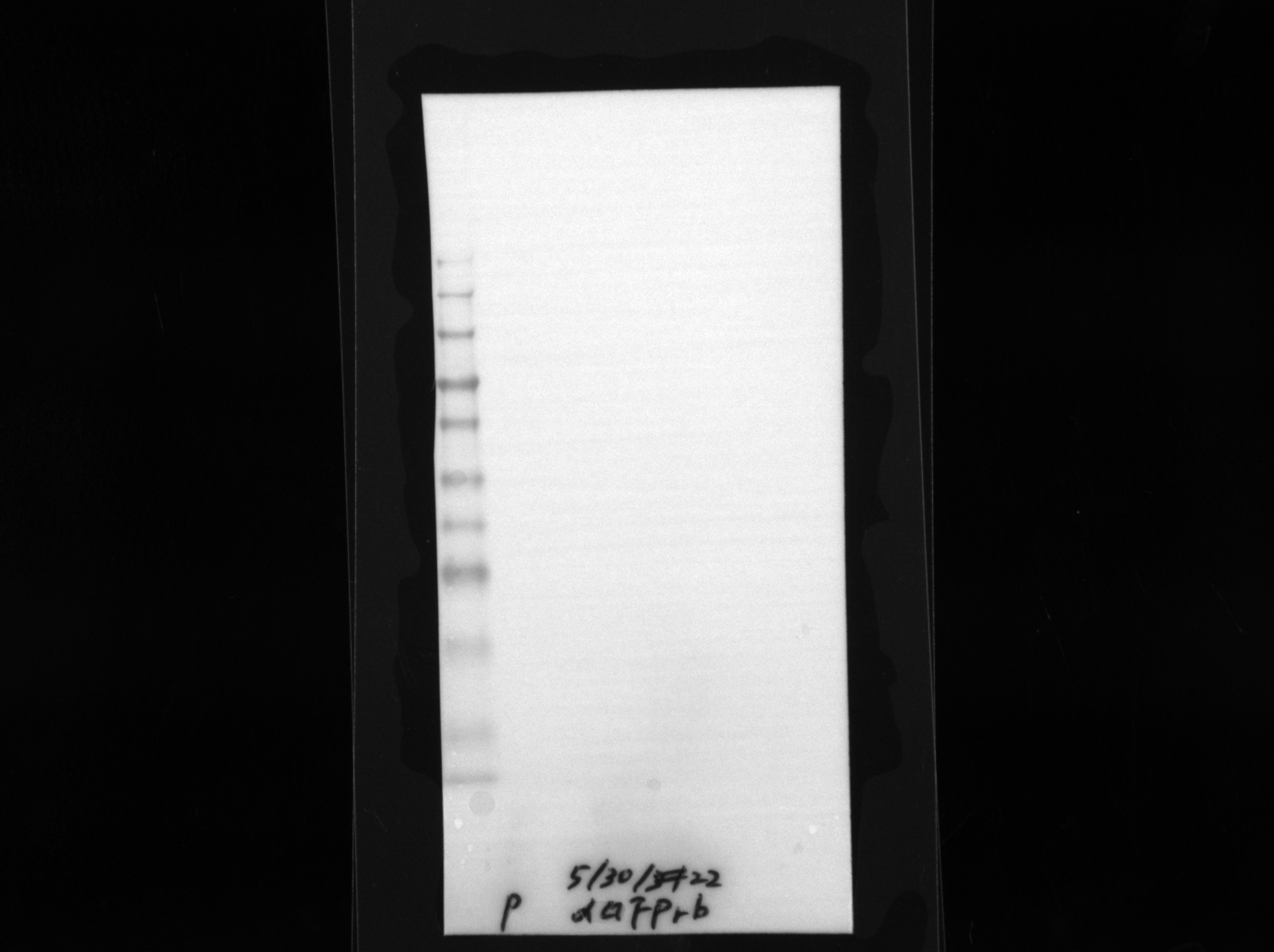

Supplement: Figure 5—figure supplement 2—source data 1. [file elife-89002-fig5-figsupp2-data1.zip › anti-GAPDH_Marker.jpg]

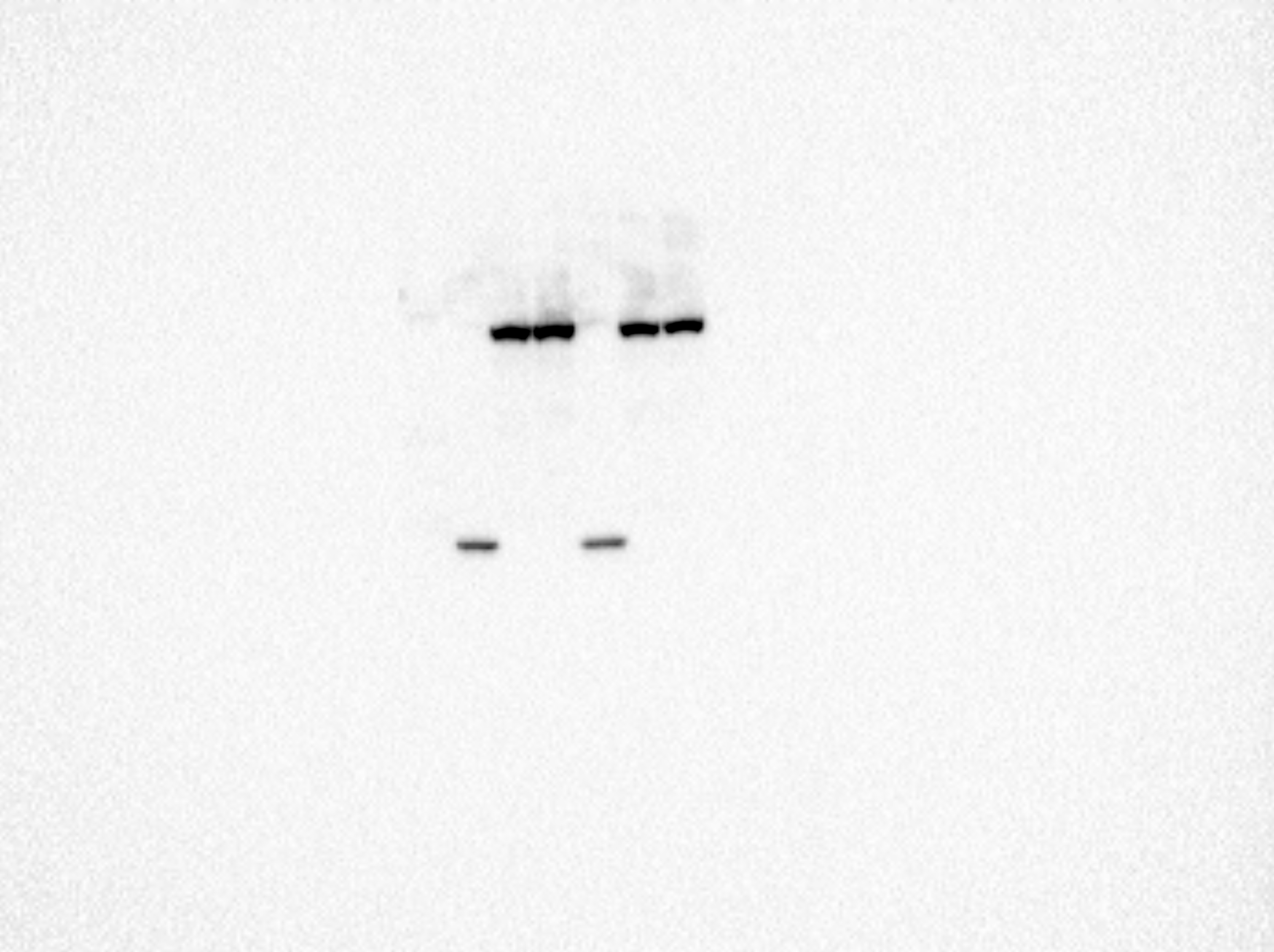

Supplement: Figure 5—figure supplement 2—source data 1. [file elife-89002-fig5-figsupp2-data1.zip › anti-GFP_Exposure_13.4sec.jpg]

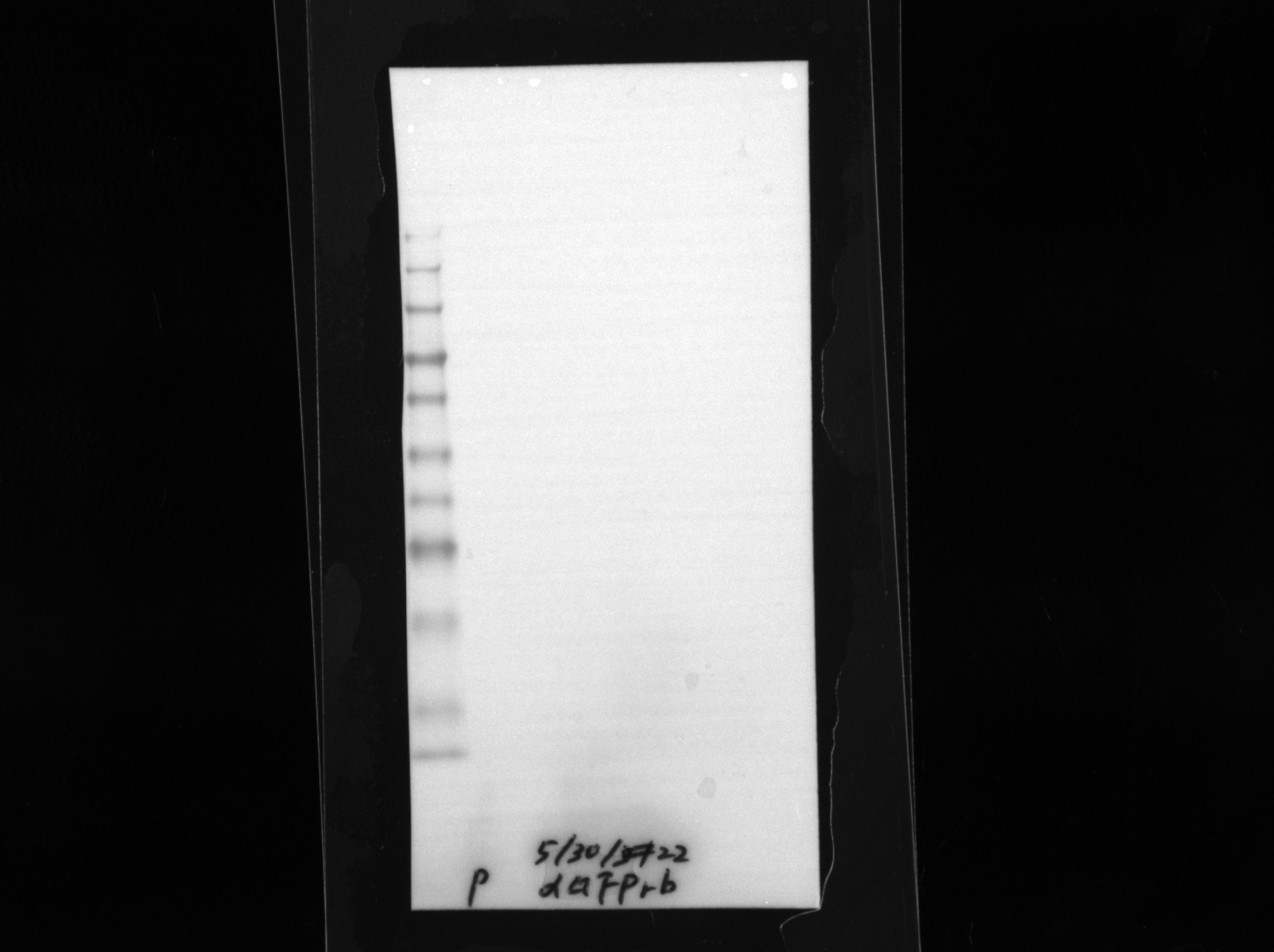

Supplement: Figure 5—figure supplement 2—source data 1. [file elife-89002-fig5-figsupp2-data1.zip › anti-GFP_Marker.jpg]

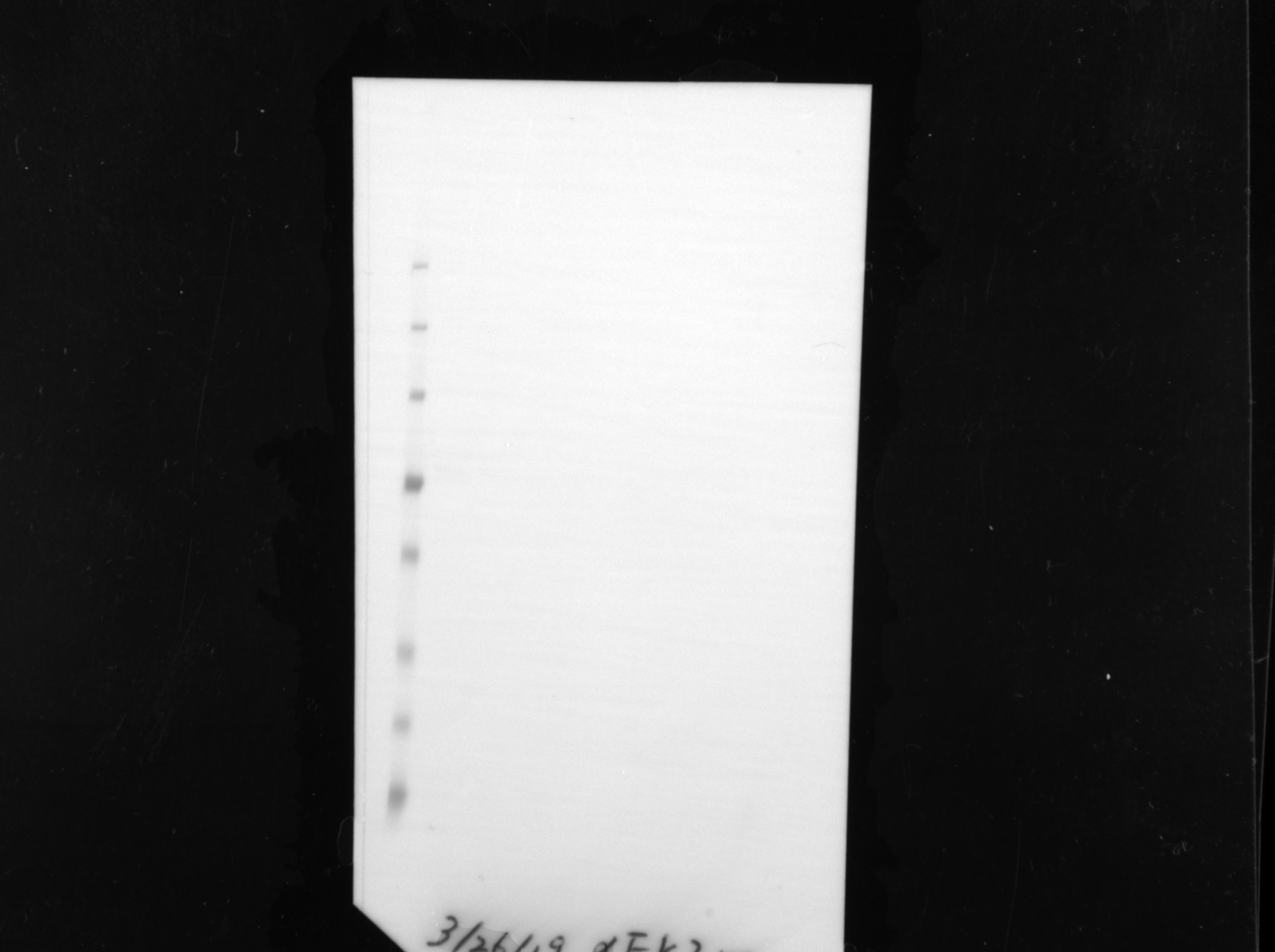

Supplement: Figure 5—figure supplement 2—source data 3. [file elife-89002-fig5-figsupp2-data3.zip › anti-FK2 ubiquitination assay.jpg]

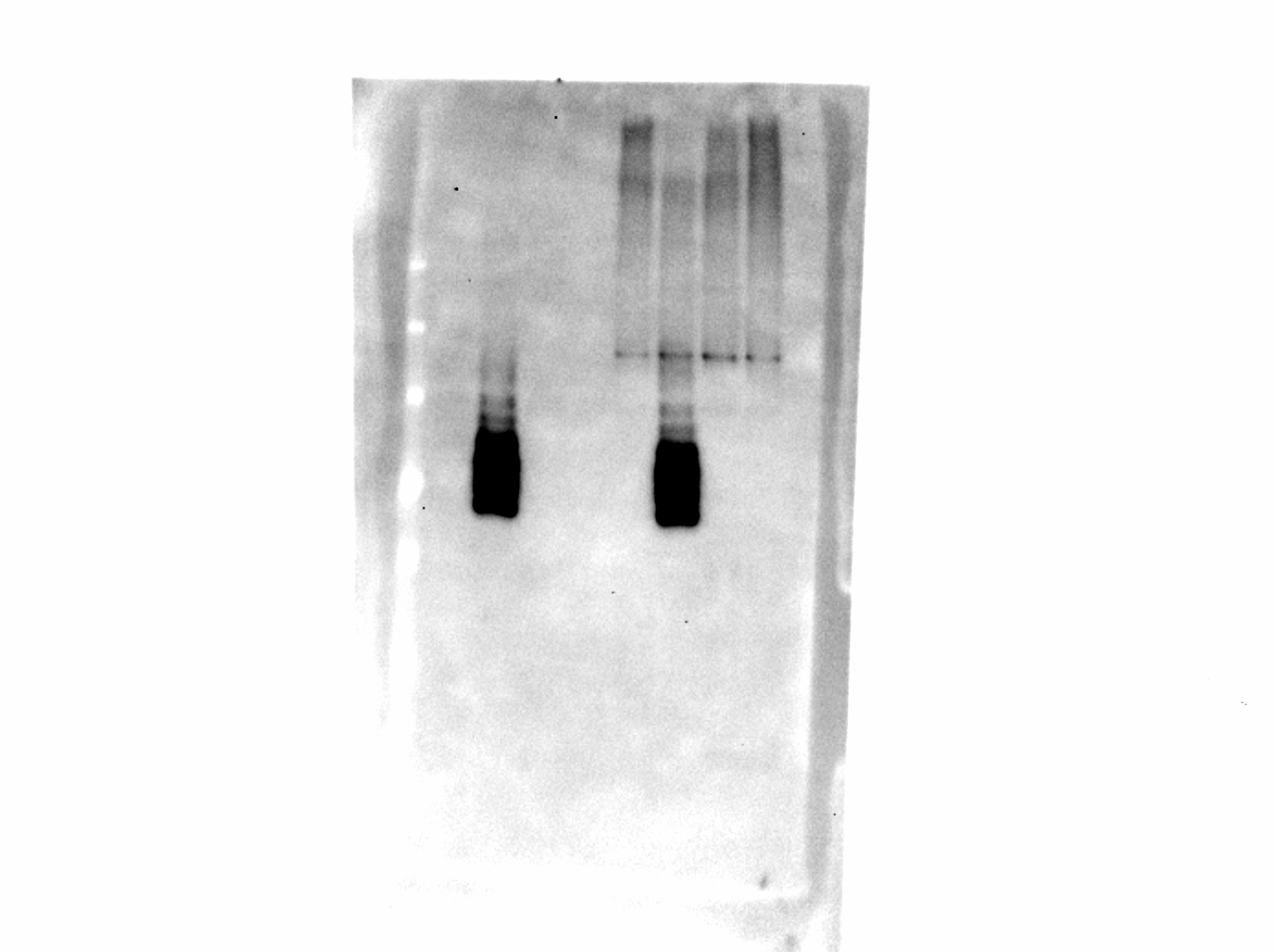

Supplement: Figure 5—figure supplement 2—source data 3. [file elife-89002-fig5-figsupp2-data3.zip › anti-FK2 ubiquitination assay_Exposure_240.0sec.jpg]

**b**

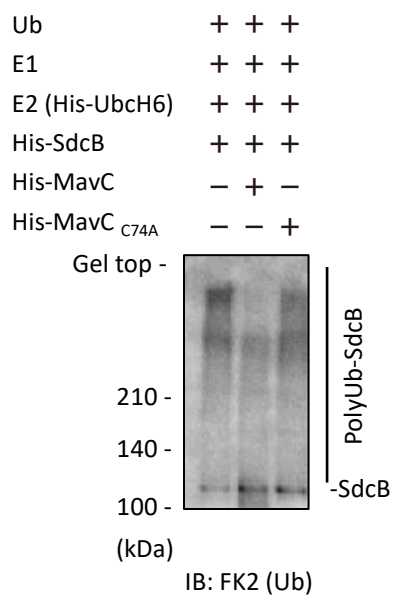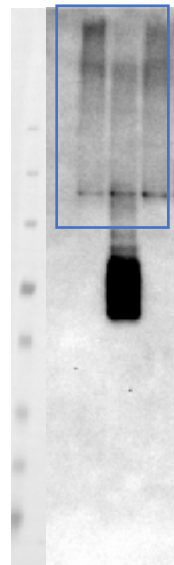

**Figure 5**  
– figure supplement 2b

Supplement: Figure 5—figure supplement 2—source data 4. [file elife-89002-fig5-figsupp2-data4.pdf]

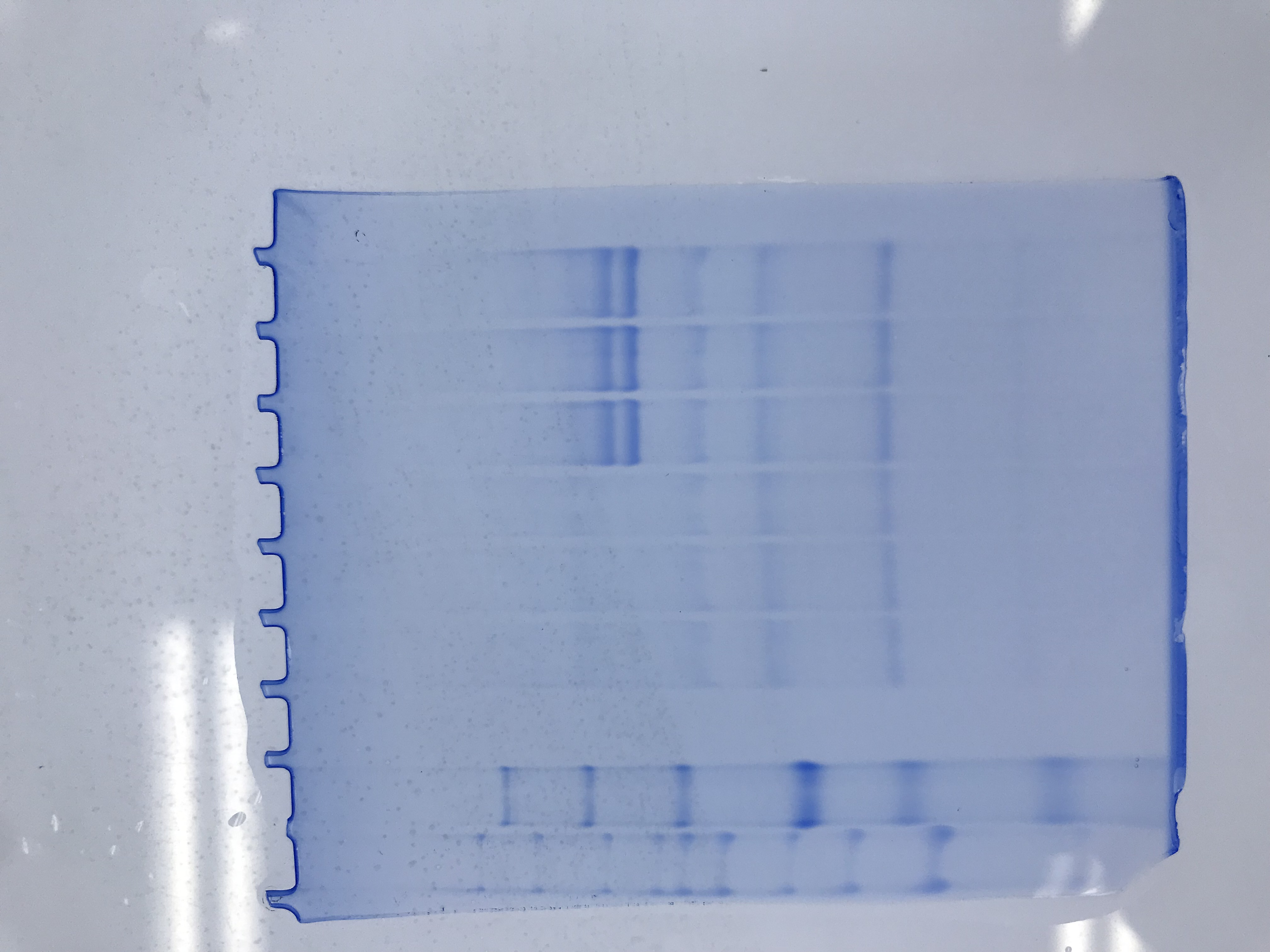

Supplement: Figure 6—source data 1. [file elife-89002-fig6-data1.zip › CBB staining for MS.jpg]

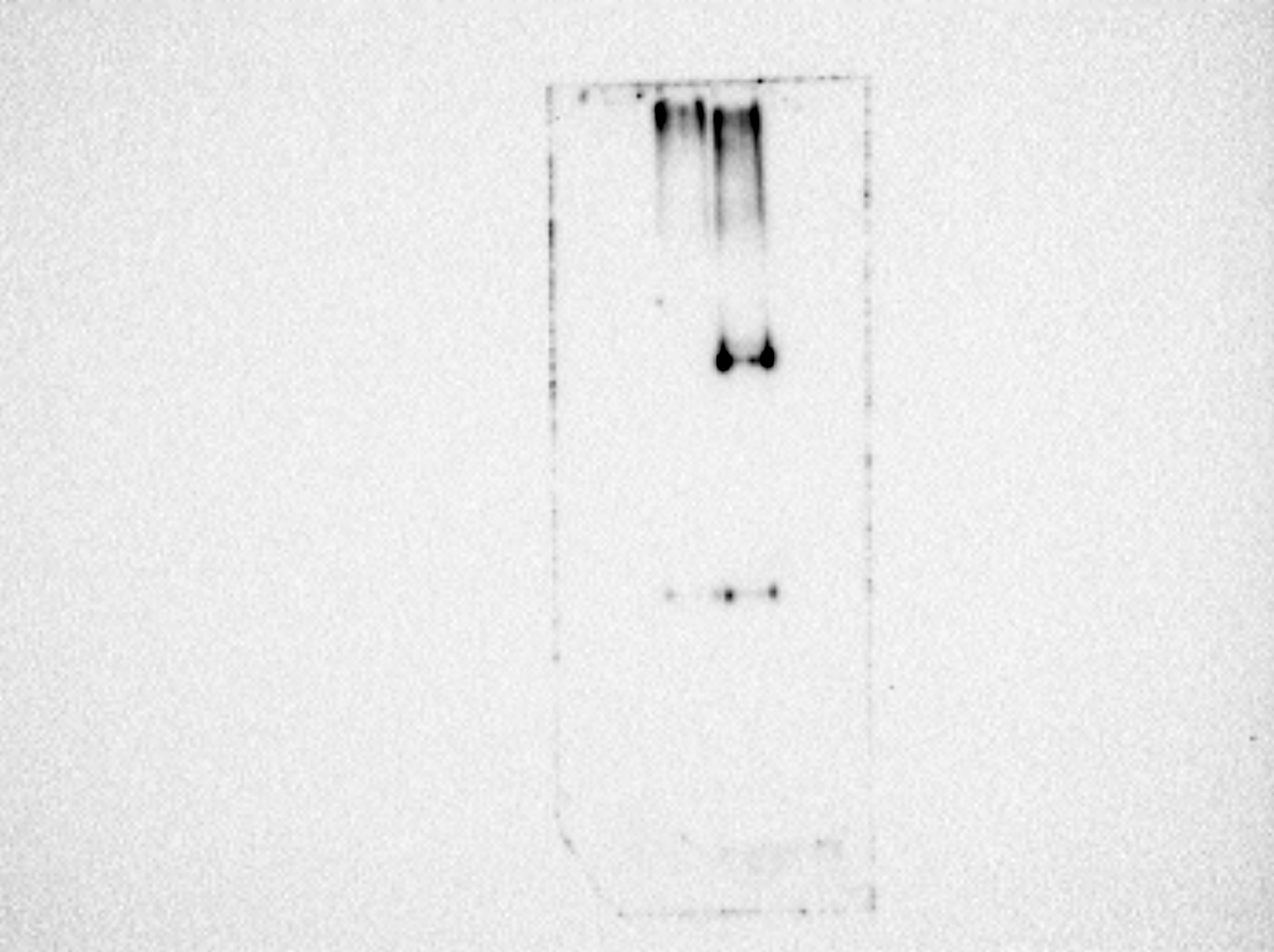

Supplement: Figure 6—source data 1. [file elife-89002-fig6-data1.zip › IP FLAG IB HAm super_Exposure_20.0sec.jpg]

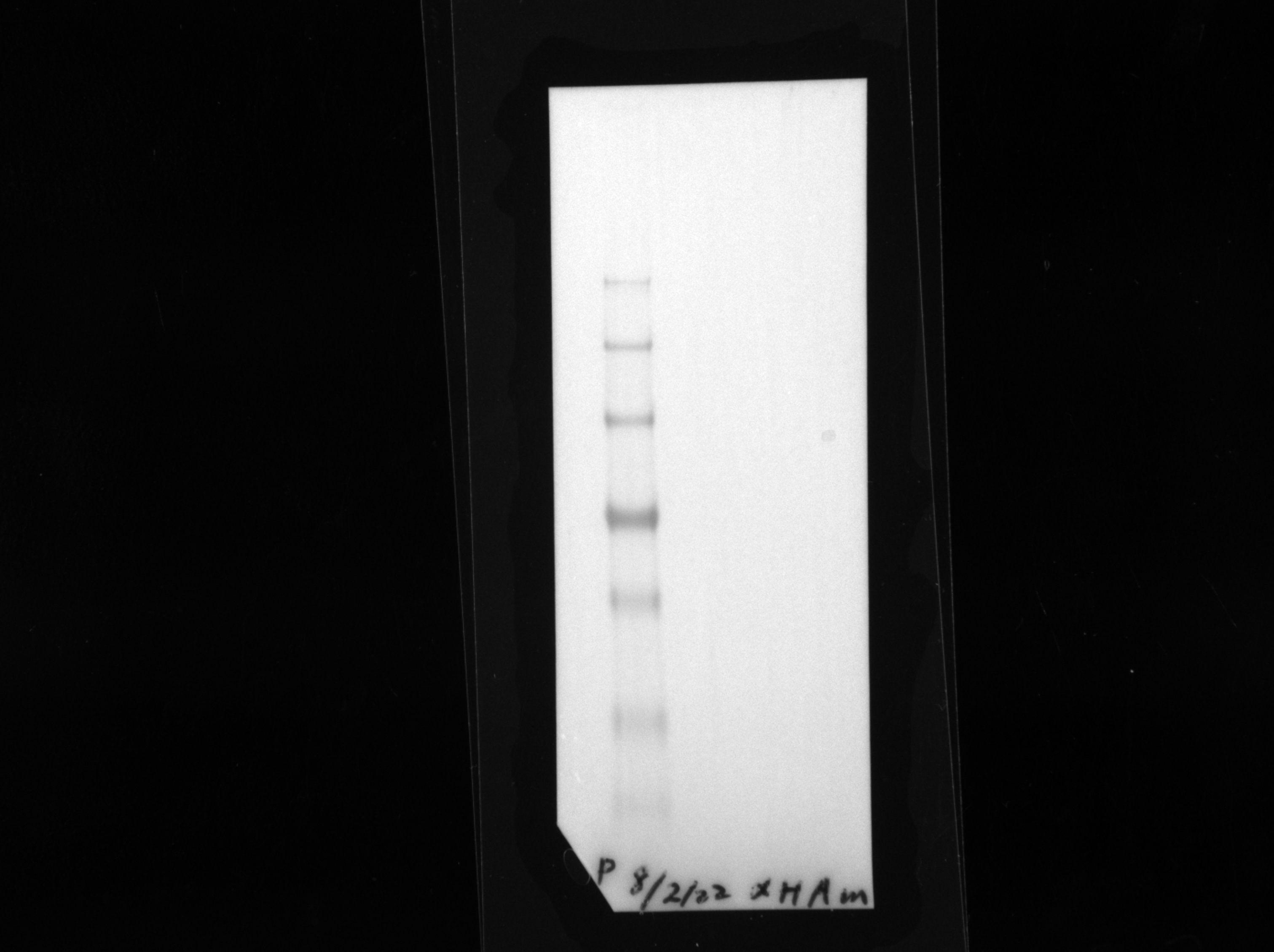

Supplement: Figure 6—source data 1. [file elife-89002-fig6-data1.zip › IP FLAG IB HAm super_Marker.jpg]

**a**

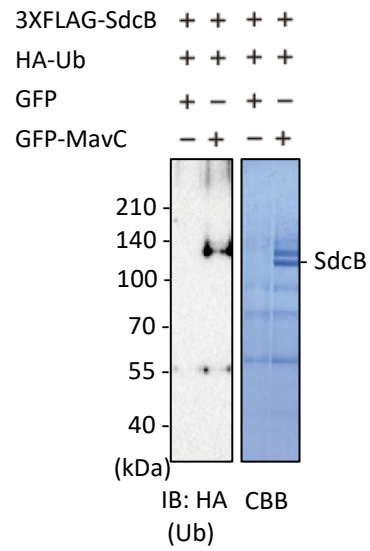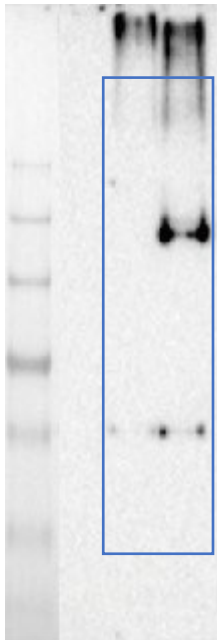

**Figure 6a  
left**

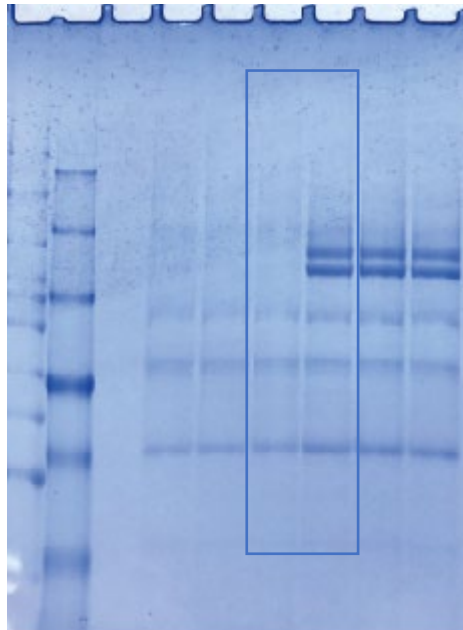

**Figure 6a  
right**

Supplement: Figure 6—source data 2. [file elife-89002-fig6-data2.pdf]

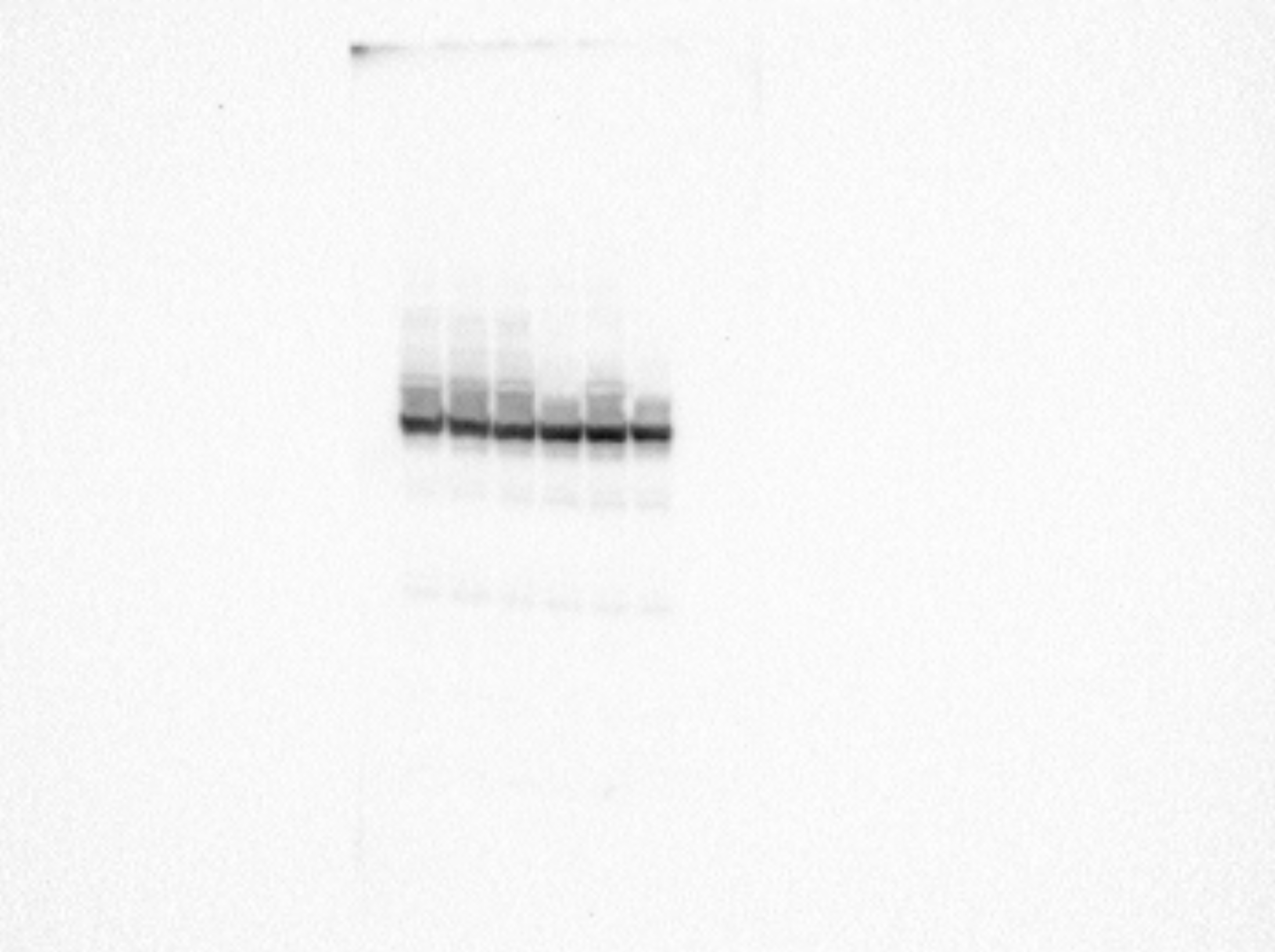

Supplement: Figure 6—source data 3. [file elife-89002-fig6-data3.zip › input anti-GFPrb_Exposure_16.5sec.jpg]

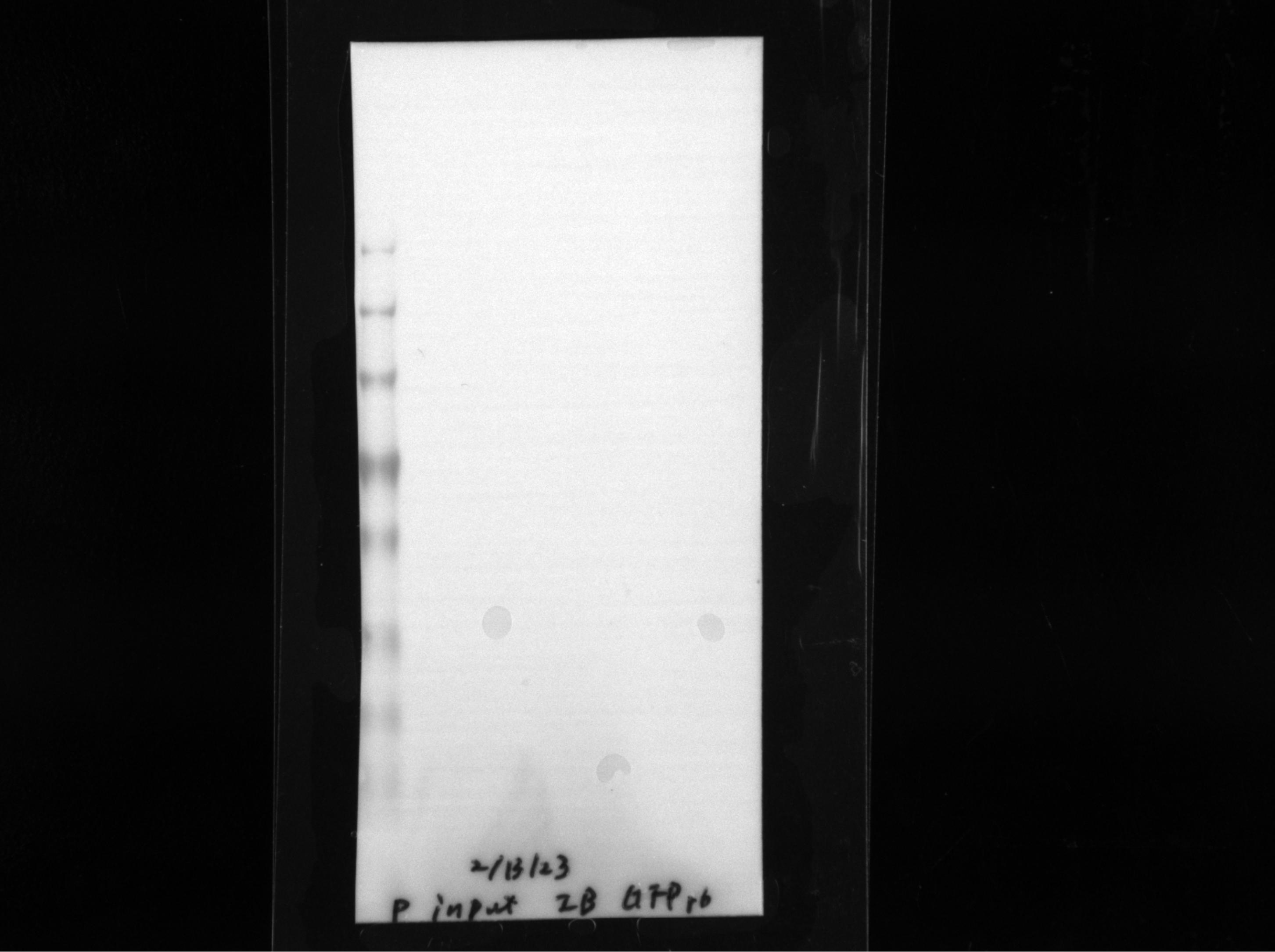

Supplement: Figure 6—source data 3. [file elife-89002-fig6-data3.zip › input anti-GFPrb_Marker.jpg]

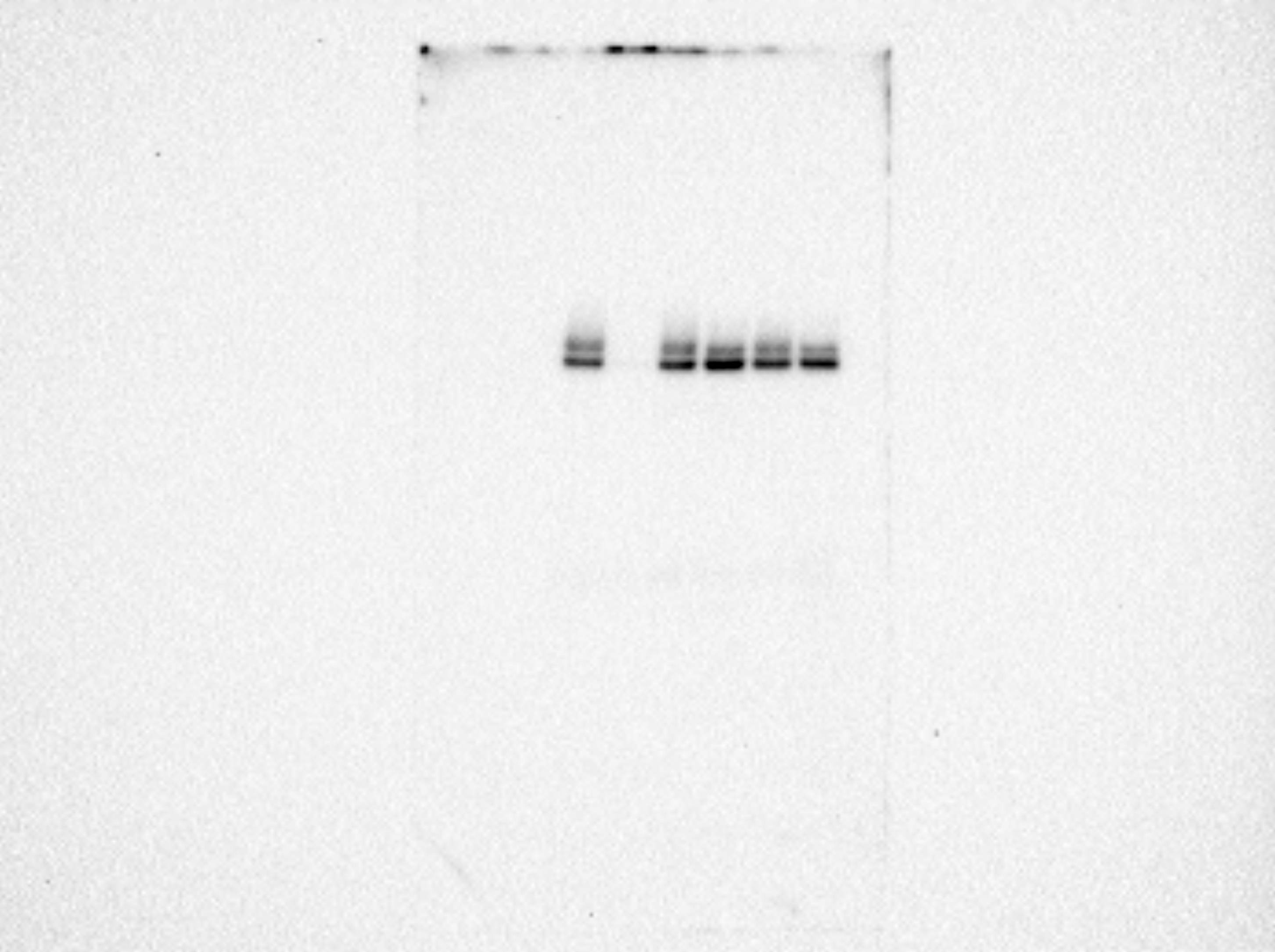

Supplement: Figure 6—source data 3. [file elife-89002-fig6-data3.zip › IP FLAG anti-FLAGm_Exposure_41.4sec.jpg]

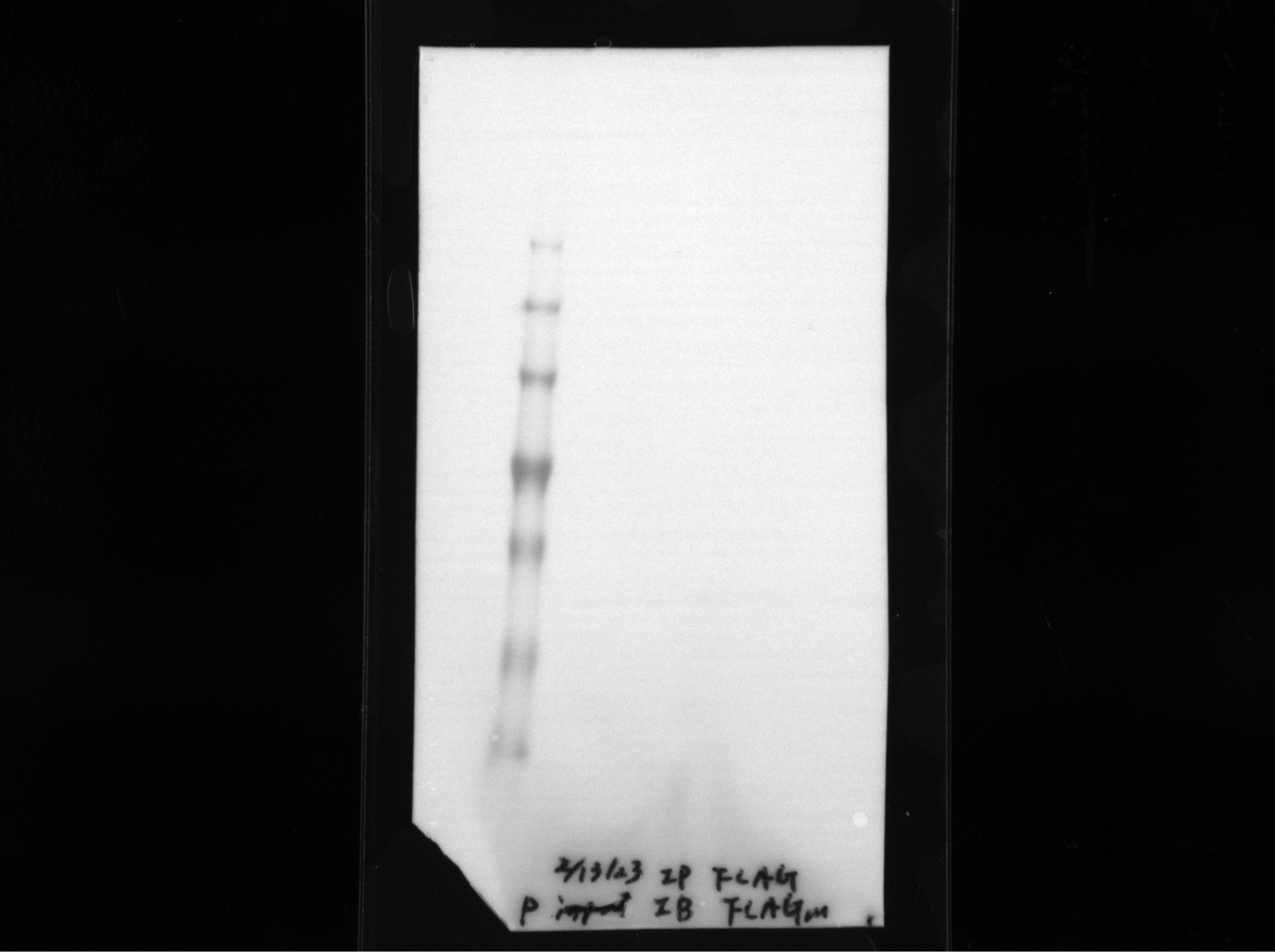

Supplement: Figure 6—source data 3. [file elife-89002-fig6-data3.zip › IP FLAG anti-FLAGm_Marker.jpg]

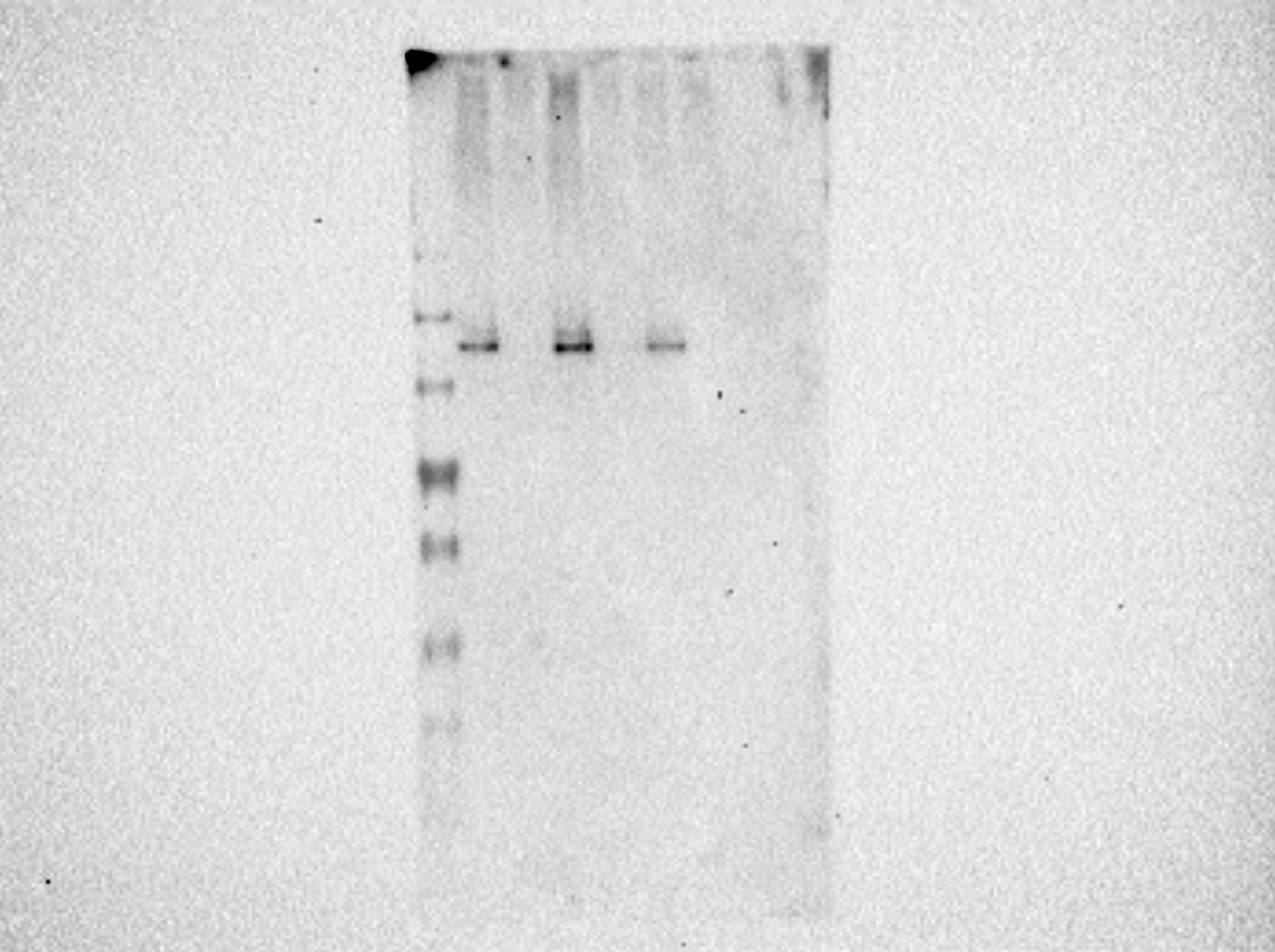

Supplement: Figure 6—source data 3. [file elife-89002-fig6-data3.zip › IP FLAG anti-HArb_Exposure_120.0sec.jpg]

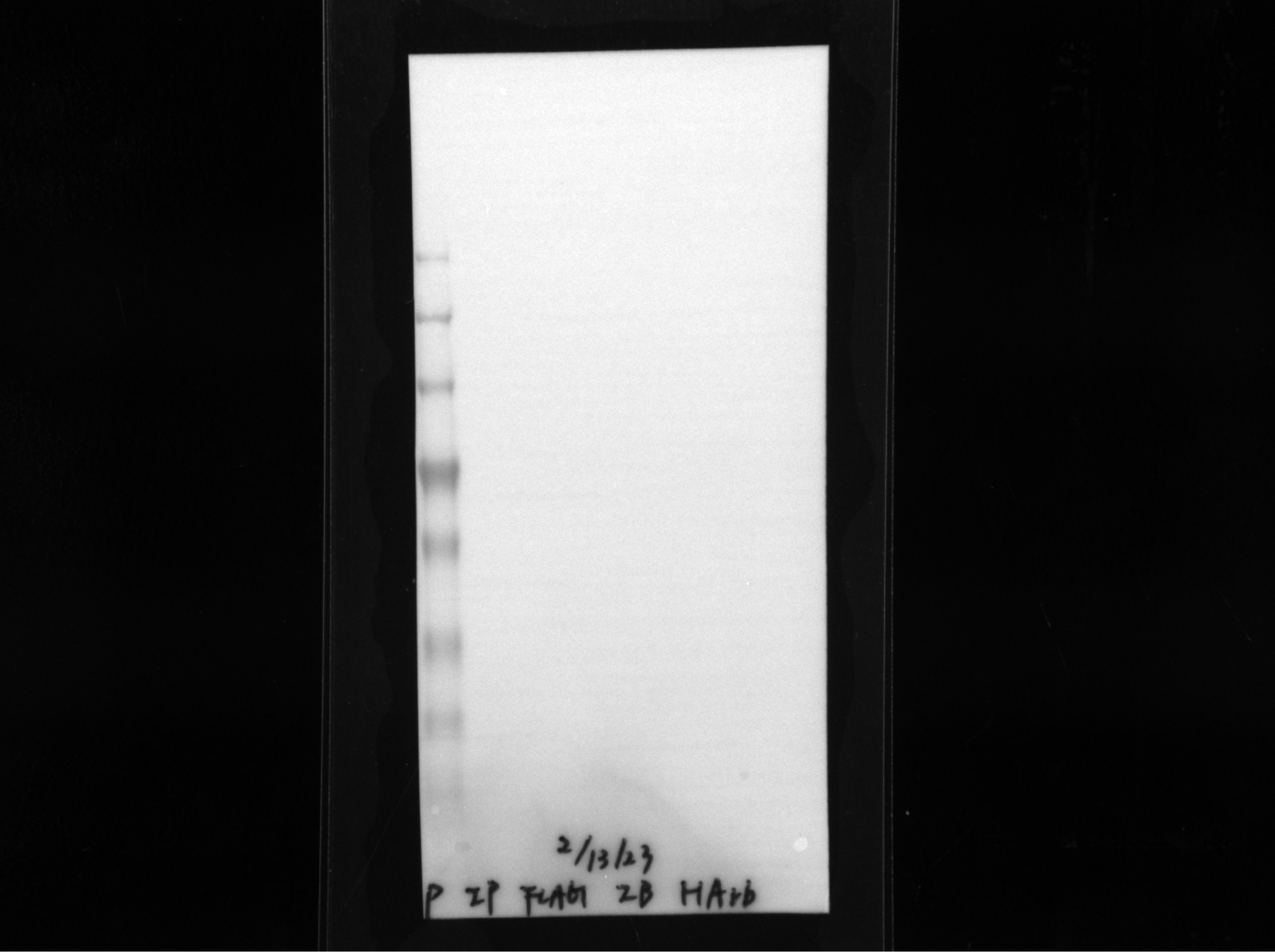

Supplement: Figure 6—source data 3. [file elife-89002-fig6-data3.zip › IP FLAG anti-HArb_Marker.jpg]

**b**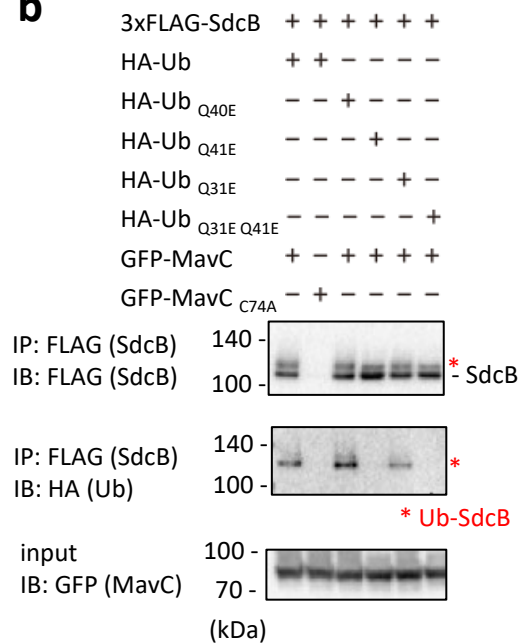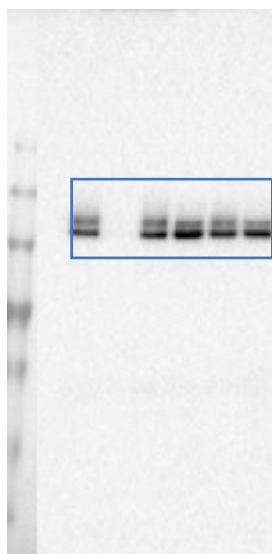

**Figure 6b**  
**top**

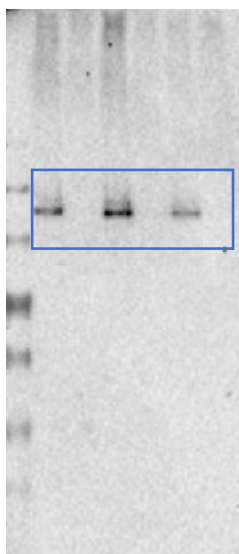

**Figure6b**  
**middle**

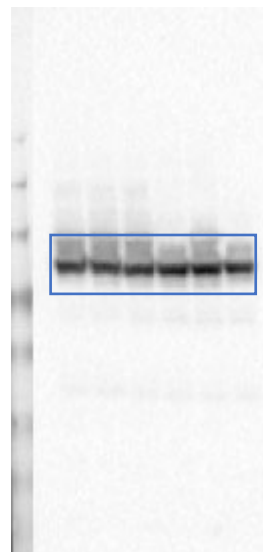

**Figure6b**  
**bottom**

Supplement: Figure 6—source data 4. [file elife-89002-fig6-data4.pdf]

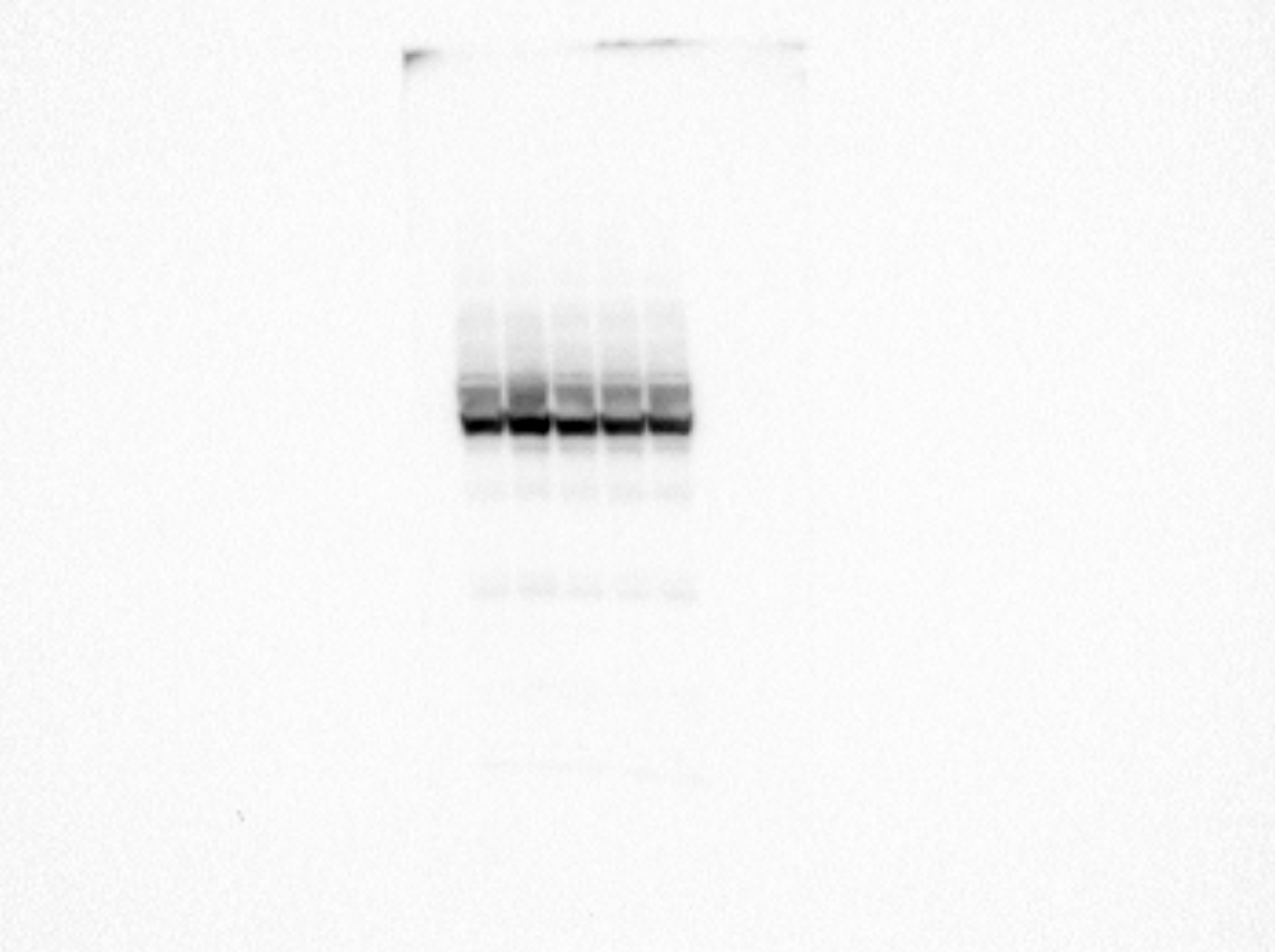

Supplement: Figure 6—source data 5. [file elife-89002-fig6-data5.zip › input anti-GFPrb_Exposure_13.4sec.jpg]

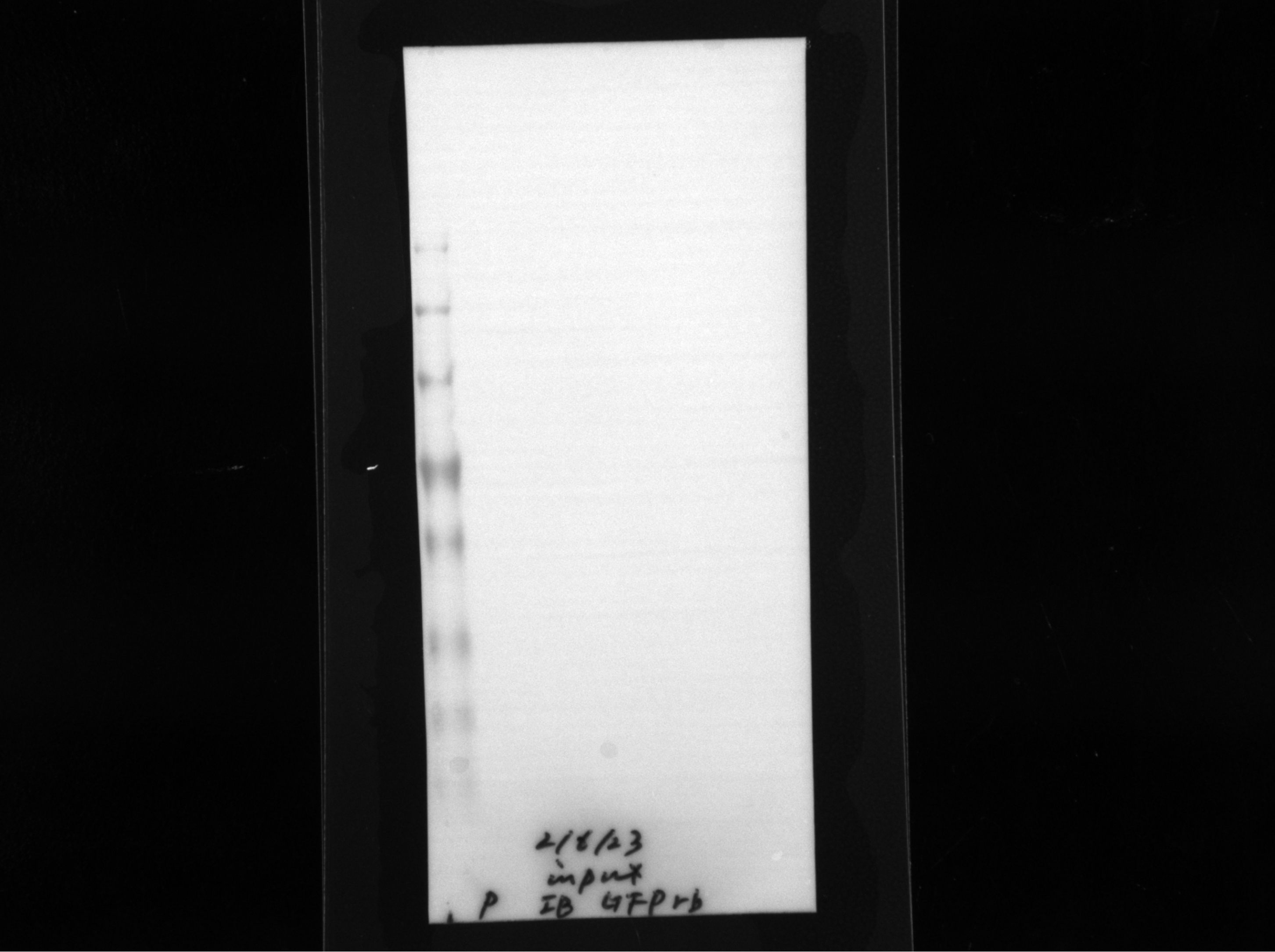

Supplement: Figure 6—source data 5. [file elife-89002-fig6-data5.zip › input anti-GFPrb_Marker.jpg]

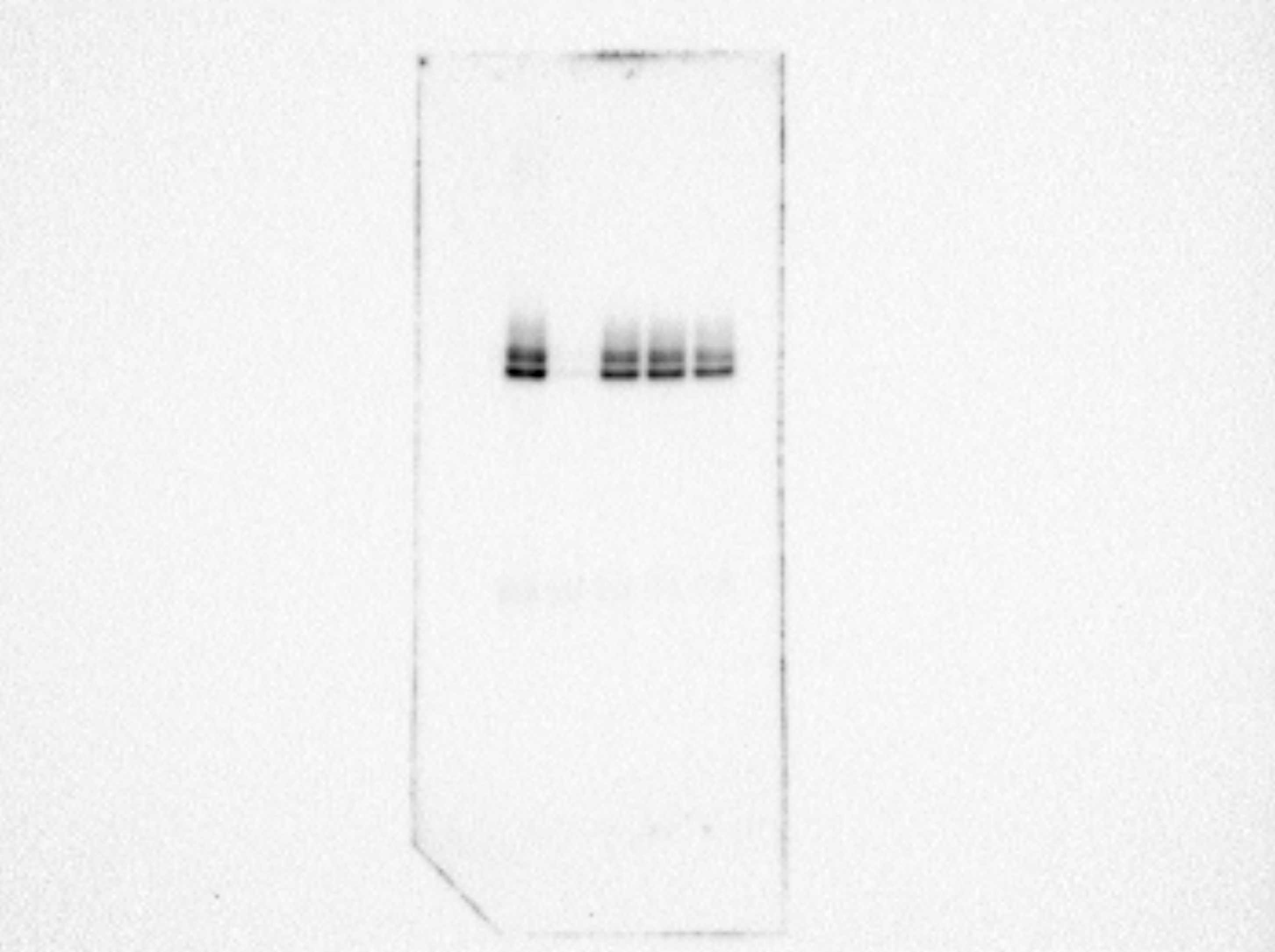

Supplement: Figure 6—source data 5. [file elife-89002-fig6-data5.zip › IP FLAG anti-FLAGm_Exposure_32.0sec.jpg]

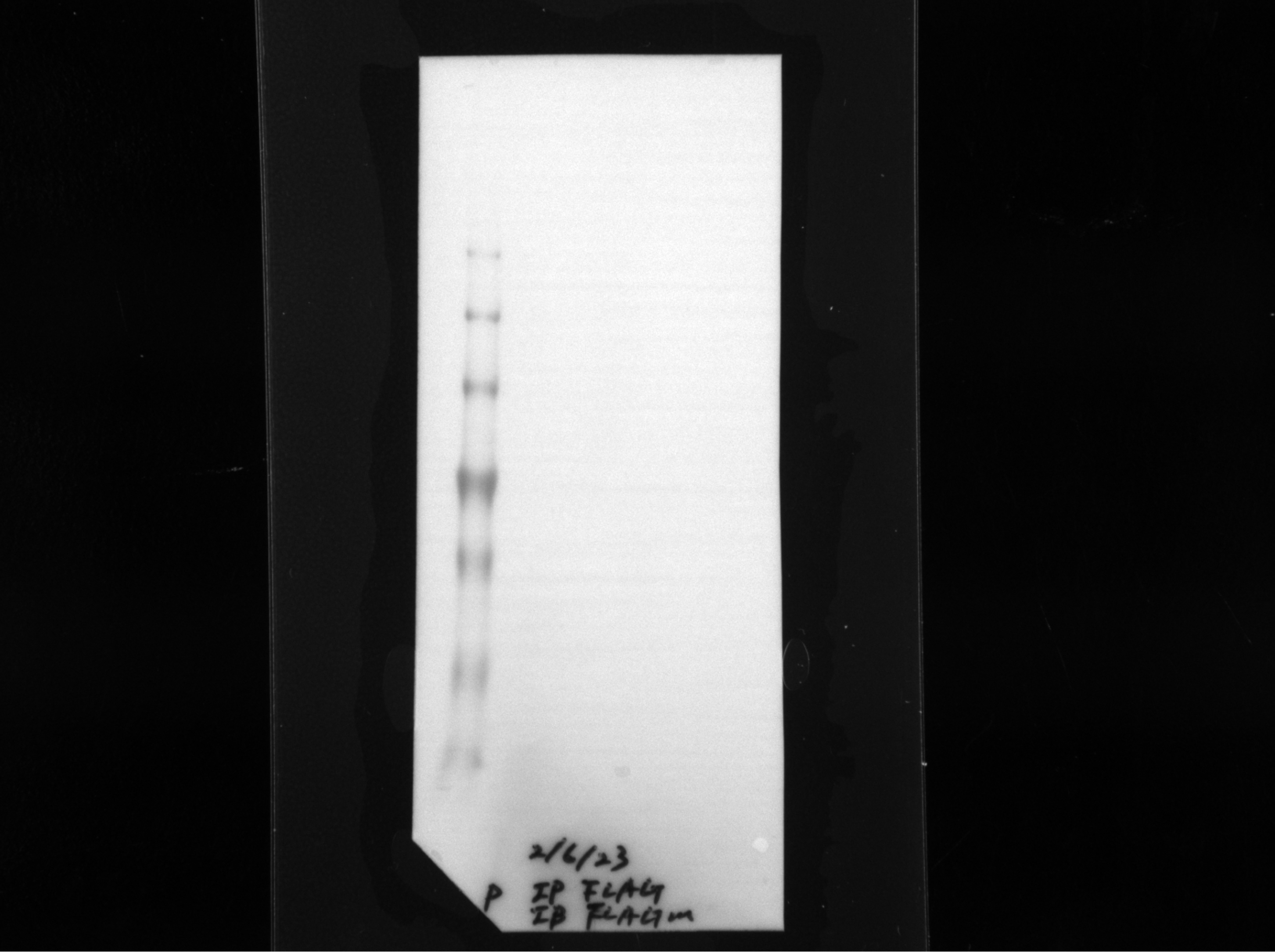

Supplement: Figure 6—source data 5. [file elife-89002-fig6-data5.zip › IP FLAG anti-FLAGm_Marker.jpg]

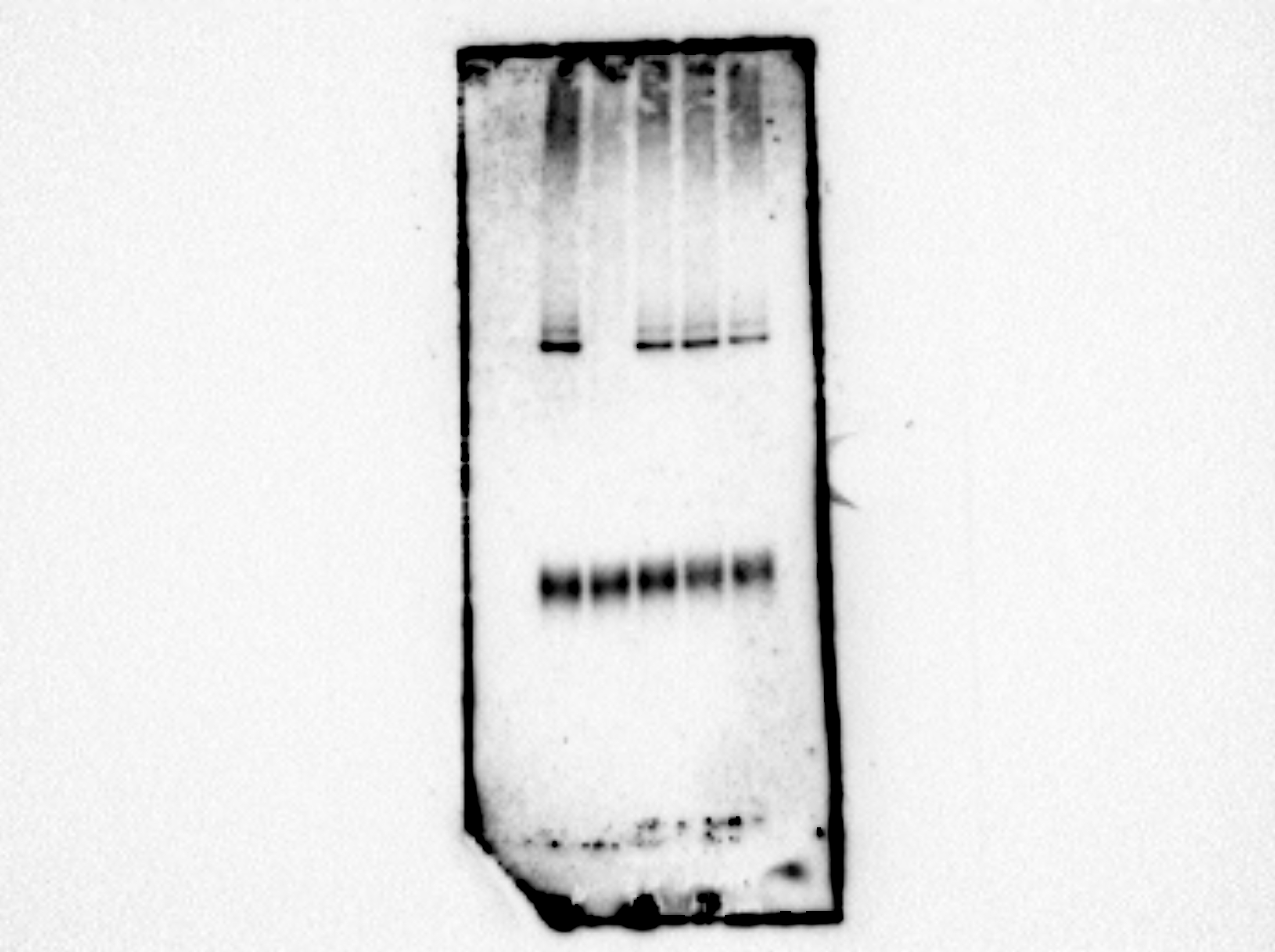

Supplement: Figure 6—source data 5. [file elife-89002-fig6-data5.zip › IP FLAG anti-HAm super_Exposure_25.8sec.jpg]

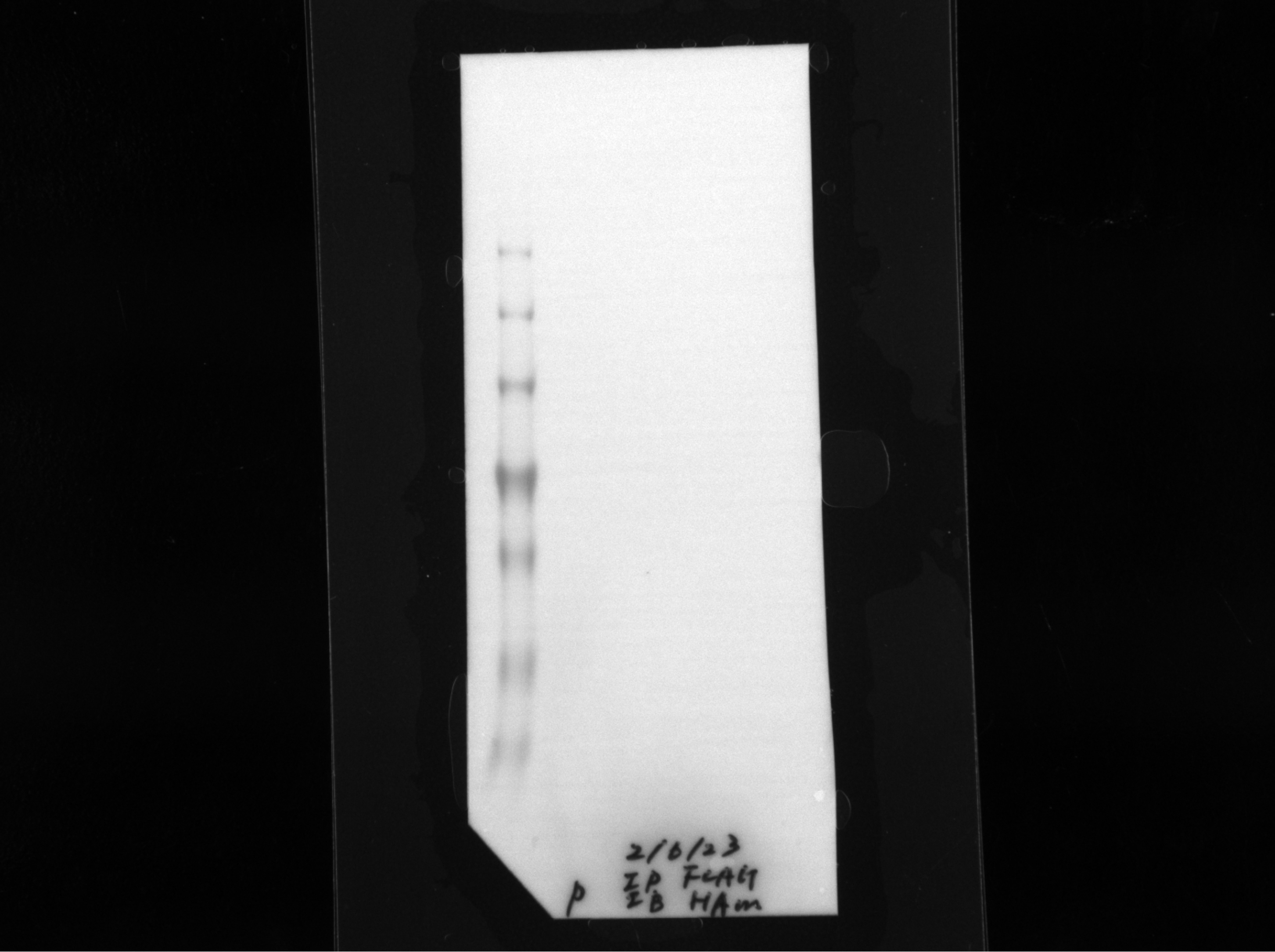

Supplement: Figure 6—source data 5. [file elife-89002-fig6-data5.zip › IP FLAG anti-HAm super_Marker.jpg]

**C**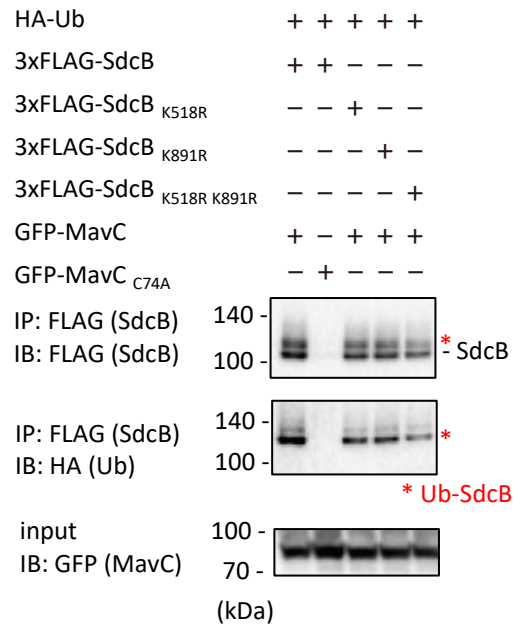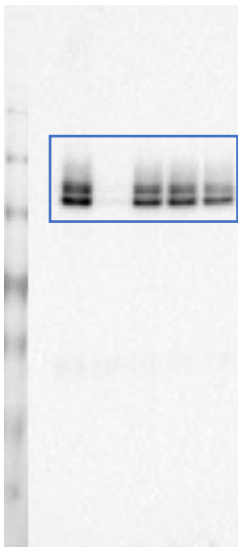

**Figure 6c**  
**top**

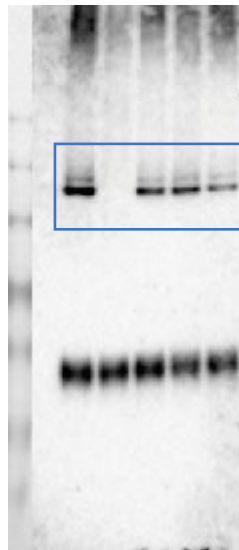

**Figure6c**  
**middle**

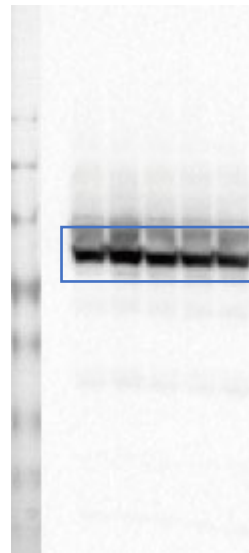

**Figure6c**  
**bottom**

Supplement: Figure 6—source data 6. [file elife-89002-fig6-data6.pdf]

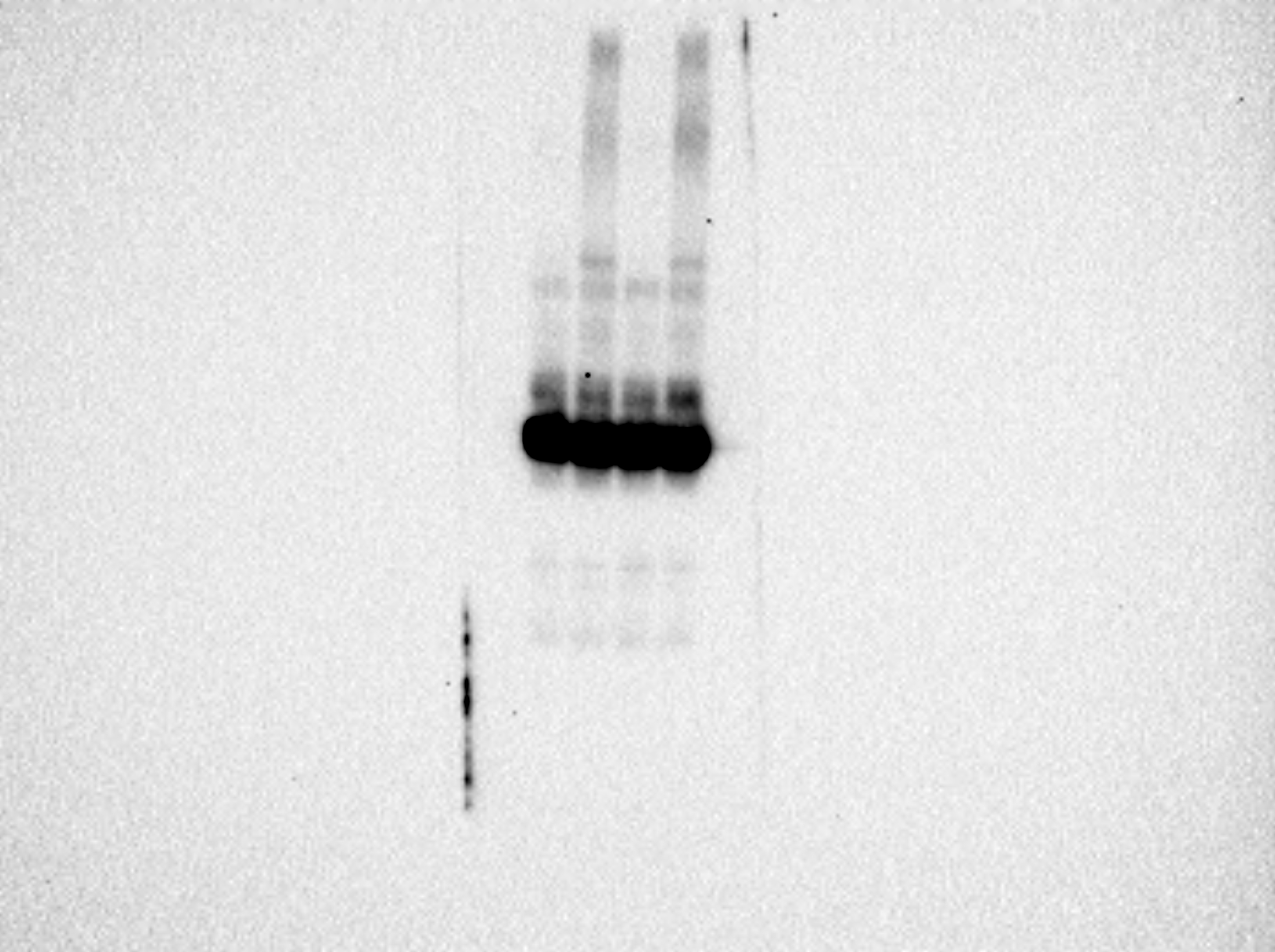

Supplement: Figure 6—figure supplement 2—source data 1. [file elife-89002-fig6-figsupp2-data1.zip › IP FLAG anti-FLAGm_Exposure_60.0sec.jpg]

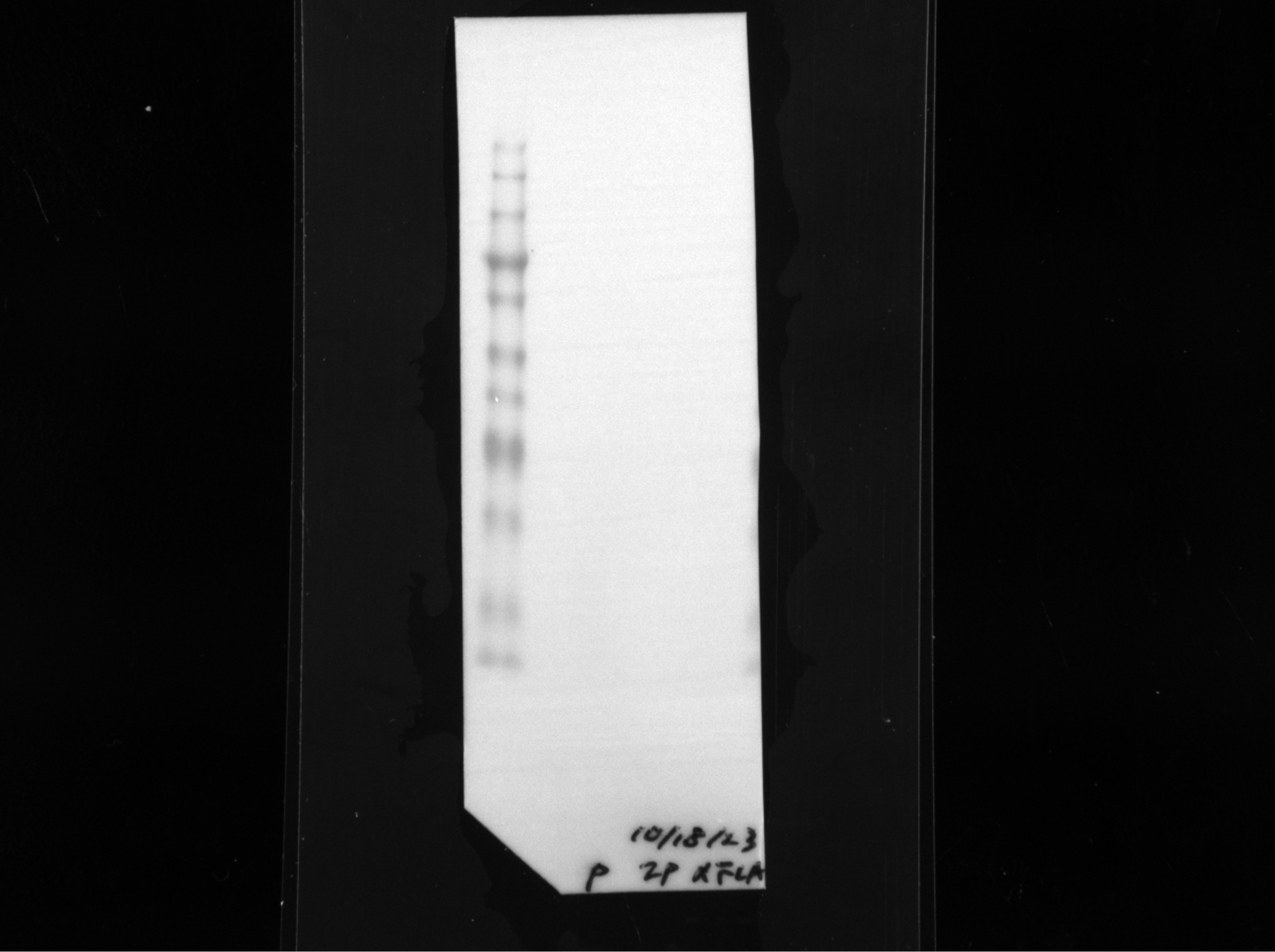

Supplement: Figure 6—figure supplement 2—source data 1. [file elife-89002-fig6-figsupp2-data1.zip › IP FLAG anti-FLAGm_Marker.jpg]

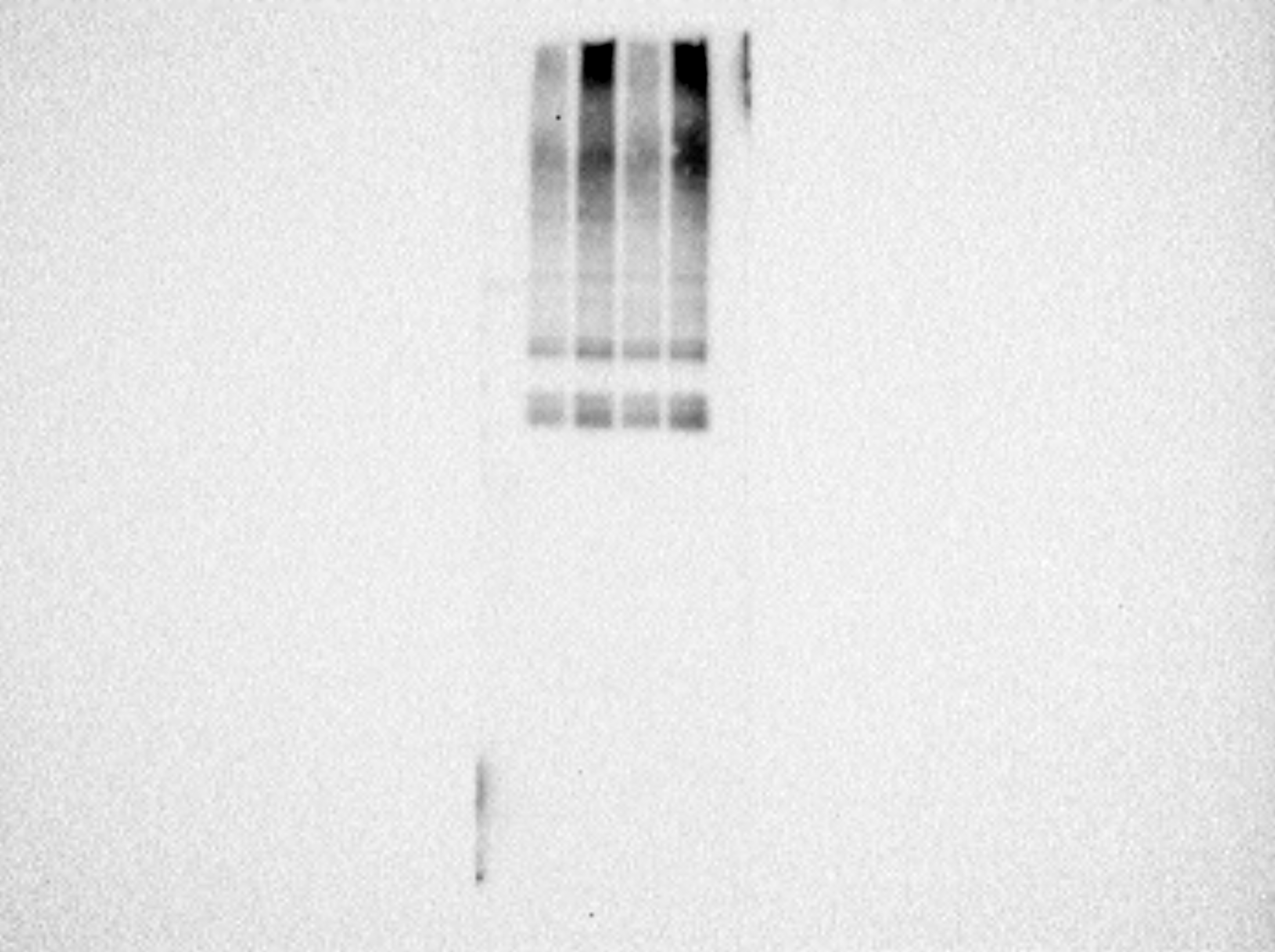

Supplement: Figure 6—figure supplement 2—source data 1. [file elife-89002-fig6-figsupp2-data1.zip › IP FLAG anti-HArb_Exposure_60.0sec.jpg]

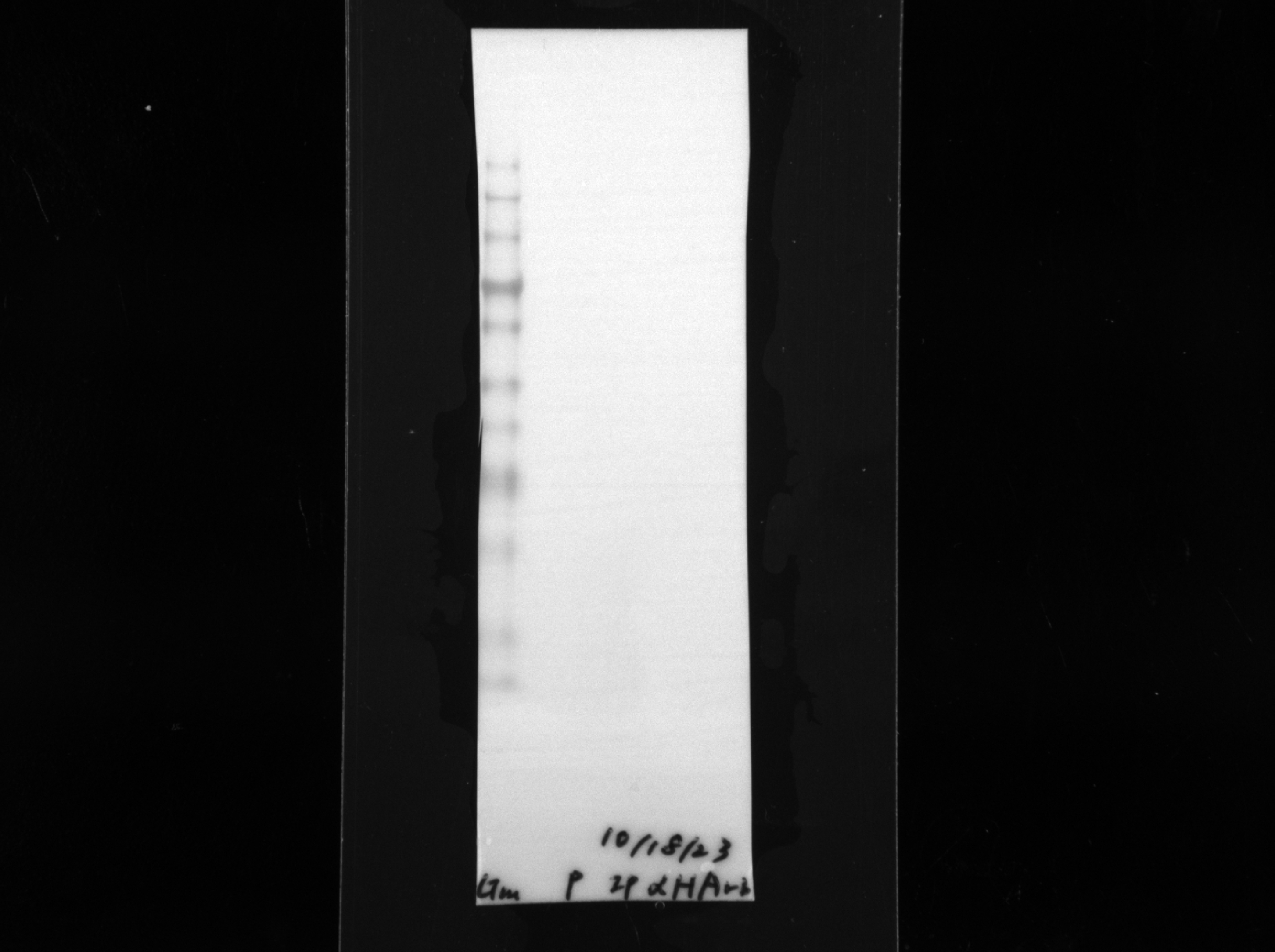

Supplement: Figure 6—figure supplement 2—source data 1. [file elife-89002-fig6-figsupp2-data1.zip › IP FLAG anti-HArb_Marker.jpg]

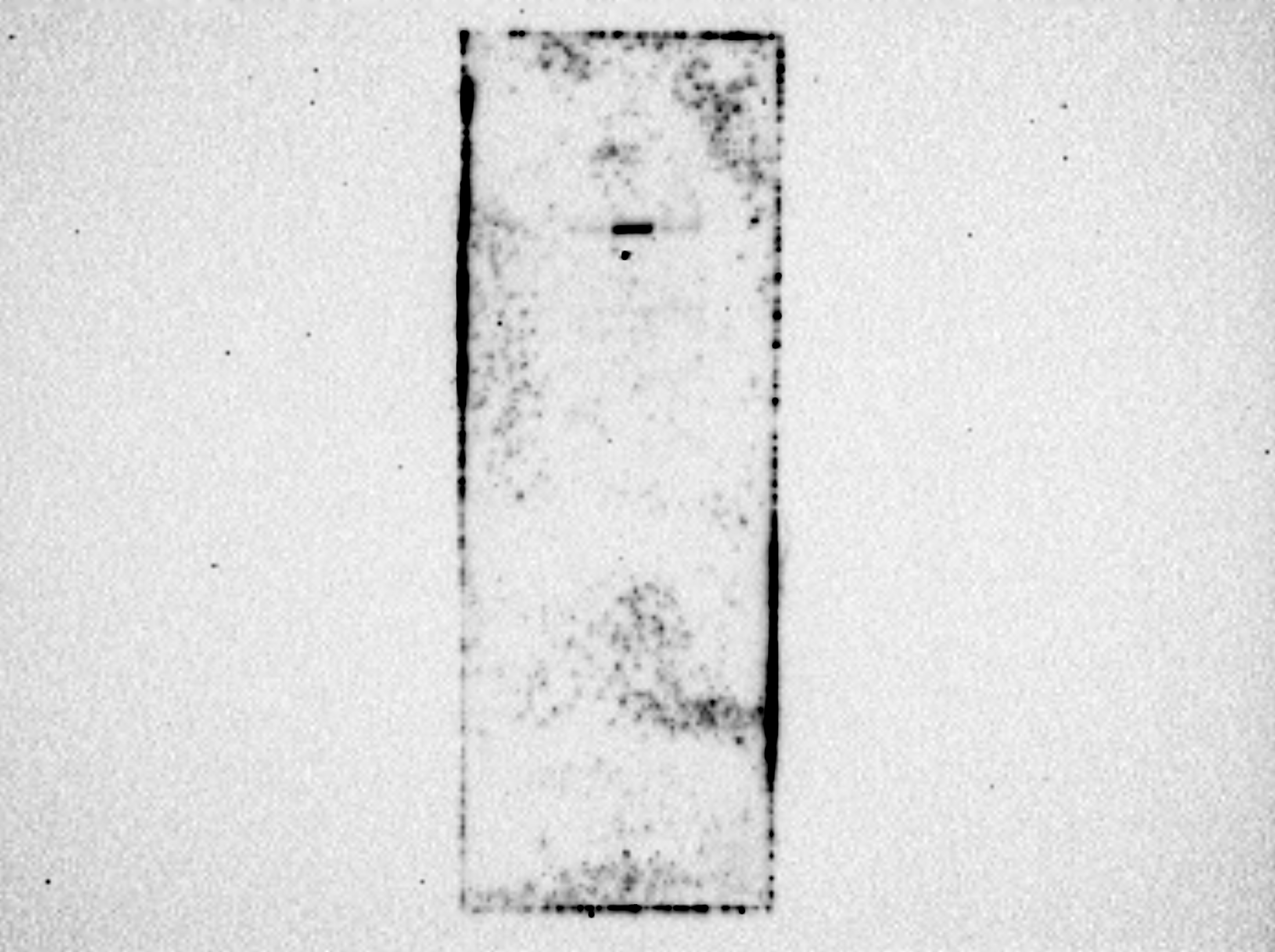

Supplement: Figure 6—figure supplement 2—source data 1. [file elife-89002-fig6-figsupp2-data1.zip › IP Myc anti-Mycm_Exposure_300.0sec.jpg]

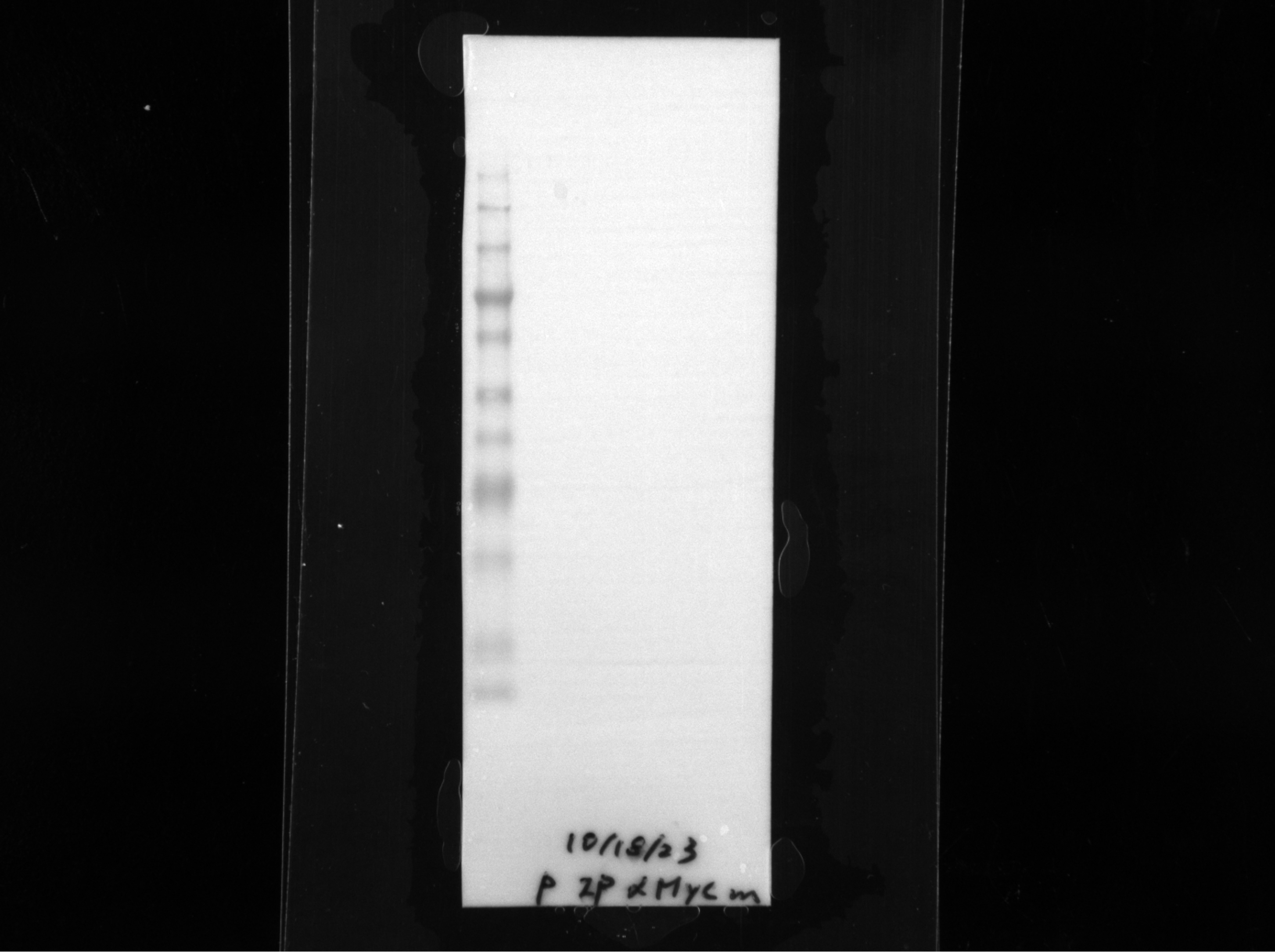

Supplement: Figure 6—figure supplement 2—source data 1. [file elife-89002-fig6-figsupp2-data1.zip › IP Myc anti-Mycm_Marker.jpg]

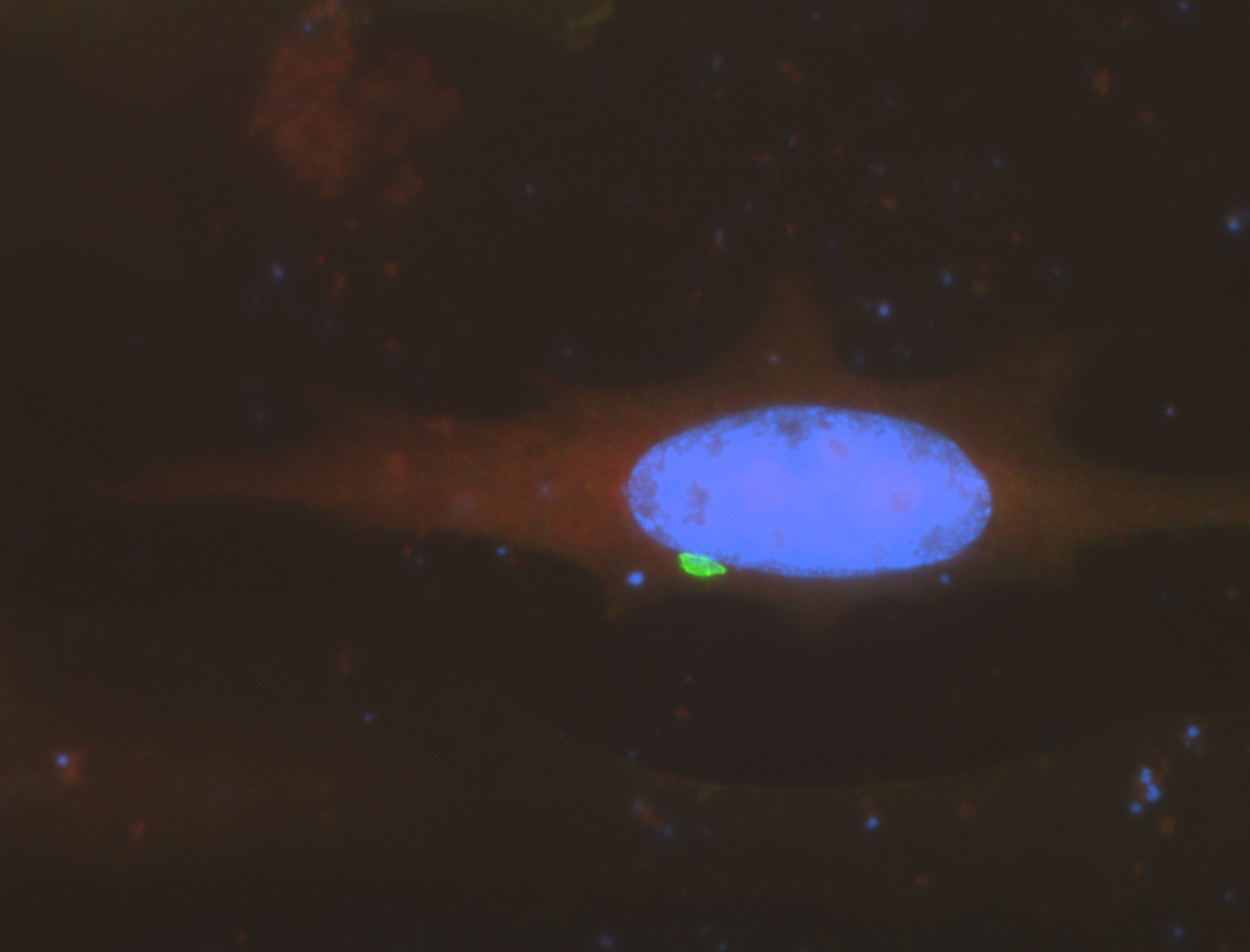

Supplement: Figure 7—source data 1. [file elife-89002-fig7-data1.zip › A1 3-.tif]

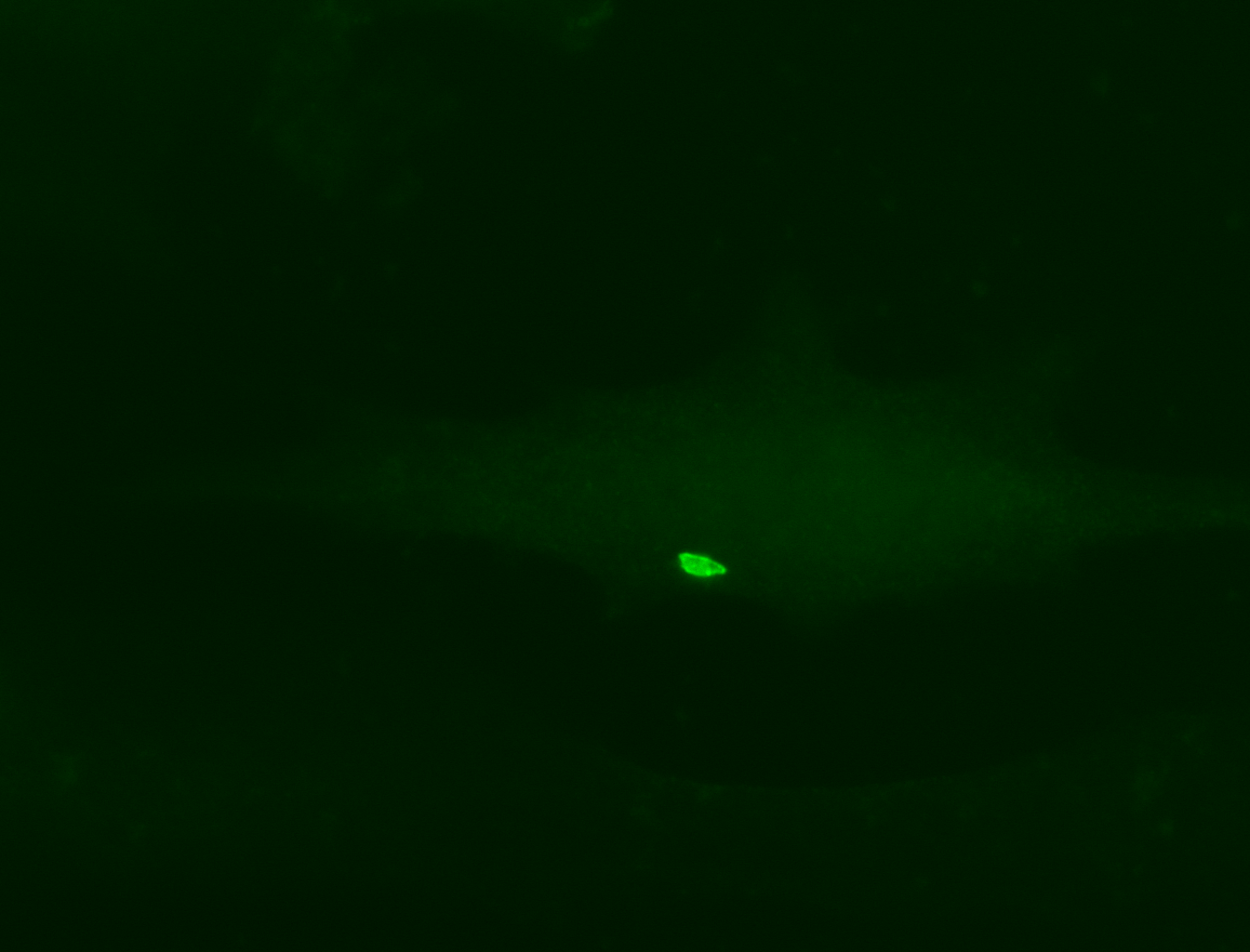

Supplement: Figure 7—source data 1. [file elife-89002-fig7-data1.zip › a1 3-c1.tif]

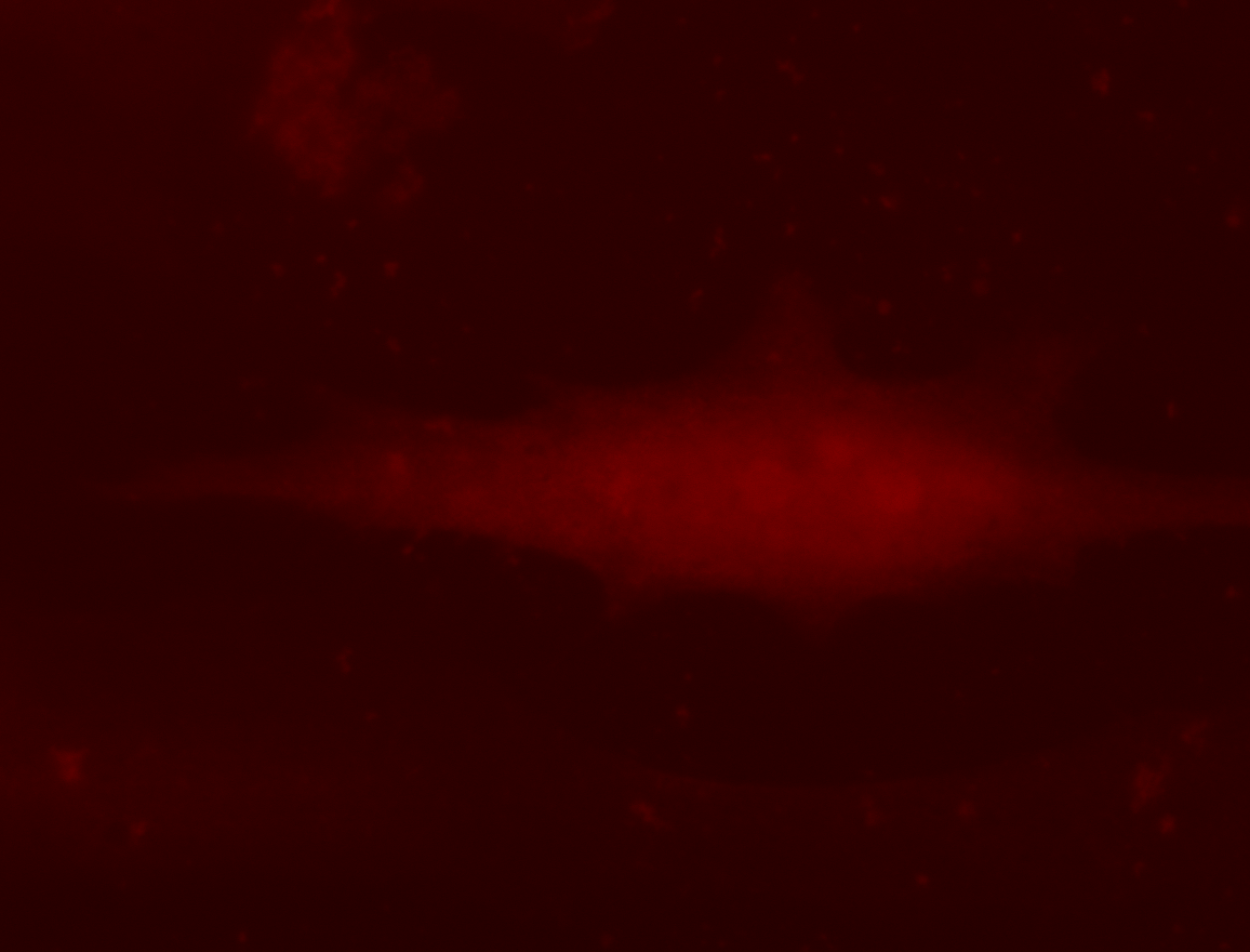

Supplement: Figure 7—source data 1. [file elife-89002-fig7-data1.zip › a1 3-c2.tif]

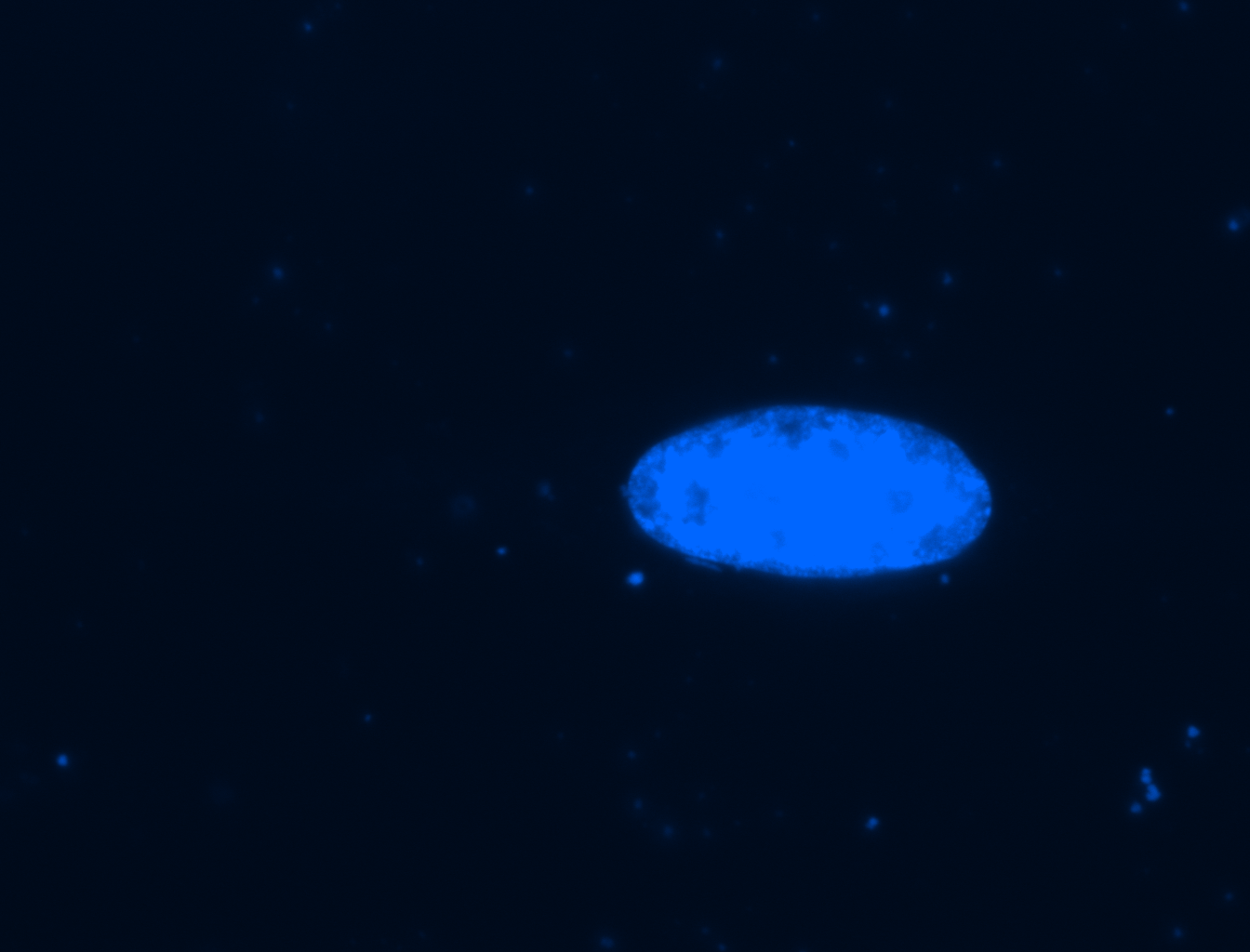

Supplement: Figure 7—source data 1. [file elife-89002-fig7-data1.zip › a1 3-c3.tif]

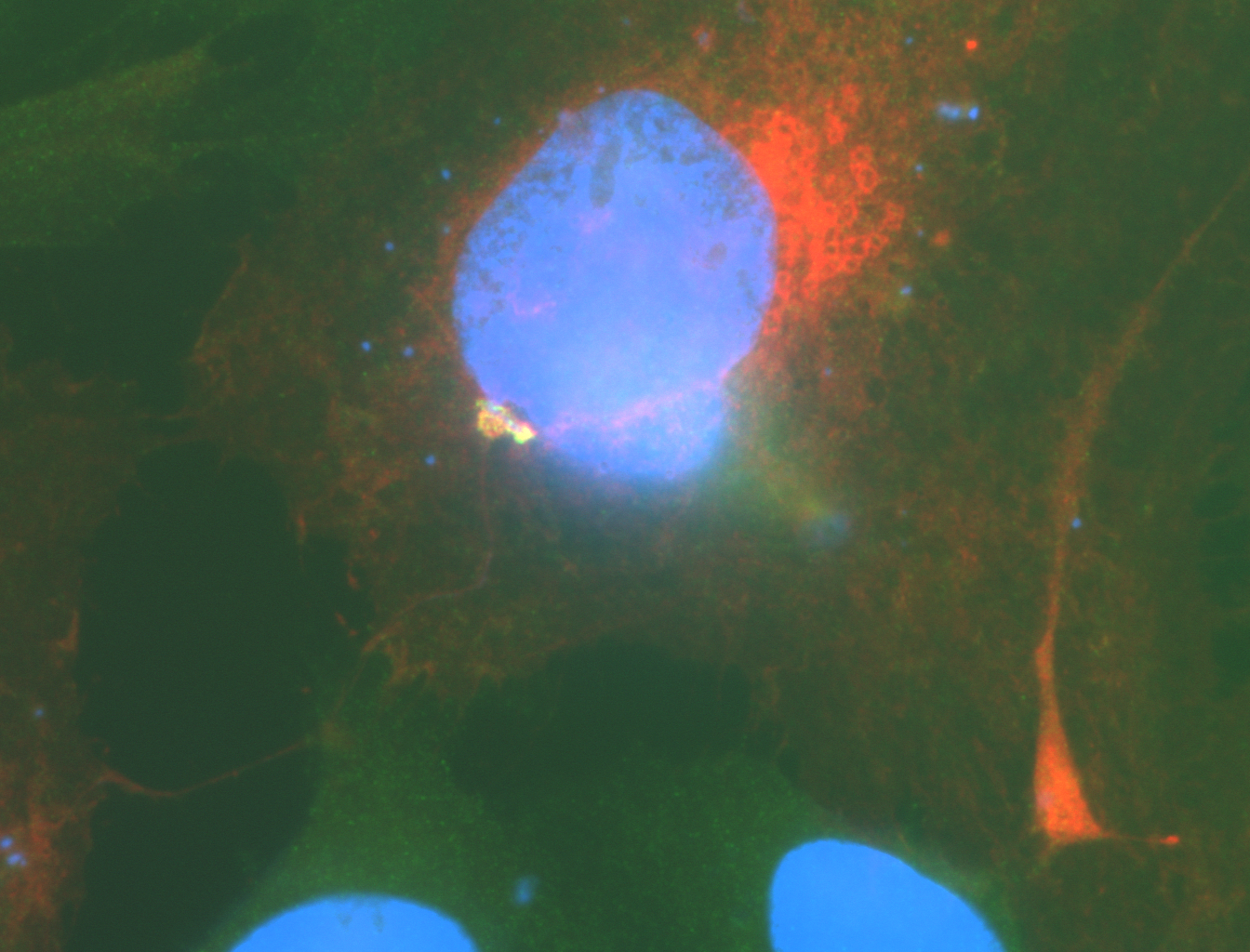

Supplement: Figure 7—source data 1. [file elife-89002-fig7-data1.zip › C1 8+.tif]

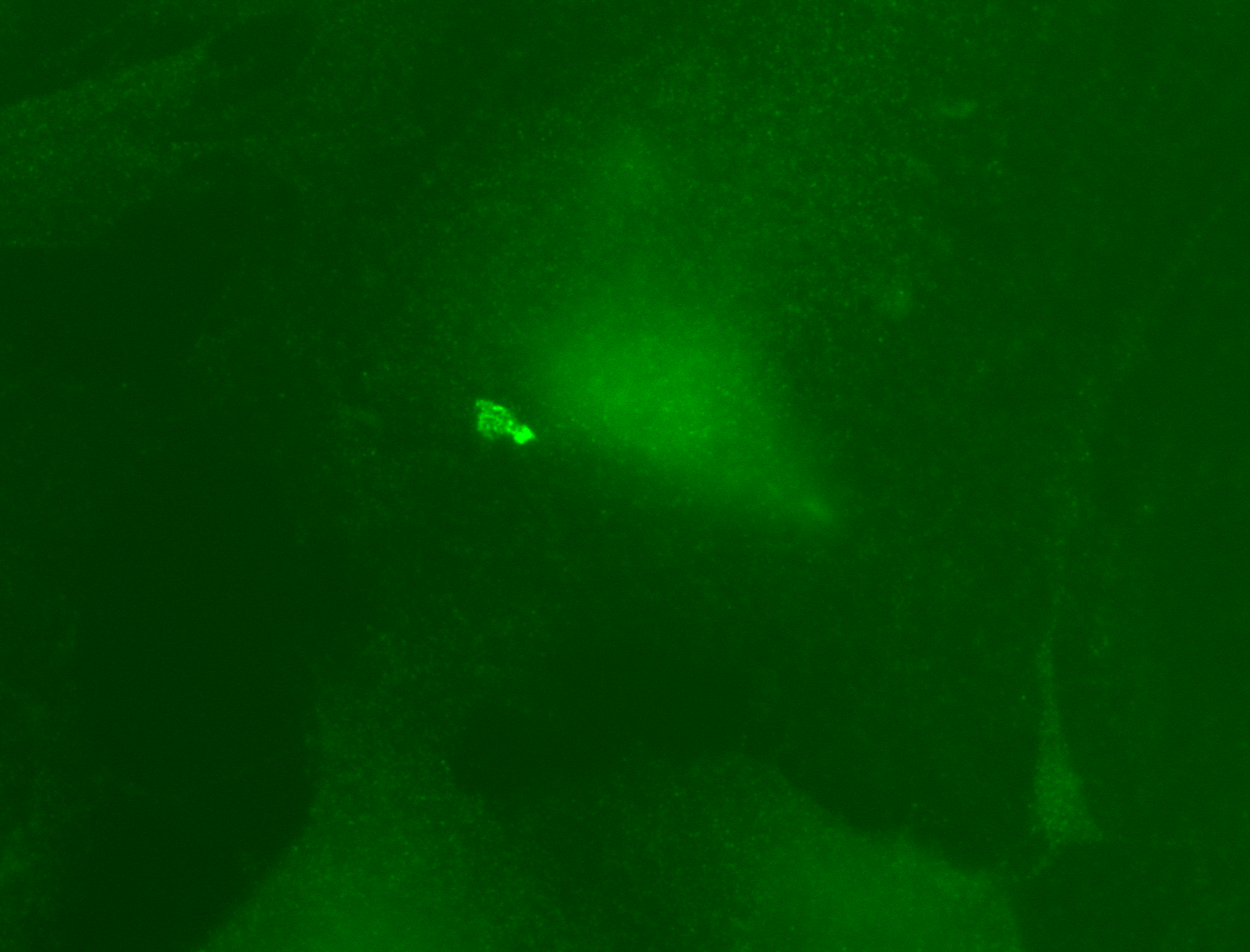

Supplement: Figure 7—source data 1. [file elife-89002-fig7-data1.zip › c1 8+c1.tif]

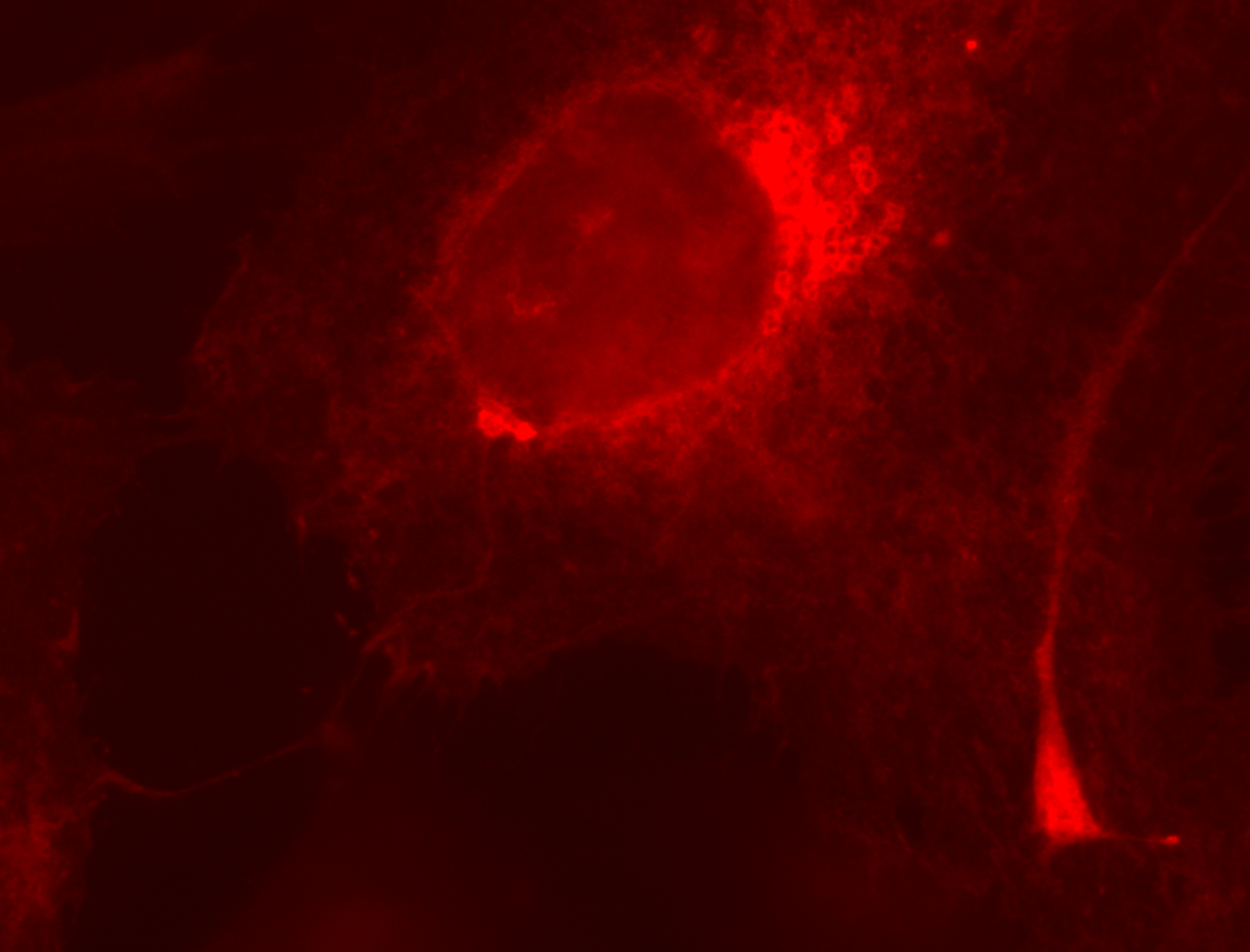

Supplement: Figure 7—source data 1. [file elife-89002-fig7-data1.zip › c1 8+c2.tif]

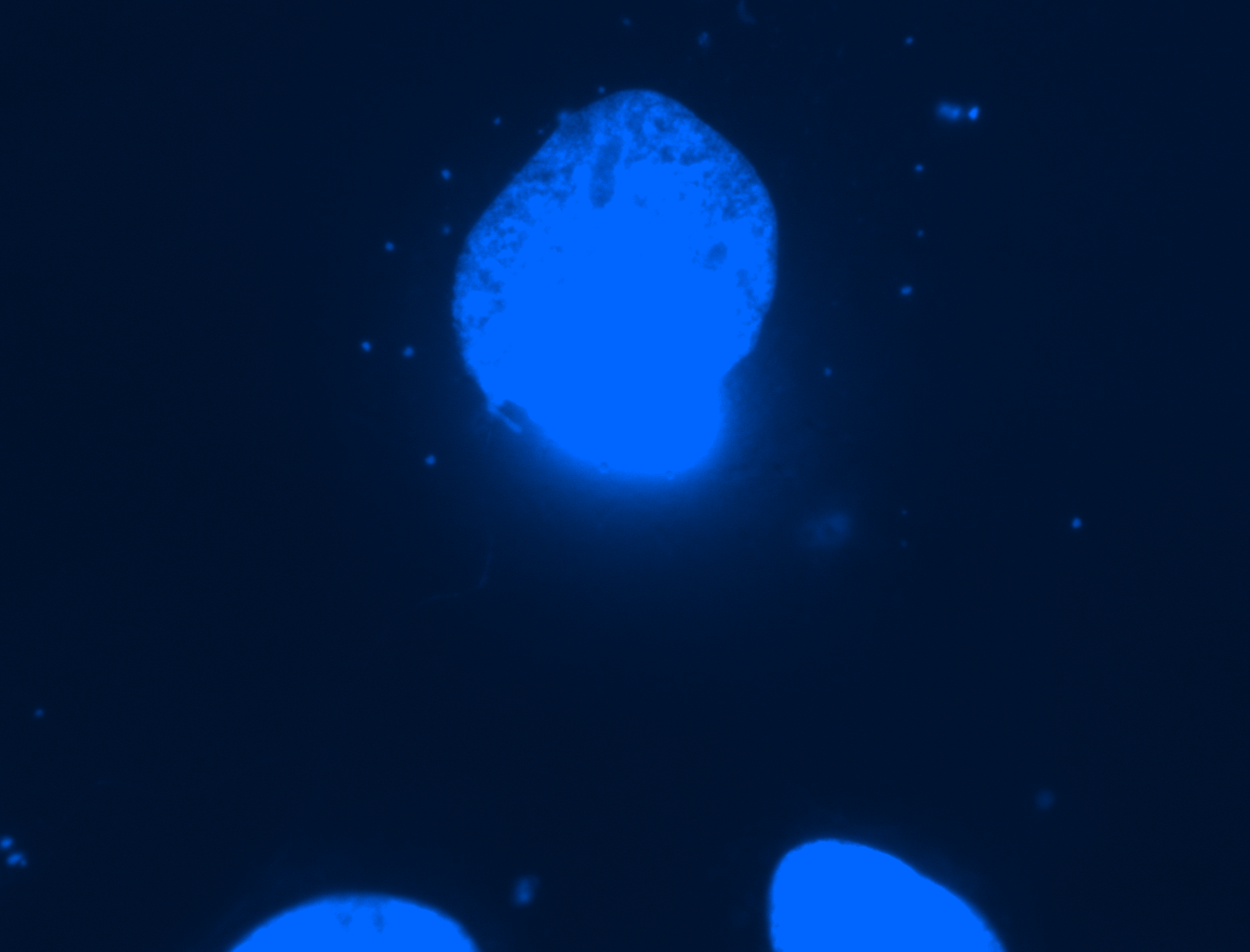

Supplement: Figure 7—source data 1. [file elife-89002-fig7-data1.zip › c1 8+c3.tif]

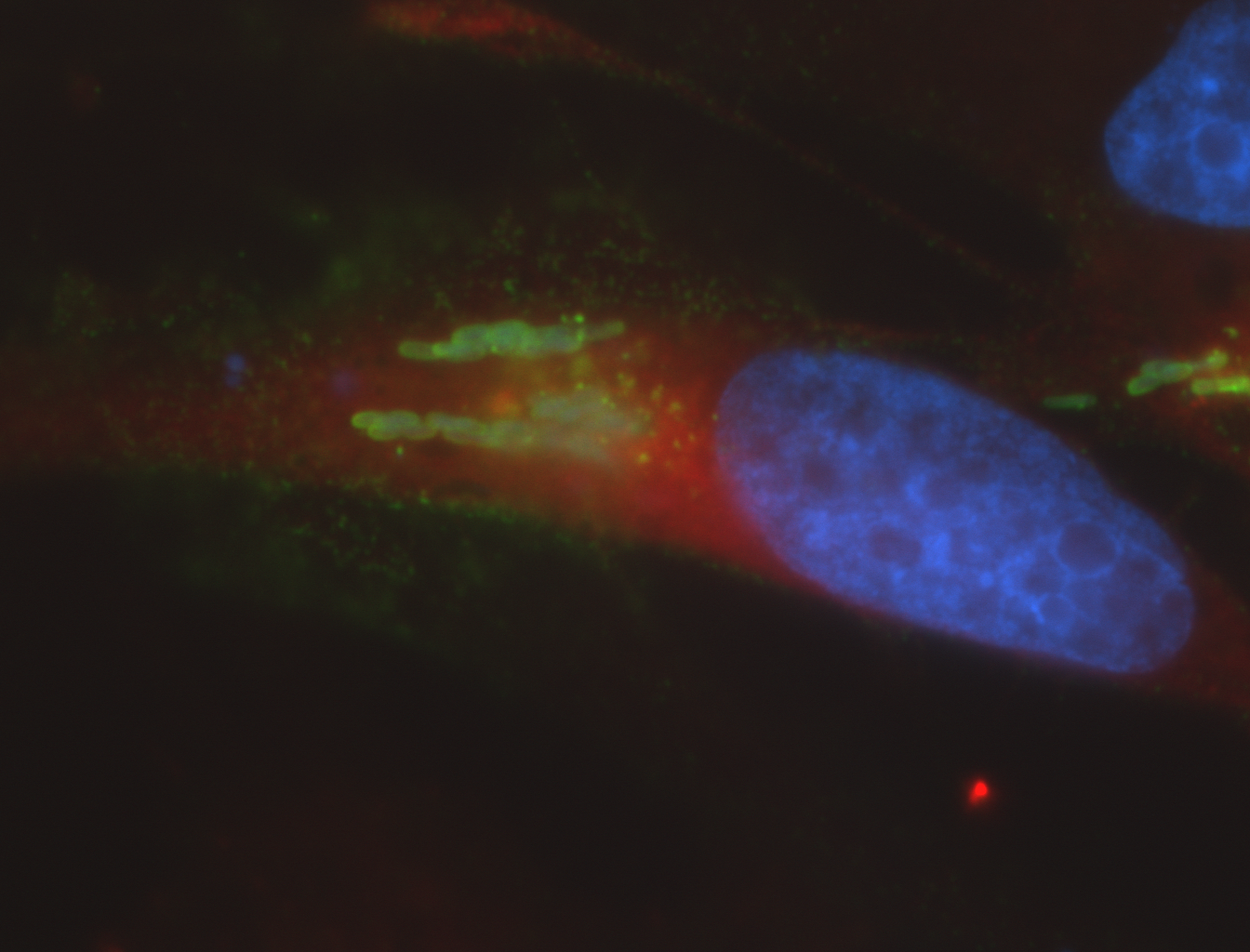

Supplement: Figure 7—figure supplement 1—source data 1. [file elife-89002-fig7-figsupp1-data1.zip › 9h del lpg2149 2-.tif]

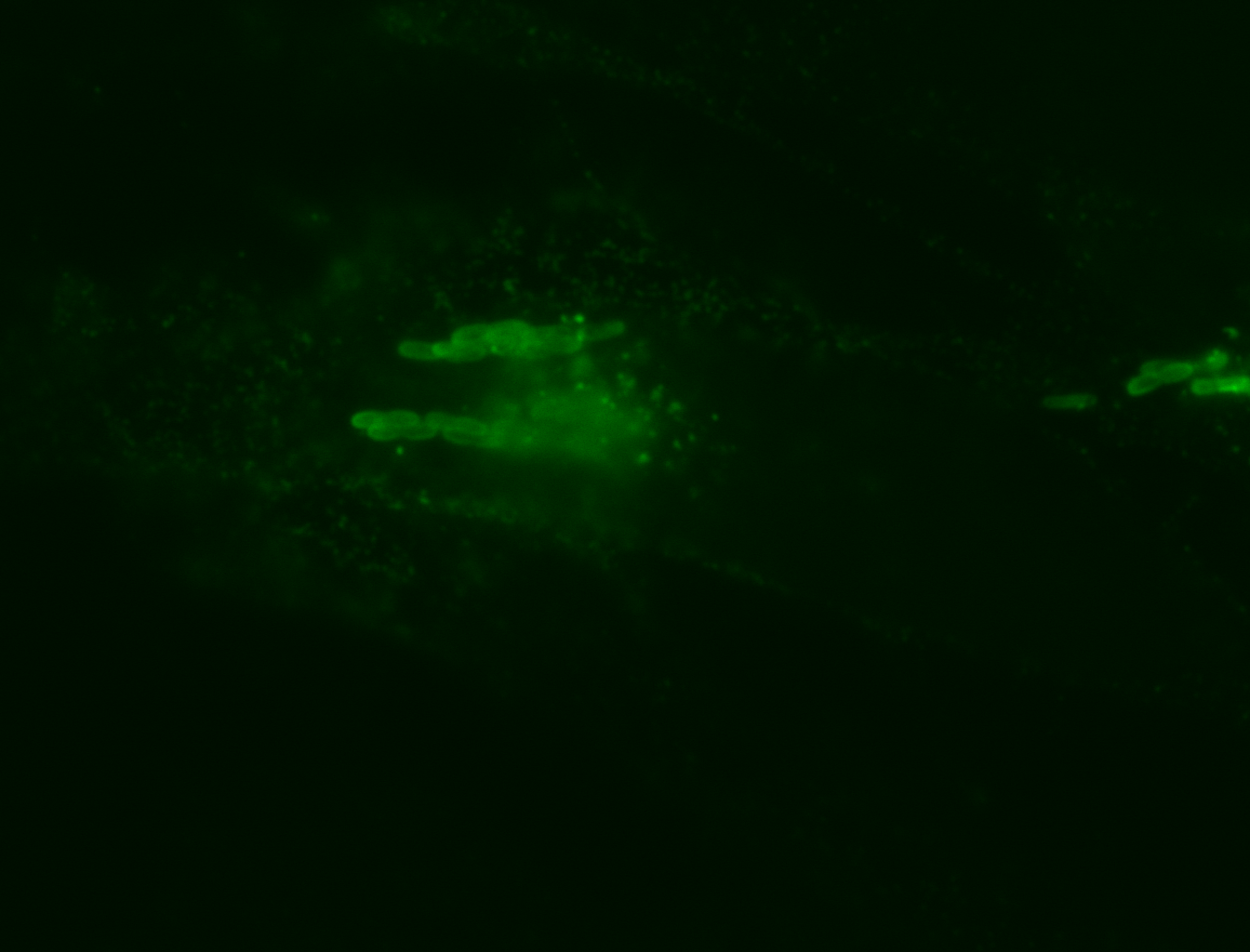

Supplement: Figure 7—figure supplement 1—source data 1. [file elife-89002-fig7-figsupp1-data1.zip › 9h del lpg2149 2-c1.tif]

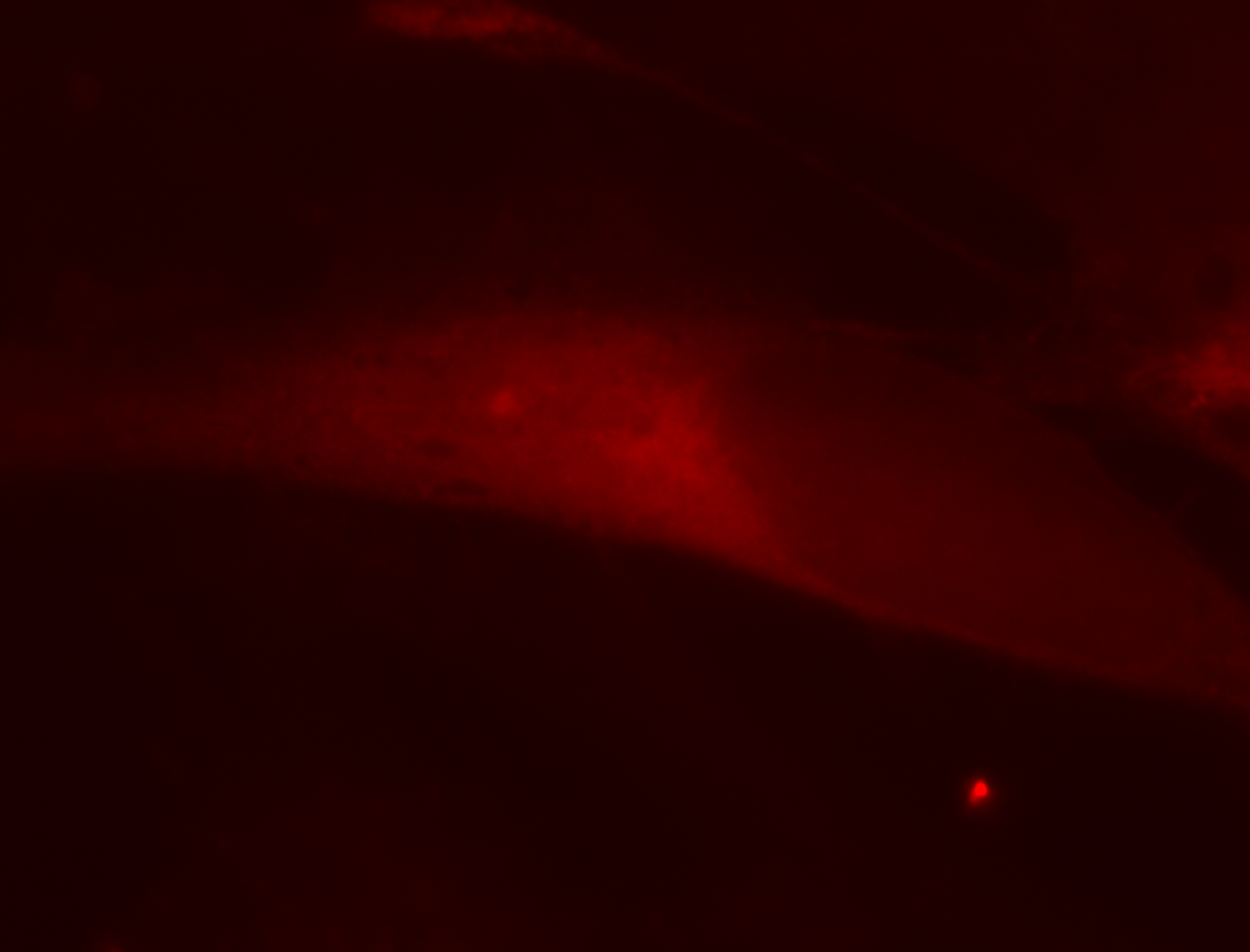

Supplement: Figure 7—figure supplement 1—source data 1. [file elife-89002-fig7-figsupp1-data1.zip › 9h del lpg2149 2-c2.tif]

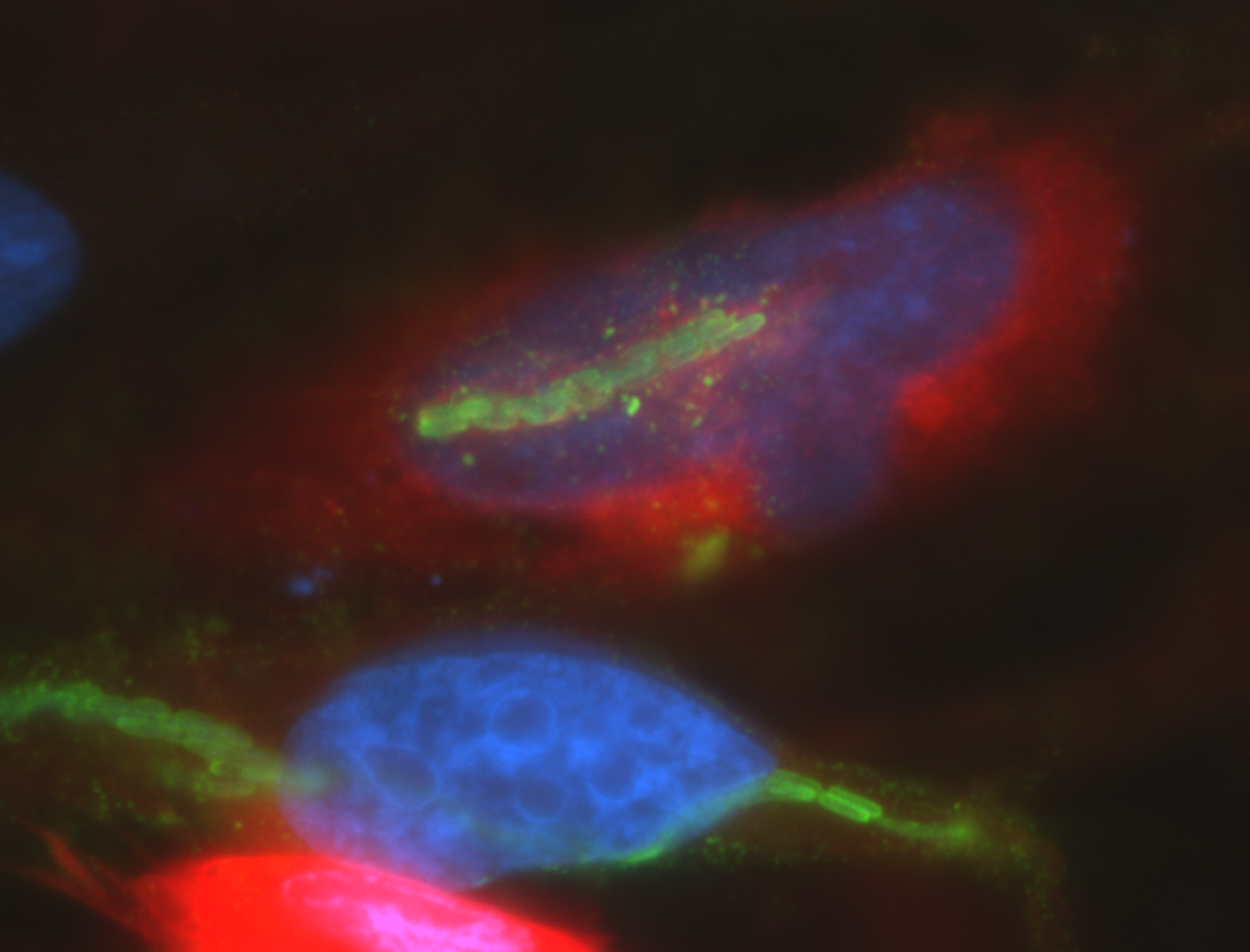

Supplement: Figure 7—figure supplement 1—source data 1. [file elife-89002-fig7-figsupp1-data1.zip › 9h del mavC mvcA 6+.tif]

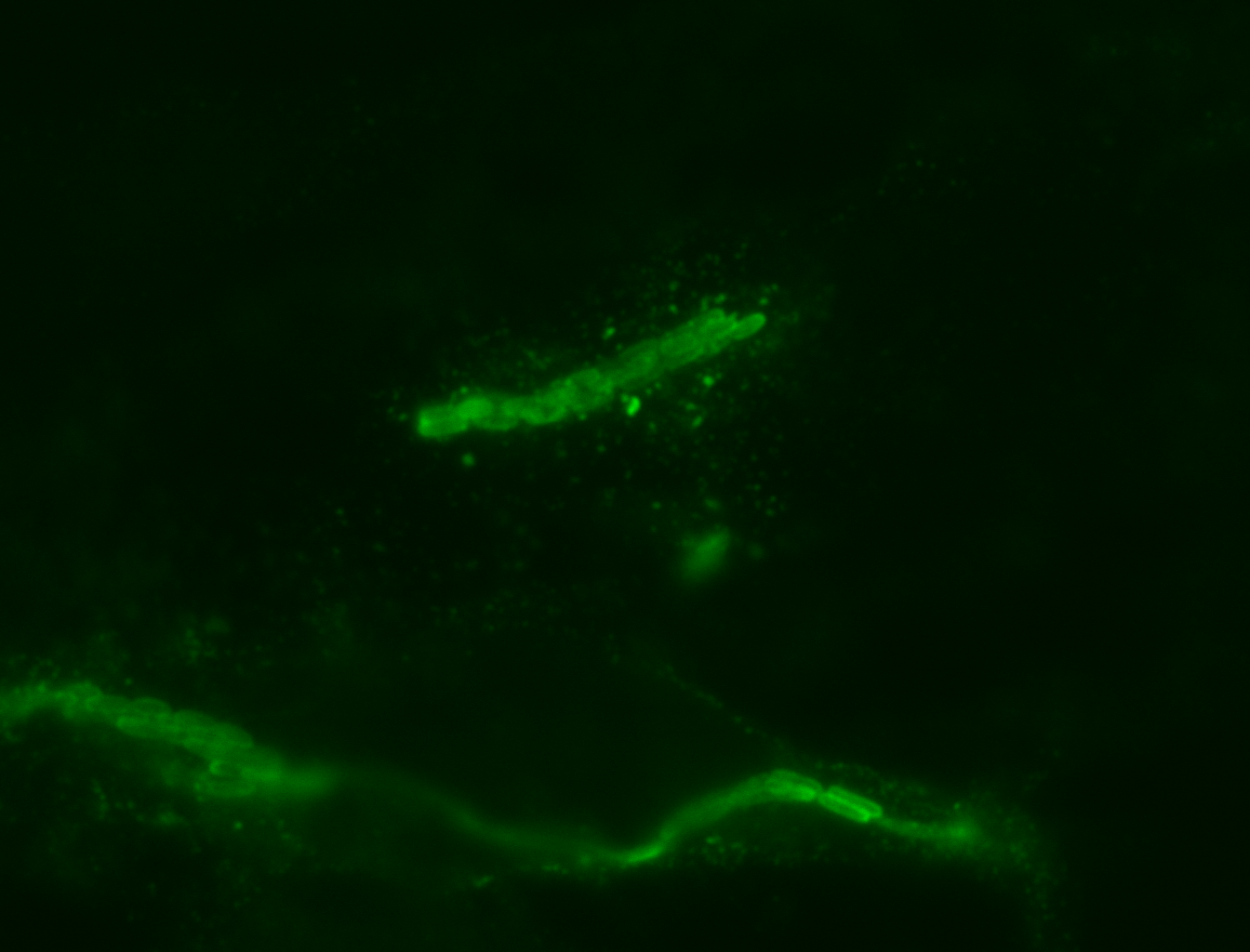

Supplement: Figure 7—figure supplement 1—source data 1. [file elife-89002-fig7-figsupp1-data1.zip › 9h del mavc mvca 6+c1.tif]

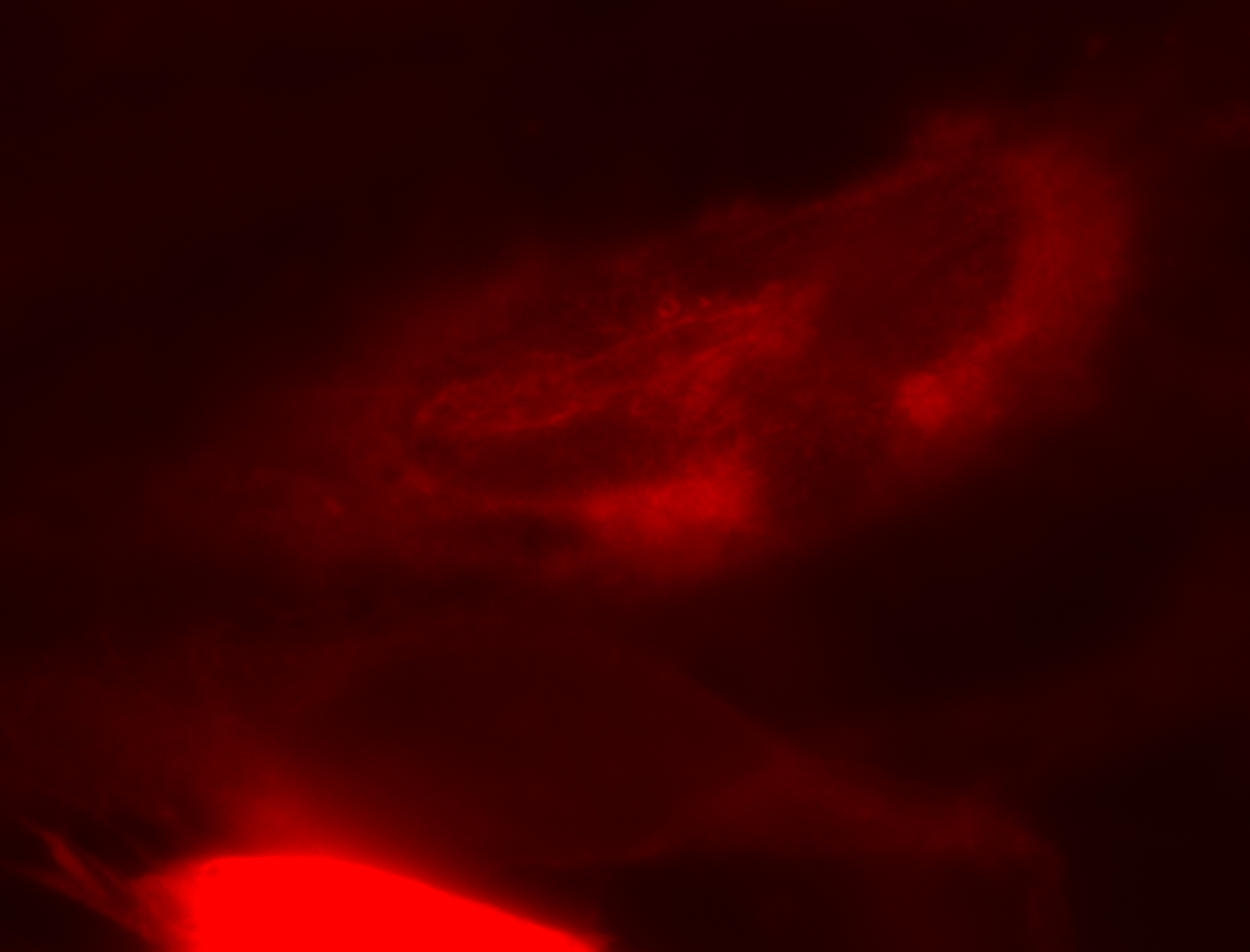

Supplement: Figure 7—figure supplement 1—source data 1. [file elife-89002-fig7-figsupp1-data1.zip › 9h del mavc mvca 6+c2.tif]

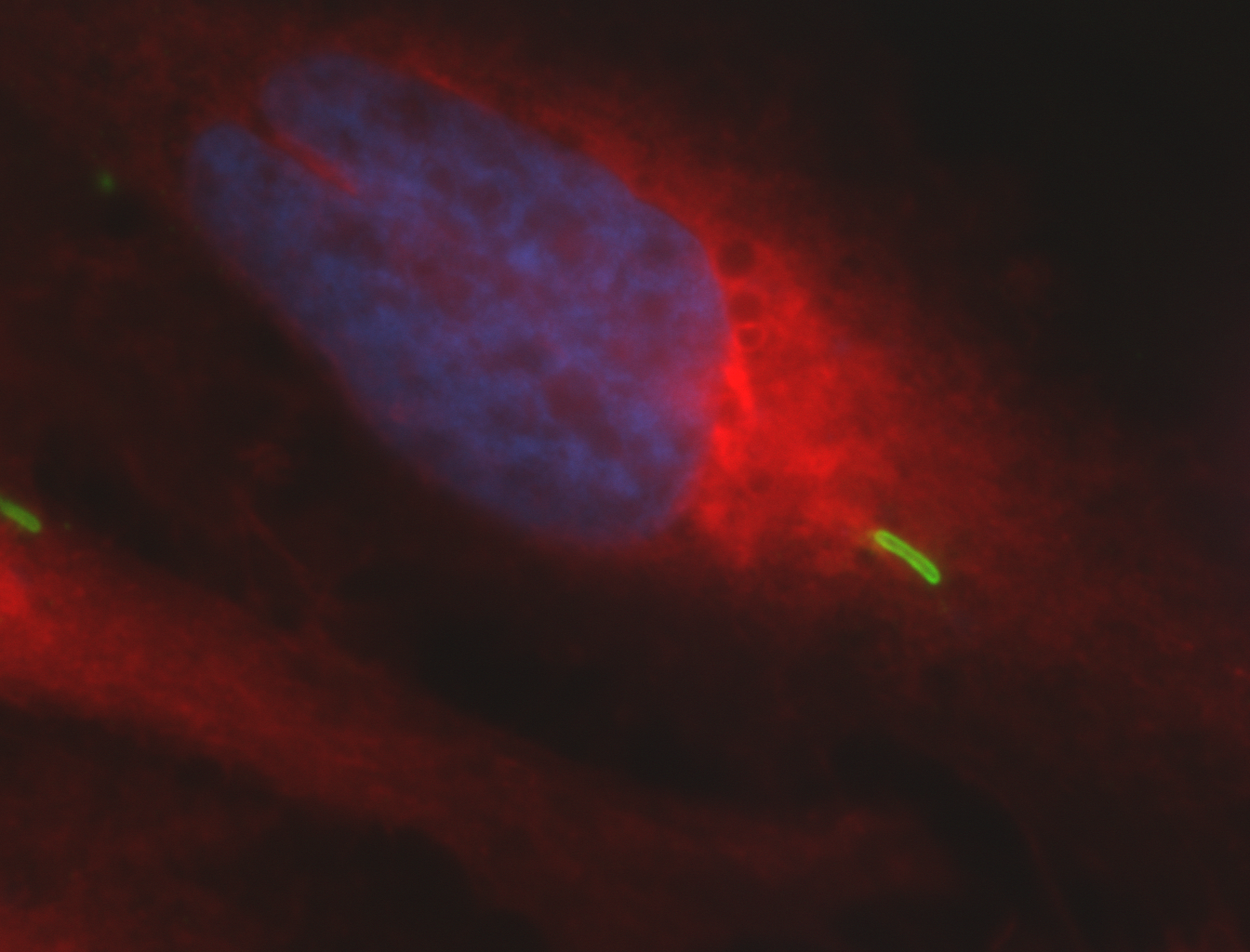

Supplement: Figure 7—figure supplement 1—source data 1. [file elife-89002-fig7-figsupp1-data1.zip › 9h del dotA 2-.tif]

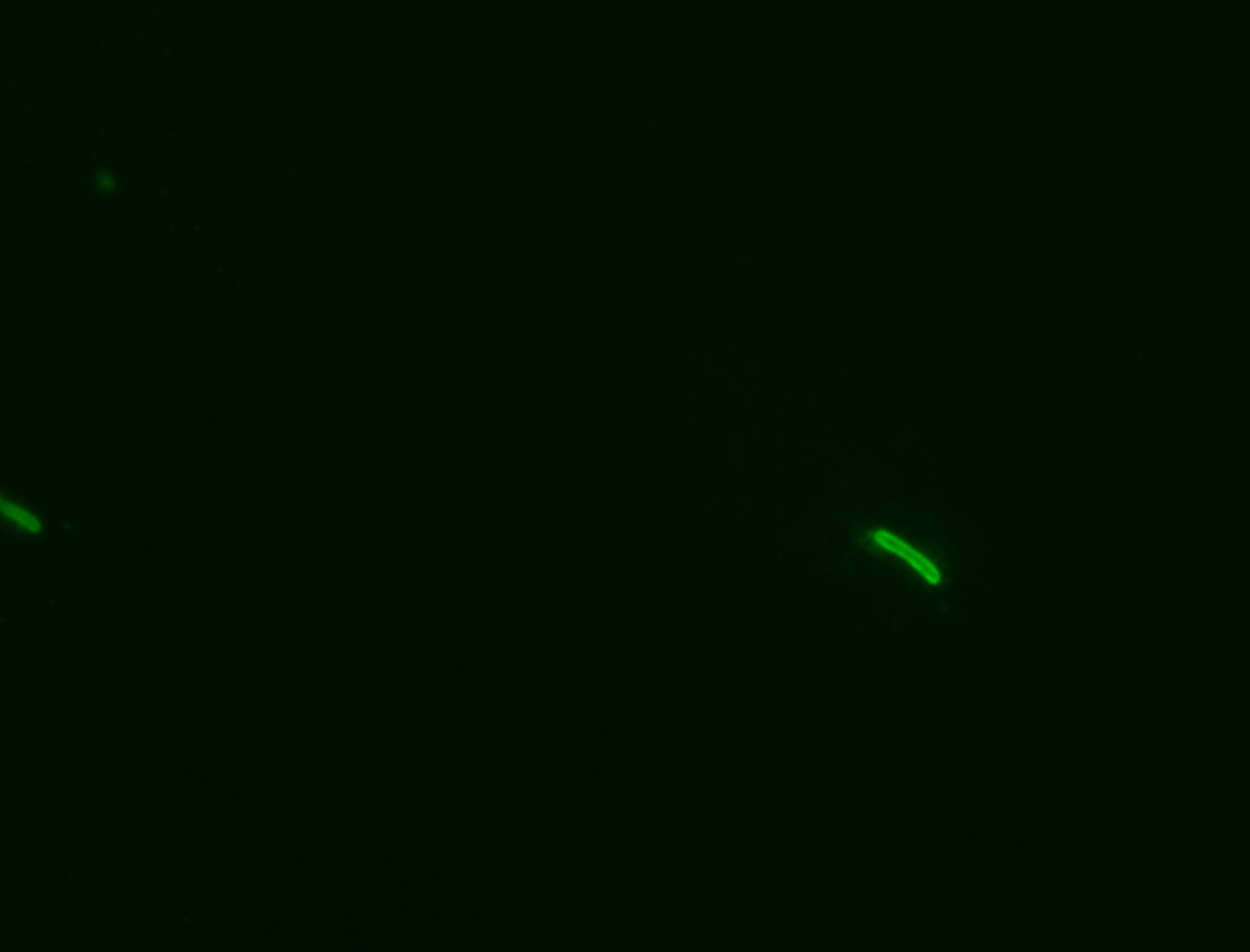

Supplement: Figure 7—figure supplement 1—source data 1. [file elife-89002-fig7-figsupp1-data1.zip › 9h del dota 2-c1.tif]

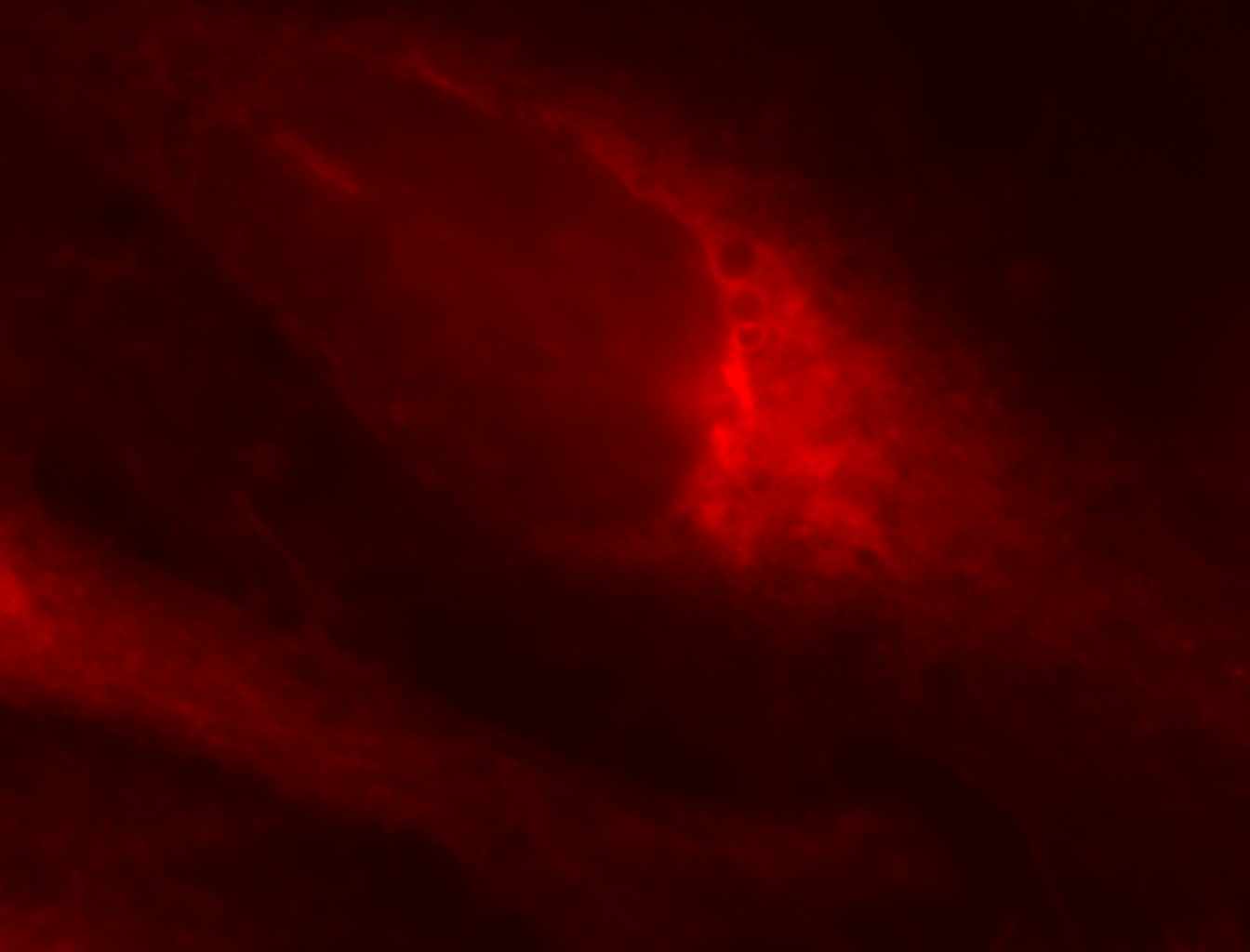

Supplement: Figure 7—figure supplement 1—source data 1. [file elife-89002-fig7-figsupp1-data1.zip › 9h del dota 2-c2.tif]

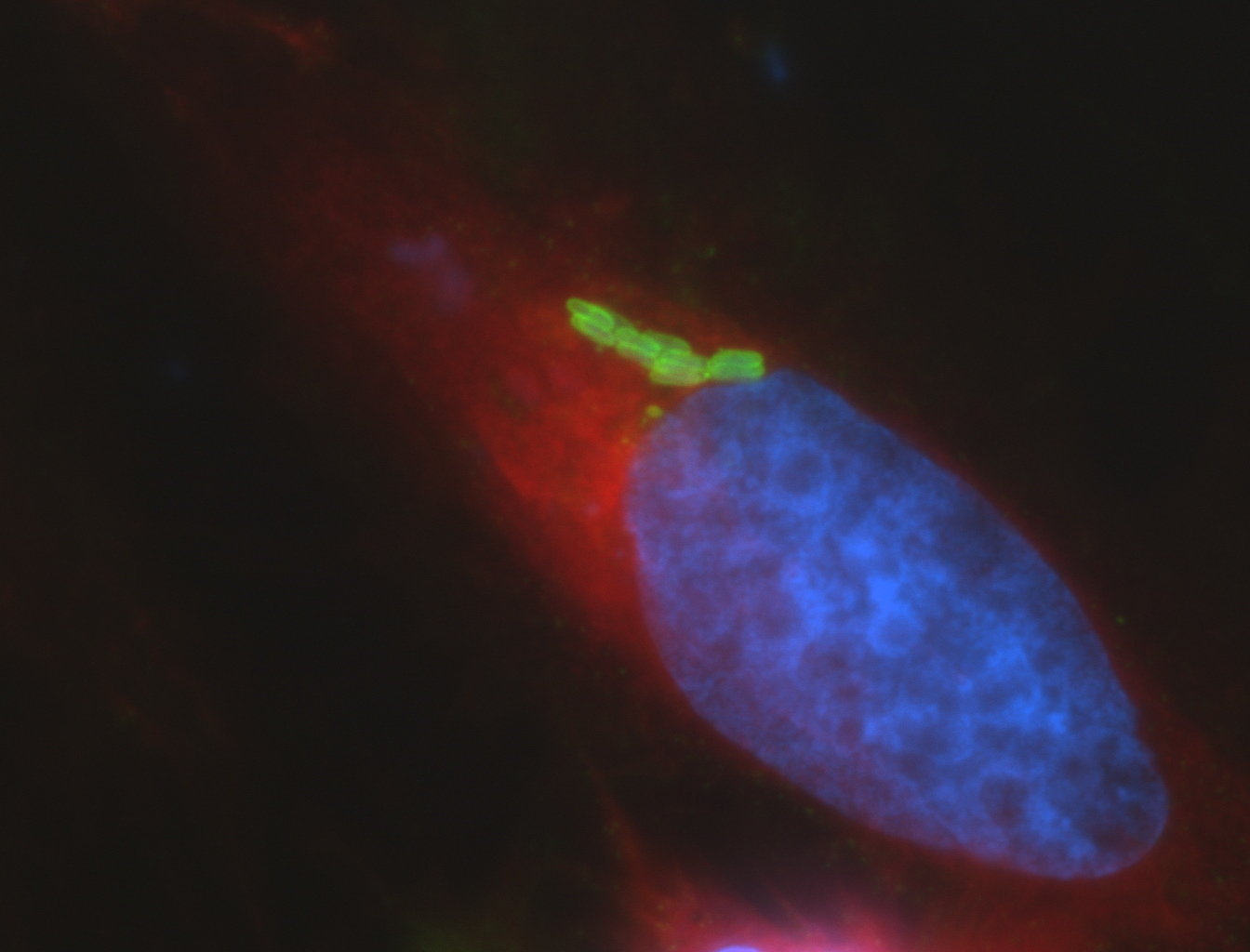

Supplement: Figure 7—figure supplement 1—source data 1. [file elife-89002-fig7-figsupp1-data1.zip › 9h Lp01 3+.tif]

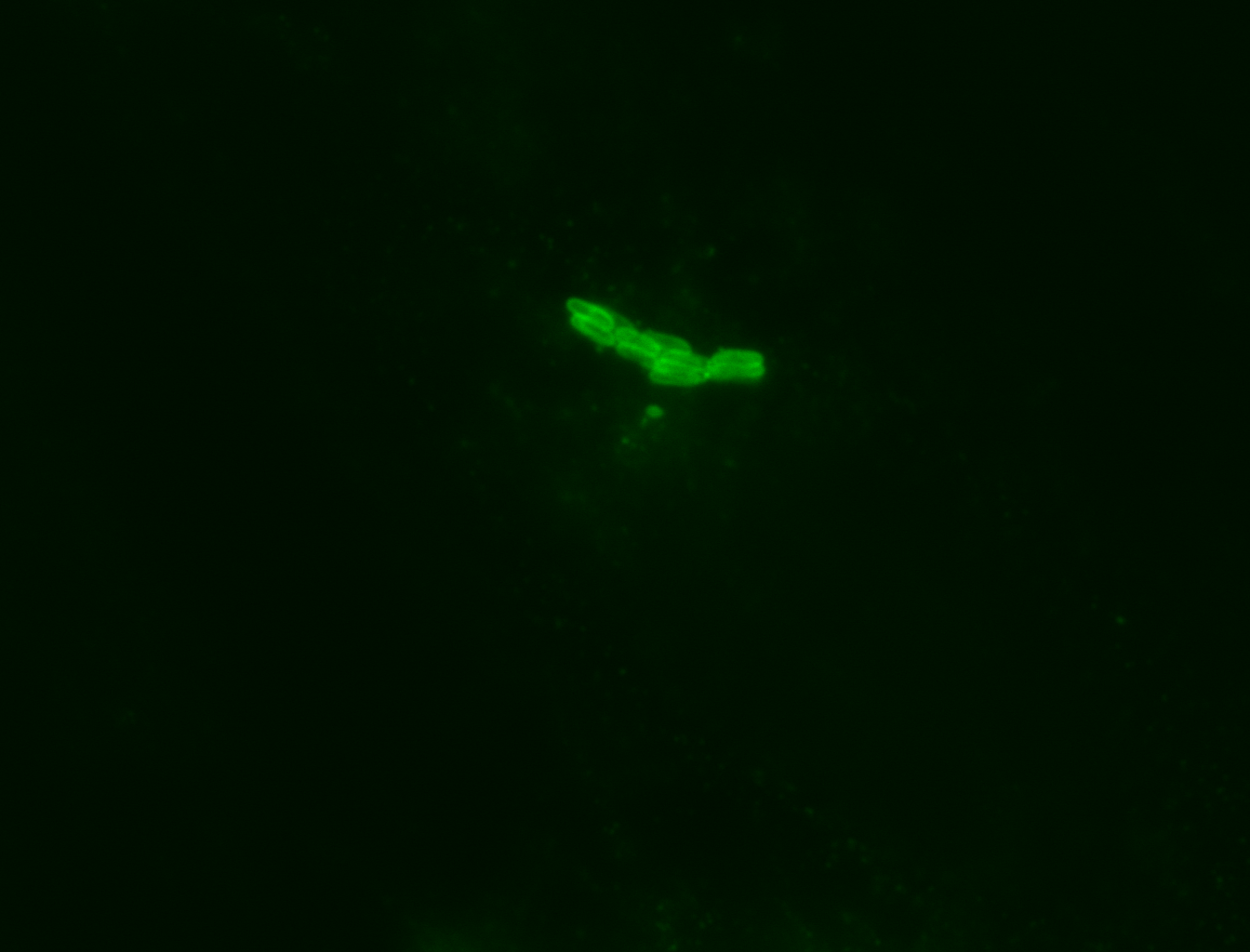

Supplement: Figure 7—figure supplement 1—source data 1. [file elife-89002-fig7-figsupp1-data1.zip › 9h lp01 3+c1.tif]

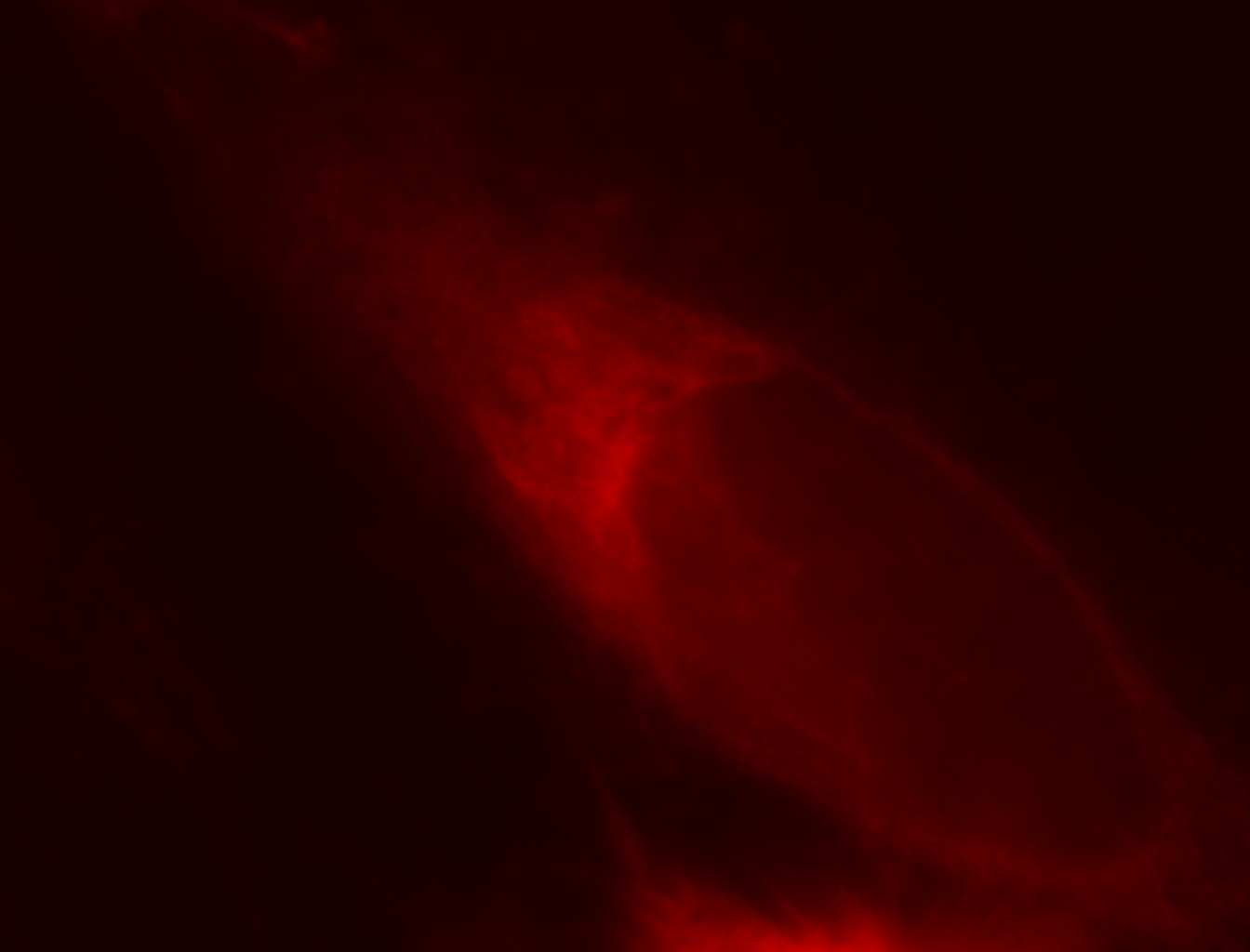

Supplement: Figure 7—figure supplement 1—source data 1. [file elife-89002-fig7-figsupp1-data1.zip › 9h lp01 3+c2.tif]

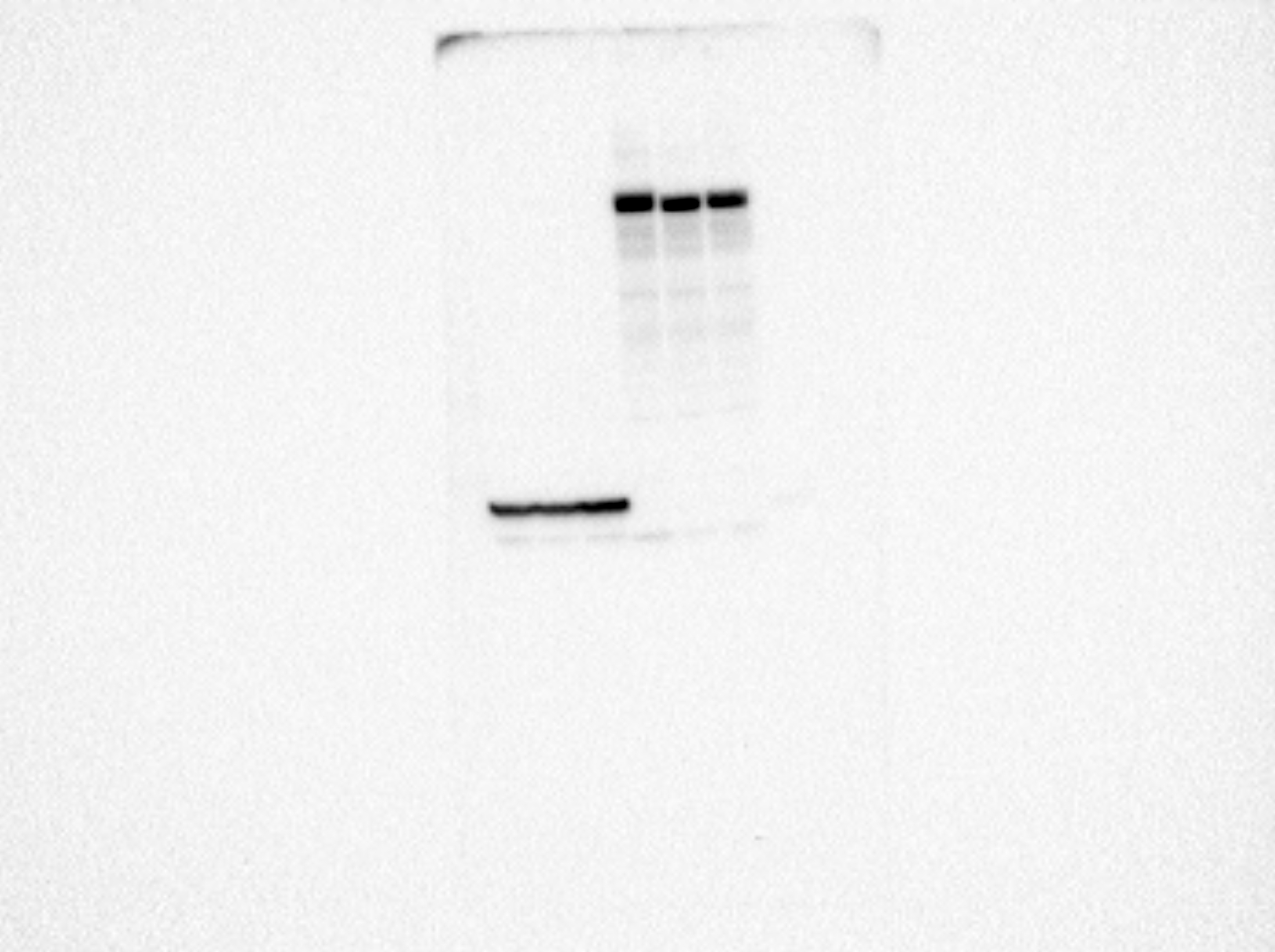

Supplement: Figure 7—figure supplement 2—source data 1. [file elife-89002-fig7-figsupp2-data1.zip › input anti-GFPrb_Exposure_28.9sec.jpg]

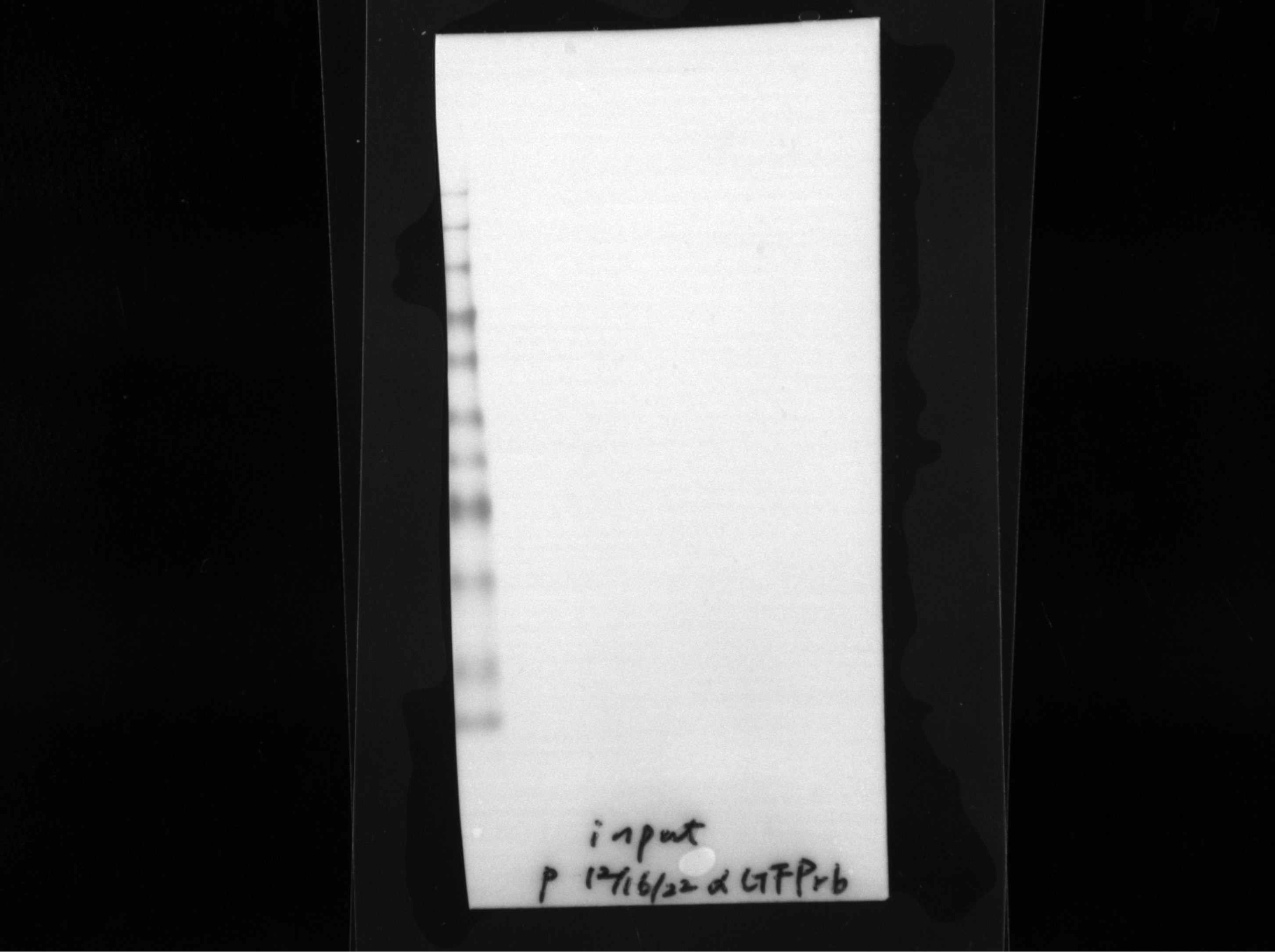

Supplement: Figure 7—figure supplement 2—source data 1. [file elife-89002-fig7-figsupp2-data1.zip › input anti-GFPrb_Marker.jpg]

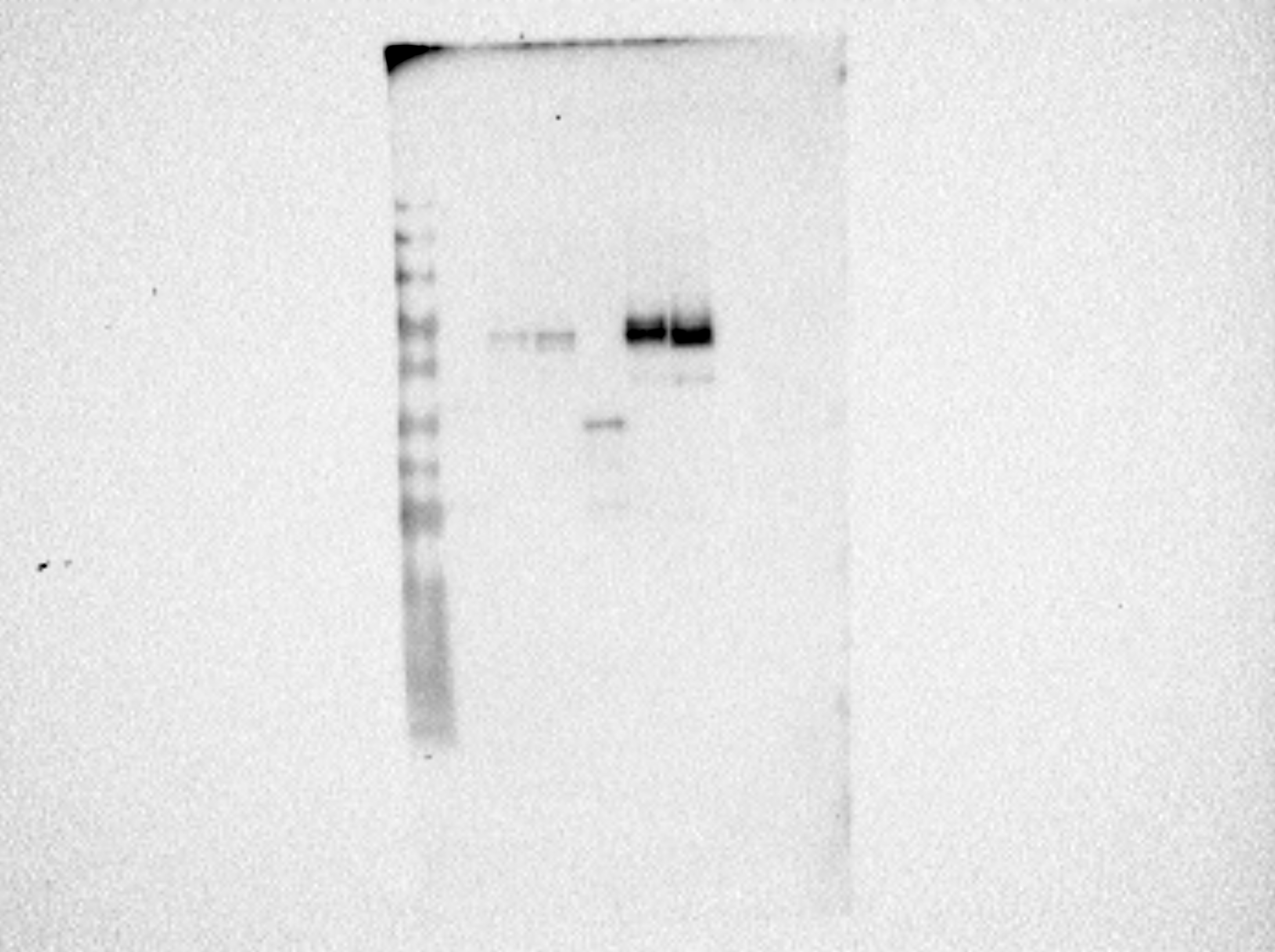

Supplement: Figure 7—figure supplement 2—source data 1. [file elife-89002-fig7-figsupp2-data1.zip › IP RFP anti-HArb_Exposure_60.0sec.jpg]

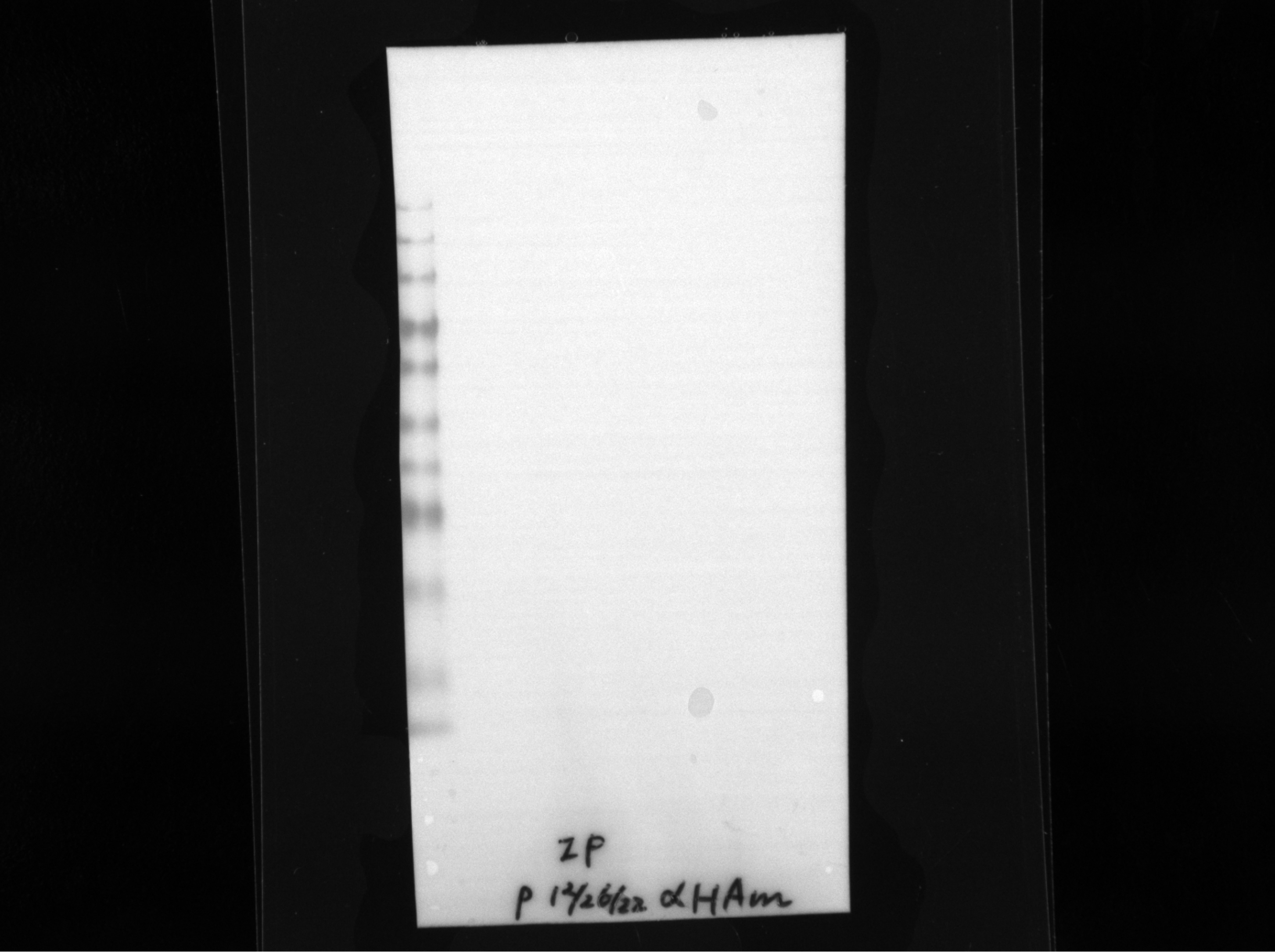

Supplement: Figure 7—figure supplement 2—source data 1. [file elife-89002-fig7-figsupp2-data1.zip › IP RFP anti-HArb_Marker.jpg]

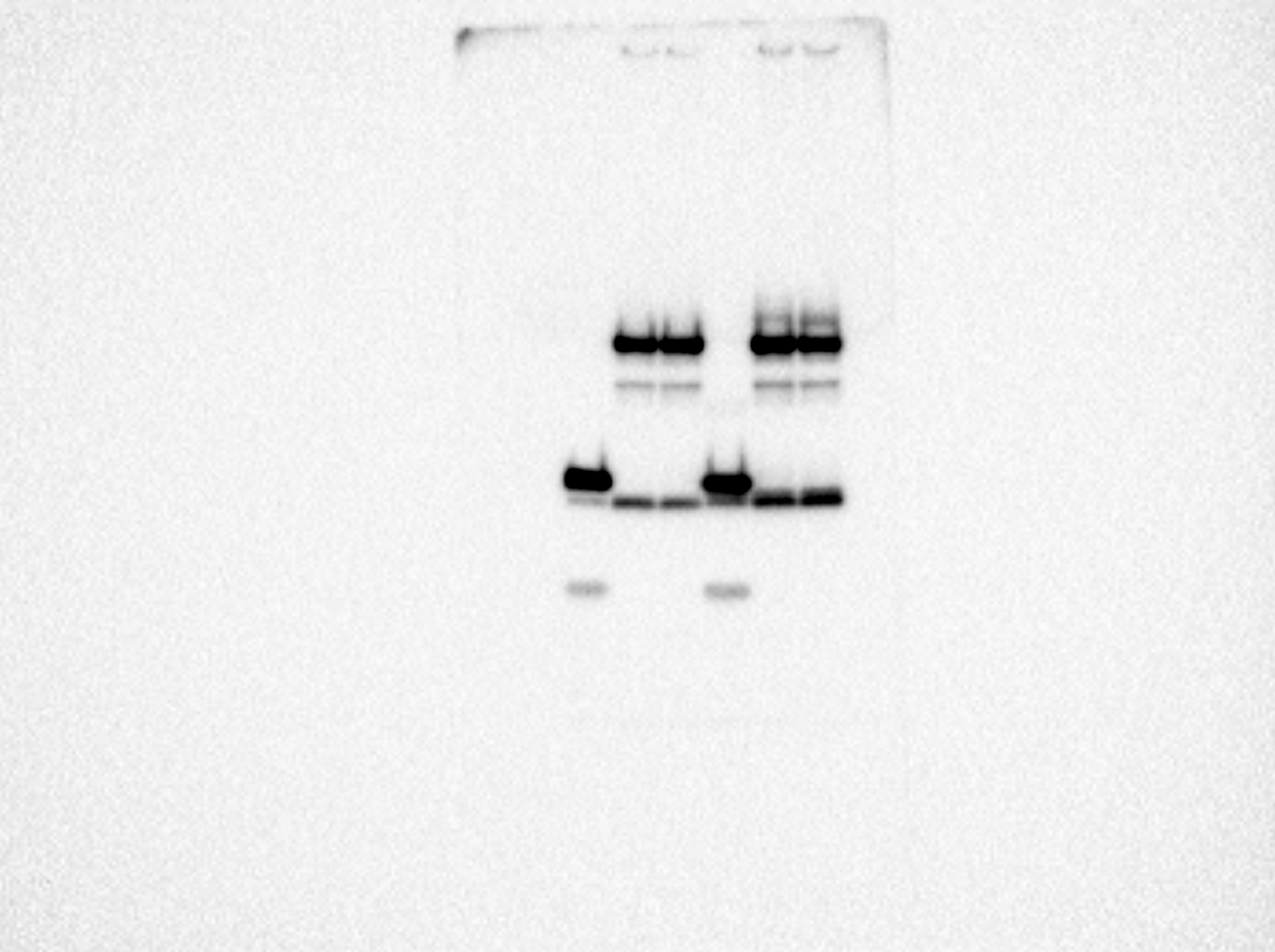

Supplement: Figure 7—figure supplement 2—source data 1. [file elife-89002-fig7-figsupp2-data1.zip › IP RFP IB RFPrb_Exposure_28.9sec.jpg]

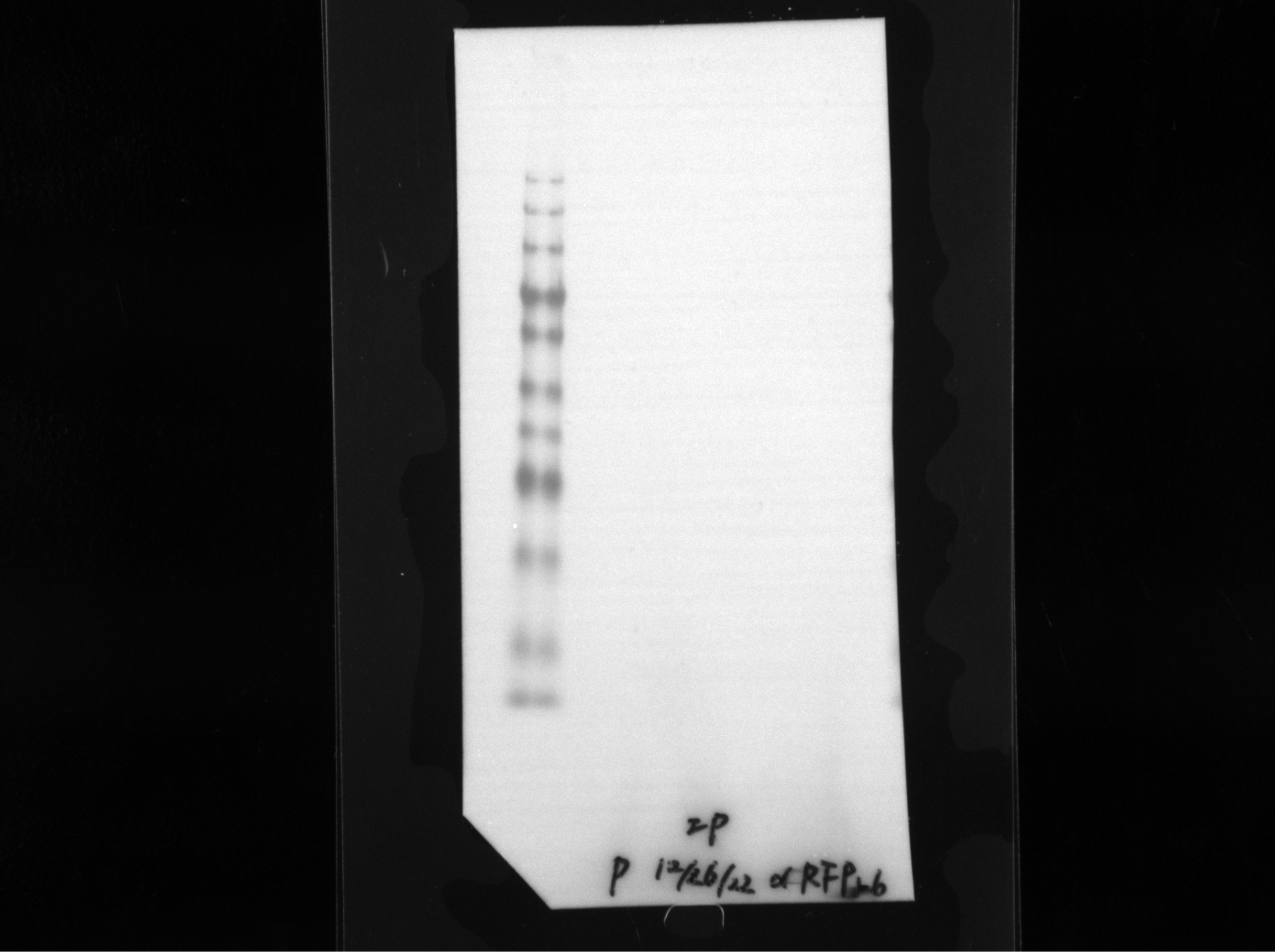

Supplement: Figure 7—figure supplement 2—source data 1. [file elife-89002-fig7-figsupp2-data1.zip › IP RFP IB RFPrb_Marker.jpg]

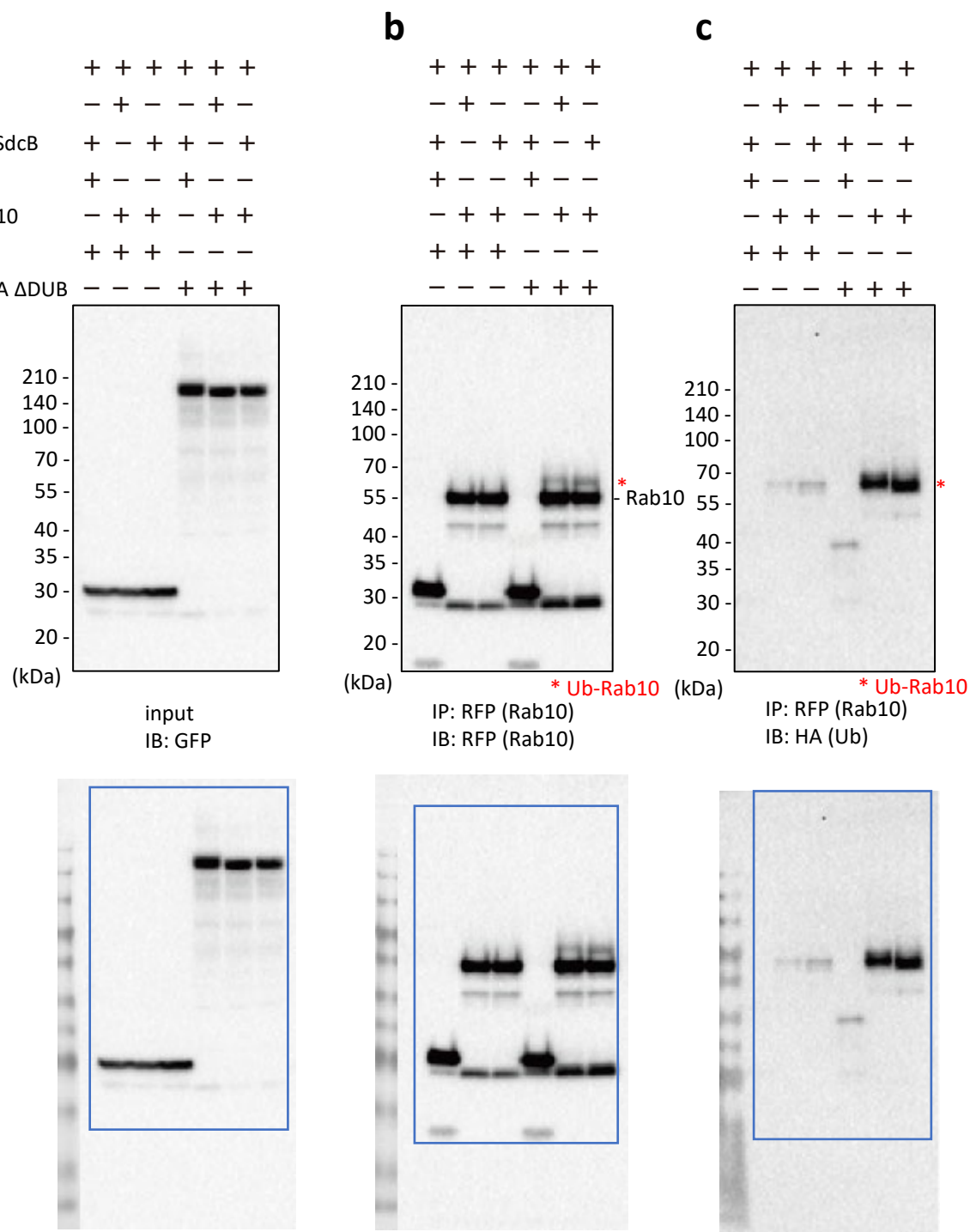

**Figure 7**  
– figure supplement 2a

**Figure 7**  
– figure supplement 2b

**Figure 7**  
– figure supplement 2c

Supplement: Figure 7—figure supplement 2—source data 2. [file elife-89002-fig7-figsupp2-data2.pdf]
