# Supplementary material for: Multi-tiered actions of Legionella effectors to modulate host Rab10 dynamics
Source: eLife. 2024 May 21;12:RP89002. doi: 10.7554/eLife.89002 (PMC11108646; doi:10.7554/eLife.89002)
Supplement: Figure 5—figure supplement 2—source data 2. [file elife-89002-fig5-figsupp2-data2.pdf]

a

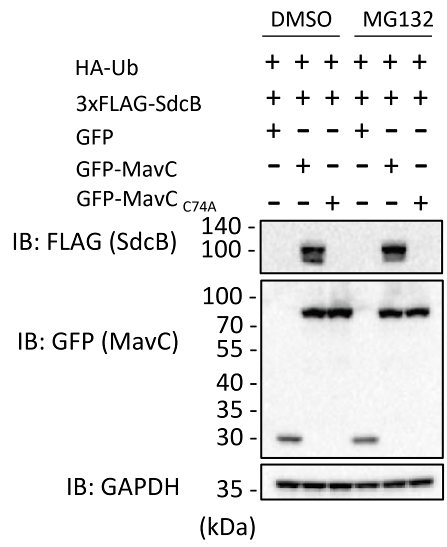

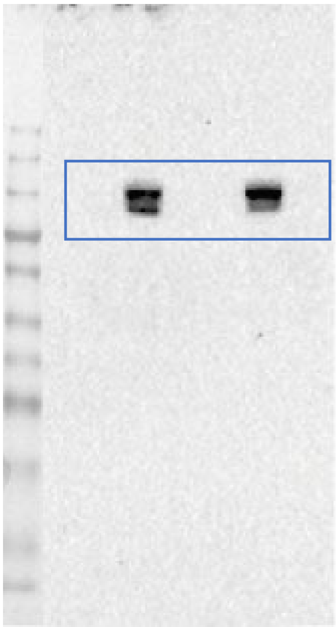

Figure 5
– figure supplement 2a
top

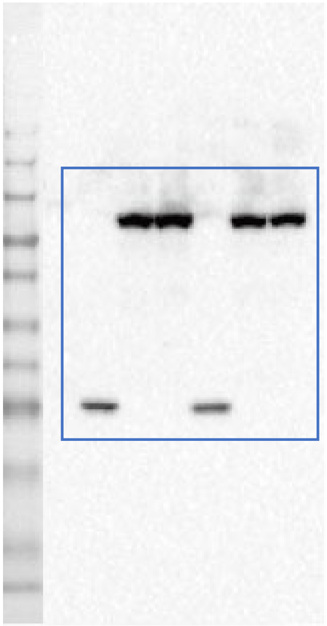

Figure 5
– figure supplement 2a
middle

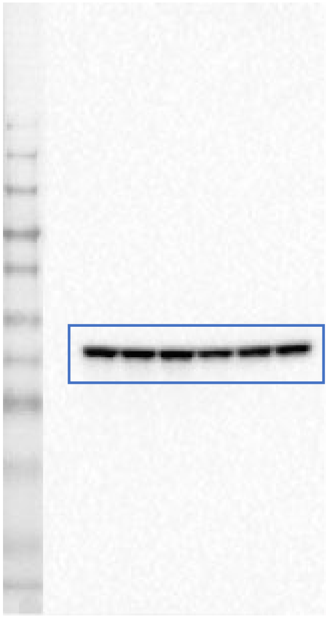

Figure 5
– figure supplement 2a
bottom
