# Supplementary material for: Multi-tiered actions of Legionella effectors to modulate host Rab10 dynamics
Source: eLife. 2024 May 21;12:RP89002. doi: 10.7554/eLife.89002 (PMC11108646; doi:10.7554/eLife.89002)
Supplement: Figure 6—figure supplement 2—source data 2. [file elife-89002-fig6-figsupp2-data2.pdf]

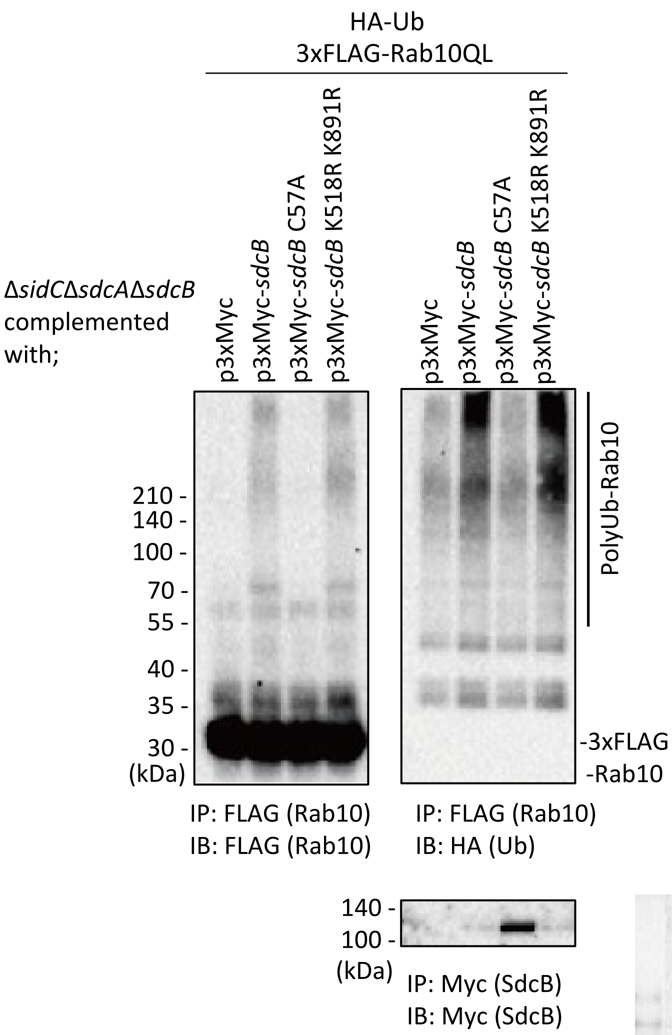

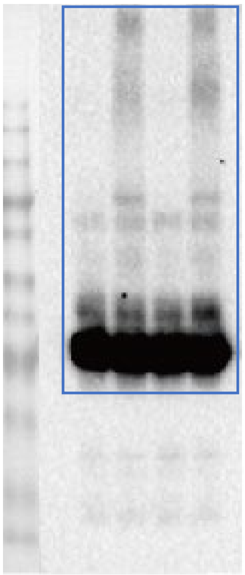

Figure 6
– figure supplement 2
left

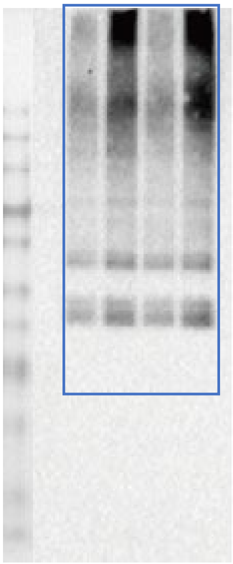

Figure 6
– figure supplement 2
right top

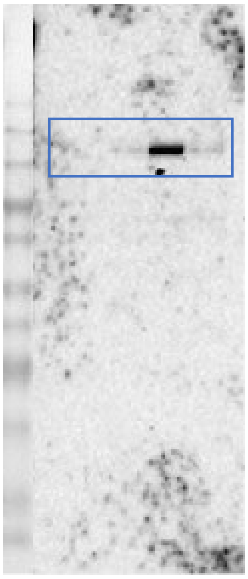

Figure 6
– figure supplement 2
right bottom
