# Supplementary material for: Multi-tiered actions of Legionella effectors to modulate host Rab10 dynamics
Source: eLife. 2024 May 21;12:RP89002. doi: 10.7554/eLife.89002 (PMC11108646; doi:10.7554/eLife.89002)
Supplement: Supplementary file 1. [file elife-89002-supp1.docx]

**Supplementary File 1.**

| **Plasmid ID** | **Plasmids** | **Genotype** | **Purpose** | **Reference or source** |
| --- | --- | --- | --- | --- |
| NA | pUC18 | Cloning vector, Amp^r^ | Cloning | TaKaRa |
| pNH2802 #2 | pUC18-*sdeA* | Cloning vector encoding SdeA, Amp^r^ | Cloning | This study |
| pNH2804 #2 | pUC18-*sdeA*_EE/AA_ | Cloning vector encoding SdeA catalytic E860A E862A mutant, Amp^r^ | Cloning | This study |
| NA | pHA-Ub | pHA encoding Ub, Amp^r^ | Transfection | Gift from Michinaga Ogawa |
| pNH2175 #2 | pHA-Ub_AA_ | pHA encoding Ub G75A G76A mutant, Amp^r^ | Transfection | This study |
| pNH2156 #1 | pHA-Ub_Q40E_ | pHA encoding Ub Q40E mutant, Amp^r^ | Transfection | This study |
| pNH2276 #1 | pHA-Ub_Q31E_ | pHA encoding Ub Q31E mutant, Amp^r^ | Transfection | This study |
| pNH2277 #1 | pHA-Ub_Q41E_ | pHA encoding Ub Q41E mutant, Amp^r^ | Transfection | This study |
| pNH2288 #1 | pHA-Ub_Q31E Q41E_ | pHA encoding Ub Q31E Q41E mutant, Amp^r^ | Transfection | This study |
| NA | p3xHA-Ub | p3xHA encoding Ub, Amp^r^ | Transfection | Gift from Jiazhang Qiu |
| NA | p3xHA-Ub_AA_ | p3xHA encoding Ub G75A G76A mutant, Amp^r^ | Transfection | Gift from Jiazhang Qiu |
| NA | pHA-Ub_K6_ | pHA encoding Ub with Lys6 (without any other Lys residues), Amp^r^ | Cloning | Addgene #22900 |
| pNH2177 #1 | pHA-Ub_No K_ | pRK5-HA encoding Ub with no Lys residues, Amp^r^ | Transfection | This study |
| NA | pEGFP-C2 | GFP-tagged protein expression vector, Kan^r^ | Transfection | BD |
| NA | pmGFP | pEGFP-C2 encoding monomeric GFP (GFP with L222K mutation), Kan^r^ | Transfection | (Kitao et al., 2020) |
| pNH2123 #1 | pmGFP*-mavC* | pmGFP encoding MavC, Kan^r^ | Transfection | This study |
| pNH2140 #1 | pmGFP*-mavC*_C74A_ | pmGFP encoding MavC C74A mutant, Kan^r^ | Transfection | This study |
| NA | pET15b | hexahistidine-tagged protein expression vector, Amp^r^ | Protein purification | Novagen |
| pNH1989 #9 | pET15b-His-*sdeA* | pET15b encoding hexahistidine-tagged SdeA, Amp^r^ | Cloning | This study |
| NA | pET15b-His-*sdeA*_ΔDUB_ | pET15b encoding hexahistidine-tagged SdeA Δ1-199aa , Amp^r^ | Cloning | This study |
| pNH2000 #6 | pmGFP*-sdeA* | pmGFP encoding SdeA, Kan^r^ | Cloning | This study |
| NA | pmGFP*-sdeA*_ΔDUB_ | pmGFP encoding SdeA Δ1-199aa , Kan^r^ | Transfection | This study |
| NA | P3xFLAG-cDNA4T/O- | 3xFLAG-tagged protein expression vector, Amp^r^ | Transfection | (Ingmundson et al., 2007) |
| NA | p3xFLAG-Rab10 | p3xFLAG encoding human Rab10A, Amp^r^ | Transfection | This study |
| pNH2515 #1 | p3xFLAG-Rab10QL | p3xFLAG encoding human Rab10A Q68L, Amp^r^ | Transfection | This study |
| pNH2516 #1 | p3xFLAG-Rab10TN | p3xFLAG encoding human Rab10A T23N, Amp^r^ | Transfection | This study |
| pNH2518 #2 | p3xFLAG-Rab10KKK | p3xFLAG encoding human Rab10A K102A K136A K154A, Amp^r^ | Transfection | This study |
| NA | p3xFLAG-CMV-10 | 3xFLAG-tagged protein expression vector, Amp^r^ | Transfection | Sigma |
| pNH1807 #1 | p3xFLAG-*sidC* | p3xFLAG encoding SidC, Amp^r^ | Transfection | This study |
| pNH1808 #26 | p3xFLAG- *sidC* _C46A_ | p3xFLAG encoding SidC C46A mutant, Amp^r^ | Transfection | This study |
| pNH2172 #1 | p3xFLAG-*sdcA* | p3xFLAG encoding SdcA, Amp^r^ | Transfection | This study |
| pNH2173 #2 | p3xFLAG- *sdcA* _C44A_ | p3xFLAG encoding SdcA C44A mutant, Amp^r^ | Transfection | This study |
| pNH2109 #4 | p3xFLAG-*sdcB* | p3xFLAG encoding SdcB mutant, Amp^r^ | Transfection | This study |
| pNH2126 #6 | p3xFLAG- *sdcB* _C57A_ | p3xFLAG encoding SdcB C57A mutant, Amp^r^ | Transfection | This study |
| pNH2286 #1 | p3xFLAG- *sdcB* _K518R_ | p3xFLAG encoding SdcB K518R mutant, Amp^r^ | Transfection | This study |
| pNH2287 #1 | p3xFLAG- *sdcB* _K891R_ | p3xFLAG encoding SdcB K891R mutant, Amp^r^ | Transfection | This study |
| pNH2289 #3 | p3xFLAG- *sdcB* _K518R K891R_ | p3xFLAG encoding SdcB K518R K891R mutant, Amp^r^ | Transfection | This study |
| NA | pCMV-HA-N | N-terminal HA-tagged protein expression vector, Amp^r^ | Transfection | Clontech |
| pNH2272 # | pHA-*mavC* | pHA encoding MavC, Amp^r^ | Transfection | This study |
| pNH2273 #1 | pHA-*mavC* _C74A_ | pHA encoding MavC C74A mutant, Amp^r^ | Transfection | This study |
| NA | pmRFP-C1 | N-terminal RFP-tagged protein expression vector, Kan^r^ | Transfection | (Murata et al., 2006) |
| NA | pmRFP-Rab10 | pRFP encoding Rab10, Kan^r^ | Transfection | This study |
| pNH1804 #1 | pET15b-His-*sidC* | pET15b encoding hexahistidine-tagged SidC, Amp^r^ | Protein purification | This study |
| pNH2120 #6 | pET15b-His-*sdcB* | pET15b encoding hexahistidine-tagged SdcB, Amp^r^ | Protein purification | This study |
| pNH2121 #9 | pET15b-His-*sdcB* _C57A_ | pET15b encoding hexahistidine-tagged SdcB C57A mutant, Amp^r^ | Protein purification | This study |
| pNH2142 #1 | pET15b-His-*mavC* | pET15b encoding hexahistidine-tagged MavC, Amp^r^ | Protein purification | This study |
| pNH2143 #5 | pET15b-His-*mavC* _C74A_ | pET15b encoding hexahistidine-tagged MavC C74A mutant, Amp^r^ | Protein purification | This study |
| pNH2144 #3 | pET15b-His-*mvcA* | pET15b encoding hexahistidine-tagged MvcA, Amp^r^ | Protein purification | This study |
| NA | pSR47S | oriR6K, *oriT* RP4, *Kan^r^*, *sacB* | Gene deletion | (Merriam et al., 1997) |
| pNH1990 #1 | pSR47S-Δ*sidE* | pSR47S carrying 30bp upstream and downstream regions of *sidE,* Kan^r^ | Gene deletion | This study |
| pNH1993 #1 | pSR47S-Δ*sdeA*Δ*sdeB* | pSR47S carrying 300bp upstream and downstream regions of *sdeA-sdeB,* Kan^r^ | Gene deletion | This study |
| pNH1994 #2 | pSR47S-Δ*sdeC* | pSR47S carrying 300bp upstream and downstream regions of *sdeC,* Kan^r^ | Gene deletion | This study |
| pNH2213 #1 | pSR47S-Δ*dupA* | pSR47S carrying 300bp upstream and downstream regions of *dupA,* Kan^r^ | Gene deletion | This study |
| pNH2196 #1 | pSR47S-Δ*dupB* | pSR47S carrying 300bp upstream and downstream regions of *dupB,* Kan^r^ | Gene deletion | This study |
| pNH2214 #1 | pSR47S-Δ*dupA*Δ*sidJ* | pSR47S carrying 300bp upstream and downstream regions of *dupA-sidJ,* Kan^r^ | Gene deletion | This study |
| pNH2197 #1 | pSR47S-Δ*dupB*Δ*sdjA* | pSR47S carrying 300bp upstream and downstream regions of *dupB-sdj,* Kan^r^ *A* | Gene deletion | This study |
| pNH1803 #1 | pSR47S-Δ*sidC*Δ*sdcA* | pSR47S carrying 300bp upstream and downstream regions of *sidC-sdcA,* Kan^r^ | Gene deletion | This study |
| pNH2111 #2 | pSR47S-Δ*sdcB* | pSR47S carrying 300bp upstream and downstream regions of *sdc,* Kan^r^ *B* | Gene deletion | This study |
| pNH2139 #1 | pSR47S-Δ*lpg2149* | pSR47S carrying 300bp upstream and downstream regions of *sdcB*, Kan^r^ | Gene deletion | This study |
| pNH2136 #3 | pSR47S-Δ*mavC*Δ*mvcA* | pSR47S carrying 300bp upstream and downstream regions of *mavC-mvcA,* Kan^r^ | Gene deletion | This study |
| NA | pMMB207NT | Cloning vector with P*icmR* derived from RSF1010 (*oriR*), Cm^r^ | Expression vector for *Legionella* | (Coers et al., 2000) |
| pNH1884 #10 | pMMB207NT-3xFLAG | pMMB207NT to express 3xFLAG-tagged protein, Cm^r^ | Expression vector for *Legionella* | (Kubori et al., 2018) |
| pNH2117 #1 | pMMB207-3xFLAG-*sdcB* | pMMB207NT encoding 3xFLAG-tagged SdcB, Cm^r^ | Expression in *Legionella* | This study |
| pNH2118 #3 | pMMB207-3xFLAG-*sdcB_C57A_* | pMMB207NT encoding 3xFLAG-tagged SdcB C57A mutant, Cm^r^ | Expression in *Legionella* | This study |
| NA | pMMB207NT-3xMyc | pMMB207NT to express 3xMyc-tagged protein, Cm^r^ | Expression vector for *Legionella* | (Kubori et al., 2022) |
| NA | pMMB207NT-3xMyc-*sdeA* | pMMB207NT to express 3xMyc-tagged SdeA, Cm^r^ | Expression in *Legionella* | This study |
| NA | pMMB207NT-3xMyc-*sdeA_EE/AA_* | pMMB207NT to express 3xMyc-tagged SdeA E860A E862A mutant, Cm^r^ | Expression in *Legionella* | This study |
| pNH2236 #3 | pMMB207NT-3xMyc- *sdcB* | pMMB207NT to express 3xMyc-tagged SdcB, Cm^r^ | Expression in *Legionella* | This study |
| pNH2237 #1 | pMMB207NT-3xMyc- *sdcB_C57A_* | pMMB207NT to express 3xMyc-tagged SdcB C57A mutant, Cm^r^ | Expression in *Legionella* | This study |
| pNH2511 #14 | pMMB207NT-3xMyc- *sdcB_K518R K891R_* | pMMB207NT to express 3xMyc-tagged SdcB K518R K891R mutant, Cm^r^ | Expression in *Legionella* | This study |

**References**

Coers J, Kagan JC, Matthews M, Nagai H, Zuckman DM, Roy CR. 2000. Identification of Icm protein complexes that play distinct roles in the biogenesis of an organelle permissive for Legionella pneumophila intracellular growth. *Mol Microbiol* **38**:719–736. doi:10.1046/j.1365-2958.2000.02176.x

Ingmundson A, Delprato A, Lambright DG, Roy CR. 2007. Legionella pneumophila proteins that regulate Rab1 membrane cycling. *Nature* **450**:365–369. doi:10.1038/nature06336

Kitao T, Taguchi K, Seto S, Arasaki K, Ando H, Nagai H, Kubori T. 2020. Legionella Manipulates Non-canonical SNARE Pairing Using a Bacterial Deubiquitinase. *Cell Rep* **32**. doi:10.1016/j.celrep.2020.108107

Kubori T, Kitao T, Ando H, Nagai H. 2018. LotA, a Legionella deubiquitinase, has dual catalytic activity and contributes to intracellular growth. *Cell Microbiol* **20**. doi:10.1111/cmi.12840

Kubori T, Lee J, Kim H, Yamazaki K, Nishikawa M, Kitao T, Oh B-H, Nagai H. 2022. Reversible modification of mitochondrial ADP/ATP translocases by paired Legionella effector proteins. *Proceedings of the National Academy of Sciences* **119**. doi:10.1073/pnas.2122872119

Merriam JJ, Mathur R, Maxfield-Boumil R, Isberg RR. 1997. Analysis of the Legionella pneumophila fliI Gene: Intracellular Growth of a Defined Mutant Defective for Flagellum Biosynthesis, INFECTION AND IMMUNITY. doi:10.1128/iai.65.6.2497-2501.1997

Murata T, Delprato A, Ingmundson A, Toomre DK, Lambright DG, Roy CR. 2006. The Legionella pneumophila effector protein DrrA is a Rab1 guanine nucleotide-exchange factor. *Nat Cell Biol* **8**:971–977. doi:10.1038/ncb1463
