# Supplementary material for: Multi-tiered actions of Legionella effectors to modulate host Rab10 dynamics
Source: eLife. 2024 May 21;12:RP89002. doi: 10.7554/eLife.89002 (PMC11108646; doi:10.7554/eLife.89002)
Supplement: Supplementary file 2. [file elife-89002-supp2.docx]

**Supplementary File 2.**

| **Primers** | | |
| --- | --- | --- |
| **ID** | **Name** | **Sequence (5' to 3')** |
| 2100 | sdeA f (BamHI) | gcGGATCCagatgcctaagtatgtcgaaggg |
| 2101 | sdeA r (XbaI) | gcTCTAGAttaaaatcctatagtttttttattgg |
| 2104 | sdeA E860A E862A f | gcgaaggcaccGCAagtGCAttctccg |
| 2105 | sdeA E860A E862A r | cggagaaTGCactTGCggtgccttcgc |
| 2548 | Ub GG75-76AA f | GTTGAGACTTCGTGCTGCTTAACTCGAGCATG |
| 2549 | Ub GG75-76AA r | CATGCTCGAGTTAAGCAGCACGAAGTCTCAAC |
| 2329 | Ub Q40E f | CCTCCTGATGAGCAGAGACTGATC |
| 2330 | Ub Q40E r | GTCTCTGCTCATCAGGAGGTATTC |
| 2935 | Ub Q31E f | GGCCAAGATCGAGGATAAGGAAGG |
| 2936 | Ub Q31E r | CCTTCCTTATCCTCGATCTTGGCC |
| 2937 | Ub Q41E f | CCTCCTGATCAGGAGAGACTGATC |
| 2938 | Ub Q41E r | GATCAGTCTCTCCTGATCAGGAGG |
| 2576 | pRK5-HA-Ub K6R f | gatcttcgtcagaacgttaacc |
| 2577 | pRK5-HA-Ub K6R r | ggttaacgttctgacgaagatc |
| NA | Rab10 f (SmaI) | TCCCCCGGGCATGGCGAAGAAGACGTACGA |
| NA | Rab10 r (SmaI) | TCCCCCGGGTCAGCAGCATTTGCTCTTCC |
| 3015 | Rab10 Q68L f | GGGATACAGCAGGCCTGGAGCGATTTCAC |
| 3016 | Rab10 Q68L r | GTGAAATCGCTCCAGGCCTGCTGTATCCC |
| 3017 | Rab10 T23N f | GGAGTGGGGAAGAACTGCGTCCTTTTTCG |
| 3018 | Rab10 T23N r | CGAAAAAGGACGCAGTTCTTCCCCACTCC |
| 3007 | Rab10 K102A f | GAAAACATCAGCGCATGGCTTAGAAAC |
| 3008 | Rab10 K102A r | GTTTCTAAGCCATGCGCTGATGTTTTC |
| 3009 | Rab10 K136A f | CCTAAAGGAGCAGGAGAACAGATTGC |
| 3010 | Rab10 K136A r | GCAATCTGTTCTCCTGCTCCTTTAGG |
| 3011 | Rab10 K154A f | CTAGTGCAGCAGCAAATATAAACATC |
| 3012 | Rab10 K154A r | GATGTTTATATTTGCTGCTGCACTAG |
| 2217 | mavC f (XhoI) | gcctcgagcATGacaacttccaagcttg |
| 2218 | mavC r (PstI) | gcgctgcagTCActtatcacgaagaactaacc |
| 2259 | mavC C74A f | ccacaaacagcgccggaaaagcg |
| 2260 | mavC C74A r | cgcttttccggcgctgtttgtgg |
| 1848 | sdeA f (XhoI) | gcCTCGAGatgcctaagtatgtcgaaggg |
| 1849 | sdeA r (BamHI) | gcGGATCCttaaaatcctatagtttttttattgg |
| 2712 | pET15b_sdeAΔDUB_F | GCCGCGCGGCAGCCATCCTACACTGGTTCTTCCTGGA |
| 2649 | pET15b_GA1 | ATGGCTGCCGCGCGGCAC |
| 1840 | sdeA f (PstI) | cgCTGCAGtATGcctaagtatgtcgaaggg |
| 2714 | pmGFP-sdeAΔDUB_F | TCCGGCCGGACTTGTACCTACACTGGTTCTTCCTGGA |
| 2715 | pmGFP-C2 GA1 | CCGGCCGGACTTGTACAGCTCGTC |
| 2574 | sidC f (KpnI) | ccgGGTACCaATGgtgataaacatggttgacg |
| 1397 | sidC r (BamHI) | gaGGATCCctatttctttataattcccgtg |
| 1408 | sidC C46A f | gataataccgcccaaacagcagttg |
| 1409 | sidC C46A r | caactgctgtttgggcggtattatc |
| 2575 | sdcA f (KpnI) | ccgGGTACCaATGaacatggttgacaaaataaaattc |
| 2222 | sdcA r (BamHI) | gcggatccCTAtattgtattcctaacag |
| 2579 | sdcA C44A f | gataataccgctgaaacaacaggtgagttattaacc |
| 2580 | sdcA C44A r | ctcacctgttgtttcagcggtattatccagtcctatttc |
| 2186 | sdcB f (EcoRI) | cgGAATTCaTTGaaagaccaattagcc |
| 2165 | sdcB r (BamHI) | GCGGATCCCTAagccagtttattggatatttc |
| 2743 | sdcB f (BamHI) | GCGGATCCTCTTGAAAGACCAATTAGCC |
| 2744 | sdcB r (SalI) | CGCGTCGACCTAagccagtttattggatatttc |
| 2200 | sdcB C57A f | ggcgataatacggccaaatcggatttag |
| 2201 | sdcB C57A r | ctaaatccgatttggccgtattatcgcc |
| 2951 | sdcB K518R f | GCTGAAGCTGTGGCGTCAAGAGTTCGCTACCTG |
| 2952 | sdcB K518R r | CAGGTAGCGAACTCTTGACGCCACAGCTTCAGC |
| 2953 | sdcB K891R f | GTTTTCTTTTCTGGCAGAGAAAATATAAAAACAGAC |
| 2954 | sdcB K891R r | GTCTGTTTTTATATTTTCTCTGCCAGAAAAGAAAAC |
| 2919 | mavC f (SalI) | cggtcgaccATGacaacttccaagcttg |
| 2306 | mavC r (XhoI) | gcctcgagTCActtatcacgaagaactaacc |
| 1396 | sidC f (XhoI) | gcCTCGAGgtgataaacatggttgacg |
| 1397 | sidC r (BamHI) | gaGGATCCctatttctttataattcccgtg |
| 2207 | sdcB f (NdeI) | gctgCATATGTTGaaagaccaattagcc |
| 2278 | mavC f (XhoI) | gcctcgagATGacaacttccaagcttg |
| 2279 | mavC r (BamHI) | gcggatccTCActtatcacgaagaactaacc |
| 2280 | mvcA f (XhoI) | gcctcgagATGacaaaaataaaactggaatcg |
| 2281 | mvcA r (BamHI) | gcggatccTTAgcttggcccctttttatacc |
| 1850 | sidE d1 (SacI) | gcGAGCTCctaattcaaacaggacttac |
| 1851 | sidE d2 | ggaagataaagaattaCATtacacaactcctg |
| 1852 | sidE d3 | tgtgtaATGtaattctttatcttccacgg |
| 1853 | sidE d4 (XbaI) | GCTCTAGActttgagaacatcacttcctg |
| 1858 | sdeB d1 (SacI) | gcGAGCTCccggaatccgaacgtac |
| 1862 | sdeBA d2 | taaggtcaattaCATtttactttctcccaagcg |
| 1863 | sdeBA d3 | gggagaaagtaaaATGtaattgaccttaaccc |
| 1857 | sdeA d4 (XbaI) | GCTCTAGAgaggtaatttcatctcaaag |
| 1983 | sdeC d1 (SacI) | gcGAGCTCcacaccaatcggaaggtttc |
| 1984 | sdeC d2 | aaaaaatactccttaCATtttactttctcccaaac |
| 1985 | sdeC d3 | gggagaaagtaaaATGtaaggagtattttttaagtg |
| 1986 | sdeC d4 (XbaI) | GCTCTAGAgattgtattcccattccgg |
| 2963 | dupA d1 (SacI) | gcGAGCTCGTTTCTCATCAAGCTGCTGC |
| 2964 | dupA d2 | GCCTCATAACCTCTTCACATGGTATGAGCCAAACC |
| 2965 | dupA d3 | GGTTTGGCTCATACCATGTGAAGAGGTTATGAGGC |
| 2696 | dupA d4 (XbaI) | GCTCTAGAGGTTGTGCTTGTAGTCG |
| 2684 | dupB d1 (SacI) | gcGAGCTCCTGATGTGTTGGAAGTGGCGG |
| 2685 | dupB d2 | ACTTTAAATAAAACAGGCTACATCGTATGAGCTAACCC |
| 2686 | dupB d3 | GGGTTAGCTCATACGATGTAGCCTGTTTTATTTAAAG |
| 2687 | dupB d4 (XbaI) | GCTCTAGAcaactcaagtctggtatcctc |
| 2697 | dupA sidJ d2 | ATTCGTTTTATCACATGGTATGAGCCAAACC |
| 2698 | dupA sidJ d3 | GGTTTGGCTCATACCATGTGATAAAACGAATACCC |
| 2699 | sidJ d4 (XbaI) | GCTCTAGACTTTCTCCCAAGCGAATATTTG |
| 2688 | dupB sdjA d2 | ACAATTGAAATGACCCTTTCACATCGTATGAGCTAACCC |
| 2689 | dupB sdjA d3 | GGGTTAGCTCATACGATGTGAAAGGGTCATTTCAATTG |
| 2690 | sdjA d4 (XbaI) | GCTCTAGAcaataagatattattgaccg |
| 1384 | sdcA d1 (XbaI) | GCTCTAGAataataggcacaatggtctcc |
| 1385 | sdcAsidC d2 | gatttcactcttacCTAcatcaccctatgc |
| 1386 | sdcAsidC d3 | agggtgatgTAGgtaagagtgaaatcactg |
| 1387 | sidC d4 (XbaI) | GCTCTAGAgaggacgtttgggctgaggag |
| 2196 | sdcB d1 (SacI) | GCGAGCTCgtatgggtatagcatcagag |
| 2197 | sdcB d2 | atccagCTACAAtttcgttatactctttc |
| 2198 | sdcB d3 | gtataacgaaaTTGTAGctggatagtttaaccg |
| 2199 | sdcB d4 (XbaI) | GCTCTAGAggattgtctttggacaggatg |
| 2272 | lpg2149 d1 (SacI) | gcGAGCTCctgaaattaaccctgaattggc |
| 2273 | lpg2149 d2 | cctcatttcaaTTACATaaatcaaactccttcg |
| 2274 | lpg2149 d3 | ttgatttATGTAAttgaaatgaggatgggg |
| 2275 | lpg2149 d4 (XbaI) | GCTCTAGAcgctgtcaaatcttgtagg |
| 2263 | mavC d1 (SacI) | gcGAGCTCcaacacagggtatgaatagc |
| 2264 | mavC mvcA d2 | gatttttattgttttattgaTTACATattaacctcactgatag |
| 2265 | mavC mvcA d3 | cagtgaggttaatATGTAAtcaataaaacaataaaaatc |
| 2266 | mvcA d4 (XbaI) | GCTCTAGAgtccatggcatcaagcgc |
| 2204 | sdcB f (BamHI) | GCGGATCCtcTTGaaagaccaattagcc |
| 2205 | sdcB r (XbaI) | GCTCTAGACTAagccagtttattggatatttc |
| 2341 | pMMB207-PicmR_2 | AAGCTTGGCTGTTTTGGCGGATG |
| 2681 | pMMB207-PicmR-3xmyc_GA1 | ACCCAGATCTTCTTCAGAGATGAGCTTCTGTTCACCTAA |
| 2658 | pMMB207-3xMyc-SdeA_F | TGAAGAAGATCTGGGTATGCCTAAGTATGTCGAAGGG |
| 2659 | pMMB207-3xMyc-SdeA_R | CAAAACAGCCAAGCTTTTAAAATCCTATAGTTTTTTTATTGGATTC |
